# Effectiveness of leader-targeted stress management interventions: A systematic review and meta-analysis

**DOI:** 10.5271/sjweh.4219

**Published:** 2025-07-01

**Authors:** Indra Dannheim, Helena Ludwig-Walz, Halina Kirsch, Martin Bujard, Anette E Buyken, Katherine M Richardson, Anja Kroke

**Affiliations:** 1 Regional Innovative Centre of Health and Quality of Live Fulda (RIGL), Fulda University of Applied Sciences, Fulda, Germany.; 2 Department of Nutritional, Food and Consumer Sciences, Fulda University of Applied Sciences, Fulda, Germany.; 3 Federal Institute for Population Research (BiB), Wiesbaden, Germany.; 4 Institute of Medical Psychology, Medical Faculty, University Heidelberg, Heidelberg, Germany.; 5 Institute of Nutrition, Consumption and Health, Faculty of Natural Sciences, Paderborn University, Paderborn, Germany.; 6 Department of Management & Management Science, Lubin School of Business, Pace University, New York, USA.

**Keywords:** evidence-based workplace health promotion, healthy leadership, occupational health, program evaluation

## Abstract

**Objective:**

Based on the well-documented role of supervisors' in fostering healthy workplaces and managing the impact of work-related stress, the aim of this study was to determine the effectiveness of leader-targeted stress management interventions (SMI) on their psychological stress, mindfulness, mental health, and work- and leadership-related outcomes.

**Methods:**

Eligible studies, including randomized controlled trials or controlled before–after studies, examining the effects of leader-targeted SMI on supervisors' psychological stress, mindfulness, mental health, and work- and leadership-related outcomes, were identified in four electronic databases and supplemented by manual search strategies. Screening for eligibility, data extraction, risk of bias assessment, and certainty of evidence grading, following PRISMA guidelines and Cochrane Handbook recommendations, were done in duplicate. Data were pooled in random effects models to synthesize g-scores. Sensitivity and moderator analyses were used to assess the robustness of the results and explore potential sources of heterogeneity.

**Results:**

The 25 studies (N=2466 participants) meeting the full inclusion criteria varied widely in population characteristics, intervention types, duration, delivery methods, and examined outcomes. The overall intervention effect was g=0.13 [95% confidence interval (CI) -0.24– -0.01] after excluding outliers. Significant intervention effects were found for mental health [g=-0.38 (95% CI -0.69– -0.08)] and, after excluding influential cases, work- [g=-0.32 (95% CI -0.63– -0.00)] and leadership-related outcomes [g=-0.23 (95% CI -0.44– -0.02)].

**Conclusion:**

Our meta-analysis suggests that leader-targeted SMI can be an effective approach for promoting occupational health.

Creating healthy workplaces is a major task in today's occupational settings, especially considering the increasing economic burden associated with work-related stress and mental health issues (1–3). Supervisors have been shown to play an important role in creating and promoting healthy workplaces (4, 5). They are chiefly responsible for establishing and anchoring health-promoting structures and processes (6, 7), shaping the health-promoting design of the workplace through their behavior (8, 9), and serving as role models to their subordinates (8–11). Simultaneously, supervisors themselves are confronted with numerous stressors at work that may substantially increase the risk of negative health effects (12–14). Heavy deadlines, performance pressure, the need to manage multiple tasks simultaneously, and frequent disruptions are commonly reported stressors (7, 15). Survey data from approximately 20 000 employees in 2018 indicated that managers across various industries were significantly more likely to face higher job demands than employees without management responsibility (15).

Based on the well-documented role of supervisors' in workplaces and the challenge of coping with diverse work-related stressors, leader-targeted stress management interventions (SMI) have garnered increasing recognition (5, 16, 17). These interventions have been proposed as an effective measure to enhance and sustain workforce health (18, 19), including the health of supervisors themselves (20–22). SMI are broadly defined as activities or programs that an organization initiates to diminish work-related stressors or assist in mitigating the negative outcomes resulting from exposure to such stressors (23). These interventions can be categorized into cognitive-behavioral, relaxation, multimodal, or organization-focused initiatives as well as alternative approaches (24, 25).

The Transactional Stress Model (26) provides a valuable framework for understanding how leader-targeted SMI operate as it conceptualizes stress as a dynamic process arising from the interaction between an individual and their environment. According to this model, the way supervisors perceive and evaluate stressors, as well as their ability to apply effective coping mechanisms, determines their emotional and behavioral responses to stress. SMI targeted at supervisors align with this framework by equipping them with tools and strategies needed to appraise and manage work-related stressors more effectively, thereby enhancing their own well-being and enabling them to foster healthier workplace environments for their followers.

Along with the increasing application of leader-targeted SMI, investigations into their effectiveness have emerged. However, to date no systematic review comprehensively summarizes and evaluates the effectiveness of these interventions. Previous systematic reviews have predominantly focused on selected intervention types, such as mindfulness interventions (27–29), which have traditionally been part of SMI (30) or specific occupational settings like healthcare (31). Some reviews solely examined intervention effects at the follower level (32), while others have included all employees, regardless of their supervisory position (24). Furthermore, it remains unclear whether the effectiveness of SMI targeted at supervisors can be influenced by aspects such as intervention type, training setting, delivery method, or target group.

Therefore, the objective of this systematic review and meta-analysis was to identify and summarize the evidence on the effectiveness of leader-targeted SMI on supervisors' psychological stress (eg, job stress), mindfulness, mental health (eg, burnout, anxiety, depression), work-related outcomes (eg, absenteeism, productivity), and leadership-related outcomes (eg, leadership style, performance).

## Methods

This systematic review and meta-analysis is reported according to the Preferred Reporting Items for Systematic Reviews and Meta-analysis (PRISMA statement) (33, 34) [supplementary material (www.sjweh.fi/article/4219) S1] and adheres to the Cochrane Handbook for Systematic Reviews (34). The meta-analysis was registered on the International Prospective Register of Systematic Review (PROSPERO; CRD42023464101).

### Data sources

Four electronic databases (MEDLINE, PsycINFO, Cochrane Central, Web of Science) were searched for eligible studies until 20 September 2023 using peer-reviewed search strings according to the evidence-based checklist Peer Review of Electronic Searches (PRESS) (35). Detailed information on database-specific search terms is provided in supplementary material S2. To identify unpublished and ongoing trials, the following trial registers were searched: ClinicalTrials.gov, Trials Register of Promoting Health Interventions and WHO International Clinical Trials Registry Platform. Additionally, manual searches in Google Scholar and the Federal Institute for Occupational Safety and Health (BAuA) were performed as well as citation tracking of all included studies to check for eligible studies.

### Inclusion and exclusion criteria

Eligibility criteria were defined following the population-intervention-comparison-outcome-study (PICOS) design scheme (34).

*Population:* studies that enrolled supervisors as the target group, independently of gender, management level and number of employees. Studies focusing on supervisors, who are diagnosed with a mental disease were excluded as well as studies, which did not distinguish between employees and supervisors.

*Intervention:* studies comparing stress management interventions targeted at supervisors with a waitlist or passive control group, or with an active control group receiving an alternative intervention. Interventions that focused on improving supervisors' leadership skills, for example by improving employee-supervisors' relationships or staff empowerment were excluded.

*Comparison:* no intervention (including waitlist and passive control groups) or alternative intervention.

*Outcome:* studies with any measure of (i) psychological stress (eg, perceived stress, job stress), (ii) mindfulness, (iii) mental health (eg, burnout, anxiety, depression), (iv) work-related outcomes (eg, work performance, absenteeism, productivity) or (v) leadership-related outcomes (eg, transformational leadership, authentic leadership, health-oriented leadership) before and after the intervention. Outcomes i–iii had to be self-reported measures. Outcomes iv and v could be objective measures (eg, absenteeism) or rated by followers (eg, leadership style.

*Study design:* Randomized controlled trials (RCT), cluster-randomized controlled trials (cRCT) and controlled before–after studies (CBA) measuring outcomes both before and after the intervention.

### Study selection

**Figure 1:**
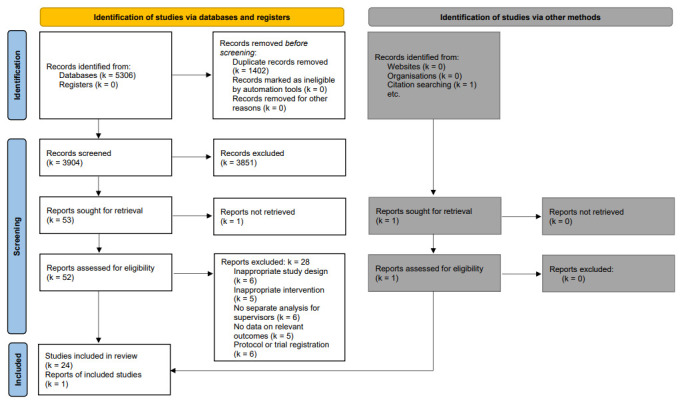
Study selection process (PRISMA Flow Chart).

Study selection followed a three-stage process. In a first step, eligible studies were imported into the EPPI reviewer software (36) and duplicates were automatically removed. In a second step, titles and abstracts were screened, followed by screening of full texts of eligible studies. Screening was performed independently in reviewer teams of two. Disagreements or uncertainty about eligibility were resolved through consensus. The study selection process is displayed in figure 1. Reasons for study exclusion are outlined in supplementary material S3.

### Data extraction

For studies meeting the inclusion criteria, two authors independently extracted key study characteristics into predefined data extraction forms (see table 2). Potential disagreements were resolved by discussion.

We also contacted authors of several publications via email to request relevant data. One author (37) supplied additional, unpublished data.

### Coding of study characteristics

For each included study, the following characteristics were coded: (i) *study framework:* first author, year of publication, country and study design; (ii) *participants:* age, percent female, higher proportion (>50%) of female participants (yes, no), management level and occupational setting (eg, healthcare, production or public administration), type of control group (alternative intervention, passive or waitlist control group); (iii) *intervention*: format, length (<1, 1–4, 5–8, 9–12, and >12 weeks), training setting (face-to-face, virtual or hybrid), home practice (yes or no), delivery mode (self-practice, group or individual session, or mixed format), type (cognitive behavioral, relaxation, biofeedback, organization-focused, alternative to the others or multimodal in accordance to (24), number of intervention components (1, 2 or 3), follow-up periods (short-, medium- or long-term (<3, 3–12 or >12 months); (iv)*outcome domains*: the primary outcome data were structured by classifying the initially reported constructs into review outcomes across the following domains: (a) psychological stress (including perceived stress and job stress), (b) mindfulness, (c) mental health (including subsyndromal symptoms and general mental health), (d) work-related outcomes and (e) leadership-related outcomes. See table 1 and supplementary material S4 and S5 for detailed information on analyzed outcome domains and applied measurement instruments.

**Table 1:** Outcome domains and subcategories based on constructs from primary studies. [NA=not applicable]

Main level outcome categories	Subcategories^a^	Original reported constructs
Stress, Psychological	NA	General subjective stress ^b^
		Secondary Traumatic Stress
		Stress
		Stress in general ^b^
		Stress reactivity
	Job stress	Effort–reward ratio
		Irritation
		Work-related stress
		Work-stress
	Perceived stress	Perceived stress
Mindfulness	NA	Mindfulness
		Leaders’ mindfulness
		Trait mindfulness
Mental health	Subsyndromal symptoms	Anxiety
		Burnout
		Depression
		Depressive symptoms
		Negative effect
		State anxiety ^b^
		State or current anxiety
		Symptoms of depression and anxiety ^b^
		Trait anxiety ^b^
		Trait or dispositional anxiety
	General mental health	Emotional distress
		General mental health
		Symptoms of distress
		Psychological distress
Work-related outcomes	NA	Career satisfaction
		General work performance
		Global job satisfaction
		Job satisfaction
		Job performance ^b^
		Non-specific sickness absence days
		Lost productive time (absenteeism days) ^b^
		Sickness absence
		Presenteeism ^b^
Leadership-related outcomes	NA	Abusive leadership
		Authentic leadership
		Health promoting self-care
		Health promoting staff-care
		Leadership competencies
		Leadership effectiveness
		Leadership practice ^b^
		Transformational leadership

^a^ When N>3 studies could be pooled for meta-analysis. ^b^ These constructs could not be included in the meta-analysis due to insufficient data availability from the studies. See supplementary table S7 for detailed information.

When a study presented more than one measure (including more than one follow-up measurement) per outcome, we calculated the weighted mean and pooled standard deviation (SD), according to Cohen (38) and displayed in equations (1) and (2):


Mpooled= M1*n1+M2*n2n1+ n2 (1)

$$SD_{pooled}=\;\sqrt{\frac{(n_{1}-1)\;*{SD}_{1}^{2}\;+\;(n_{2}-1)\;*{SD}_{2}^{2}}{n_{1}+\;n_{2}-\;2}}$$ (2)

If both self- and follower-reported data were available, we prioritized the inclusion of self-reported data. In instances of two control groups, preference was given to the passive control group. Similarly, when two intervention groups were available, we prioritized the inclusion of the intervention group with a broader intervention format.

### Risk of bias assessment

Two reviewers independently assessed all studies, and disagreements were resolved by consensus. RCT were assessed using the Cochrane 'risk-of-bias' (RoB) assessment tool for randomized trials (ROBINS-II) (39). Based on the rating criteria, the five bias domains and overall RoB were evaluated as 'low RoB', 'some concerns RoB' or 'high RoB'. CBA were evaluated using the Cochrane RoB assessment tool for non-randomized studies of interventions (ROBINS-I) (40). Based on the overall risk of bias judgment, each domain of bias was rated as 'low RoB', 'moderate RoB' or 'serious RoB', 'critical RoB' or 'no information on RoB'. For the meta-analysis, we employed the RoB2 scheme, where a 'moderate RoB' in CBA is classified as 'some concern RoB', while 'serious' and 'critical RoB' were assigned as 'high RoB'. Detailed information on rating criteria for RoB assessment are presented in supplementary material S6.

### Synthesis methods

To conduct the meta-analyses, we used the statistical software R Studio (version 4.3.3) (41), utilizing the 'meta' package (42). We performed meta-analyses when data from ≥4 studies with different study populations could be pooled. If the included studies did not furnish adequate data for incorporation into the meta-analysis (eg, reporting no SD/effect) and, despite our requests, we did not receive the necessary information from the authors, the findings were documented in narrative tables (supplementary material S7).

We computed the standardized mean difference (SMD) scores between the intervention and control groups as the dependent variable (Hedges' g = [m_Intervention Group_ – m_Control Group_]/sd_pooled_). A g-score was constructed for all analyzed outcomes and each review outcome, with lower values indicated greater improvements in the intervention groups. The values assigned to Hedges' g were interpreted following Cohen's guidance (38), where |g|=0.20–0.49 indicates a small effect, |g|=0.50–0.79 indicates a medium-sized effect, and |g|≥0.80 indicates a large effect. To address the substantial heterogeneity observed among endpoints within each outcome, we employed the random effects model with a restricted maximum likelihood technique (43). Additionally, we utilized the Hartung-Knapp method to estimate the 95% confidence intervals (CI).

Sensitivity analyses were undertaken to validate the overall credibility of g-scores as substantial outliers were detected among effect sizes across all outcome categories except mindfulness (supplementary material S8, S9 and S10). For that, the 'find.outliers' command within the dmetar package (44) was used.

Heterogeneity was evaluated using the I^2^ statistic (45) and tested with Cochran's Q statistic (46), as recommended by the Cochrane Collaboration (34). The I^2^ values were interpreted as follows: ≤25% indicated low heterogeneity, >25–≤ 50% indicated moderate heterogeneity, >50–≤ 75% indicated substantial heterogeneity, and >75% indicated high heterogeneity (45).

We tried to explain heterogeneity by conducting exploratory moderator analyses, when ≥10 studies per outcome were examined (47). Categorical moderators [RoB, study design, control group, follow-up, management level, higher proportion (>50%) of female participants (>50%), intervention type, number of intervention components, intervention length, delivery mode and training setting] were explored via subgroup analyses (supplementary material S11) and continuous moderators (age, % female, sample size, publication year) via meta-regression (supplementary material S12).

Publication bias was assessed by conducting visual inspection of (contour-enhanced) funnel plots (48, 49) (supplementary material S13). When a meta-analysis included ≥10 studies, we applied the Egger's test (50) (supplementary material S14).

All statistical analyses were conducted in accordance with the methodological standards outlined in the Cochrane Handbook for Systematic Reviews of Interventions (34).

### Certainty of evidence assessment

We utilized the Grading of Recommendations Assessment, Development and Evaluation (GRADE) framework (51) to evaluate the overall certainty of evidence for each primary outcome. Two review authors independently assessed the certainty of evidence for each outcome, with any discrepancies resolved through discussion. The Summary of Findings (table 3) succinctly presents the certainty of evidence results. Detailed information on the criteria used for grading the evidence are outlined in supplementary material S15.

## Results

Electronic search retrieved 3904 non-duplicate records and one grey literature publication. A total of 53 full-text articles were retrieved of which 25 studies (37, 52–72) met full inclusion criteria. Details on the selection process and reasons for exclusion of the full-text screened studies are described in figure 1 and supplementary material S3.

### Study characteristics

A comprehensive overview of the included publications is presented in table 2.

**Table 2:** Characteristics of the studies included. [CBA=controlled before and after; IG=intevention group; CG=control group; ERI=effort–reward model; NR=not reported; PS=psychological stress; RCT=randomized control trial.]

First author (year); country; study design	Control group; Follow-up period	Management level; setting	Number of participants (IG; CG)	Intervention	Intervention format	Intervention type (number of intervention components); length	Training setting (home practice); delivery mode	Outcome categories	Subcategories
Allen (1980); Australia; CBA (52)	Waitlist; short-term	Middle; NR	10; 10	Biofeedback-based stress management training program	Once-weekly 1-hr sessions	Biofeedback (1); 5-8 wk	Face-to-face (yes); individual sessions	PS; mental health; work-related outcome	Subsyndromal symptoms
Bennett (2011); USA; RCT (53)	Passive; medium-term	Diverse; diverse settings	72; 73	Psycho- educational health and leadership development program	Interactive learning elements (self-assessment, sumulation tools. short videos, reading material), web-based coaching and seminars, links to other online health courses, minimum of 10 hrs spend on user platform	Multimodal (2); >12 wk	Virtual (no); self-practice	Mental health	General mental health
Blank (2018); Austria; RCT (54)	Alternative (free time at home); short-term	Middle; diverse settings	20; 20	Short vacation based on effort– recovery model	4 nights including one session of moderate physical activity and one session of active recovery	Alternative to the others (1); <1week	Face-to-face (no); mixed (individual sessions, self-practice)	PS	Perceived stress
Cedstrand (2022); Sweden; CBA (55)	passive; long-term	Lower; production	54; 20	Co-created occupational health intervention built on behavior change weel framework; structured roundmaking and duties clarification and staffing plan	4 modules: 1 full-day face-to-face workshops plus a 2-hr online follow-up, 1 online full day plus 2x 3-hr online follow-ups	Organization-focused (1); NR	Hybrid (no); group sessions	PS	/
Deval (2017); France; CBA (56)	Passive; short-term	NR; service	53; 27	Acceptance and commitment therapy intervention	3x4-hr sessions including homework assignments on mindfulness and values-based action	Cognitiv-behavioral (1); 5-8 wk	Face-to-face (yes); mixed (group sessions, self-practice)	PS; mindfulness; mental health; work-related outcomes	Perceived stress; general mental health
Gast (2022); Germany; RCT (57)	Waitlist; short-term	Diverse; production	36; 45	Intervention on own and employees work-related stress based on ERI model	1-day intervention incl. theoretical input, interactive group work, case discussion	Multimodal (2); <1 week	Virtual (no); group sessions	PS; mental health	Perceived stress, job stress; subsyndromal symptoms
Igu (2023); Nigeria; RCT (58)	Waitlist; mixed	NR; teaching	38; 39	Problem-solving client-based psychoeducational intervention for managing workplace threats to mental health	1 opening meeting, eight weekly coaching sessions of 120min	Cognitive-behavioral (1); 9-12 wk	Face-to-face (no); mixed (one group session, individual sessions)	Mental health	Subsyndromal symptoms
Janka (2017); Austria, Germany, Luxembourg; RCT (59)	Waitlist; Sshort-term	Diverse; public adminstration	18; 18	Biofeedback training	9x45 mins sessions, home practice	Biofeedback (1); 5-8 wk	Face-to-face (yes); individual sessions	PS	Perceived stress
Lange (2019); Germany; CBA (21)	Passive; medium-term	Diverse; mixed	19; 18	Mindful leadership intervention built on interest- and intrinstic motivation theories covering stress, stress management, mindfulness, leadership & communication	1-day training (7hr), two follow-up sessions (one-on-one coaching (30min); group session (90min) and digital based mindfulness/relaxation instructional videos (voluntary)	Multimodal (3); 9-12 wk	Hybrid (yes); mixed (group sessions, self-practice)	PS; mindfulness; leadership-related outcomes	Job stress
Li (2017); Germany; RCT (22)	Passive; long-term	Diverse; production	94; 94	Group-oriented stress prevention program built on ERI model	8x90 min sessions over 2 consecutive days, 2 refresher courses comprising 2x180 min sessions	Cognitive-behavioral (1); >12 wk	Face-to-face (no); goup sessions	PS; mental health	Job stress; subsyndromal symptoms
Limm (2011); Germany; RCT (60)	Waitlist; long-term	Diverse; production	75; 79	Group-oriented stress prevention program built on effort-reward imbalance model	8x90 min sessions over 2 consecutive days, 2 refresher courses comprising 2x 180 min sessions	Cognitive-behavioral (1); >12 wk	Face-to-face (no); group sessions	PS; mental health	Job stress; subsyndromal symptoms
First author (year); country; study design	Control group; Follow-up period	Management level; setting	Number of participants (IG; CG)	Intervention	Intervention format	Intervention type (N); length	Training setting (home practice); delivery mode	Outcome categories	Subcategories
Ly (2014); Sweden; RCT (62)	Waitlist; short-term	Middle; diverse	36; 37	Acceptance and commitment therapy based smartphone intervention	6 modules (short audio lecture, 2-3 texts, 2-4 exercises); minimum of daily 15 min spend on program	Cognitiv-behavioral (1); 5-8 wk	Virtual (no); self-practice	PS; mental health; leadership-related outcome	Perceived stress; general mental health
Martin (2020); Australia; RCT (63)	Waitlist; medium-term	Top; diverse	78; 104	Self- administered educational intervention on promoting mental health focused skills development	DVD program (60 mins), resource kit (30-page manual, fact sheets, booklets, posters), telephone support (6x30 mins)	Cognitiv-behavioral (1); NR	Virtual (no); self-practice	Mental; work-related outcomes	General mental health
Mellner (2022); Sweden; RCT (64)	Waitlist; mixed	Diverse; service	20; 20	Mindfulness-based stress reduction program	8-week structured group format including weekly 2.5 hr group sessions plus web-based audio recordings	Multimodal (2); 5-8 wk	Face-to-face (yes); group sessions	Mindfulness	/
Munafo (2016); Italy; RCT (37)	Alternative (daily stress diary); short-term	Diverse; finance; public adminstration	16; 15	Respiratory sinus arrhythmia biofeedback intervention	Weekly 45min sessions	Biofeedback (1); 5-8 wk	Face-to-face (no); individual sessions	Mental health	Subsyndromal symptoms
Ni (2022); China; CBA (61)	Waitlist; short-term	Lower; production	36; 36	Mindfulness self-training based on mindfulness cognitive therapy and mindfulness-based stress reduction	Daily mindfulness practices (3 min breathing space, 10 min mindfulness of breathing mediation, body scan mediation, and mindful walking)	Multimodal (2); 1-4 wk	Hybrid (no); mixed (group sessions, self-practice)	Mindfulness	/
Nübold (2020); Germany, Netherlands; RCT (65)	Waitlist; short-term	NR; diverse	93; 80	Self-guided, app-based mindfulness meditation training	Guided mindfulness meditation exercises developed by Headspace, Inc	Relaxation (1); 1-4 wk	Virtual (no); self-practice	Mindfulness; leadership-related outcome	/
Reitz (2020); England; CBA (66)	Waitlist; short-term	NR; diverse	27; 30	Mindful leader intervention based on mindfulness‐based stress reduction and mindfulness-based cognitive therapy	3 half-day workshops every two wk, 1 full day workshop, 1 small group conference call over 8 wk	Multimodal (2); 5-8 wk	Face-to-face (yes); mixed (group sessions, self-practice)	Mindfulness; leadership-related outcome	/
Sawyer (2023); USA; RCT (67)	Waitlist; medium-term	Lower; healthcare	39; 38	Psychoeducational Group Program on mental well-being based on an integrative theoretical framework of mindfulness, acceptance and commitment therapy, and cognitive-behavioral therapy	Nine weekly 90 min online group sessions promoting self-care, growth and adaptive coping	Multimodal (2); 9-12 wk	Virtual (no); group sessions	PS; mental health; work-related outcome	Perceived stress; subsyndromal symptoms
Shonin (2014); UK; RCT (68)	Alternative (education group program on cognitive behavioral theory); mixed	Middle; NR	76; 76	Meditation awareness training	Weekly 90 min workshops, daily self-practice, two optional one-to-one support sessions (50 min) plus daily self-practice	Relaxation (1); 5-8 wk	Face-to-face (yes); mixed (group sessions, self-practice)	PS; mental health; work-related outcomes	Job stress; general mental health
Vonderlin (2021); Germany; CBA (69)	Passive; medium-term	Diverse; diverse	117; 117	Health-promoting leadership intervention covering the topics (a) health-promoting selfcare, (b) health-promoting staff-care, and (c) addressing employees under stress built on the health-oriented leadership model	3 full-day courses (8 hr each), 2x3-hr booster, optional mindfulness practice; 30 hr over a period of 6 months	Multimodal (3); >12 wk	Face-to-face (yes); mixed (group sessions, self-practice)	Mental health; leadership-related outcome	Subsyndromal symptoms
First author (year); country; study design	Control group; Follow-up period	Management level; setting	Number of participants (IG; CG)	Intervention	Intervention format	Intervention type (N); length	Training setting (home practice); delivery mode	Outcome categories	Subcategories
Vonderlin (2023); Germany; CBA (20)	Passive; long-term	Diverse; healthcare and science	13; 269	Health-promoting leadership intervention covering the topics (a) health-promoting selfcare, (b) health-promoting staff-care, and (c) addressing employees under stress built on the health-oriented leadership model	3 full-day courses (8 hr each), 2x3-hr booster, optional mindfulness practice; 30 hr over 6 months	Multimodal (3); >12 wk	Face-to-face (yes); mixed (group sessions, self-practice)	Work-related outcome	/
Wasylkiw (2015); Canada; CBA (70)	Passive; short-term	Middle; healthcare	11; 10	Intensive weekend retreat on mindfulness	14 x16 hrs of mindfulness-based guided practice, follow-up 2-hr webinar intensive weekend retreat on mindfulness	Relaxation (1); 5-8 wk	Hybrid (yes); mixed (group sessions, self-practice)	PS; mindfulness; leadership-related outcomes	Perceived stress
Yong (2020); South Korea; CBA (71)	Passive; mixed	Middle; healthcare	27; 27	Holy Name Meditation Program based on Easwaran’s 8-point program	Five weekly sessions followed by 3 monthly 90 min sessions	Relaxation (1); >12 wk	Face-to-face (yes); mixed (group sessions, self-practice)	Mental health; work-related outcome; leadership-related outcome	Subsyndromal symptoms
Zolnierczyk-Zreda (2016); Poland; RCT (72)	Waitlist; medium-term	Middle; finance and service	72; 72	Mindfulness‐based stress reduction training	8 weekly 180 min group sessions, one 7-hr group session (the ‘Mindfulness Day’), individual follow-up session, daily homework exercises	Multimodal (2); 9-12 wk	Face-to-face (yes); mixed (group sessions, self-practice)	PS; mental health; work-related outcome	Job stress; subsyndromal symptoms

Among the 25 identified studies, 15 were RCT (22, 37, 53, 54, 57–60, 62–65, 67, 68, 72) and 10 CBA (20, 21, 52, 55, 56, 61, 66, 69–71). Publications spanned >40 years of research: 12 studies were published in 2020–2023 (20, 55, 57, 58, 61, 63–67, 69, 71), 10 in 2014 –2019 (21, 22, 37, 54, 56, 59, 62, 68, 70, 72), 2 in 2011 (53, 60) and 1 in 1980 (52).

The majority of studies (72%) were conducted in European countries (20–22, 37, 54, 55, 57, 59, 60, 62, 64–66, 68, 69, 72), followed by studies from the USA (53, 67) and Australia (52, 63). One study each was conducted, in Canada (70), China (61), Nigeria (58) and South Korea (71).

In total, 2466 supervisors participated in the included studies, with 1120 assigned to the intervention arm. Sample size ranged from 9–269 participants, with an average of 45 supervisors participating in the intervention groups and 41 supervisors in the control groups. The mean age of participants, which was reported in 17 studies, was 43.2 years. Based on 24 studies providing data on gender there was a nearly equal distribution between female (49,8%) and male (50,2%) participants. Supervisors represented diverse management levels and worked in various occupational settings, including finance, healthcare, science, service, production, public administration or school.

Overall, the identified occupational stress management interventions targeting supervisors could be categorized into different types. The majority (k=10) were multimodal interventions (20, 21, 53, 56, 57, 62, 64, 66, 67, 69, 72), combining cognitive-behavioral, relaxation, or leadership-specific components. Six studies (25%) applied a cognitive-behavioral approach (22, 56, 58, 60, 62, 63), four (16%) focused on relaxation techniques (65, 68, 70, 71), followed by biofeedback (37, 52, 59), organization-focused (55), and alternative intervention (54). Most multimodal interventions (66%) applied two different intervention type components.

Interventions varied widely, from one 8-hour virtual group sessions (57) to eight weekly 180-minute group sessions with individual follow-up sessions and daily homework exercises over three months (72) or interventions providing 30 hours of learning over six months (20, 69). Most commonly, interventions employed multiple delivery modes, including group or individual sessions and self-practice (20, 21, 54, 56, 58, 61, 66, 68–72). Specifically, all three biofeedback interventions (37, 52, 59) were administered through individual face-to-face sessions, while virtual training formats (k=6) (53, 57, 62, 63, 65, 67) included group sessions or self-practice. Home practice was encouraged in 48% of included studies.

The majority of studies (91%) compared intervention effects to waitlist (k=13) (52, 57–67, 72) or passive (k=9) (20–22, 53, 55, 56, 69–71) control groups. Among the included studies, eleven (44%) measured short-term (37, 52, 54, 56, 57, 59, 61, 62, 65, 66, 70), six (24%) medium-term (21, 53, 63, 67, 69, 72), and four (16%) long-term outcomes (20, 55, 60). Additionally, four publications (16%) combined data from short- and medium-term follow-ups (58, 64, 68, 71).

In total, five distinct outcome categories were examined: (i) psychological stress, (ii) mindfulness, (iii) mental health, (iv) work-related outcomes and (v.) leadership-related outcomes. When ≥4 studies were available measuring similar constructs, we further subdivided these outcome categories into subcategories. As a result, the following four subcategories emerged: (i.i.) perceived stress, (i.ii.) job stress, (iii.i.) subsyndromal symptoms, and (iii.ii.) general mental health. On average, studies reported two outcome categories or three (sub)categories. Additional information on grouped outcome categories, including the original reported constructs and measurement instruments used, is provided in table 1 and supplementary material S4 and S5.

### Results of the meta-analyses and sensitivity analyses

Overall, 21 studies could be included in the meta-analysis (see table 3 and supplementary material S8). On the outcome category level, the number of pooled studies for meta-analyses varied between k=12 for mental health and k=5 for work-related outcomes (see table 3 and supplementary material S9).

When pooling all intervention outcomes, the meta-analysis revealed no significant intervention effect for leader-targeted SMI in comparison to passive, active or waitlist control groups [g=-0.18 (95% CI -0.38– 0.03)] (table 3 and supplementary material S8). After removing outliers due to baseline imbalance and large effect sizes (58, 69, 72), a significant intervention effect on leader-targeted SMI emerged [g=-0.13 (95% CI -0.24– -0.01)]

**Table 3:** Summary of findings. [CI=confidence interval]

Outcome	Number of studies (reference)	g standardized mean difference scores, 95% CI (Hedges’ g)	Summary of findings	Certainty of evidence (GRADE)
**All analyzed interventions**				
All interventions	21 studies (20–22, 37, 53–56, 58, 60–70, 72)	All studies: -0.18 (95% CI -0.38–0.03); Without outliers: -0.13 (95% CI -0.24– -0.01)*	After excluding outliers, significant improvements in various health- and performance-related outcomes were observed among supervisors participating in leader-targeted SMIs, when contrasted with supervisors in control groups or alternative interventions. Due to high heterogeneity, a minor effect size, and significant limitations concerning study quality, caution is warranted when interpreting the results.	/
**Main level outcome categories**				
Psychological Stress	11 studies (21, 22, 54–56, 60, 62, 67, 68, 70, 7, 2)	All studies: -0.02 (95% CI-0.43–0.39); Without outliers (k=9): -0.12 (95% CI -0.28–0.04)	No significant effect on psychological stress was observed among supervisors participating in occupational stress management interventions when contrasted with supervisors undergoing alternative interventions or the control group. Due to high heterogeneity and a contrary effect between high RoB and some concerns RoB studieIs, we downgraded the results to reflect ‘low certainty of evidence’.	⊕⊕⊝⊝ Low^ade^
Mindfulness	7 studies (21, 56, 61, 64–66, 70)	All studies: -0.03 (95% CI -0.32–0.27); No outliers	No significant effect was observed on mindfulness among supervisors engaged in occupational stress management interventions, in comparison to supervisors undergoing alternative interventions or serving as the control group. Given the small sample size and high RoB in five studies, we downgraded the certainty of evidence to ‘low’.	⊕⊕⊝⊝ Low^bdf^
Mental health	12 studies (22, 37, 53, 56, 58, 60, 62, 63, 67–69, 72)	All studies: -0.38 (95% CI -0.69– -0.08)*; Without outliers (k=10): -0.21 (95% CI -0.41– -0.02)*	A significant small-to-moderate effect on the improvement of mental health was observed among supervisors participating in occupational stress management interventions compared to a control group or alternative intervention. After excluding outliers, the significant effect persisted. Considering that 50% of the included studies had a high RoB, along with moderate 95% confidence intervals across all studies and substantial heterogeneity, we downgraded the certainty of evidence to ‘low’.	⊕⊕⊝⊝ Low^ade^
Work-related outcomes	5 studies (20, 56, 67, 68, 72)	All studies: -0.68 (95% CI -1.84–0.48); Without outliers (k=4): -0.32 (95% CI -0.63– -0.00)*	Without outliers, a small significant effect on the improvement of work-related outcomes was observed among supervisors participating in occupational stress management interventions, in comparison to supervisors in the control group or those undergoing alternative interventions. Since 50% of the included studies had a high RoB, moderate 95% confidence intervals across all studies, high heterogeneity, as well as indications of publication bias, we downgraded the certainty of evidence to ‘very low’.	⊕⊝⊝⊝ Very low^bdfi^
Leadership-related outcomes	6 studies (21, 62, 65, 66, 69, 70)	All studies: -1.16 (95% CI -3.42–1.10); Without outliers (k=5): -0.23 (95% CI -0.44– -0.02)*	Without outliers, a small significant effect on the improvement of leadership-related outcomes was observed among supervisors compared to a control group or alternative intervention. Due to 50% of the included studies exhibiting a high RoB, a moderate 95% confidence interval, substantial heterogeneity, and indications of publication bias, we downgraded the certainty of evidence to ‘very low’.	⊕⊝⊝⊝ Very low^bdfi^
**Subcategory level outcomes**				
Psychological Stress: Perceived stress	5 studies (54, 56, 62, 67, 70)	All studies: -0.15 (95% CI -0.38–0.08); No outliers	Findings suggest that no significant effect on perceived stress was found among supervisors participating in occupational stress management interventions, in comparison to those undergoing alternative interventions or serving as the control group. Considering the small sample size, three out of five studies exhibiting a high RoB, and assumed publication bias, we downgraded the certainty of evidence to ‘low’.	⊕⊕⊝⊝ Low^beh^
Psychological Stress: Job stress	5 studies (21, 22, 60, 68, 72)	All studies: 0.02 (95% CI -1.09–1.14); No outliers	No significant effect on job stress was observed among supervisors participating in occupational stress management interventions when contrasted with supervisors undergoing alternative interventions or comprising the control group. Due to the contradictory effect size between studies with some concerns of RoB versus those with high RoB, along with a massive 95% confidence interval in high RoB studies and high heterogeneity, the certainty of evidence was downgraded to ‘very low’.	⊕⊝⊝⊝ Very low^cde^
Mental health: Subsyndromal symptoms	8 studies (22, 37, 53, 58, 60, 67, 69, 72)	All studies: -0.36 (95% CI -0.80–0.07); Without outliers (k=7): -0.22 (95% CI 0.53–0.09)	No significant effect on general mental health was observed among supervisors participating in occupational stress management interventions when contrasted with supervisors undergoing alternative interventions or comprising the control group. Due to substantial heterogeneity and moderate 95% confidence intervals in both high RoB and in studies with some RoB concerns, we downgraded the certainty of evidence to ‘low’.	⊕⊕⊝⊝ Low^ade^
Mental health: General mental health	5 studies (53, 56, 62, 63, 68)	All studies: -0.32 (95% CI -0.88–0.24); No outliers	No significant effect on general mental health was observed among supervisors participating in occupational stress management interventions when contrasted with supervisors undergoing alternative interventions or comprising the control group. Due to substantial heterogeneity and moderate 95% confidence intervals in both studies with high RoB and some RoB concerns, we downgraded the certainty of evidence to ‘low certainty’.	⊕⊕⊝⊝ Low^ade^

* P≤0.05. ^a^ Downgraded by -0.5 points due to risk of bias. ^b^ Downgraded by -1 points due to risk of bias. ^c^ Downgraded by -1.5 points due to risk of bias. ^d^ Downgraded by -1 points due to inconsistency. ^e^ Downgraded by -0.5 due to imprecision. ^f^ Downgraded by -1 due to imprecision. ^h^ Downgraded by -0.5 due to potential publication bias. ^i^ Downgraded by -1 due to publication bias.

On the outcome category level, a significant small-to-moderate intervention effect for occupational stress management programs targeting supervisors was observed for mental health [g=-0.38, (95% CI -0.69 – -0.08)] (figure 2a), even after excluding influential cases [g=-0.21, (95% CI -0.41– -0.02)]. Significant small-to-moderate intervention effects on work-related outcomes (g=-0.32, (95% CI -0.63– -0.00)] and leadership-related outcomes [g=-0.23 (95% CI -0.44– -0.02)] were seen when outliers (69, 72) were excluded (figure 2b and 2c). For psychological stress and mindfulness, no significant intervention effects were detected. The certainty of evidence was rated low for the outcomes of psychological stress, mindfulness, and mental health and very low for the outcomes of work- and leadership-related variables (see table 3 and supplementary material S9 for a detailed display of the calculated effect sizes and certainty of evidence ratings).

On the subcategory level, effect sizes were calculated based on k=5 for perceived stress, job stress, and general mental health and k=8 for subsyndromal symptoms. Meta-analyses revealed no significant effects in any of the addressed subcategories. Sensitivity analysis on the subcategory level only identified outliers (58) in subsyndromal symptoms [g=-0.22, (95% CI 0.53– 0.09)], without discerning significant intervention effects (see supplementary material S10). The certainty of evidence was rated very low for job stress and low for perceived stress, subsyndromal symptoms, as well as general mental health (see table 3 and supplementary material S15).

**Figure 2a:**
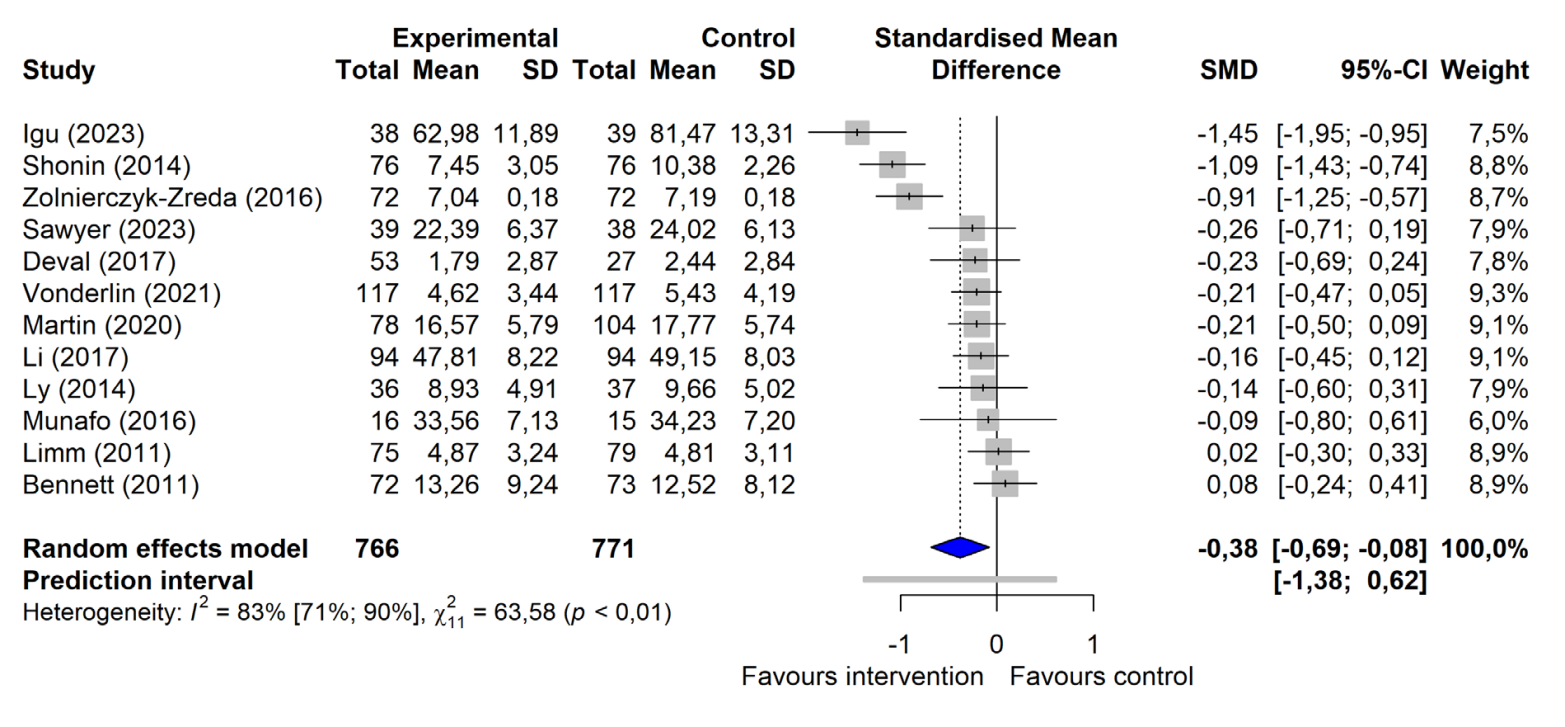
Forest plot of changes in mental health

**Figure 2b:**
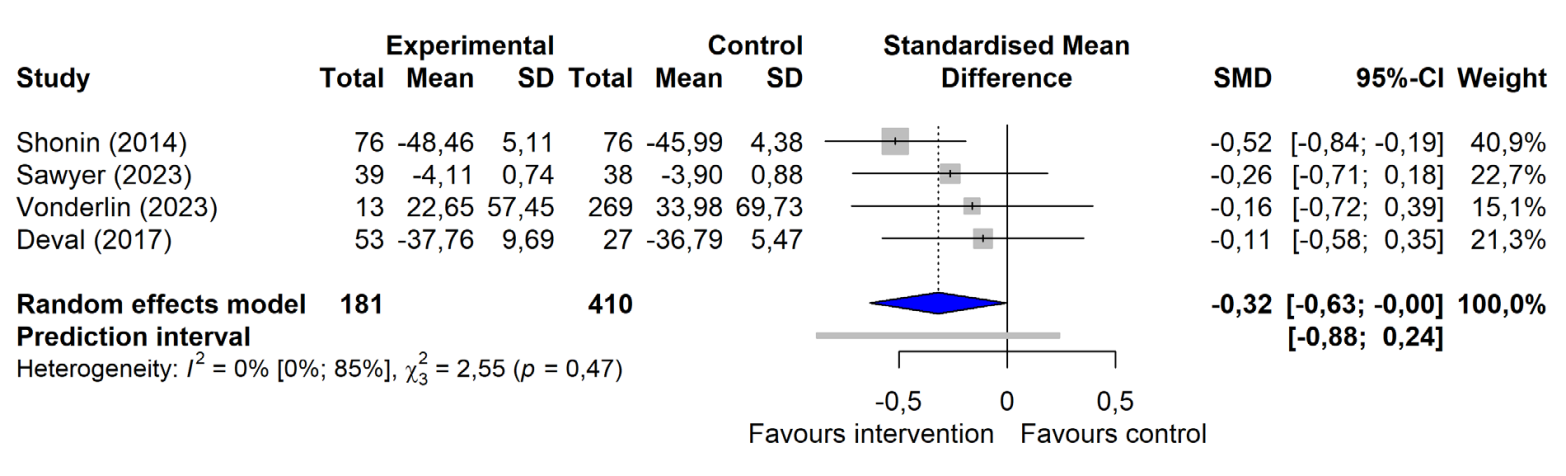
Forest plot of changes in work-related outcomes after exclusion of outliers.

**Figure 2c:**
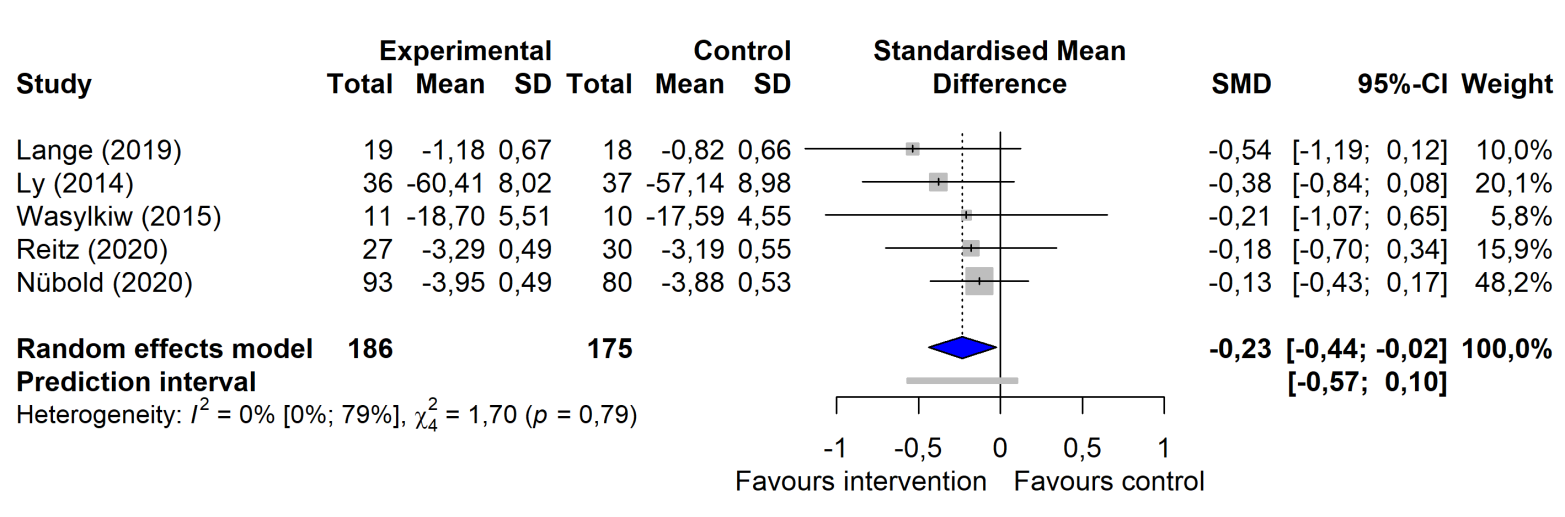
orest plot of changes in leadership-related outcomes after exclusion of outliers

### Heterogeneity and exploratory moderator analyses

The meta-analyses revealed substantially high heterogeneity with I^2^ >72% and wide prediction intervals for all interventions, all main and subcategory level outcome categories except mindfulness and perceived stress (see supplementary material S8, S9 and S10). In order to explain heterogeneity in effect sizes, exploratory moderator analyses were performed (see supplementary material S11 and S12). When considering all analyzed interventions, moderator analyses indicated that leader-targeted SMI rated as having 'some concerns' due to the fact that their RoB produced significantly higher effect sizes [g=-0.27 (95% CI -0.54– -0.01)] than studies with a high RoB [g=-0.12 (95% CI -0.45 –0.22)]. Additionally, leader-targeted SMI with a higher proportion (>50%) of female participants [g=-0.27 (95% CI, -0.50– -0.03)] and interventions lasting 5–8 weeks [g=-0.25 (95% CI -0.48– -0.03)] yielded significantly larger effect sizes compared to interventions with more men [g=-0.17 (95% CI -0.56–0.22)] and other time frames. For psychological stress, the moderator analysis found that cognitive-behavioral interventions yielded significantly higher effect sizes [g=-0.17, (95% CI -0.36– -0,00)] compared to other intervention types. Regarding mental health, moderator analyses indicated that the intervention effect of leader-targeted SMI was significantly higher in RCT [g=-0.42, (95% CI, -0.79– -0.04)] compared to CBA [g=-0.21, (95% CI -0.44–0.01)]. Furthermore, it was demonstrated that interventions employing face-to-face training [g=-0.51, (95% CI, -0.97– -0.06)] and utilizing a mixed delivery mode approach [g=-0.76, (95% CI -1.43– -0.09)] yielded significantly larger effect sizes compared to interventions utilizing virtual training, as well as those employing a single delivery approach. Detailed information on moderator analyses is displayed in supplementary material S11 and S12.

### Risk of bias

Of the 15 RCT assessed, 6 publications exhibited some concerns regarding RoB (37, 53, 54, 60, 62, 67), while 9 were classified as having high RoB (22, 57–59, 63–65, 67, 72). Of the 10 CBA, 3 publications had some concerns regarding RoB (20, 21, 69), 6 were classified as having a high RoB (52, 55, 61, 66, 70, 71) and one publication received a rating of critical RoB (56). When grouping RoB assessments, the majority of studies (64%) exhibited a high RoB. Further elaboration on the RoB criteria and ratings can be found in supplementary material S6, S16 and S17.

### Publication bias

To evaluate publication bias, we generated contour-enhanced funnel plots for all interventions, outcome categories and subcategories (see supplementary material S13). Visual examination indicates potential reporting bias for perceived stress, work-related outcomes, and leadership-related outcomes. Due to the limited number of studies pooled in these outcome categories (<10), statistical testing for funnel plot asymmetry was not feasible.

## Discussion

The objective of our systematic review and meta-analysis was to identify and summarize the evidence regarding the effectiveness of leader-targeted SMI on supervisors' psychological stress, mindfulness, mental health and work- and leadership-related outcomes.

Overall, our results suggest that leader-targeted SMI have the potential to contribute to occupational health, particularly in improving supervisors' mental health as well as work- and leadership-related outcomes. A statistically significant intervention effect for leader-targeted SMI was observed after excluding influential cases. At the main outcome category level, a small-to-moderate effect size was found on mental health, and after excluding outliers, on work- and leadership-related outcomes. Given that research on leader-targeted SMI remains in its early stages, however, some caution is warranted when interpreting the results. In particular, we observed high heterogeneity in the examined target groups, intervention formats and durations, as well as analyzed outcomes. We also found limitations in study quality, which resulted in low and very low certainty of evidence ratings. Nevertheless, our results underscore the potential benefits of SMI specifically targeted at supervisors and emphasize the need for professionals in workplace health promotion to establish and implement these interventions. From a public health perspective, this appears timely given the recent increase in mental health illnesses (73–75). Consequently, investing in these preventive activities not only has the potential to enhance supervisors' health and well-being but may also lead to economic benefits through improved productivity levels, reduced absenteeism, and lower healthcare costs (76).

It is surprising that our findings did not show significant intervention effects of leader-targeted SMI on psychological stress, despite these programs being specifically designed to mitigate stress. Similarly, no statistically significant intervention effect was observed on mindfulness even though it was a major component in many of the analyzed studies. These findings contradict previous meta-analytical results of SMI targeting employees (24) and workplace mindfulness trainings, which have traditionally been part of SMI (30) and shown significant intervention effects for employees (77–79) and promising effects for supervisors (27, 29). Two observations suggest that these rather surprising findings may stem from the quality of study design. First, sensitivity analysis showed that after the exclusion of outliers effect sizes on psychological stress changed from -0.02 to -0.12. Second, subgroup analysis on mental health revealed significantly higher efficacy in RCT compared to CBA. This indicates the need for future research to prioritize high-quality studies with robust methodological designs and validated measurement instruments to provide more reliable evidence on the effectiveness of leader-targeted SMI on psychological stress and mindfulness.

The transactional model (26) provides a useful framework to explain the variability in intervention effects. Using the model, it can be reasoned that the lack of significant intervention effects may indicate that the leader-targeted SMI did not adequately address participants' appraisals or coping strategies related to their experienced workplace stressors. Therefore, focusing on individual differences – such as baseline stress levels, target-specific stressors and work environments or developing and implementing tailored or case-specific interventions – could help to further understand intervention effects for leader-targeted SMI.

Although several reviews (5, 31, 80) emphasize the necessity of leadership interventions analyzing work-related outcomes like absenteeism, job performance or efficacy, our review only identified five studies (20, 56, 67, 68, 72) measuring work-related outcomes like job satisfaction, general work performance or sickness absence. Despite the understanding that conducting intervention studies in dynamic and complex settings like workplaces is rather challenging (81), it may be more reasonable for future research to first identify potential barriers of interventions addressing work-related outcomes. This could be more beneficial than simply advocating for more interventions that include work-related measurements.

Substantial RoB and high heterogeneity were major concerns in the analyzed interventions. To address these limitations, we downgraded the certainty of evidence in GRADE to low and very low and conducted exploratory moderator analysis to identify potential influencing variables. Among all analyzed interventions, significant moderator effects were identified for those with a higher proportion (>50%) of female participants as also recognized by Michaelsen et al (79) who examined the effectiveness of mindfulness-based or -informed interventions targeting employees. This suggests stronger intervention effects in studies with a higher proportion of female participants. Therefore, future research could benefit from exploring gender-specific leadership interventions to provide effective interventions for both genders. When designing these interventions, it should be noted that interventions lasting 5–8 weeks yielded significantly higher effect sizes compared to other timeframes in our analysis. This finding is also consistent with Michaelsen's result (79), which found that effect sizes on mental health outcomes decreased as the intervention duration increased. However, while Michaelsen identified that one-to-one sessions are most effective for mental health outcomes, our subgroup analysis on mental health yielded significantly larger effect sizes for face-to-face training and mixed delivery mode approaches compared to virtual training or single delivery approaches. This suggests that, especially in times of digitalization and remote work, social interactions seem particularly important for supervisors. As a result, future research could benefit from not only offering online self-paced intervention programs but also recognizing the crucial role that social interactions may play in enhancing mental health outcomes for supervisors through leader-targeted SMI. Accordingly, when designing these interventions, it may be beneficial to incorporate face-to-face components and mixed delivery modes. Additionally, cognitive-behavioral components, which intend to change individuals' appraisal of stressful situations and their subsequent reactions to them (82), may be considered. Our moderator analysis revealed that cognitive-behavioral approaches yield significantly higher effect sizes on psychological stress than relaxation, organizational, biofeedback, or alternative SMI components. This finding aligns with previous research that recognized cognitive-behavioral interventions as particularly effective in enhancing psychological outcomes (24, 83).

The most common intervention type were multimodal interventions. These interventions not only focused on addressing supervisors' behavior but also considered their role in promoting employees' health, acknowledging that supervisors are often regarded as being responsible for fostering healthy workplaces. Consequently, future research could examine whether participation in leader-targeted SMI leads to positive effects on knowledge and awareness regarding workplace health promotion as well as an increased willingness to implement workplace health promotion programs. First respective results (84, 85) point to positive associations between leadership interventions and improved behavior and attitudes towards workplace health activities.

### Strengths and limitations

Our systematic review and meta-analysis adheres to the methodological recommendations of the Cochrane Handbook for Systematic Reviews (34) and is characterized by a number of strengths. A broad number of studies were included, and unpublished data were requested from authors. Moderator analyses were conducted, allowing more nuanced recommendations for the effectiveness of leader-targeted SMI. The use of the broad definition of leader-targeted SMI reflects the diversity and evolving nature of this research field. However, the overall certainty of evidence of the included studies is rated as low and very low, indicating that evidence on the effectiveness of these interventions is still in its early stages. The majority of studies (64%) exhibited a high RoB and substantial heterogeneity was observed across interventions, characterized by variations in target groups, intervention length and format, and analyzed outcomes. These factors complicate the accurate estimation of true effect sizes and underscore the need to refine the definition of leader-targeted SMI. Additionally, it may be that the natural settings in which the interventions were conducted made it challenging to achieve randomization, which would be a gold standard of high-quality intervention research. Since there was no a priori selection of interventions identified in the literature research, interventions with very short duration and/or small sample sizes were included in the analyses. Although it might be argued that such interventions are unlikely to be effective. For work- and leadership-related outcomes, no more than five studies could be pooled, and substantial limitations in the quality of the included studies were observed. Exploratory moderator analyses were only feasible for the outcome domains of psychological stress and mental health due to the limited number of studies for all other outcomes except for all analyzed outcomes combined.

### Concluding remarks

In summary, our meta-analysis suggests that leader-targeted SMI can be an effective approach for promoting occupational health of supervisors in workplace settings, especially regarding mental health, work-related outcomes and leadership-related outcomes. It complements influential meta-analyses on occupational SMI [eg (24),] by offering novel insights and a specific focus on supervisors. Given that research on leader-targeted SMI is still in its early stages, our review provides an initial overview of the available evidence along with its limitations and areas for further investigation. It highlights the need for investment in intervention studies with robust designs, validated measurement instruments, and the development and evaluation of targeted-oriented SMI. Such interventions might prioritize face-to-face delivery, mixed delivery methods, durations of 5–8 weeks, cognitive-behavioral components, and gender-specific adaptations. Additionally, future research may refine the definition of leader-targeted SMI and investigate barriers to measuring work-related outcomes. It may also explore the specific effects of leader-targeted SMI for supervisors on workplace health promotion. It is important to emphasize however, that leader-targeted SMI can never be considered as a stand-alone approach for promoting the health and wellbeing of supervisors and employees. Individual factors (eg, health history, general stress level, personal resources, family situation) and organizational aspects (eg, company size, organizational culture and values, financial resources), along with the transformative impact of artificial intelligence on the workplace, also influence the effectiveness of these interventions and the health and wellbeing in workplace settings (86, 87). As a consequence, leader-targeted SMI should always be regarded as one component of a holistic approach to workplace health promotion.

## Supplementary material

Supplementary material

## References

[1] Thomas R. Mental health at work has remained in the shadows for too long: Belgium's Deputy Prime Minister urges EU to dedicate the next 10 years to mental health. 23rd ed.; 2024 [cited 2024 Jun 24]. Available from: https://eurohealthnet-magazine.eu/mental-health-at-work-has-remained-in-the-shadows-for-too-long-belgiums-deputy-prime-minister-urges-eu-to-dedicate-the-next-10-years-to-mental-health/

[2] Trautmann S, Rehm J, Wittchen HU. The economic costs of mental disorders: do our societies react appropriately to the burden of mental disorders? EMBO Rep 2016 Sep;17(9):1245–9. 10.15252/embr.201642951 27491723 PMC5007565

[3] Melzner L, Kröger C. Arbeitsunfähigkeit bei psychischen Störungen – ökonomische, individuelle und behandlungsspezifische Aspekte [Work disability due to mental disorders – economic, individual and treatment-specific aspects]. Bundesgesundheitsblatt Gesundheitsforschung Gesundheitsschutz 2024 Jul;67(7):751–9. 10.1007/s00103-024-03894-6 38789543 PMC11230963

[4] Hübers M, Walter UN, Krapf F, Schaller J, Mraß U, Mess F et al. #whatsnext2020 – Erfolgsfaktoren für gesundes Arbeiten in der digitalen Arbeitswelt [#whatsnext2020 - Success factors for healthy working in the digital world of work]; 2020 [cited 2024 Jul 15]. Available from: https://www.tk.de/resource/blob/2090400/70d8fb1c6221fd7bc4253a946e1fa308/whatsnext-2020-data.pdf

[5] Rudolph CW, Murphy LD, Zacher H. A systematic review and critique of research on “healthy leadership”. Leadersh Q 2020;31(1):101335. 10.1016/j.leaqua.2019.101335

[6] Justesen JB, Eskerod P, Christensen JR, Sjøgaard G. Implementing workplace health promotion – role of middle managers. IJWHM 2017;10(2):164–78. 10.1108/IJWHM-04-2016-0030

[7] Dannheim I, Buyken AE, Kroke A. Work-related stressors and coping behaviors among leaders in small and medium-sized IT and technological services enterprises. BMC Public Health 2023 Apr;23(1):700. 10.1186/s12889-023-15581-3 37059975 PMC10103039

[8] Kaluza AJ, Boer D, Buengeler C, van Dick R. Leadership behaviour and leader self-reported well-being: A review, integration and meta-analytic examination. Work Stress 2020;34(1):34–56. 10.1080/02678373.2019.1617369

[9] Harms PD, Credé M, Tynan M, Leon M, Jeung W. Leadership and stress: A meta-analytic review. Leadersh Q 2017;28(1):178–94. 10.1016/j.leaqua.2016.10.006

[10] Franke F, Felfe J, Pundt A. The Impact of Health-Oriented Leadership on Follower Health: Development and Test of a New Instrument Measuring Health-Promoting Leadership. German Journal of Human Resource Management. Zeitschrift für Personalforschung 2014;28(1-2):139–61.

[11] Barling J, Cloutier A. Leaders’ mental health at work: Empirical, methodological, and policy directions. J Occup Health Psychol 2017 Jul;22(3):394–406. 10.1037/ocp0000055 27732006

[12] Zimber A, Hentrich S, Bockhoff K, Wissing C, Petermann F. Wie stark sind Führungskräfte psychisch gefährdet? [To what extent are managers mentally at risk?]. Z Gesundhpsychol 2015;23(3):123–40. 10.1026/0943-8149/a000143

[13] Hentrich S, Zimber A, Sosnowsky-Waschek N, Gregersen S, Petermann F. The Role of Core Self-Evaluations in Explaining Depression and Work Engagement among Managers. Curr Psychol 2017;36(3):516–29. 10.1007/s12144-016-9439-x

[14] Wallis A, Robertson J, Bloore RA, Jose PE. Differences and similarities between leaders and nonleaders on psychological distress, well-being, and challenges at work. Consult Psychol J 2021;73(4):325–48. 10.1037/cpb0000214

[15] Federal Institute of Occupational Safety and Health. Stressreport Deutschland 2019 [Stress Report Germany 2019]; 2020.

[16] GKV-Spitzenverband. Leitfaden Prävention – Handlungsfelder und Kriterien nach § 20 Abs. 2 SGB V: Ausgabe 2021 [Prevention Guidelines – Areas of Action and Criteria according to Section 20, Paragraph 2 of the German Social Code (SGB V): 2021 Edition]. Berlin; 2021.

[17] Erschens R, Adam SH, Schröpel C, Diebig M, Rieger MA, Gündel H et al. Improving Well-Being and Fostering Health-Oriented Leadership among Leaders in Small and Medium-Sized Enterprises (SMEs): A Systematic Review. Healthcare (Basel) 2024 Feb;12(4):486. 10.3390/healthcare12040486 38391861 PMC10888323

[18] Kelloway EK. Mental health in the workplace: towards evidence-based practice. Can Psychol 2017;58(1):1–6. 10.1037/cap0000084

[19] Kelloway EK, Barling J. Leadership development as an intervention in occupational health psychology. Work Stress 2010;24(3):260–79. 10.1080/02678373.2010.518441

[20] Vonderlin R, Schmidt B, Biermann M, Lyssenko L, Heinzel-Gutenbrunner M, Kleindienst N et al. Improving Health and Reducing Absence Days at Work: Effects of a Mindfulness- and Skill-Based Leadership Intervention on Supervisor and Employee Sick Days. Mindfulness (N Y) 2023;14(7):1751–66. 10.1007/s12671-023-02172-x

[21] Lange S, Rowold J. Mindful leadership: evaluation of a mindfulness-based leader intervention. Gr Interakt Org 2019;50(3):319–35. 10.1007/s11612-019-00482-0

[22] Li J, Riedel N, Barrech A, Herr RM, Aust B, Mörtl K et al. Long-Term Effectiveness of a Stress Management Intervention at Work: A 9-Year Follow-Up Study Based on a Randomized Wait-List Controlled Trial in Male Managers. BioMed Res Int 2017;2017:2853813. 10.1155/2017/2853813 29181392 PMC5664277

[23] Ivancevich JM, Matteson MT, Freedman SM, Phillips JS. Worksite stress management interventions. Am Psychol 1990 Feb;45(2):252–61. 10.1037/0003-066X.45.2.252 2178505

[24] Richardson KM, Rothstein HR. Effects of occupational stress management intervention programs: a meta-analysis. J Occup Health Psychol 2008 Jan;13(1):69–93. 10.1037/1076-8998.13.1.69 18211170

[25] van der Klink JJ, Blonk RW, Schene AH, van Dijk FJ. The benefits of interventions for work-related stress. Am J Public Health 2001 Feb;91(2):270–6. 10.2105/AJPH.91.2.270 11211637 PMC1446543

[26] Lazarus RS, Folkman S. Stress, Appraisal, and Coping: Springer Publishing Company; 1984.

[27] Donaldson-Feilder E, Lewis R, Yarker J. What outcomes have mindfulness and meditation interventions for managers and leaders achieved? A systematic review. Eur J Work Organ Psychol 2019;28(1):11–29. 10.1080/1359432X.2018.1542379

[28] Urrila LI. From personal wellbeing to relationships: A systematic review on the impact of mindfulness interventions and practices on leaders. Hum Resour Manage Rev 2022;32(3):100837. 10.1016/j.hrmr.2021.100837

[29] Zhou Y, Wang C, Sin HP. Being “there and aware”: a meta-analysis of the literature on leader mindfulness. Eur J Work Organ Psychol 2023;32(3):299–316. 10.1080/1359432X.2022.2150170

[30] Tetrick LE, Winslow CJ. Workplace Stress Management Interventions and Health Promotion. Annu Rev Organ Psychol Organ Behav 2015;2(1):583–603. 10.1146/annurev-orgpsych-032414-111341

[31] Stuber F, Seifried-Dübon T, Rieger MA, Gündel H, Ruhle S, Zipfel S et al. The effectiveness of health-oriented leadership interventions for the improvement of mental health of employees in the health care sector: a systematic review. Int Arch Occup Environ Health 2020;188(653):481. 33011902 10.1007/s00420-020-01583-w PMC7532985

[32] Dannheim I, Ludwig-Walz H, Buyken AE, Grimm V, Kroke A. Effectiveness of health-oriented leadership interventions for improving health and wellbeing of employees: a systematic review. J Public Health (Berl) 2021;28(2):197.

[33] Page MJ, McKenzie JE, Bossuyt PM, Boutron I, Hoffmann TC, Mulrow CD et al. The PRISMA 2020 statement: an updated guideline for reporting systematic reviews. BMJ 2021 Mar;372(71):n71. 10.1136/bmj.n71 33782057 PMC8005924

[34] Higgins JP, Thomas J, Chandler J, Cumpston M, Li T, Page MJ et al., editors. Cochrane handbook for systematic reviews of interventions: Version 6.4 (updated August 2023); 2023. 10.1002/14651858.ED000142 PMC10284251 31643080

[35] McGowan J, Sampson M, Salzwedel DM, Cogo E, Foerster V, Lefebvre C. PRESS Peer Review of Electronic Search Strategies: 2015 Guideline Statement. J Clin Epidemiol 2016 Jul;75:40–6. 10.1016/j.jclinepi.2016.01.021 27005575

[36] Thomas J, Graziosi S, Brunton J, Ghouze Z, O’Driscoll P, Bond M. EPPI-Reviewer: Advanced software for systematic reviews, maps and evidence synthesis. London: UCL Social Research Institute; 2020.

[37] Munafò M, Patron E, Palomba D. Improving Managers’ Psychophysical Well-Being: Effectiveness of Respiratory Sinus Arrhythmia Biofeedback. Appl Psychophysiol Biofeedback 2016 Jun;41(2):129–39. 10.1007/s10484-015-9320-y 26446978

[38] Cohen J. Statistical power analysis for the behavioral sciences. 2nd ed. Hillsdale, NJ: Erlbaum; 1988. 567 p.

[39] Sterne JA, Savović J, Page MJ, Elbers RG, Blencowe NS, Boutron I et al. RoB 2: a revised tool for assessing risk of bias in randomised trials. BMJ 2019 Aug;366:l4898. 10.1136/bmj.l4898 31462531

[40] Sterne JA, Hernán MA, Reeves BC, Savović J, Berkman ND, Viswanathan M et al. ROBINS-I: a tool for assessing risk of bias in non-randomised studies of interventions. BMJ 2016 Oct;355:i4919. 10.1136/bmj.i4919 27733354 PMC5062054

[41] R Studio Team. RStudio: Integrated Development for R. Boston; 2022.

[42] Balduzzi S, Rücker G, Schwarzer G. How to perform a meta-analysis with R: a practical tutorial. Evid Based Ment Health 2019 Nov;22(4):153–60. 10.1136/ebmental-2019-300117 31563865 PMC10231495

[43] Langan D, Higgins JP, Jackson D, Bowden J, Veroniki AA, Kontopantelis E et al. A comparison of heterogeneity variance estimators in simulated random-effects meta-analyses. Res Synth Methods 2019 Mar;10(1):83–98. 10.1002/jrsm.1316 30067315

[44] Viechtbauer W, Cheung MW. Outlier and influence diagnostics for meta-analysis. Res Synth Methods 2010 Apr;1(2):112–25. 10.1002/jrsm.11 26061377

[45] Higgins JP, Thompson SG, Deeks JJ, Altman DG. Measuring inconsistency in meta-analyses. BMJ 2003 Sep;327(7414):557–60. 10.1136/bmj.327.7414.557 12958120 PMC192859

[46] Cochran WG. The Combination of Estimates from Different Experiments. Biometrics 1954;10(1):101. 10.2307/3001666

[47] Jonathan J. Deeks, Julian PT Higgins, Douglas G Altman, on behalf of the Cochrane Statistical Methods Group. Chapter 10: Analysing data and undertaking meta-analyses [Internet]. Available from: https://training.cochrane.org/handbook/current/chapter-10

[48] Sterne JA, Sutton AJ, Ioannidis JP, Terrin N, Jones DR, Lau J et al. Recommendations for examining and interpreting funnel plot asymmetry in meta-analyses of randomised controlled trials. BMJ 2011 Jul;343:d4002. 10.1136/bmj.d4002 21784880

[49] Matthew J. Page, Julian PT Higgins, Jonathan AC Sterne. Chapter 13: Assessing risk of bias due to missing results in a synthesis. In: Higgins JPT, Thomas J, Chandler J, Cumpston M, Li T, Page MJ, et al., editors. Cochrane handbook for systematic reviews of interventions: Version 6.4 (updated August 2023); 2023.

[50] Egger M, Davey Smith G, Schneider M, Minder C. Bias in meta-analysis detected by a simple, graphical test. BMJ 1997 Sep;315(7109):629–34. 10.1136/bmj.315.7109.629 9310563 PMC2127453

[51] Schünemann HJ, Brożek J, Guyatt G, Oxman A. GRADE Handbook: Handbook for grading the quality of evidence and the strength of recommendations using the GRADE approach. Updated October 2013. [Internet] [cited 2024 Mar 6]. Available from: https://gdt.gradepro.org/app/handbook/handbook.html

[52] Allen JK, Blanchard EB. Biofeedback-based stress management training with a population of business managers. Biofeedback Self Regul 1980 Dec;5(4):427–38. 10.1007/BF01001358 7213824

[53] Bennett JB, Broome KM, Schwab-Pilley A, Gilmore P. A web-based approach to address cardiovascular risks in managers: results of a randomized trial. J Occup Environ Med 2011 Aug;53(8):911–8. 10.1097/JOM.0b013e3182258bd8 21785368 PMC3160446

[54] Blank C, Gatterer K, Leichtfried V, Pollhammer D, Mair-Raggautz M, Duschek S et al. Short Vacation Improves Stress-Level and Well-Being in German-Speaking Middle-Managers-A Randomized Controlled Trial. Int J Environ Res Public Health 2018 Jan;15(1):130. 10.3390/ijerph15010130 29342844 PMC5800229

[55] Cedstrand E, Augustsson H, Alderling M, Sánchez Martinez N, Bodin T, Nyberg A et al. Effects of a co-created occupational health intervention on stress and psychosocial working conditions within the construction industry: A controlled trial. Front Public Health 2022 Sep;10:973890. 10.3389/fpubh.2022.973890 36211695 PMC9542354

[56] Deval C, Bernard-Curie S, Monestès JL. Effects of an acceptance and commitment therapy intervention on leaders’ and managers’ psychological flexibility. J Ther Comport Cogn 2017;27(1):34–42. 10.1016/j.jtcc.2016.10.002

[57] Gast M, Lehmann J, Schwarz E, Hirning C, Hoelzer M, Guendel H et al. A Single-Day Training for Managers Reduces Cognitive Stigma Regarding Mental Health Problems: A Randomized Trial. Int J Environ Res Public Health 2022 Mar;19(7):4139. 10.3390/ijerph19074139 35409821 PMC8998400

[58] Igu NC, Onyishi CN, Amujiri BA, Binuomote MO, Modebelu MN, Okafor IP et al. Raising Leadership Self-Efficacy and Minimizing Organizational Burnout Among School Administrators in a GROW Model of Cognitive Behavioral Coaching. J Leadersh Organ Stud 2023;30(4):464–82. 10.1177/15480518231171748

[59] Janka A, Adler C, Brunner B, Oppenrieder S, Duschek S. Biofeedback Training in Crisis Managers: A Randomized Controlled Trial. Appl Psychophysiol Biofeedback 2017 Jun;42(2):117–25. 10.1007/s10484-017-9360-6 28349228

[60] Limm H, Gündel H, Heinmüller M, Marten-Mittag B, Nater UM, Siegrist J et al. Stress management interventions in the workplace improve stress reactivity: a randomised controlled trial. Occup Environ Med 2011 Feb;68(2):126–33. 10.1136/oem.2009.054148 20833759

[61] Ni D, Zheng X, Liang LH. How and when leader mindfulness influences team member interpersonal behavior: evidence from a quasi-field experiment and a field survey. Hum Relat 2023;76(12):1940–65. 10.1177/00187267221128571

[62] Ly KH, Asplund K, Andersson G. Stress management for middle managers via an acceptance and commitment-based smartphone application: A randomized controlled trial. Internet Interv 2014;1(3):95–101. 10.1016/j.invent.2014.06.003

[63] Martin A, Kilpatrick M, Scott J, Cocker F, Dawkins S, Brough P et al. Protecting the Mental Health of Small-to-Medium Enterprise Owners: A Randomized Control Trial Evaluating a Self-Administered Versus Telephone Supported Intervention. J Occup Environ Med 2020 Jul;62(7):503–10. 10.1097/JOM.0000000000001882 32730026 PMC7337118

[64] Mellner C, Osika W, Niemi M. Mindfulness practice improves managers’ job demands-resources, psychological detachment, work-nonwork boundary control, and work-life balance – a randomized controlled trial. IJWHM 2022;15(4):493–514. 10.1108/IJWHM-07-2021-0146

[65] Nübold A, van Quaquebeke N, Hülsheger UR. Be(com)ing Real: a Multi-source and an Intervention Study on Mindfulness and Authentic Leadership. J Bus Psychol 2020;35(4):469–88. 10.1007/s10869-019-09633-y

[66] Reitz M, Waller L, Chaskalson M, Olivier S, Rupprecht S. Developing leaders through mindfulness practice. JMD 2020;39(2):223–39. 10.1108/JMD-09-2018-026433271368

[67] Sawyer AT, Tao H, Bailey AK. The Impact of a Psychoeducational Group Program on the Mental Well-Being of Unit-Based Nurse Leaders: A Randomized Controlled Trial. Int J Environ Res Public Health 2023 Jun;20(11):6035. 10.3390/ijerph20116035 37297639 PMC10252280

[68] Shonin E, van Gordon W, Dunn TJ, Singh NN, Griffiths MD. Meditation Awareness Training (MAT) for Work-related Wellbeing and Job Performance: A Randomised Controlled Trial. Int J Ment Health Addict 2014;12(6):806–23. 10.1007/s11469-014-9513-2

[69] Vonderlin R, Müller G, Schmidt B, Biermann M, Kleindienst N, Bohus M et al. Effectiveness of a mindfulness- and skill-based health-promoting leadership intervention on supervisor and employee levels: A quasi-experimental multisite field study. J Occup Health Psychol 2021 Dec;26(6):613–28. 10.1037/ocp0000301 34591521

[70] Wasylkiw L, Holton J, Azar R, Cook W. The impact of mindfulness on leadership effectiveness in a health care setting: a pilot study. J Health Organ Manag 2015;29(7):893–911. 10.1108/JHOM-06-2014-0099 26556157

[71] Yong JS, Park JF, Park Y, Lee H, Lee G, Rim S. Effects of Holy Name Meditation on the Quality of Life of Hospital Middle Manager Nurses in Korea: A 6-Month Follow-Up. J Contin Educ Nurs 2020 May;51(5):215–24. 10.3928/00220124-20200415-06 32347958

[72] Żołnierczyk-Zreda D, Sanderson M, Bedyńska S. Mindfulness-based stress reduction for managers: a randomized controlled study. Occup Med (Lond) 2016 Nov;66(8):630–5. 10.1093/occmed/kqw091 27440398

[73] Greenwood K, Anas J. It's a New Era for mental health at work. Harvard Business Review [Internet] 2021 [cited 2024 Jul 15]. Available from: https://hbr.org/2021/10/its-a-new-era-for-mental-health-at-work

[74] Meyer M, Meinicke M, Schenkel A. Krankheitsbedingte Fehlzeiten in der deutschen Wirtschaft im Jahr 2022 [Sickness-related absences in the German economy in 2022]. In: Badura B, Ducki A, Baumgardt J, Meyer M, Schröder H, editors. Fehlzeiten-Report 2023. Fehlzeiten-Report. Berlin, Heidelberg: Springer Berlin Heidelberg; 2023. p. 435–520.

[75] Wittmers A, Maier GW. Leaders’ mental health in times of crisis: work intensification, emotional demands and the moderating role of organizational support and self-efficacy. Front Psychol 2023 May;14:1122881. 10.3389/fpsyg.2023.1122881 37205088 PMC10186101

[76] Hassard J, Teoh KR, Visockaite G, Dewe P, Cox T. The cost of work-related stress to society: A systematic review. J Occup Health Psychol 2018 Jan;23(1):1–17. 10.1037/ocp0000069 28358567

[77] Bartlett L, Martin A, Neil AL, Memish K, Otahal P, Kilpatrick M et al. A systematic review and meta-analysis of workplace mindfulness training randomized controlled trials. J Occup Health Psychol 2019 Feb;24(1):108–26. 10.1037/ocp0000146 30714811

[78] Vonderlin R, Biermann M, Bohus M, Lyssenko L. Mindfulness-Based Programs in the Workplace: a Meta-Analysis of Randomized Controlled Trials. Mindfulness (N Y) 2020;56(7):721. 10.1007/s12671-020-01328-3

[79] Michaelsen MM, Graser J, Onescheit M, Tuma MP, Werdecker L, Pieper D et al. Mindfulness-Based and Mindfulness-Informed Interventions at the Workplace: A Systematic Review and Meta-Regression Analysis of RCTs. Mindfulness (N Y) 2023 May;(May):1–34. 10.1007/s12671-023-02130-7 37362186 PMC10172073

[80] Kuehnl A, Seubert C, Rehfuess E, von Elm E, Nowak D, Glaser J. Human resource management training of supervisors for improving health and well-being of employees. Cochrane Database Syst Rev 2019 Sep;9(9):CD010905. 10.1002/14651858.CD010905.pub2 31560414 PMC6764461

[81] Kristensen TS. Intervention studies in occupational epidemiology. Occup Environ Med 2005 Mar;62(3):205–10. 10.1136/oem.2004.016097 15723887 PMC1740975

[82] Bellarosa C, Chen PY. The effectiveness and practicality of occupational stress management interventions: a survey of subject matter expert opinions. J Occup Health Psychol 1997 Jul;2(3):247–62. 10.1037/1076-8998.2.3.247 9552295

[83] Bhui KS, Dinos S, Stansfeld SA, White PD. A synthesis of the evidence for managing stress at work: a review of the reviews reporting on anxiety, depression, and absenteeism. J Environ Public Health 2012;2012:515874. 10.1155/2012/515874 22496705 PMC3306941

[84] Dimoff JK, Kelloway EK. With a little help from my boss: the impact of workplace mental health training on leader behaviors and employee resource utilization. J Occup Health Psychol 2019 Feb;24(1):4–19. 10.1037/ocp0000126 29939045

[85] Shann C, Martin A, Chester A, Ruddock S. Effectiveness and application of an online leadership intervention to promote mental health and reduce depression-related stigma in organizations. J Occup Health Psychol 2019 Feb;24(1):20–35. 10.1037/ocp0000110 29300098

[86] Hurrelmann K, Richter M. Determinaten der Gesundheit [Determinants of health]. In: Bundeszentrale für gesundheitliche Aufklärung (BzgA), editor. Leitbegriffe der Gesundheitsförderung und Prävention.

[87] Ernst E, Berg J, Moore PV. Editorial: Artificial intelligence and the future of work: humans in control. Front Artif Intell 2024 Mar;7:1378893. 10.3389/frai.2024.1378893 38545375 PMC10966127

