# Supplementary material for: Effectiveness of leader-targeted stress management interventions: A systematic review and meta-analysis
Source: Scand J Work Environ Health. 2025 Jun 26;51(4):265–81. doi: 10.5271/sjweh.4219 (PMC12278447; doi:10.5271/sjweh.4219)
Supplement: Supplementary material [file SJWEH-51-265-S001.pdf]

# Effectiveness of leader-targeted stress management interventions: A systematic review and meta-analysis[1]

***by Indra Dannheim, MA,[2] Helena Ludwig-Walz, Dr, Halina Kirsch, MSc, Martin Bujard, Prof, Dr, Anette E Buyken, Prof, Dr, Katherine Richardson, Prof, PhD, Anja Kroke, Prof, Dr***

1. Supplementary materials
2. Correspondence to: Indra Dannheim, M.A., Fulda University of Applied Sciences, Leipziger Straße 123, 36037 Fulda, Germany. [E-mail: indra.dannheim@oe.hs-fulda.de]

S1. PRISMA item checklist for systematic reviews ....................2
S2. Search Strategy ....................7
S3. Reasons for exclusion of after full-text screening ....................9
S4. Applied measurement instruments in included studies ....................10
S5. Description of subcategories ....................15
S6. Summary of details on risk of bias (RoB) assessment in included studies ....................16
S7. Narrative study results ....................114
S8. Meta-analysis results and sensitivity analyses of all analyzed interventions ....................118
S9. Meta-analysis results and sensitivity analyses of main level outcome categories ....................120
S10. Meta-analysis results and sensitivity analyses of identified subcategories ....................128
S11. Results of subgroup analyses ....................133
S12. Results of meta-regression ....................137
S13. Funnel plots for detection of publication bias ....................138
S14. Eggers´ test ....................148
S15. GRADE evidence profile ....................149
S16. Traffic-light plots of the domain-level judgements for each individual result ....................156
S17. Weighted-bar plots of risk of bias judgements within each bias domain ....................158
S18. References ....................159

# S1. PRISMA item checklist for systematic reviews

| Section and Topic | Item # | Checklist item | Location where item is reported |
|---|---|---|---|
| TITLE | | | |
| Title | 1 | Identify the report as a systematic review. | Title |
| ABSTRACT | | | |
| Abstract | 2 | See the PRISMA 2020 for Abstracts checklist. | not applicable |
| INTRODUCTION | | | |
| Rationale | 3 | Describe the rationale for the review in the context of existing knowledge. | Background |
| Objectives | 4 | Provide an explicit statement of the objective(s) or question(s) the review addresses. | Background |
| METHODS | | | |
| Eligibility criteria | 5 | Specify the inclusion and exclusion criteria for the review and how studies were grouped for the syntheses. | Methods: Inclusion and exclusion criteria |
| Information sources | 6 | Specify all databases, registers, websites, organisations, reference lists and other sources searched or consulted to identify studies. Specify the date when each source was last searched or consulted. | Methods: Data sources; |
| Search strategy | 7 | Present the full search strategies for all databases, registers and websites, including any filters and limits used. | AF: S2 |
| Selection process | 8 | Specify the methods used to decide whether a study met the inclusion criteria of the review, including how many reviewers screened each record and each report retrieved, whether they worked independently, and if applicable, details of automation tools used in the process. | Methods: Inclusion and exclusion criteria and Study selection; AF: S4 |
| Data collection process | 9 | Specify the methods used to collect data from reports, including how many reviewers collected data from each report, whether they worked independently, any processes for obtaining or confirming data from study investigators, and if applicable, details of automation tools used in the process. | Methods: Study selection and Data extraction |

| Section and Topic | Item # | Checklist item | Location where item is reported |
|---|---|---|---|
| Data items | 10a | List and define all outcomes for which data were sought. Specify whether all results that were compatible with each outcome domain in each study were sought (e.g. for all measures, time points, analyses), and if not, the methods used to decide which results to collect. | Methods: Coding of Study Characteristics, Synthesis Methods and AF: S5 and S6 |
| | 10b | List and define all other variables for which data were sought (e.g. participant and intervention characteristics, funding sources). Describe any assumptions made about any missing or unclear information. | Methods: Coding of Study Characteristics and Synthesis Methods |
| Study risk of bias assessment | 11 | Specify the methods used to assess risk of bias in the included studies, including details of the tool(s) used, how many reviewers assessed each study and whether they worked independently, and if applicable, details of automation tools used in the process. | Methods: Risk of Bias Assessment; AF: S7 |
| Effect measures | 12 | Specify for each outcome the effect measure(s) (e.g. risk ratio, mean difference) used in the synthesis or presentation of results. | Methods: Synthesis Methods |
| Synthesis methods | 13a | Describe the processes used to decide which studies were eligible for each synthesis (e.g. tabulating the study intervention characteristics and comparing against the planned groups for each synthesis (item #5)). | Methods: Synthesis Methods |
| | 13b | Describe any methods required to prepare the data for presentation or synthesis, such as handling of missing summary statistics, or data conversions. | Methods: Synthesis Methods |
| | 13c | Describe any methods used to tabulate or visually display results of individual studies and syntheses. | Methods: Synthesis Methods |
| | 13d | Describe any methods used to synthesize results and provide a rationale for the choice(s). If meta-analysis was performed, describe the model(s), method(s) to identify the presence and extent of statistical heterogeneity, and software package(s) used. | Methods: Synthesis Methods |

| Section and Topic | Item # | Checklist item | Location where item is reported |
|---|---|---|---|
| | 13e | Describe any methods used to explore possible causes of heterogeneity among study results (e.g. subgroup analysis, meta-regression). | Methods: Synthesis Methods |
| | 13f | Describe any sensitivity analyses conducted to assess robustness of the synthesized results. | Methods: Synthesis Methods |
| Reporting bias assessment | 14 | Describe any methods used to assess risk of bias due to missing results in a synthesis (arising from reporting biases). | Methods: Synthesis Methods |
| Certainty assessment | 15 | Describe any methods used to assess certainty (or confidence) in the body of evidence for an outcome. | Methods: Certainty of Evidence Assessment |
| RESULTS | | | |
| Study selection | 16a | Describe the results of the search and selection process, from the number of records identified in the search to the number of studies included in the review, ideally using a flow diagram. | Results, AF: S3 |
| | 16b | Cite studies that might appear to meet the inclusion criteria, but which were excluded, and explain why they were excluded. | AF: S4 |
| Study characteristics | 17 | Cite each included study and present its characteristics. | Table 1 |
| Risk of bias in studies | 18 | Present assessments of risk of bias for each included study. | Results, Table 1, AF: S7, S14, S15 S17 and S18 |
| Results of individual studies | 19 | For all outcomes, present, for each study: (a) summary statistics for each group (where appropriate) and (b) an effect estimate and its precision (e.g. confidence/credible interval), ideally using structured tables or plots. | Table 2 and AF: S9, S10 and S11 |
| Results of syntheses | 20a | For each synthesis, briefly summarise the characteristics and risk of bias among contributing studies. | Results and Table 2 |

| Section and Topic | Item # | Checklist item | Location where item is reported |
|---|---|---|---|
| | 20b | Present results of all statistical syntheses conducted. If meta-analysis was done, present for each the summary estimate and its precision (e.g. confidence/credible interval) and measures of statistical heterogeneity. If comparing groups, describe the direction of the effect. | Results, Table 2 and AF: S9, S10 and S11 |
| | 20c | Present results of all investigations of possible causes of heterogeneity among study results. | Results and AF: S12 and S13 |
| | 20d | Present results of all sensitivity analyses conducted to assess the robustness of the synthesized results. | Results, Table 2 and AF: S9, S10 and S11 |
| Reporting biases | 21 | Present assessments of risk of bias due to missing results (arising from reporting biases) for each synthesis assessed. | Results, Table 2 and AF: S7, S14, S15, S17 and S18 |
| Certainty of evidence | 22 | Present assessments of certainty (or confidence) in the body of evidence for each outcome assessed. | Results, Table 2 and AF: S16 |
| DISCUSSION | | | |
| Discussion | 23a | Provide a general interpretation of the results in the context of other evidence. | Discussion |
| | 23b | Discuss any limitations of the evidence included in the review. | Discussion: Strengths and Limitations |
| | 23c | Discuss any limitations of the review processes used. | Discussion: Strengths and Limitations |
| | 23d | Discuss implications of the results for practice, policy, and future research. | Discussion |
| OTHER INFORMATION | | | |
| Registration and protocol | 24a | Provide registration information for the review, including register name and registration number, or state that the review was not registered. | Methods |
| | 24b | Indicate where the review protocol can be accessed, or state that a protocol was not prepared. | Methods |
| | 24c | Describe and explain any amendments to information provided at registration or in the protocol. | Methods |

| Section and Topic | Item # | Checklist item | Location where item is reported |
|---|---|---|---|
| Support | 25 | Describe sources of financial or non-financial support for the review, and the role of the funders or sponsors in the review. | not applicable |
| Competing interests | 26 | Declare any competing interests of review authors. | Conflict of interest |
| Availability of data, code and other materials | 27 | Report which of the following are publicly available and where they can be found: template data collection forms; data extracted from included studies; data used for all analyses; analytic code; any other materials used in the review. | Supplementary information (online) |

# S2. Search Strategy

## 2.1 Cochrane Library

| # | search terms |
|---|---|
| 1 | manager*:ti,ab or supervisor*:ti,ab or leader*:ti,ab or executive*:ti,ab |
| 2 | Work:ti,ab or workplace*:ti,ab or worksite*:ti,ab or organization*:ti,ab |
| 3 | #1 AND #2 |
| 4 | stress*:ti,ab or |
| 5 | #3 AND #4 |
| 6 | training*:ti,ab or program*:ti,ab or intervention*:ti,ab or coaching:ti,ab |
| 7 | #5 AND #6 |
| 8 | effect*:ti,ab OR evaluat*:ti,ab OR efficac*:ti,ab |
| 9 | #7 AND #8 |

## 2.2 MEDLINE (via PubMed)

| # | search terms |
|---|---|
| 1 | manager*[Title/Abstract] OR supervisor*[Title/Abstract] OR leader*[Title/Abstract] OR executive*[Title/Abstract] |
| 2 | Work[Title/Abstract] OR workplace*[Title/Abstract] OR worksite*[Title/Abstract] OR job*[Title/Abstract] OR organization*[ Title/Abstract] |
| 3 | #1 AND #2 |
| 4 | occupational stress[MeSH Terms] OR stress, psychological[MeSH Terms] OR stress*[Title/Abstract] OR strain*[Title/Abstract] |
| 5 | #3 AND #4 |
| 6 | training*[Title/Abstract] OR program*[Title/Abstract] OR intervention*[Title/Abstract] OR coaching*[Title/Abstract] |
| 7 | #5 AND #6 |
| 8 | effect*[Title/Abstract] OR evaluat*[Title/Abstract] OR program evaluation[MeSH Terms] OR efficac*[Title/Abstract] |
| 9 | 7 AND #8 |

## 2.3 PsycInfo (via EBSCOhost)

| S | search terms |
|---|---|
| 1 | TI ( manager* or supervisor* or leader* or executive* ) OR AB ( manager* or supervisor* or leader* or executive ) |
| 2 | TI ( Work or workplace* or worksite* OR job* OR organization* ) OR AB ( Work or workplace* or worksite* OR job* OR organization* ) |
| 3 | S1 AND S2 |
| 4 | TI ( Stress* ) OR AB ( Stress* ) OR DE "Occupational Stress" OR MM "Perceived Stress" OR DE “Psychological Stress” |
| 5 | S3 AND S4 |
| 6 | TI (training* or program* or intervention* or coaching*) OR AB (training* or program* or intervention* or coaching*) OR MM ("Intervention") |
| 7 | S5 AND S6 |
| 8 | TI ( effect* OR evaluat* OR efficac* ) OR AB ( effect* OR evaluat* efficac* ) OR MM ("Evaluation") |
| 9 | S7 AND S8 |

## 2.4 Web of Science Core Collection

| # | search terms |
|---|---|
| 1 | TS=(manager* or supervisor* or leader* or executive*) |
| 2 | TS=(Work or workplace* or worksite* or job* or organization*) |
| 3 | #1 AND #2 |
| 4 | TS=(stress*) |
| 5 | #3 AND #4 |
| 6 | TS=(training* or program* or intervention* or coaching*) |
| 7 | #5 AND #6 |
| 8 | TS=(effect* OR evaluat* or efficac*) |
| 9 | #7 and #8 |

# S3. Reasons for exclusion of after full-text screening

| # | Study | Reason for exclusion |
|---|---|---|
| 1 | Balint (1) | No relevant outcomes |
| 2 | Bernardez (2) | No separate analysis for supervisors |
| 3 | Biggs (3) | No separate analysis for supervisors |
| 4 | Born (4) | Inappropriate study design: No control group |
| 5 | Carlisle (5) | Inappropriate study design; no control group at pre-measurement, only at post-measurement |
| 6 | Coetzer (6) | Inappropriate study design: No control group |
| 7 | Dalgaard (7) | Study protocol. Contact with responding author on Nov. 21st, 2023. To this time, no published or unpublished data available. |
| 8 | DRKS00023457 (8) | Trail registration, data has been published in Lehmann (9) |
| 9 | Grant (10) | Inappropriate intervention |
| 10 | Gray (11) | No separate analysis for supervisors |
| 11 | Harenstam (2022) (12) | Inappropriate intervention; no control group |
| 12 | Iodice (2022) (13) | No relevant outcomes |
| 13 | ISRCTN14104664 (2022) | Study protocol, which was published in Arensman (14). Contacted responding author on Nov. 21st. No response. |
| 14 | Jeon (15) | Inappropriate intervention |
| 15 | Kawakami (16) | Inappropriate study design: No appropriate control group |
| 16 | Kersemaekers (17) | Inappropriate study design: No appropriate control group |
| 17 | Lehmann (9) | Study protocol. Contacted responding author on Nov. 21st. No response. |
| 18 | MacPhee (18) | Inappropriate intervention |
| 19 | Maddi (19) | No study effects reported |
| 20 | NCT04727255 (20) | Trail registration. Contacted author on Nov. 21st. No response. |
| 21 | NCT05154019 (21) | Trial registration. Contact with responding author on Nov. 21st, 2023. To this time, no published or unpublished data available. |
| 22 | Nielsen (22) | Inappropriate intervention |
| 23 | Nishiuchi (23) | No relevant outcomes |
| 24 | Pipe (24) | Contacted authors on December 18th to provide data for relevant outcomes. Contact was not successful and therefore no data could be provided. Consequently, exclusion due to no study effects reported. |
| 25 | Sreekumar (25) | Inappropriate study design |
| 26 | Tan (26) | No separate analysis for supervisors |
| 27 | Zheng (27) | No separate analysis for supervisors |
| 28 | Zimber (28) | No separate analysis for supervisors |
| Not retrieved | De Armond (29) | Dissertation. It could not be detected over Google, Google scholar, researchgate or library service and library association of Fulda´s University of Applied Science. |

# S4. Applied measurement instruments in included studies

| First author (year) | Applied measurement instruments |
|---|---|
| Allen (1980) (30)<br><br>Excluded from meta-analysis due to lack of available or insufficient data. | Psychological stress:<br>1. Subjectively experienced stress. 4 items, Subjective Stress Scale(31)<br>Mindfulness: none<br>Mental health:<br>1. State anxiety. 20 items, State-Trait Anxiety Inventory (32)<br>2. Trait anxiety. 20 items, State-Trait Anxiety Inventory (32)<br>Work-related outcomes:<br>1. Job Performance. 4 items, (33)<br>Leadership-related outcomes: none |
| Bennett (2011) (34) | Psychological stress: none<br>Mindfulness: none<br>Mental health:<br>1. Symptoms of distress. 15 items Scale capturing the frequency of various physical and emotional symptoms of stress (35)<br>Work-related outcomes: none<br>Leadership-related outcomes: none |
| Blank (2018) (36) | Psychological stress:<br>1. Perceived stress. 20 items, Short German version of Perceived Stress Questionnaire (PSQ) (37,38)<br>Mindfulness: none<br>Mental health: none<br>Work-related outcomes: none<br>Leadership-related outcomes: none |
| Cedstrand (2022) (39) | Stress:<br>1. Stress. 3 items, Copenhagen Psychosocial Questionnaire (COPSOQ) III (40,41)<br>Mindfulness: none<br>Mental health: none<br>Work-related outcomes: none<br>Leadership-related outcomes: none |
| Deval (2017) (42) | Psychological stress:<br>1. Perceived stress. 10 items, French version of the Perceived Stress Scale (PSS-10) (43)<br>Mindfulness:<br>1. Trait mindfulness. 15 items, French version of the Mindful Attention and Awareness Scale (MAAS) (44)<br>Mental health:<br>1. General mental health, 12 items, French version of the General Health Questionnaire (GHQ-12) (45)<br>Work-related outcomes:<br>1. Job Satisfaction. 15 items, Job Satisfaction subscale of Warr's work attitudes and aspects of psychological wellbeing scales (WARR) (46)<br>2. Career satisfaction. 5 items, French adaptation of the Satisfaction With Life Scale (ESVP) (47)<br>Leadership-related outcomes: none |

| | |
|---|---|
| Gast (2022) (48)<br><br>Excluded from meta-analysis due to lack of available or insufficient data. | Psychological stress:<br>1. Stress in general. 4 items, Perceived Stress Scale (PSS-4) (49,50)<br>2. Effort-reward ratio: Effort, Reward. 17 items, Effort-reward imbalance (ERI) model (51)<br>3. Irritation. 8 items, Irritation Scale (IS-8) (52)<br>Mindfulness: none<br>Mental health:<br>1. Symptoms of depression and anxiety. 4 items, Patient Health Questionnaire (PHQ-4) (53)<br>Work-related outcomes: none<br>Leadership-related outcomes: none |
| Igu (2023) (54) | Psychological stress: none<br>Mindfulness: none<br>Mental health:<br>1. Burnout: Emotional exhaustion, depersonalization/cynicism, poor personal/professional accomplishments. 22 items, Burnout Inventory-Educators' Survey (MBI-ES) (55)<br>Work-related outcomes: none<br>Leadership-related outcomes: none |
| Janka (2017) (56)<br><br>Excluded from meta-analysis due to lack of available or insufficient data. | Psychological stress:<br>1. General subjective stress. 10 items, German version of the perceived stress scale (PSS-10) (50)<br>Mindfulness: none<br>Mental health: none<br>Work-related outcomes: none<br>Leadership-related outcomes: none |
| Lange (2019) (57) | Psychological stress:<br>1. Irritation. 8 items, Irritation Scale (58)<br>Mindfulness:<br>1. Mindfulness. 24 items, short (59) and German (60) version of Five Facet Mindfulness Questionnaire (FFMQ) (61)<br>Mental health: none<br>Work-related outcomes: none<br>Leadership related outcomes:<br>1. Abusive leadership. 5 items, Fragebogen zur Integrativen Führung (62)<br>2. Transformational leadership. 24 items, Fragebogen zur Integrativen Führung (62) |
| Li (2017) (63) | Psychological stress:<br>1. Effort-reward Ratio. 16 items, Effort-reward imbalance (ERI) model (51,64,65)<br>Mindfulness: none<br>Mental health (IG):<br>1. Depressive symptoms. 14 items, Hospital Anxiety and Depression Scale (HADS) (66)<br>Mental health (CG):<br>1. Depressive symptoms. 12 items, Short-Form 12 health survey (SF-12) (67)<br>Work-related outcomes: none<br>Leadership-related outcomes: none |
| Limm (2011) (68) | Psychological stress:<br>1. Stress reactivity. 29 items, Stress Reactivity Scale (SRS)<br>2. Effort-reward Ratio. 16 items, Effort-reward imbalance (ERI) model (51,64,65)<br>Mindfulness: none<br>Mental health:<br>1. Anxiety. 7 items, Hospital Anxiety and Depression Scale (HADS) (66)<br>2. Depression. 7 items, Hospital Anxiety and Depression Scale (HADS) (66) |

| | |
|---|---|
| | Work-related outcomes: none<br>Leadership-related outcomes: none |
| Ly (2014) (69) | Psychological stress:<br>1. Perceived stress. 14 items, Perceived Stress Scale (PSS-14) (50)<br>Mindfulness: none<br>Mental health:<br>1. General mental health, 12 items, General Health Questionnaire (GHQ-12) (70)<br>Work-related outcomes: none<br>Leadership-related outcomes:<br>1. Transformative leadership. 20 items, Swedish-validated version of Multifactor Leadership Questionnaire (MLQ) (71) |
| Martin (2020) (72)<br><br>Work-related outcomes were excluded from meta-analysis due to a lack of available data. | Psychological stress: none<br>Mindfulness: none<br>Mental health:<br>1. Psychological distress. 10 items, Kessler 10 (K10) Screening Scale for Psychological Distress (73)<br>Work-related outcomes:<br>1. Lost Productive Time (absenteeism days). 1 item, World Health Organizations Health and Work Performance Questionnaires (HPQ) (74)<br>2. Presenteeism. 2 items, Australian workforce context (75)<br>Leadership related outcomes: none |
| Mellner (2022) (76) | Psychological stress: none<br>Mindfulness:<br>1. Mindfulness. 39 items, Swedish validated version (77) of the Five Facet Mindfulness Questionnaire, FFMQ (61)<br>Mental health: none<br>Work-related outcomes:<br>Leadership-related outcomes: none |
| Munafo (2016) (78) | Psychological stress: none<br>Mindfulness: none<br>Mental health:<br>1. State or current anxiety (STAI-Y1). 20 items, State-Trait Anxiety Inventory (32,79)<br>2. Trait or dispositional anxiety (STAI-Y2). 20 items, State-Trait Anxiety Inventory (32,79)<br>Work-related outcomes: none<br>Leadership related outcomes: none |
| Ni (2022) (80) | Psychological stress: none<br>Mindfulness:<br>1. Mindfulness. 15-items, Mindful Attention and Awareness Scale (81)<br>Mental health: none<br>Work-related outcomes: none<br>Leadership related outcomes: none |
| Nübold (2020) (82) | Psychological stress: none<br>Mindfulness:<br>1. Leaders' mindfulness. 15 items, Mindfulness Attention and Awareness Scale (MAAS) (81)<br>Mental health: none<br>Work-related outcomes: none<br>Leadership related outcomes:<br>1. Authentic leadership. 14 items, Authentic Leadership Inventory (ALI) (83) |
| Reitz (2020) (84) | Psychological stress: none<br>Mindfulness:<br>1. Mindfulness. 39 items, Five Facet Mindfulness Questionnaire (FFMQ) (61) |

| | |
|---|---|
| | Mental health: none<br>Work-related outcomes: none<br>Leadership related outcomes:<br>1. Leadership competencies. 65 items, Competence Questionnaire (84) |
| Sawyer (2023) (85) | Psychological stress:<br>1. Perceived stress. 10 items, Perceived Stress Scale (PSS-10) (50)<br>2. Secondary Traumatic Stress (STS) Professional Quality of Life (ProQOL) Scale (86,87)<br>Mental health:<br>1. Burnout. 30 items, Professional Quality of Life (ProQOL) Scale (86,87)<br>Mindfulness: none<br>Work-related outcomes:<br>1. Job Satisfaction 4 items, Brief Index of Affective Job Satisfaction (BIAJS) (88)<br>Leadership related outcomes: none |
| Shonin (2014) (89) | Psychological stress:<br>1. Work-stress. 35 items, HSE Management Standards Work-Related Stress Indicator Tool (WSIT) (90)<br>Mindfulness: none<br>Mental health:<br>1. Emotional distress. 21 items, Depression, Anxiety, and Stress Scale (DASS) (91)<br>Work-related outcomes:<br>1. Global job satisfaction. 8 items, Abridged Job in General Scale (AJIGS) (92)<br>2. General work performance. 20 items, Role-Based Performance Scale (RBPS) (93)<br>Leadership related outcomes: none |
| Vonderlin (2021) (94) | Psychological stress: none<br>Mindfulness: none<br>Mental health:<br>1. Anxiety. 7 items, Hospital Anxiety and Depression Scale (HADS) (95,96)<br>2. Depression. 7 items, Hospital Anxiety and Depression Scale (HADS) (95,96)<br>Work-related outcomes: none<br>Leadership related outcomes:<br>1. Health promoting self-care: Awareness, Value, Behavior. 31 items, Health-Oriented Leadership Scale (97)<br>2. Health promoting staff-care, Awareness, Value, Behavior. 31 items, Health-Oriented Leadership Scale (97) |
| Vonderlin (2023) (98) | Psychological stress: none<br>Mindfulness: none<br>Mental health: none<br>Work-related outcomes:<br>1. Nonspecific sickness absence days. Health insurance data<br>Leadership related outcomes: none |
| Wasylkiw (2015) (99) | Psychological stress:<br>1. Perceived stress. 10 items, Perceived stress scale (50)<br>Mindfulness:<br>1. Mindfulness. 15 items, Mindful Attention Awareness Scale (81)<br>Mental health: none<br>Work-related outcomes: none<br>Leadership-related outcomes:<br>1. Leadership effectiveness. 5 items, Scale assessing leadership effectiveness (100,101)<br>2. Authentic leadership. 5 items, Authentic Leadership Questionnaire (ALQ) (102) |
| Yong (2020) (103) | Psychological stress: none |

| | |
|---|---|
| Excluded from meta-analysis due to lack of available or insufficient data. | Mindfulness: none<br>Mental health:<br>1. Burnout. 22 items, Burnout Inventory-Educators' Survey (MBI-ES) (104)<br>2. Anxiety & Depression: Korean version (105) of Symptom Checklist 90-Revised (106).<br>Work-related outcomes:<br>1. Job Satisfaction. Eight-item instrument (107).<br>Leadership-related outcomes:<br>1. Leadership practice. Leadership Practice Inventory (108). |
| Zolnierczyk-Zreda (2016) (109) | Psychological stress:<br>1. Work-related stress. 40 items, Occupational Stress Indicator (OSI-2) (110)<br>Mindfulness: none<br>Mental health:<br>1. Negative effect. 5 items, Bradburn's Affect Experience Index (AEI) (111)<br>Work-related outcomes:<br>1. Sickness absence. 1 item, 'For how many days were you absent from your job due to sickness during the past 3 months?'<br>Leadership related outcomes: none |

# S5. Description of subcategories

| Subcategories |
|---|
| **Job stress** refers to the assessment of psychological stress experienced in workplace settings (52). While the Occupational Stress Indicator (OSI-2) (110) is specifically designed to assess managerial stress, the effort-reward ratio measure (51) captures stressful experiences arising from an imbalance between high effort and low reward, highlighting the mismatch between job demands and individual resources or capabilities (51). |
| According to the Perceived Stress Scale (PSS) (37, 38), **perceived stress** is defined as the degree to which individuals appraise situations in their lives as stressful, encompassing the subjective assessment of stress levels. The PSS was developed by Cohen, Kamarck, and Mermelstein (50) and has been used in all studies that examined perceived stress. |
| **Subsyndromal symptoms** refer to a collection of psychological or emotional symptoms associated with anxiety, depression, and burnout. The Hospital Anxiety and Depression Scale (HADS) was the most commonly used measurement instrument (66). |
| **General mental health** refers to an individual's overall psychological well-being, including emotional, cognitive, and behavioral functioning (70). It was assessed using various instruments designed to capture the presence of psychological or emotional distress. |

# S6. Summary of details on risk of bias (RoB) assessment in included studies

**RoB in randomized controlled trials (RCTs)** were assessed by using the revised tool for assessing risk of bias in randomised trials (RoB 2), described in Sterne et al. (2019) (112).

**RoB in controlled-before-after studies (CBAs)** were assessed by using the ROBINS-I tool for non-randomized studies of interventions, described in Sterne et al. (2016) (113).

## 6.1 Randomized controlled trials

| **Bennett (34)** | | | | |
|---|---|---|---|---|
| Bias domain and signalling question* | Response options | | | Comments |
| | Lower risk of bias | Higher risk of bias | Other | |
| **Bias arising from the randomisation process** | | | | |
| 1.1 Was the allocation sequence random? | Y/PY | N/PN | NI | No clear information on randomization. |
| 1.2 Was the allocation sequence concealed until participants were enrolled and assigned to interventions? | Y/PY | N/PN | NI | No information on allocation concealment. |
| 1.3 Did baseline differences between intervention groups suggest a problem with the randomisation process? | N/PN | Y/PY | NI | "These comparisons identified only two significant differences: the experimental group was slightly younger (M = 39.7 vs 43.2, SD = 9.7, 10.6, respectively), t141 = –2.05, P = 0.04, and had a higher percentage of women (72% vs 56%), χ2 (1) = 4.06, P = 0.04. However, both represented only a modest correlation with condition assignment (r = –0.17 for age and 0.17 for gender), and both appeared in the analyses as covariates, adjusting for any potential bias. |
| Risk-of-bias judgment (low/high/some concerns) | Low risk of bias | | | For judgement we followed the suggested algorithm process: 1.2.: NI → 1.3.: N/PN → Some concerns |
| **Bias due to deviations from intended interventions** | | | | |

| | | | | |
|---|---|---|---|---|
| 2.1 Were participants aware of their assigned intervention during the trial? | N/PN | Y/PY | NI | "Managers in the control group had no project-related training or activities." |
| 2.2 Were carers and people delivering the interventions aware of participants' assigned intervention during the trial? | N/PN | Y/PY | NI | Internet-based program |
| 2.3 If Y/PY/NI to 2.1 or 2.2: Were there deviations from the intended intervention that arose because of the trial context? | N/PN | Y/PY | NA/NI | No information |
| 2.4 If Y/PY/NI to 2.3: Were these deviations likely to have affected the outcome? | N/PN | Y/PY | NA/NI | NA |
| 2.5 If Y/PY to 2.4: Were these deviations from intended intervention balanced between groups? | Y/PY | N/PN | NA/NI | NA |
| 2.6 Was an appropriate analysis used to estimate the effect of assignment to intervention? | Y/PY | N/PN | NI | "The current study examined overall effectiveness using an "intention-to-treat" approach" |
| 2.7 If N/PN/NI to 2.6: Was there potential for a substantial impact (on the result) of the failure to analyse participants in the group to which they were randomised? | N/PN | Y/PY | NA/NI | |
| Risk-of-bias judgment (low/high/some concerns) | Some concerns | | | For judgement we followed the suggested algorithm process:<br>Part 1: 2.1: Y/PY & 2.2: N/PN → 2.3: NI → Some concerns<br>Part 2: 2.6: Y/PY → Low risk<br>Part 1: Some concerns AND Part2: low risk → Some concerns |
| **Bias due to missing outcome data** | | | | |
| 3.1 Were data for this outcome available for all, or nearly all, participants randomised? | Y/PY | N/PN | NI | IG: 47 completed 6-month follow-up (65%)<br>EG: 62 completed 6-month follow-up (85%) |

| | | | | |
|---|---|---|---|---|
| 3.2 If N/PN/NI to 3.1: Is there evidence that the result was not biased by missing outcome data? | Y/PY | N/PN | NA | "These findings, combined with an analytic approach that incorporated incomplete data, suggested attrition was unlikely to bias study results substantially." |
| 3.3 If N/PN to 3.2: Could missingness in the outcome depend on its true value? | N/PN | Y/PY | NA/NI | NA |
| 3.4 If Y/PY/NI to 3.3: Is it likely that missingness in the outcome depended on its true value? | N/PN | Y/PY | NA/NI | NA |
| Risk-of-bias judgment (low/high/some concerns) | Low risk of bias | | | For judgement we followed the suggested algorithm process: 3.1: N/PN → 3.2: Y/PY → low risk |
| **Bias in measurement of the outcome** | | | | |
| 4.1 Was the method of measuring the outcome inappropriate? | N/PN | Y/PY | NI | Pre-specified outcomes |
| 4.2 Could measurement or ascertainment of the outcome have differed between intervention groups? | N/PN | Y/PY | NI | Same measurements/ascertainments of the outcome |
| 4.3 If N/PN/NI to 4.1 and 4.2: Were outcome assessors aware of the intervention received by study participants? | N/PN | Y/PY | NI | Participant-reported outcomes |
| 4.4 If Y/PY/NI to 4.3: Could assessment of the outcome have been influenced by knowledge of intervention received? | N/PN | Y/PY | NA/NI | Participants had knowledge about the assigned intervention (see also 2.1) |
| 4.5 If Y/PY/NI to 4.4: Is it likely that assessment of the outcome was influenced by knowledge of intervention received? | N/PN | Y/PY | NA/NI | Knowledge of intervention status could have influenced outcome assessment but there is no reason to believe that it did due to 6-month follow-up |
| Risk-of-bias judgment (low/high/some concerns) | Some concerns | | | For judgement we followed the suggested algorithm process: 4.1: N/PN → 4.2: N/PN → 4.3: Y/PY → 4.4: Y/PY → 4.5: N/PN → some concerns |
| **Bias in selection of the reported result** | | | | |
| 5.1 Were the data that produced this result analysed in accordance with a prespecified analysis plan that | Y/PY | N/PN | NI | No study protocol available |

| | | | | |
|---|---|---|---|---|
| was finalised before unblinded outcome data were available for analysis? | | | | |
| Is the numerical result being assessed likely to have been selected, on the basis of the results, from: | | | | |
| 5.2 ... multiple eligible outcome measurements (eg, scales, definitions, time points) within the outcome domain? | N/PN | Y/PY | NI | See 5.1. Analysis intentions are not available |
| 5.3 ... multiple eligible analyses of the data? | N/PN | Y/PY | NI | See 5.1. Analysis intentions are not available |
| Risk-of-bias judgment (low/high/some concerns) | Some concerns | | | For judgement we followed the suggested algorithm process: 5.2: NI & 5.3: NI → Some concerns |
| **Overall bias** | | | | |
| Risk-of-bias judgment (low/high/some concerns) | Some concerns | | | The study is judged to raise some concerns in at least one domain for this result, but not to be at high risk of bias for any domain. |

| **Blank (36)** | | | | |
|---|---|---|---|---|
| Bias domain and signalling question* | Response options | | | Comments |
| | Lower risk of bias | Higher risk of bias | Other | |
| **Bias arising from the randomisation process** | | | | |
| 1.1 Was the allocation sequence random? | Y/PY | N/PN | NI | Only information is that stratified randomization was used. |
| 1.2 Was the allocation sequence concealed until participants were enrolled and assigned to interventions? | Y/PY | N/PN | NI | “To minimize a possible bias affecting subjective well-being from the joyful anticipation of going on vacation, participants were matched after they filled in the baseline questionnaires“ |

| | | | | |
|---|---|---|---|---|
| 1.3 Did baseline differences between intervention groups suggest a problem with the randomisation process? | N/PN | Y/PY | NI | Table 1.<br>“A successful randomization was confirmed by analyses of the baseline values of the matching criteria (age: p = 0.28; gender: p = 0.74; stress level: p = 0.42).” |
| Risk-of-bias judgment (low/high/some concerns) | Low risk | | | For judgement we followed the suggested algorithm process: 1.2: Y/PY → 1.1: Ni → 1.3: N/PN → low risk |
| **Bias due to deviations from intended interventions** | | | | |
| 2.1 Were participants aware of their assigned intervention during the trial? | N/PN | Y/PY | NI | Participants of the IG had to pay 150€ per stay. In addition, see description by 1.2. Consequently, it can be assumed that participants were aware of their assigned interventions during trail. |
| 2.2 Were carers and people delivering the interventions aware of participants’ assigned intervention during the trial? | N/PN | Y/PY | NI | Due to stratified randomization, it can be assumed that outcome assessors knew about the intervention received by study participants. |
| 2.3 If Y/PY/NI to 2.1 or 2.2: Were there deviations from the intended intervention that arose because of the trial context? | N/PN | Y/PY | NA/NI | No information on deviations. |
| 2.4 If Y/PY/NI to 2.3: Were these deviations likely to have affected the outcome? | N/PN | Y/PY | NA/NI | NA |
| 2.5 If Y/PY to 2.4: Were these deviations from intended intervention balanced between groups? | Y/PY | N/PN | NA/NI | NA |
| 2.6 Was an appropriate analysis used to estimate the effect of assignment to intervention? | Y/PY | N/PN | NI | ITT Analysis |
| 2.7 If N/PN/NI to 2.6: Was there potential for a substantial impact (on the result) of the failure to analyse participants in the group to which they were randomised? | N/PN | Y/PY | NA/NI | NA |
| Risk-of-bias judgment (low/high/some concerns) | Some concerns | | | For judgement we followed the suggested algorithm process: |

| | | | | |
|---|---|---|---|---|
| | | | | Part 1: 2.1: Y/PY / 2.2: Y/PY → 2.3: NI → Some concerns<br>Part 2: 2.6: Y/PY → Low risk<br>Part 1 & Part 2: Some concerns in Part 1 → Some concerns |
| **Bias due to missing outcome data** | | | | |
| 3.1 Were data for this outcome available for all, or nearly all, participants randomised? | Y/PY | N/PN | NI | No missing data. |
| 3.2 If N/PN/NI to 3.1: Is there evidence that the result was not biased by missing outcome data? | Y/PY | N/PN | NA | NA |
| 3.3 If N/PN to 3.2: Could missingness in the outcome depend on its true value? | N/PN | Y/PY | NA/NI | NA |
| 3.4 If Y/PY/NI to 3.3: Is it likely that missingness in the outcome depended on its true value? | N/PN | Y/PY | NA/NI | NA |
| Risk-of-bias judgment (low/high/some concerns) | Low risk of bias | | | For judgement we followed the suggested algorithm process:<br>3.1: Y/PY → Low risk |
| **Bias in measurement of the outcome** | | | | |
| 4.1 Was the method of measuring the outcome inappropriate? | N/PN | Y/PY | NI | Short German version of Perceived Stress Questionnaire (PSQ) (Fliege et al. 2005; Levenstein et al. 1993) |
| 4.2 Could measurement or ascertainment of the outcome have differed between intervention groups? | N/PN | Y/PY | NI | Same measurement instrument and measurement point |
| 4.3 If N/PN/NI to 4.1 and 4.2: Were outcome assessors aware of the intervention received by study participants? | N/PN | Y/PY | NI | Participant reported outcomes. |
| 4.4 If Y/PY/NI to 4.3: Could assessment of the outcome have been influenced by knowledge of intervention received? | N/PN | Y/PY | NA/NI | Assessment of the outcome could have been influenced |

| | | | | |
|---|---|---|---|---|
| 4.5 If Y/PY/NI to 4.4: Is it likely that assessment of the outcome was influenced by knowledge of intervention received? | N/PN | Y/PY | NA/NI | Knowledge of intervention status could have influenced outcome assessment but there is no reason to believe that it did. |
| Risk-of-bias judgment (low/high/some concerns) | Some concerns | | | For judgement we followed the suggested algorithm process:<br>4.1: N/PN → 4.2: N/PN → 4.3: Y/PY → 4.4: Y/PY → 4.5: Y/PY → Some concerns |
| **Bias in selection of the reported result** | | | | |
| 5.1 Were the data that produced this result analysed in accordance with a prespecified analysis plan that was finalised before unblinded outcome data were available for analysis? | Y/PY | N/PN | NI | No study protocol available |
| Is the numerical result being assessed likely to have been selected, on the basis of the results, from: | | | | |
| 5.2 ... multiple eligible outcome measurements (eg, scales, definitions, time points) within the outcome domain? | N/PN | Y/PY | NI | See 5.1. Analysis intentions are not available |
| 5.3 ... multiple eligible analyses of the data? | N/PN | Y/PY | NI | See 5.1. Analysis intentions are not available |
| Risk-of-bias judgment (low/high/some concerns) | Some concerns | | | For judgement we followed the suggested algorithm process:<br>5.2: NI / 5.3: NI → Some concerns |
| **Overall bias** | | | | |
| Risk-of-bias judgment (low/high/some concerns) | Some concerns | | | The study is judged to raise some concerns in at least one domain for this result, but not to be at high risk of bias for any domain. |

| **Gast (48)** | | |
|---|---|---|
| Bias domain and signalling question* | Response options | Comments |

| | Lower risk of bias | Higher risk of bias | Other | |
|---|---|---|---|---|
| **Bias arising from the randomisation process** | | | | |
| 1.1 Was the allocation sequence random? | Y/PY | N/PN | NI | Only information is that stratified randomization was used. |
| 1.2 Was the allocation sequence concealed until participants were enrolled and assigned to interventions? | Y/PY | N/PN | NI | No information |
| 1.3 Did baseline differences between intervention groups suggest a problem with the randomisation process? | N/PN | Y/PY | NI | Table 1 |
| Risk-of-bias judgment (low/high/some concerns) | Some concerns | | | For judgement we followed the suggested algorithm process:<br>1.2: NI → 1.3: N/PN → Some concerns |
| **Bias due to deviations from intended interventions** | | | | |
| 2.1 Were participants aware of their assigned intervention during the trial? | N/PN | Y/PY | NI | Waitlist control and "Managers randomized to the intervention group received sign-in dates for the training." |
| 2.2 Were carers and people delivering the interventions aware of participants' assigned intervention during the trial? | N/PN | Y/PY | NI | Following information is available:<br>"Subsequently, the Institute for Epidemiology and Biometry of the University of Ulm randomly assigned the participants to either the intervention or waiting-list control group."<br>„The workshops were each led by a specialist in psychosomatic medicine and psychotherapy, accompanied by another physician so that there were two trainers present per workshop."<br>However, it is unclear whether the trainers were part of the people delivering the intervention. |

| | | | | |
|---|---|---|---|---|
| 2.3 If Y/PY/NI to 2.1 or 2.2: Were there deviations from the intended intervention that arose because of the trial context? | N/PN | Y/PY | NA/NI | No information on deviations |
| 2.4 If Y/PY/NI to 2.3: Were these deviations likely to have affected the outcome? | N/PN | Y/PY | NA/NI | NA |
| 2.5 If Y/PY to 2.4: Were these deviations from intended intervention balanced between groups? | Y/PY | N/PN | NA/NI | NA |
| 2.6 Was an appropriate analysis used to estimate the effect of assignment to intervention? | Y/PY | N/PN | NI | Per protocol analysis |
| 2.7 If N/PN/NI to 2.6: Was there potential for a substantial impact (on the result) of the failure to analyse participants in the group to which they were randomised? | N/PN | Y/PY | NA/NI | PY because of the low sample size. |
| Risk-of-bias judgment (low/high/some concerns) | High risk of bias | | | For judgement we followed the suggested algorithm process:<br>Part 1: 2.1: Y/PY / 2.2: NI → 2.3: NI → Some concerns<br>Part 2: 2.6: N/PN → 2.7: Y/PY → High risk<br>Part 1: Low risk AND Part 2: High Risk → High Risk |
| **Bias due to missing outcome data** | | | | |
| 3.1 Were data for this outcome available for all, or nearly all, participants randomised? | Y/PY | N/PN | NI | High drop-out rate |
| 3.2 If N/PN/NI to 3.1: Is there evidence that the result was not biased by missing outcome data? | Y/PY | N/PN | NA | No information on sensitivity analyses or correction of missing outcome data. |
| 3.3 If N/PN to 3.2: Could missingness in the outcome depend on its true value? | N/PN | Y/PY | NA/NI | No information and could therefore depend on its true value |
| 3.4 If Y/PY/NI to 3.3: Is it likely that missingness in the outcome depended on its true value? | N/PN | YPY | NA/NI | Reasons for drop-out are: Not available at workshop date, Long-term sickness absence, Lost interest in topic, No response. Therefore, it can be assumed that drop-outs reasons do not depend on its true value. |

| | | | | |
|---|---|---|---|---|
| Risk-of-bias judgment (low/high/some concerns) | High risk of bias | | | For judgement we followed the suggested algorithm process:<br>3:1: N/PN → 3.2: N/PN → 3.3: Y/PY → 3.4: Y/PY → High risk |
| **Bias in measurement of the outcome** | | | | |
| 4.1 Was the method of measuring the outcome inappropriate? | N/PN | Y/PY | NI | Appropriate measurements |
| 4.2 Could measurement or ascertainment of the outcome have differed between intervention groups? | N/PN | Y/PY | NI | Same measurements/ascertainments of the outcome |
| 4.3 If N/PN/NI to 4.1 and 4.2: Were outcome assessors aware of the intervention received by study participants? | N/PN | Y/PY | NI | Participant-reported outcome |
| 4.4 If Y/PY/NI to 4.3: Could assessment of the outcome have been influenced by knowledge of intervention received? | N/PN | Y/PY | NA/NI | Assessment of the outcome could have been influenced |
| 4.5 If Y/PY/NI to 4.4: Is it likely that assessment of the outcome was influenced by knowledge of intervention received? | N/PN | Y/PY | NA/NI | Knowledge of intervention status could have influenced outcome assessment but there is no reason to believe that it did due to three-month follow-up |
| Risk-of-bias judgment (low/high/some concerns) | Some concerns | | | For judgement we followed the suggested algorithm process:<br>4.1: N/PN → 4.2: N/PN → 4.3: Y/PY → 4.4: Y/PY → 4.5: N/PN → Some concerns |
| **Bias in selection of the reported result** | | | | |
| 5.1 Were the data that produced this result analysed in accordance with a prespecified analysis plan that was finalised before unblinded outcome data were available for analysis? | Y/PY | N/PN | NI | No clear information |
| Is the numerical result being assessed likely to have been selected, on the basis of the results, from: | | | | |

| | | | | |
|---|---|---|---|---|
| 5.2 ... multiple eligible outcome measurements (eg, scales, definitions, time points) within the outcome domain? | N/PN | Y/PY | NI | Analysis intentions are not available |
| 5.3 ... multiple eligible analyses of the data? | N/PN | Y/PY | NI | Analysis intentions are not available |
| Risk-of-bias judgment (low/high/some concerns) | Some concerns | | | For judgement we followed the suggested algorithm process:<br>5.2: NI / 5.3: NI → Some concerns |
| **Overall bias** | | | | |
| Risk-of-bias judgment (low/high/some concerns) | High risk of bias | | | The study is judged to be at high risk of bias in at least one domain for this result. |

| **Igu (54)** | | | | |
|---|---|---|---|---|
| Bias domain and signalling question* | Response options | | | Comments |
| | Lower risk of bias | Higher risk of bias | Other | |
| **Bias arising from the randomisation process** | | | | |
| 1.1 Was the allocation sequence random? | Y/PY | N/PN | NI | "Sequence allocation software was used for the allocation of the participants into groups (participants were asked to pick 1 envelope containing pressure-sensitive paper labeled with either GROW-M or CG from a container). |
| 1.2 Was the allocation sequence concealed until participants were enrolled and assigned to interventions? | Y/PY | N/PN | NI | Randomization information was concealed from the participants until the assignment of the intervention." |
| 1.3 Did baseline differences between intervention groups suggest a problem with the randomisation process? | N/PN | Y/PY | NI | No, see explanation in section "Results" |

| | | | | |
|---|---|---|---|---|
| Risk-of-bias judgment (low/high/some concerns) | Low risk | | | For judgement we followed the suggested algorithm process:<br>1.2: Y/PY → 1.1: Y/PY → 1.3: N/PN → Low risk |
| **Bias due to deviations from intended interventions** | | | | |
| 2.1 Were participants aware of their assigned intervention during the trial? | N/PN | Y/PY | NI | Due to the nature of the waitlist control group it can be assumed that participants were aware of the assigned intervention. |
| 2.2 Were carers and people delivering the interventions aware of participants' assigned intervention during the trial? | N/PN | Y/PY | NI | No information. |
| 2.3 If Y/PY/NI to 2.1 or 2.2: Were there deviations from the intended intervention that arose because of the trial context? | N/PN | Y/PY | NA/NI | No information on deviations |
| 2.4 If Y/PY/NI to 2.3: Were these deviations likely to have affected the outcome? | N/PN | Y/PY | NA/NI | No information |
| 2.5 If Y/PY to 2.4: Were these deviations from intended intervention balanced between groups? | Y/PY | N/PN | NA/NI | NA |
| 2.6 Was an appropriate analysis used to estimate the effect of assignment to intervention? | Y/PY | N/PN | NI | ITT Analysis |
| 2.7 If N/PN/NI to 2.6: Was there potential for a substantial impact (on the result) of the failure to analyse participants in the group to which they were randomised? | N/PN | Y/PY | NA/NI | NA |
| Risk-of-bias judgment (low/high/some concerns) | Some concerns | | | For judgement we followed the suggested algorithm process:<br>Questions 2.1 to 2.5:2.1: Y/PY / 2.2: NI → 2.3: NI → Some concerns<br>Questions 2.6 & 2.7: 2.6: Y/PY → Low risk<br>Part 1 & Part 2: Some concerns in Part 2 → Some concerns |
| **Bias due to missing outcome data** | | | | |

| | | | | |
|---|---|---|---|---|
| 3.1 Were data for this outcome available for all, or nearly all, participants randomised? | Y/PY | N/PN | NI | IG: Lost to follow-up (n=0)<br>CG: Lost to follow-up (n=2) / Lost to compensatory intervention (n=1) |
| 3.2 If N/PN/NI to 3.1: Is there evidence that the result was not biased by missing outcome data? | Y/PY | N/PN | NA | NA |
| 3.3 If N/PN to 3.2: Could missingness in the outcome depend on its true value? | N/PN | Y/PY | NA/NI | NA |
| 3.4 If Y/PY/NI to 3.3: Is it likely that missingness in the outcome depended on its true value? | N/PN | Y/PY | NA/NI | NA |
| Risk-of-bias judgment (low/high/some concerns) | Low risk of bias | | | For judgement we followed the suggested algorithm process:<br>3.1: Y/PY → Low risk |
| **Bias in measurement of the outcome** | | | | |
| 4.1 Was the method of measuring the outcome inappropriate? | N/PN | Y/PY | NI | Appropriate measurements. |
| 4.2 Could measurement or ascertainment of the outcome have differed between intervention groups? | N/PN | Y/PY | NI | Same measurements/ascertainments of the outcome |
| 4.3 If N/PN/NI to 4.1 and 4.2: Were outcome assessors aware of the intervention received by study participants? | N/PN | Y/PY | NI | Participant-reported outcome |
| 4.4 If Y/PY/NI to 4.3: Could assessment of the outcome have been influenced by knowledge of intervention received? | N/PN | Y/PY | NA/NI | Assessment of the outcome could have been influenced |
| 4.5 If Y/PY/NI to 4.4: Is it likely that assessment of the outcome was influenced by knowledge of intervention received? | N/PN | Y/PY | NA/NI | "Another limitation is that of using selfreported measures, which could stir up a state of cognitive dissonance leading to a false elevation of the posttreatment rating of the treatment group. Further, the use of waitlisted CG may also have contributed to the cognitive dissonance as it may have given room for further cognitive dissonance through interpersonal interactions between the treatment and CGs." |

| | | | | |
|---|---|---|---|---|
| Risk-of-bias judgment (low/high/some concerns) | High risk of bias | | | For judgement we followed the suggested algorithm process:<br>4.1: N/PN → 4.2: N/PN → 4.3: Y/PY → 4.4: Y/PY → 4.5: Y/PY → High risk |
| **Bias in selection of the reported result** | | | | |
| 5.1 Were the data that produced this result analysed in accordance with a prespecified analysis plan that was finalised before unblinded outcome data were available for analysis? | Y/PY | N/PN | NI | Trail registry was performed retrospectively. (“This study is also part of a project registered retrospectively with the AEA RCT trial Registry with an identity number: “AEARCTR-0008614). However, it is unclear whether data was analyzed in accordance with a pre-specified analysis plan. |
| Is the numerical result being assessed likely to have been selected, on the basis of the results, from: | | | | |
| 5.2 ... multiple eligible outcome measurements (eg, scales, definitions, time points) within the outcome domain? | N/PN | Y/PY | NI | See 5.1. Analysis intentions are not available |
| 5.3 ... multiple eligible analyses of the data? | N/PN | Y/PY | NI | See 5.1. Analysis intentions are not available |
| Risk-of-bias judgment (low/high/some concerns) | Some concerns | | | For judgement we followed the suggested algorithm process:<br>5.2: NI / 5.3: NI → Some concerns |
| **Overall bias** | | | | |
| Risk-of-bias judgment (low/high/some concerns) | High risk of bias | | | The study is judged to be at high risk of bias in at least one domain for this result. |

| **Janka (56)** | | | | |
|---|---|---|---|---|
| Bias domain and signalling question* | Response options | | | Comments |
| | Lower risk of bias | Higher risk of bias | Other | |
| **Bias arising from the randomisation process** | | | | |

| | | | | |
|---|---|---|---|---|
| 1.1 Was the allocation sequence random? | Y/PY | N/PN | NI | Only stated that the study is randomized. |
| 1.2 Was the allocation sequence concealed until participants were enrolled and assigned to interventions? | Y/PY | N/PN | NI | No clear information |
| 1.3 Did baseline differences between intervention groups suggest a problem with the randomisation process? | N/PN | Y/PY | NI | Table 2 |
| Risk-of-bias judgment (low/high/some concerns) | Some concerns | | | For judgement we followed the suggested algorithm process:<br>1.2: NI → 1.3: N/PN → Some concerns |
| **Bias due to deviations from intended interventions** | | | | |
| 2.1 Were participants aware of their assigned intervention during the trial? | N/PN | Y/PY | NI | "Those in the control group were informed about the possibility of participating in the training after completion of the study." |
| 2.2 Were carers and people delivering the interventions aware of participants' assigned intervention during the trial? | N/PN | Y/PY | NI | No information. |
| 2.3 If Y/PY/NI to 2.1 or 2.2: Were there deviations from the intended intervention that arose because of the trial context? | N/PN | Y/PY | NA/NI | No information on deviations |
| 2.4 If Y/PY/NI to 2.3: Were these deviations likely to have affected the outcome? | N/PN | Y/PY | NA/NI | No information |
| 2.5 If Y/PY to 2.4: Were these deviations from intended intervention balanced between groups? | Y/PY | N/PN | NA/NI | NA |
| 2.6 Was an appropriate analysis used to estimate the effect of assignment to intervention? | Y/PY | N/PN | NI | No information about ITT or PP Analysis. This is because no information about missing data or drop out is reported. |

| | | | | |
|---|---|---|---|---|
| 2.7 If N/PN/NI to 2.6: Was there potential for a substantial impact (on the result) of the failure to analyse participants in the group to which they were randomised? | N/PN | Y/PY | NA/NI | No information |
| Risk-of-bias judgment (low/high/some concerns) | High risk of bias | | | For judgement we followed the suggested algorithm process:<br>Questions 2.1 to 2.5: 2.1: Y/PY / 2.2: NI → 2.3: NI → Some concerns<br>Questions 2.6 & 2.7: 2.6: NI → 2.7: NI → High risk<br>Part 1 & Part 2: High risk in Part 2 → High risk |
| **Bias due to missing outcome data** | | | | |
| 3.1 Were data for this outcome available for all, or nearly all, participants randomised? | Y/PY | N/PN | NI | No information about missing data or drop-outs. |
| 3.2 If N/PN/NI to 3.1: Is there evidence that the result was not biased by missing outcome data? | Y/PY | N/PN | NA | No evidence |
| 3.3 If N/PN to 3.2: Could missingness in the outcome depend on its true value? | N/PN | Y/PY | NA/NI | No information |
| 3.4 If Y/PY/NI to 3.3: Is it likely that missingness in the outcome depended on its true value? | N/PN | Y/PY | NA/NI | No information |
| Risk-of-bias judgment (low/high/some concerns) | High risk of bias | | | For judgement we followed the suggested algorithm process:<br>3.1: NI → 3.2: N/PN → 3.3: NI → 3.4: NI → High risk |
| **Bias in measurement of the outcome** | | | | |
| 4.1 Was the method of measuring the outcome inappropriate? | N/PN | Y/PY | NI | Appropriate Measurements |
| 4.2 Could measurement or ascertainment of the outcome have differed between intervention groups? | N/PN | Y/PY | NI | Same measurements/ascertainments of the outcome |

| | | | | |
|---|---|---|---|---|
| 4.3 If N/PN/NI to 4.1 and 4.2: Were outcome assessors aware of the intervention received by study participants? | N/PN | Y/PY | NI | Participant-reported outcomes |
| 4.4 If Y/PY/NI to 4.3: Could assessment of the outcome have been influenced by knowledge of intervention received? | N/PN | Y/PY | NA/NI | Assessment of the outcome could have been influenced |
| 4.5 If Y/PY/NI to 4.4: Is it likely that assessment of the outcome was influenced by knowledge of intervention received? | N/PN | Y/PY | NA/NI | Knowledge of intervention status could have influenced outcome assessment. |
| Risk-of-bias judgment (low/high/some concerns) | High risk of bias | | | For judgement we followed the suggested algorithm process:<br>4.1: N/PN → 4.2: N/PN → 4.3: Y/PY → 4.4: Y/PY → 4.5: Y/PY |
| **Bias in selection of the reported result** | | | | |
| 5.1 Were the data that produced this result analysed in accordance with a prespecified analysis plan that was finalised before unblinded outcome data were available for analysis? | Y/PY | N/PN | NI | No study protocol available |
| Is the numerical result being assessed likely to have been selected, on the basis of the results, from: | | | | |
| 5.2 ... multiple eligible outcome measurements (eg, scales, definitions, time points) within the outcome domain? | N/PN | Y/PY | NI | See 5.1. Analysis intentions are not available. |
| 5.3 ... multiple eligible analyses of the data? | N/PN | Y/PY | NI | See 5.1. Analysis intentions are not available. |
| Risk-of-bias judgment (low/high/some concerns) | Some concerns | | | For judgement we followed the suggested algorithm process:<br>5.2: NI / 5.3: NI → Some concerns |
| **Overall bias** | | | | |
| Risk-of-bias judgment (low/high/some concerns) | High risk of bias | | | The study is judged to be at high risk of bias in at least one domain for this result. |

| **Li (63)** | | | | |
|---|---|---|---|---|
| Bias domain and signalling question* | Response options | | | Comments |
| | Lower risk of bias | Higher risk of bias | Other | |
| **Bias arising from the randomisation process** | | | | |
| 1.1 Was the allocation sequence random? | Y/PY | N/PN | NI | It is only reported that participants were randomly assigned to the intervention/control group. |
| 1.2 Was the allocation sequence concealed until participants were enrolled and assigned to interventions? | Y/PY | N/PN | NI | No explicit information on allocation sequence. |
| 1.3 Did baseline differences between intervention groups suggest a problem with the randomisation process? | N/PN | Y/PY | NI | Table 1 |
| Risk-of-bias judgment (low/high/some concerns) | Some concerns | | | For judgement we followed the suggested algorithm process:<br>1.2: NI → 1.3: N/PN → Some concerns |
| **Bias due to deviations from intended interventions** | | | | |
| 2.1 Were participants aware of their assigned intervention during the trial? | N/PN | Y/PY | NI | "Given the fact that both the intervention group and the wait-list control group received the SMI, the two groups were merged for the current analysis and termed "MAN-GO participants." In order to evaluate the long-term effectiveness of this SMI at work, an "unexposed" external control group was established post hoc by using data from the German Socioeconomic Panel (SOEP), offering a sample with comparable sociodemographic characteristics." |

| | | | | |
|---|---|---|---|---|
| 2.2 Were carers and people delivering the interventions aware of participants' assigned intervention during the trial? | N/PN | Y/PY | NI | No information |
| 2.3 If Y/PY/NI to 2.1 or 2.2: Were there deviations from the intended intervention that arose because of the trial context? | N/PN | Y/PY | NA/NI | No information on deviations |
| 2.4 If Y/PY/NI to 2.3: Were these deviations likely to have affected the outcome? | N/PN | Y/PY | NA/NI | NA |
| 2.5 If Y/PY to 2.4: Were these deviations from intended intervention balanced between groups? | Y/PY | N/PN | NA/NI | NA |
| 2.6 Was an appropriate analysis used to estimate the effect of assignment to intervention? | Y/PY | N/PN | NI | 9-year follow-up. Consequently, only participants, who reported data were included into the analysis. Therefore, it can be claimed as modified Intention to treat analysis |
| 2.7 If N/PN/NI to 2.6: Was there potential for a substantial impact (on the result) of the failure to analyse participants in the group to which they were randomised? | N/PN | Y/PY | NA/NI | NA |
| Risk-of-bias judgment (low/high/some concerns) | Some concerns | | | For judgement we followed the suggested algorithm process:<br>Questions 2.1 to 2.5: 2.1: Y/PY / 2.2: NI → 2.3: NI → Some concerns<br>Questions 2.6 & 2.7: 2.6: Y/PY → Low risk<br>Part 1 & Part 2: Some concerns in Part 1 → Some concerns |
| **Bias due to missing outcome data** | | | | |
| 3.1 Were data for this outcome available for all, or nearly all, participants randomised? | Y/PY | N/PN | NI | "Moreover, the considerable attrition rates (i.e., sample reduction in the intervention group from 174 to 94 and in the post hoc external control group from 264 to 94) during the follow-up deserved attention." |
| 3.2 If N/PN/NI to 3.1: Is there evidence that the result was not biased by missing outcome data? | Y/PY | N/PN | NA | "Additional drop-out analyses were conducted to check whether the two groups of initial participants and those who remained till study end differed in main characteristics; we nevertheless did not observe any |

| | | | | |
|---|---|---|---|---|
| | | | | significant differences between those who continued to participate in surveys and those who dropped out during the follow-up.” |
| 3.3 If N/PN to 3.2: Could missingness in the outcome depend on its true value? | N/PN | Y/PY | NA/NI | NA |
| 3.4 If Y/PY/NI to 3.3: Is it likely that missingness in the outcome depended on its true value? | N/PN | Y/PY | NA/NI | NA |
| Risk-of-bias judgment (low/high/some concerns) | Low risk of bias | | | For judgement we followed the suggested algorithm process:<br>3.1: N/PN → 3.2: Y/PY → Low risk |
| **Bias in measurement of the outcome** | | | | |
| 4.1 Was the method of measuring the outcome inappropriate? | N/PN | Y/PY | NI | Measurements appropriate |
| 4.2 Could measurement or ascertainment of the outcome have differed between intervention groups? | N/PN | Y/PY | NI | Passive control group from SOEP panel |
| 4.3 If N/PN/NI to 4.1 and 4.2: Were outcome assessors aware of the intervention received by study participants? | N/PN | Y/PY | NI | Participant reported outcomes. Due to passive control group, outcome assessors could not be aware of assigned intervention. However, IG was aware of assigned intervention. |
| 4.4 If Y/PY/NI to 4.3: Could assessment of the outcome have been influenced by knowledge of intervention received? | N/PN | Y/PY | NA/NI | Due to 9-year follow-up |
| 4.5 If Y/PY/NI to 4.4: Is it likely that assessment of the outcome was influenced by knowledge of intervention received? | N/PN | Y/PY | NA/NI | NA |
| Risk-of-bias judgment (low/high/some concerns) | High risk of bias | | | For judgement we followed the suggested algorithm process:<br>4.1: N/PN → 4.2: Y/PY → High risk |
| **Bias in selection of the reported result** | | | | |

| | | | | |
|---|---|---|---|---|
| 5.1 Were the data that produced this result analysed in accordance with a prespecified analysis plan that was finalised before unblinded outcome data were available for analysis? | Y/PY | N/PN | NI | No study protocol found |
| Is the numerical result being assessed likely to have been selected, on the basis of the results, from: | | | | |
| 5.2 ... multiple eligible outcome measurements (eg, scales, definitions, time points) within the outcome domain? | N/PN | Y/PY | NI | Analysis intentions are not available |
| 5.3 ... multiple eligible analyses of the data? | N/PN | Y/PY | NI | Analysis intentions are not available |
| Risk-of-bias judgment (low/high/some concerns) | Some concerns | | | For judgement we followed the suggested algorithm process:<br>5.2: NI / 5.3: NI → Some concerns |
| **Overall bias** | | | | |
| Risk-of-bias judgment (low/high/some concerns) | High risk of bias | | | The study is judged to be at high risk of bias in at least one domain for this result. |

| **Limm (68)** | | | | |
|---|---|---|---|---|
| Bias domain and signalling question* | Response options | | | Comments |
| | Lower risk of bias | Higher risk of bias | Other | |
| **Bias arising from the randomisation process** | | | | |
| 1.1 Was the allocation sequence random? | Y/PY | N/PN | NI | It is only reported that participants were randomly assigned to the intervention/control group. |

| | | | | |
|---|---|---|---|---|
| 1.2 Was the allocation sequence concealed until participants were enrolled and assigned to interventions? | Y/PY | N/PN | NI | No explicit information on allocation sequence. |
| 1.3 Did baseline differences between intervention groups suggest a problem with the randomisation process? | N/PN | Y/PY | NI | Table 1 |
| Risk-of-bias judgment (low/high/some concerns) | Low risk | | | For judgement we followed the suggested algorithm process:<br>1.2: NI → 1.1: NI → 1.3: N/PN → Low risk |
| **Bias due to deviations from intended interventions** | | | | |
| 2.1 Were participants aware of their assigned intervention during the trial? | N/PN | Y/PY | NI | Impossible to blind participants due to waitlist control group |
| 2.2 Were carers and people delivering the interventions aware of participants' assigned intervention during the trial? | N/PN | Y/PY | NI | No information |
| 2.3 If Y/PY/NI to 2.1 or 2.2: Were there deviations from the intended intervention that arose because of the trial context? | N/PN | Y/PY | NA/NI | No information on deviations. |
| 2.4 If Y/PY/NI to 2.3: Were these deviations likely to have affected the outcome? | N/PN | Y/PY | NA/NI | No information |
| 2.5 If Y/PY to 2.4: Were these deviations from intended intervention balanced between groups? | Y/PY | N/PN | NA/NI | NA |
| 2.6 Was an appropriate analysis used to estimate the effect of assignment to intervention? | Y/PY | N/PN | NI | ITT-Analysis |
| 2.7 If N/PN/NI to 2.6: Was there potential for a substantial impact (on the result) of the failure to analyse participants in the group to which they were randomised? | N/PN | Y/PY | NA/NI | NA |

| | | | | |
|---|---|---|---|---|
| Risk-of-bias judgment (low/high/some concerns) | Some concerns | | | For judgement we followed the suggested algorithm process:<br>Questions 2.1 to 2.5: 2.1: Y/PY / 2.2: NI → 2.3: NI → Some concerns<br>Questions 2.6 & 2.7: 2.6: Y/PY → Low risk<br>Part 1 & Part 2: |
| **Bias due to missing outcome data** | | | | |
| 3.1 Were data for this outcome available for all, or nearly all, participants randomised? | Y/PY | N/PN | NI | IG: Lost to follow-up 13%<br>CG: Lost to follow up 9% |
| 3.2 If N/PN/NI to 3.1: Is there evidence that the result was not biased by missing outcome data? | Y/PY | N/PN | NA | NA |
| 3.3 If N/PN to 3.2: Could missingness in the outcome depend on its true value? | N/PN | Y/PY | NA/NI | NA |
| 3.4 If Y/PY/NI to 3.3: Is it likely that missingness in the outcome depended on its true value? | N/PN | Y/PY | NA/NI | NA |
| Risk-of-bias judgment (low/high/some concerns) | Low risk of bias | | | For judgement we followed the suggested algorithm process:<br>3.1: Y/PY → Low risk |
| **Bias in measurement of the outcome** | | | | |
| 4.1 Was the method of measuring the outcome inappropriate? | N/PN | Y/PY | NI | Measurements appropriate |
| 4.2 Could measurement or ascertainment of the outcome have differed between intervention groups? | N/PN | Y/PY | NI | Same measurements/ascertainments of the outcome |
| 4.3 If N/PN/NI to 4.1 and 4.2: Were outcome assessors aware of the intervention received by study participants? | N/PN | Y/PY | NI | Participant reported outcomes |

| | | | | |
|---|---|---|---|---|
| 4.4 If Y/PY/NI to 4.3: Could assessment of the outcome have been influenced by knowledge of intervention received? | N/PN | Y/PY | NA/NI | Assessment of the outcome could have been influenced |
| 4.5 If Y/PY/NI to 4.4: Is it likely that assessment of the outcome was influenced by knowledge of intervention received? | N/PN | Y/PY | NA/NI | knowledge of intervention status could have influenced outcome assessment but there is no reason to believe that it did due to one-year follow-up |
| Risk-of-bias judgment (low/high/some concerns) | Some concerns | | | For judgement we followed the suggested algorithm process:<br>4.1: N/PN → 4.2: N/PN → 4.3: Y/PY → 4.4: Y/PY → 4.5: N/PN → Some concerns |
| **Bias in selection of the reported result** | | | | |
| 5.1 Were the data that produced this result analysed in accordance with a prespecified analysis plan that was finalised before unblinded outcome data were available for analysis? | Y/PY | N/PN | NI | No study protocol found. |
| Is the numerical result being assessed likely to have been selected, on the basis of the results, from: | | | | |
| 5.2 ... multiple eligible outcome measurements (eg, scales, definitions, time points) within the outcome domain? | N/PN | Y/PY | NI | See 5.1. Analysis intentions are not available. |
| 5.3 ... multiple eligible analyses of the data? | N/PN | Y/PY | NI | See 5.1. Analysis intentions are not available. |
| Risk-of-bias judgment (low/high/some concerns) | Some concerns | | | For judgement we followed the suggested algorithm process:<br>5.2: NI / 5.3: NI → Some concerns |
| **Overall bias** | | | | |
| Risk-of-bias judgment (low/high/some concerns) | Some concerns | | | The study is judged to raise some concerns in at least one domain for this result, but not to be at high risk of bias for any domain. |

| **Ly (69)** | | | | |
|---|---|---|---|---|
| Bias domain and signalling question* | Response options | | | Comments |
| | Lower risk of bias | Higher risk of bias | Other | |
| **Bias arising from the randomisation process** | | | | |
| 1.1 Was the allocation sequence random? | Y/PY | N/PN | NI | Online randomization tool (www.random.org) |
| 1.2 Was the allocation sequence concealed until participants were enrolled and assigned to interventions? | Y/PY | N/PN | NI | Randomization via online tool and independent person, who was separate from the study personnel. |
| 1.3 Did baseline differences between intervention groups suggest a problem with the randomisation process? | N/PN | Y/PY | NI | Table 1 & description Chapter 3 |
| Risk-of-bias judgment (low/high/some concerns) | Low risk of bias | | | For judgement we followed the suggested algorithm process:<br>1.2: Y/PY → 1.1: Y/PY → 1.3: N/PN → Low risk |
| **Bias due to deviations from intended interventions** | | | | |
| 2.1 Were participants aware of their assigned intervention during the trial? | N/PN | Y/PY | NI | Waitlist control group. Therefore, impossible to blind participants. |
| 2.2 Were carers and people delivering the interventions aware of participants' assigned intervention during the trial? | N/PN | Y/PY | NI | Independent person, who performed randomization. Therefore, it is unlikely that carers and people delivering the intervention knew about intervention status. |
| 2.3 If Y/PY/NI to 2.1 or 2.2: Were there deviations from the intended intervention that arose because of the trial context? | N/PN | Y/PY | NA/NI | No information on deviations |
| 2.4 If Y/PY/NI to 2.3: Were these deviations likely to have affected the outcome? | N/PN | Y/PY | NA/NI | No information |

| | | | | |
|---|---|---|---|---|
| 2.5 If Y/PY to 2.4: Were these deviations from intended intervention balanced between groups? | Y/PY | N/PN | NA/NI | NA |
| 2.6 Was an appropriate analysis used to estimate the effect of assignment to intervention? | Y/PY | N/PN | NI | ITT Analysis |
| 2.7 If N/PN/NI to 2.6: Was there potential for a substantial impact (on the result) of the failure to analyse participants in the group to which they were randomised? | N/PN | Y/PY | NA/NI | NA |
| Risk-of-bias judgment (low/high/some concerns) | Some concerns | | | For judgement we followed the suggested algorithm process:<br>Questions 2.1 to 2.5: 2.1: Y/PY / 2.2: N/PN → 2.3: NI → Some concerns<br>Questions 2.6 & 2.7: 2.6: Y/PY → Low risk<br>Part 1 & Part 2: Some concerns in Part 2 → Some concerns |
| **Bias due to missing outcome data** | | | | |
| 3.1 Were data for this outcome available for all, or nearly all, participants randomised? | Y/PY | N/PN | NI | "Of the 74 participants randomized, one participant decided not to participate in the study. Five out of the 73 participants (6.8%) did not provide post-treatment data with a distribution of three participants from the intervention group and two participants from the control group." |
| 3.2 If N/PN/NI to 3.1: Is there evidence that the result was not biased by missing outcome data? | Y/PY | N/PN | NA | NA |
| 3.3 If N/PN to 3.2: Could missingness in the outcome depend on its true value? | N/PN | Y/PY | NA/NI | NA |
| 3.4 If Y/PY/NI to 3.3: Is it likely that missingness in the outcome depended on its true value? | N/PN | Y/PY | NA/NI | NA |
| Risk-of-bias judgment (low/high/some concerns) | Low risk of bias | | | For judgement we followed the suggested algorithm process:<br>3.1: Y/PY → Low risk |
| **Bias in measurement of the outcome** | | | | |

| | | | | |
|---|---|---|---|---|
| 4.1 Was the method of measuring the outcome inappropriate? | N/PN | Y/PY | NI | Measurments appropriate |
| 4.2 Could measurement or ascertainment of the outcome have differed between intervention groups? | N/PN | Y/PY | NI | Same measurements/ascertainments of the outcome |
| 4.3 If N/PN/NI to 4.1 and 4.2: Were outcome assessors aware of the intervention received by study participants? | N/PN | Y/PY | NI | Participant reported outcomes |
| 4.4 If Y/PY/NI to 4.3: Could assessment of the outcome have been influenced by knowledge of intervention received? | N/PN | Y/PY | NA/NI | Assessment of the outcome could have been influenced |
| 4.5 If Y/PY/NI to 4.4: Is it likely that assessment of the outcome was influenced by knowledge of intervention received? | N/PN | Y/PY | NA/NI | Knowledge of intervention status could have influenced outcome assessment but there is no reason to believe that it did |
| Risk-of-bias judgment (low/high/some concerns) | Some concerns | | | For judgement we followed the suggested algorithm process:<br>4.1: N/PN → 4.2: N/PN → 4.3: Y/PY → 4.4: Y/PY → 4.5: N/PN → Some concerns |
| **Bias in selection of the reported result** | | | | |
| 5.1 Were the data that produced this result analysed in accordance with a prespecified analysis plan that was finalised before unblinded outcome data were available for analysis? | Y/PY | N/PN | NI | No study protocol available |
| Is the numerical result being assessed likely to have been selected, on the basis of the results, from: | | | | |
| 5.2 ... multiple eligible outcome measurements (eg, scales, definitions, time points) within the outcome domain? | N/PN | Y/PY | NI | See 5.1. Analysis intentions are not available |
| 5.3 ... multiple eligible analyses of the data? | N/PN | Y/PY | NI | See 5.1. Analysis intentions are not available |

| | | |
|---|---|---|
| Risk-of-bias judgment (low/high/some concerns) | Some concerns | For judgement we followed the suggested algorithm process:<br>5.2: NI / 5.3: NI → Some concerns |
| **Overall bias** | | |
| Risk-of-bias judgment (low/high/some concerns) | Some concerns | The study is judged to raise some concerns in at least one domain for this result, but not to be at high risk of bias for any domain. |

| **Martin (72)** | | | | |
|---|---|---|---|---|
| Bias domain and signalling question* | Response options | | | Comments |
| | Lower risk of bias | Higher risk of bias | Other | |
| **Bias arising from the randomisation process** | | | | |
| 1.1 Was the allocation sequence random? | Y/PY | N/PN | NI | Random number generation |
| 1.2 Was the allocation sequence concealed until participants were enrolled and assigned to interventions? | Y/PY | N/PN | NI | "Participants were therefore not blind to intervention allocation." |
| 1.3 Did baseline differences between intervention groups suggest a problem with the randomisation process? | N/PN | Y/PY | NI | Table 1 |
| Risk-of-bias judgment (low/high/some concerns) | High risk of bias | | | For judgement we followed the suggested algorithm process:<br>1.2: N/PN → High risk |
| **Bias due to deviations from intended interventions** | | | | |
| 2.1 Were participants aware of their assigned intervention during the trial? | N/PN | Y/PY | NI | "Participants were advised via email that they would be mailed the intervention materials, and for the telephone-supported group, that they would be contacted by telephone." |

| | | | | |
|---|---|---|---|---|
| 2.2 Were carers and people delivering the interventions aware of participants' assigned intervention during the trial? | N/PN | Y/PY | NI | "Data collection and management activities were co-ordinated through the trial management database, including survey administration and issuing email templates set up for different stages of the trial (welcome emails, links to the on-line surveys, or manual processes associated with participants who elected to use hard copy mail surveys)." |
| 2.3 If Y/PY/NI to 2.1 or 2.2: Were there deviations from the intended intervention that arose because of the trial context? | N/PN | Y/PY | NA/NI | No deviations from the intended intervention |
| 2.4 If Y/PY/NI to 2.3: Were these deviations likely to have affected the outcome? | N/PN | Y/PY | NA/NI | NA |
| 2.5 If Y/PY to 2.4: Were these deviations from intended intervention balanced between groups? | Y/PY | N/PN | NA/NI | NA |
| 2.6 Was an appropriate analysis used to estimate the effect of assignment to intervention? | Y/PY | N/PN | NI | ITT Analysis |
| 2.7 If N/PN/NI to 2.6: Was there potential for a substantial impact (on the result) of the failure to analyse participants in the group to which they were randomised? | N/PN | Y/PY | NA/NI | NA |
| Risk-of-bias judgment (low/high/some concerns) | Low risk if bias | | | For judgement we followed the suggested algorithm process:<br>Questions 2.1 to 2.5: 2.1: Y/PY /2.2: Y/PY → 2.3: N/PN → Low risk<br>Questions 2.6 & 2.7: 2.6: Y/PY → Low risk<br>Part 1 & Part 2: Low risk in Part 1 and 2 → Low risk |
| **Bias due to missing outcome data** | | | | |
| 3.1 Were data for this outcome available for all, or nearly all, participants randomised? | Y/PY | N/PN | NI | „A total of 147 respondents completed the post-intervention survey, producing a response rate of 49.5%." |
| 3.2 If N/PN/NI to 3.1: Is there evidence that the result was not biased by missing outcome data? | Y/PY | N/PN | NA | Sensitivity analysis was performed, showing that results were not biased by missing outcome. |

| | | | | |
|---|---|---|---|---|
| 3.3 If N/PN to 3.2: Could missingness in the outcome depend on its true value? | N/PN | Y/PY | NA/NI | NA |
| 3.4 If Y/PY/NI to 3.3: Is it likely that missingness in the outcome depended on its true value? | N/PN | Y/PY | NA/NI | NA |
| Risk-of-bias judgment (low/high/some concerns) | Low risk of bias | | | For judgement we followed the suggested algorithm process:<br>3.1: N/PN → 3.2: Y/PY → Low risk |
| **Bias in measurement of the outcome** | | | | |
| 4.1 Was the method of measuring the outcome inappropriate? | N/PN | Y/PY | NI | No information |
| 4.2 Could measurement or ascertainment of the outcome have differed between intervention groups? | N/PN | Y/PY | NI | Same measurements/ascertainments of the outcome |
| 4.3 If N/PN/NI to 4.1 and 4.2: Were outcome assessors aware of the intervention received by study participants? | N/PN | Y/PY | NI | Participant reported outcomes |
| 4.4 If Y/PY/NI to 4.3: Could assessment of the outcome have been influenced by knowledge of intervention received? | N/PN | Y/PY | NA/NI | Assessment of the outcome could have been influenced |
| 4.5 If Y/PY/NI to 4.4: Is it likely that assessment of the outcome was influenced by knowledge of intervention received? | N/PN | Y/PY | NA/NI | Knowledge of intervention status could have influenced outcome assessment but there is no reason to believe that it did due to four month follow-up. |
| Risk-of-bias judgment (low/high/some concerns) | Some concerns | | | For judgement we followed the suggested algorithm process:<br>4.1: NI → 4.2: N/PN → 4.3: Y/PY → 4.4: Y/PY → 4.5: N/PN → Some concerns |
| **Bias in selection of the reported result** | | | | |
| 5.1 Were the data that produced this result analysed in accordance with a prespecified analysis plan that | Y/PY | N/PN | NI | Registered trial number: ISRCTN 62853520<br>Primary outcome “occupational stress (Job Tension Index) not reported in study. Given there was such a large attrition rate, further follow up after |

| | | | | |
|---|---|---|---|---|
| was finalised before unblinded outcome data were available for analysis? | | | | 6 and 12 months did not produce sufficient data for analysis and is thus not reported." S. 509 |
| Is the numerical result being assessed likely to have been selected, on the basis of the results, from: | | | | |
| 5.2 ... multiple eligible outcome measurements (eg, scales, definitions, time points) within the outcome domain? | N/PN | Y/PY | NI | All eligible reported results for the outcome domain correspond. |
| 5.3 ... multiple eligible analyses of the data? | N/PN | Y/PY | NI | Analysis intentions are not available |
| Risk-of-bias judgment (low/high/some concerns) | Some concerns | | | For judgement we followed the suggested algorithm process:<br>5.2: N/PN / 5.3: NI → Some concerns |
| **Overall bias** | | | | |
| Risk-of-bias judgment (low/high/some concerns) | High risk of bias | | | The study is judged to be at high risk of bias in at least one domain for this result. |

| **Mellner (76)** | | | | |
|---|---|---|---|---|
| Bias domain and signalling question* | Response options | | | Comments |
| | Lower risk of bias | Higher risk of bias | Other | |
| **Bias arising from the randomisation process** | | | | |
| 1.1 Was the allocation sequence random? | Y/PY | N/PN | NI | "The randomizations were conducted by using an online tool for research studies: www.random.org. |
| 1.2 Was the allocation sequence concealed until participants were enrolled and assigned to interventions? | Y/PY | N/PN | NI | Randomization was performed by HR department. Therefore, it can be assumed that principal investigator had no knowledge about forthcoming allocation. However, in discussion it is written that "the participants attended an information meeting prior to the start of the intervention and |

| | | | | |
|---|---|---|---|---|
| | | | | also knew which group they had been allocated to before they filled out the baseline assessments." Consequently, allocation sequence is not concealed. |
| 1.3 Did baseline differences between intervention groups suggest a problem with the randomisation process? | N/PN | Y/PY | NI | Table 1 |
| Risk-of-bias judgment (low/high/some concerns) | High risk of bias | | | For judgement we followed the suggested algorithm process:<br>1.2: N/PN → High risk |
| **Bias due to deviations from intended interventions** | | | | |
| 2.1 Were participants aware of their assigned intervention during the trial? | N/PN | Y/PY | NI | Waitlist control group and also see 2.1 |
| 2.2 Were carers and people delivering the interventions aware of participants' assigned intervention during the trial? | N/PN | Y/PY | NI | "The PI and all researchers/co-authors of the present study were blinded to the group allocation." |
| 2.3 If Y/PY/NI to 2.1 or 2.2: Were there deviations from the intended intervention that arose because of the trial context? | N/PN | Y/PY | NA/NI | No information |
| 2.4 If Y/PY/NI to 2.3: Were these deviations likely to have affected the outcome? | N/PN | Y/PY | NA/NI | |
| 2.5 If Y/PY to 2.4: Were these deviations from intended intervention balanced between groups? | Y/PY | N/PN | NA/NI | |
| 2.6 Was an appropriate analysis used to estimate the effect of assignment to intervention? | Y/PY | N/PN | NI | Intention to treat analysis |
| 2.7 If N/PN/NI to 2.6: Was there potential for a substantial impact (on the result) of the failure to analyse participants in the group to which they were randomised? | N/PN | Y/PY | NA/NI | NA |

| | | | | |
|---|---|---|---|---|
| Risk-of-bias judgment (low/high/some concerns) | Some concerns | | | For judgement we followed the suggested algorithm process:<br>Questions 2.1 to 2.5: 2.1: Y/PY / 2.2: N/PN → 2.3: NI → Some concerns<br>Questions 2.6 & 2.7: 2.6: Y/PY → Low risk<br>Part 1 & Part 2: Some concerns in Part 2 → Some concerns |
| **Bias due to missing outcome data** | | | | |
| 3.1 Were data for this outcome available for all, or nearly all, participants randomised? | Y/PY | N/PN | NI | 10% attrition rate |
| 3.2 If N/PN/NI to 3.1: Is there evidence that the result was not biased by missing outcome data? | Y/PY | N/PN | NA | NA |
| 3.3 If N/PN to 3.2: Could missingness in the outcome depend on its true value? | N/PN | Y/PY | NA/NI | NA |
| 3.4 If Y/PY/NI to 3.3: Is it likely that missingness in the outcome depended on its true value? | N/PN | Y/PY | NA/NI | NA |
| Risk-of-bias judgment (low/high/some concerns) | Low risk of bias | | | For judgement we followed the suggested algorithm process:<br>3.1: Y/PY → Low risk |
| **Bias in measurement of the outcome** | | | | |
| 4.1 Was the method of measuring the outcome inappropriate? | N/PN | Y/PY | NI | Five Facet Mindfulness Questionnaire |
| 4.2 Could measurement or ascertainment of the outcome have differed between intervention groups? | N/PN | Y/PY | NI | Same measurements/ascertainments of the outcome |
| 4.3 If N/PN/NI to 4.1 and 4.2: Were outcome assessors aware of the intervention received by study participants? | N/PN | Y/PY | NI | Participant reported outcomes |

| | | | | |
|---|---|---|---|---|
| 4.4 If Y/PY/NI to 4.3: Could assessment of the outcome have been influenced by knowledge of intervention received? | N/PN | Y/PY | NA/NI | See information in 1.2. |
| 4.5 If Y/PY/NI to 4.4: Is it likely that assessment of the outcome was influenced by knowledge of intervention received? | N/PN | Y/PY | NA/NI | Yes, as it is also reported in the discussion of the manuscript (p.505/506). |
| Risk-of-bias judgment (low/high/some concerns) | High risk of bias | | | For judgement we followed the suggested algorithm process:<br>4.1: N/PN → 4.2: N/PN → 4.3: Y/PY → 4.4: Y/PY → 4.5; Y/PY → high risk |
| **Bias in selection of the reported result** | | | | |
| 5.1 Were the data that produced this result analysed in accordance with a prespecified analysis plan that was finalised before unblinded outcome data were available for analysis? | Y/PY | N/PN | NI | No study protocol found |
| Is the numerical result being assessed likely to have been selected, on the basis of the results, from: | | | | |
| 5.2 ... multiple eligible outcome measurements (eg, scales, definitions, time points) within the outcome domain? | N/PN | Y/PY | NI | See 5.1. Analysis intentions are not available |
| 5.3 ... multiple eligible analyses of the data? | N/PN | Y/PY | NI | See 5.1. Analysis intentions are not available |
| Risk-of-bias judgment (low/high/some concerns) | Some concerns | | | For judgement we followed the suggested algorithm process:<br>5.2: NI / 5.3: NI → Some concerns |
| **Overall bias** | | | | |
| Risk-of-bias judgment (low/high/some concerns) | High risk of bias | | | The study is judged to be at high risk of bias in at least one domain for this result. |

| **Munafò (78)** | | | | |
|---|---|---|---|---|
| Bias domain and signalling question* | Response options | | | Comments |
| | Lower risk of bias | Higher risk of bias | Other | |
| **Bias arising from the randomisation process** | | | | |
| 1.1 Was the allocation sequence random? | Y/PY | N/PN | NI | The only information about randomization methods is a statement that the study is randomized |
| 1.2 Was the allocation sequence concealed until participants were enrolled and assigned to interventions? | Y/PY | N/PN | NI | No clear information. |
| 1.3 Did baseline differences between intervention groups suggest a problem with the randomisation process? | N/PN | Y/PY | NI | Table 1 |
| Risk-of-bias judgment (low/high/some concerns) | Some concerns | | | For judgement we followed the suggested algorithm process:<br>1.2: NI → 1.3: N/PN → Some concerns |
| **Bias due to deviations from intended interventions** | | | | |
| 2.1 Were participants aware of their assigned intervention during the trial? | N/PN | Y/PY | NI | No clear information. However, due to the type of intervention (IG: biofeedback training; EG: stress diary) it can be assumed that participants new about assigned intervention |
| 2.2 Were carers and people delivering the interventions aware of participants' assigned intervention during the trial? | N/PN | Y/PY | NI | "Self-report questionnaires for anxiety symptoms and health status were administered individually at pre- and post-training by a trained psychologist blind to the patient's group assignment (RSA-BF or control group)." |
| 2.3 If Y/PY/NI to 2.1 or 2.2: Were there deviations from the intended intervention that arose because of the trial context? | N/PN | Y/PY | NA/NI | No deviations from the intended intervention |

| | | | | |
|---|---|---|---|---|
| 2.4 If Y/PY/NI to 2.3: Were these deviations likely to have affected the outcome? | N/PN | Y/PY | NA/NI | NA |
| 2.5 If Y/PY to 2.4: Were these deviations from intended intervention balanced between groups? | Y/PY | N/PN | NA/NI | NA |
| 2.6 Was an appropriate analysis used to estimate the effect of assignment to intervention? | Y/PY | N/PN | NI | Per protocol analysis |
| 2.7 If N/PN/NI to 2.6: Was there potential for a substantial impact (on the result) of the failure to analyse participants in the group to which they were randomised? | N/PN | Y/PY | NA/NI | Drop-out (n=9) due to their work schedule. |
| Risk-of-bias judgment (low/high/some concerns) | Some concerns | | | For judgement we followed the suggested algorithm process:<br>Questions 2.1 to 2.5: 2.1: Y/PY / 2.2: N/PN → 2.3: N/PN → low risk<br>Questions 2.6 & 2.7: 2.6: N/PN → 2.7: N/PN → Some concerns<br>Part 1 & Part 2: Some concerns in Part 2 → Some concerns |
| **Bias due to missing outcome data** | | | | |
| 3.1 Were data for this outcome available for all, or nearly all, participants randomised? | Y/PY | N/PN | NI | IG: Drop-out 32%<br>CG: Drop-out 30% |
| 3.2 If N/PN/NI to 3.1: Is there evidence that the result was not biased by missing outcome data? | Y/PY | N/PN | NA | No information on sensitivity analyses or correction of missing outcome data. |
| 3.3 If N/PN to 3.2: Could missingness in the outcome depend on its true value? | N/PN | Y/PY | NA/NI | Reasons for drop-out were that participants "were unable to complete training because of their work schedule." |
| 3.4 If Y/PY/NI to 3.3: Is it likely that missingness in the outcome depended on its true value? | N/PN | Y/PY | NA/NI | NA |
| Risk-of-bias judgment (low/high/some concerns) | Low risk of bias | | | For judgement we followed the suggested algorithm process:<br>3.1: N/PN → 3.2: N/PN → 3.3: N/PN → Low risk |

| | | | | |
|---|---|---|---|---|
| **Bias in measurement of the outcome** | | | | |
| 4.1 Was the method of measuring the outcome inappropriate? | N/PN | Y/PY | NI | |
| 4.2 Could measurement or ascertainment of the outcome have differed between intervention groups? | N/PN | Y/PY | NI | No information |
| 4.3 If N/PN/NI to 4.1 and 4.2: Were outcome assessors aware of the intervention received by study participants? | N/PN | Y/PY | NI | Participant reported outcomes |
| 4.4 If Y/PY/NI to 4.3: Could assessment of the outcome have been influenced by knowledge of intervention received? | N/PN | Y/PY | NA/NI | Probably no, as CG received an alternative intervention |
| 4.5 If Y/PY/NI to 4.4: Is it likely that assessment of the outcome was influenced by knowledge of intervention received? | N/PN | Y/PY | NA/NI | NA |
| Risk-of-bias judgment (low/high/some concerns) | Some concerns | | | For judgement we followed the suggested algorithm process:<br>4.1: N/PN → 4.2: NI → 4.3: Y/PY → 4.4: N/PN → Some concerns |
| **Bias in selection of the reported result** | | | | |
| 5.1 Were the data that produced this result analysed in accordance with a prespecified analysis plan that was finalised before unblinded outcome data were available for analysis? | Y/PY | N/PN | NI | No study protocol found |
| Is the numerical result being assessed likely to have been selected, on the basis of the results, from: | | | | |
| 5.2 ... multiple eligible outcome measurements (eg, scales, definitions, time points) within the outcome domain? | N/PN | Y/PY | NI | See 5.1 |
| 5.3 ... multiple eligible analyses of the data? | N/PN | Y/PY | NI | See 5.1 |

| | | |
|---|---|---|
| Risk-of-bias judgment (low/high/some concerns) | Some concerns | For judgement we followed the suggested algorithm process:<br>5.2: NI / 5.3: NI → Some concerns |
| **Overall bias** | | |
| Risk-of-bias judgment (low/high/some concerns) | Some concerns | The study is judged to raise some concerns in at least one domain for this result, but not to be at high risk of bias for any domain. |

| **Nübold (82)** | | | | |
|---|---|---|---|---|
| Bias domain and signalling question* | Response options | | | Comments |
| | Lower risk of bias | Higher risk of bias | Other | |
| **Bias arising from the randomisation process** | | | | |
| 1.1 Was the allocation sequence random? | Y/PY | N/PN | NI | The only information about randomization methods is that participants were randomly assigned. |
| 1.2 Was the allocation sequence concealed until participants were enrolled and assigned to interventions? | Y/PY | N/PN | NI | No clear information. |
| 1.3 Did baseline differences between intervention groups suggest a problem with the randomisation process? | N/PN | Y/PY | NI | No information |
| Risk-of-bias judgment (low/high/some concerns) | Some concerns | | | For judgement we followed the suggested algorithm process:<br>1.2: NI → 1.3: NI → Some concerns |
| **Bias due to deviations from intended interventions** | | | | |
| 2.1 Were participants aware of their assigned intervention during the trial? | N/PN | Y/PY | NI | Due to the nature of their study design. |

| | | | | |
|---|---|---|---|---|
| 2.2 Were carers and people delivering the interventions aware of participants' assigned intervention during the trial? | N/PN | Y/PY | NI | App-based intervention |
| 2.3 If Y/PY/NI to 2.1 or 2.2: Were there deviations from the intended intervention that arose because of the trial context? | N/PN | Y/PY | NA/NI | No information |
| 2.4 If Y/PY/NI to 2.3: Were these deviations likely to have affected the outcome? | N/PN | Y/PY | NA/NI | No information |
| 2.5 If Y/PY to 2.4: Were these deviations from intended intervention balanced between groups? | Y/PY | N/PN | NA/NI | |
| 2.6 Was an appropriate analysis used to estimate the effect of assignment to intervention? | Y/PY | N/PN | NI | Intention to treat analysis. |
| 2.7 If N/PN/NI to 2.6: Was there potential for a substantial impact (on the result) of the failure to analyse participants in the group to which they were randomised? | N/PN | Y/PY | NA/NI | |
| Risk-of-bias judgment (low/high/some concerns) | Some concerns | | | For judgement we followed the suggested algorithm process:<br>Questions 2.1 to 2.5: 2.1: Y/PY / 2.2: N/PN → 2.3: NI → Some concerns<br>Questions 2.6 & 2.7: 2.6: Y/PY → Low risk<br>Part 1 & Part 2: Some concerns in Part 1 → Some concerns |
| **Bias due to missing outcome data** | | | | |
| 3.1 Were data for this outcome available for all, or nearly all, participants randomised? | Y/PY | N/PN | NI | IG: 93 (t1), 52 (t2)<br>CG: 80 (t1), 52 (t2) |
| 3.2 If N/PN/NI to 3.1: Is there evidence that the result was not biased by missing outcome data? | Y/PY | N/PN | NA | ITT approach was applied. |

| | | | | |
|---|---|---|---|---|
| 3.3 If N/PN to 3.2: Could missingness in the outcome depend on its true value? | N/PN | Y/PY | NA/NI | NA |
| 3.4 If Y/PY/NI to 3.3: Is it likely that missingness in the outcome depended on its true value? | N/PN | Y/PY | NA/NI | |
| Risk-of-bias judgment (low/high/some concerns) | Low risk of bias | | | For judgement we followed the suggested algorithm process:<br>3.1: N/PN → 3.2: Y/PY → Low risk |
| **Bias in measurement of the outcome** | | | | |
| 4.1 Was the method of measuring the outcome inappropriate? | N/PN | Y/PY | NI | |
| 4.2 Could measurement or ascertainment of the outcome have differed between intervention groups? | N/PN | Y/PY | NI | Same measurements/ascertainments of the outcome |
| 4.3 If N/PN/NI to 4.1 and 4.2: Were outcome assessors aware of the intervention received by study participants? | N/PN | Y/PY | NI | Participant and follower reported outcomes |
| 4.4 If Y/PY/NI to 4.3: Could assessment of the outcome have been influenced by knowledge of intervention received? | N/PN | Y/PY | NA/NI | Assessment of the outcome could have been influenced |
| 4.5 If Y/PY/NI to 4.4: Is it likely that assessment of the outcome was influenced by knowledge of intervention received? | N/PN | Y/PY | NA/NI | Knowledge of intervention status could have influenced outcome assessment |
| Risk-of-bias judgment (low/high/some concerns) | High risk of bias | | | For judgement we followed the suggested algorithm process:<br>4.1: N/PN → 4.2: N/PN → 4.3: Y/PY → 4.4: Y/PY → 4.5: Y/PY → High risk |
| **Bias in selection of the reported result** | | | | |
| 5.1 Were the data that produced this result analysed in accordance with a prespecified analysis plan that | Y/PY | N/PN | NI | No study protocol found |

| | | | | |
|---|---|---|---|---|
| was finalised before unblinded outcome data were available for analysis? | | | | |
| Is the numerical result being assessed likely to have been selected, on the basis of the results, from: | | | | |
| 5.2 ... multiple eligible outcome measurements (eg, scales, definitions, time points) within the outcome domain? | N/PN | Y/PY | NI | Analysis intentions are not available |
| 5.3 ... multiple eligible analyses of the data? | N/PN | Y/PY | NI | Analysis intentions are not available |
| Risk-of-bias judgment (low/high/some concerns) | Some concerns | | | For judgement we followed the suggested algorithm process:<br>5.2 / 5.3: NI → Some concerns |
| **Overall bias** | | | | |
| Risk-of-bias judgment (low/high/some concerns) | High risk of bias | | | The study is judged to be at high risk of bias in at least one domain for this result. |

| **Sawyer (85)** | | | | |
|---|---|---|---|---|
| Bias domain and signalling question* | Response options | | | Comments |
| | Lower risk of bias | Higher risk of bias | Other | |
| **Bias arising from the randomisation process** | | | | |
| 1.1 Was the allocation sequence random? | Y/PY | N/PN | NI | Randomization via computer-generated randomized number. |
| 1.2 Was the allocation sequence concealed until participants were enrolled and assigned to interventions? | Y/PY | N/PN | NI | “The randomization list was saved by the principal investigator in a password-protected file, and group assignments for each participant were shared with the recruitment team member after they were enrolled in the study.“ |

| | | | | |
|---|---|---|---|---|
| 1.3 Did baseline differences between intervention groups suggest a problem with the randomisation process? | N/PN | Y/PY | NI | Table 1 |
| Risk-of-bias judgment (low/high/some concerns) | Low risk of bias | | | For judgement we followed the suggested algorithm process:<br>1.2: Y/PY → 1.1: Y/PY → 1.3: N/PN → Low risk |
| **Bias due to deviations from intended interventions** | | | | |
| 2.1 Were participants aware of their assigned intervention during the trial? | N/PN | Y/PY | NI | Waitlist control group |
| 2.2 Were carers and people delivering the interventions aware of participants' assigned intervention during the trial? | N/PN | Y/PY | NI | Due to the described randomization process it can be assumed that outcome assessors were blinded. |
| 2.3 If Y/PY/NI to 2.1 or 2.2: Were there deviations from the intended intervention that arose because of the trial context? | N/PN | Y/PY | NA/NI | Trial was performed during Covid-19 pandemic. Therefore, the intervention was adapted. See section 2.3 Intervention for detailed description. |
| 2.4 If Y/PY/NI to 2.3: Were these deviations likely to have affected the outcome? | N/PN | Y/PY | NA/NI | |
| 2.5 If Y/PY to 2.4: Were these deviations from intended intervention balanced between groups? | Y/PY | N/PN | NA/NI | NA |
| 2.6 Was an appropriate analysis used to estimate the effect of assignment to intervention? | Y/PY | N/PN | NI | Per protocol analysis |
| 2.7 If N/PN/NI to 2.6: Was there potential for a substantial impact (on the result) of the failure to analyse participants in the group to which they were randomised? | N/PN | Y/PY | NA/NI | See Fig.1 for drop-out rates |
| Risk-of-bias judgment (low/high/some concerns) | High risk of bias | | | For judgement we followed the suggested algorithm process:<br>Questions 2.1 to 2.5: 2.1: Y/PY / 2.2: N/PN → 2.3: Y/PY → 2.4: N/PN → Some concerns |

| | | | | |
|---|---|---|---|---|
| | | | | Questions 2.6 & 2.7: 2.6: N/PN → 2.7: Y/PY → High risk<br>Part 1 & Part 2:High risk in Part 2 → High risk |
| **Bias due to missing outcome data** | | | | |
| 3.1 Were data for this outcome available for all, or nearly all, participants randomised? | Y/PY | N/PN | NI | IG: 20% drop-out rate between baseline and 6-month follow up: 20%<br>CG: 38% drop-out rate between baseline and 6-month follow up: 20% |
| 3.2 If N/PN/NI to 3.1: Is there evidence that the result was not biased by missing outcome data? | Y/PY | N/PN | NA | No information on sensitivity analyses or correction of missing outcome data. |
| 3.3 If N/PN to 3.2: Could missingness in the outcome depend on its true value? | N/PN | Y/PY | NA/NI | No information |
| 3.4 If Y/PY/NI to 3.3: Is it likely that missingness in the outcome depended on its true value? | N/PN | Y/PY | NA/NI | |
| Risk-of-bias judgment (low/high/some concerns) | Some concerns | | | For judgement we followed the suggested algorithm process:<br>3.1: N/PN → 3.2: N/PN → 3.3: NI → 3.4: N/PN → Some concerns |
| **Bias in measurement of the outcome** | | | | |
| 4.1 Was the method of measuring the outcome inappropriate? | N/PN | Y/PY | NI | No clear information |
| 4.2 Could measurement or ascertainment of the outcome have differed between intervention groups? | N/PN | Y/PY | NI | Same measurements/ascertainments of the outcome |
| 4.3 If N/PN/NI to 4.1 and 4.2: Were outcome assessors aware of the intervention received by study participants? | N/PN | Y/PY | NI | Participant reported outcomes |
| 4.4 If Y/PY/NI to 4.3: Could assessment of the outcome have been influenced by knowledge of intervention received? | N/PN | Y/PY | NA/NI | Assessment of the outcome could have been influenced |

| | | | | |
|---|---|---|---|---|
| 4.5 If Y/PY/NI to 4.4: Is it likely that assessment of the outcome was influenced by knowledge of intervention received? | N/PN | Y/PY | NA/NI | Knowledge of intervention status could have influenced outcome assessment but there is no reason to believe that it did |
| Risk-of-bias judgment (low/high/some concerns) | Some concerns | | | For judgement we followed the suggested algorithm process:<br>4.1: NI → 4.2: N/PN → 4.3: Y/PY → 4.4: Y/PY → N/PN → Some concerns |
| **Bias in selection of the reported result** | | | | |
| 5.1 Were the data that produced this result analysed in accordance with a prespecified analysis plan that was finalised before unblinded outcome data were available for analysis? | Y/PY | N/PN | NI | Registered on ClinicalTrials.gov (NCT05254600). Due to the Covid-Pandemic changes in the intervention and outcome assessments were performed: https://clinicaltrials.gov/study/NCT05254600?id=NCT05254600&rank=1 |
| Is the numerical result being assessed likely to have been selected, on the basis of the results, from: | | | | |
| 5.2 ... multiple eligible outcome measurements (eg, scales, definitions, time points) within the outcome domain? | N/PN | Y/PY | NI | See 5.1 for link of trial registration. All eligible reported results for the outcome domain correspond. |
| 5.3 ... multiple eligible analyses of the data? | N/PN | Y/PY | NI | No clear information. |
| Risk-of-bias judgment (low/high/some concerns) | Some concerns | | | For judgement we followed the suggested algorithm process:<br>5.2: N/PN / 5.3: NI → Some concerns |
| **Overall bias** | | | | |
| Risk-of-bias judgment (low/high/some concerns) | High risk of bias | | | The study is judged to be at high risk of bias in at least one domain for this result. |

| **Shonin (89)** | | |
|---|---|---|
| Bias domain and signalling question* | Response options | Comments |

| | Lower risk of bias | Higher risk of bias | Other | |
|---|---|---|---|---|
| **Bias arising from the randomisation process** | | | | |
| 1.1 Was the allocation sequence random? | Y/PY | N/PN | NI | “A list of eligible participant pseudonyms, grouped by sex, was then passed to the second author who conducted the randomisation procedure. On a sex-strata basis, participant pseudonyms were placed into a bowl, and then selected one at a time and inserted alternately into two separate envelopes.” |
| 1.2 Was the allocation sequence concealed until participants were enrolled and assigned to interventions? | Y/PY | N/PN | NI | “Randomisation occurred prior to participants completing any baseline assessments.” |
| 1.3 Did baseline differences between intervention groups suggest a problem with the randomisation process? | N/PN | Y/PY | NI | Table 1 and description page 813/814 |
| Risk-of-bias judgment (low/high/some concerns) | Low risk of bias | | | For judgement we followed the suggested algorithm process:<br>1.2: Y/PY → 1.1: Y/PY → 1.3: N/PN → Low riks |
| **Bias due to deviations from intended interventions** | | | | |
| 2.1 Were participants aware of their assigned intervention during the trial? | N/PN | Y/PY | NI | CG received alternative intervention, which was identical to the intervention condition. |
| 2.2 Were carers and people delivering the interventions aware of participants’ assigned intervention during the trial? | N/PN | Y/PY | NI | Detailed description of randomization and blinding process. However, no clear information on blinded outcome assessors. |
| 2.3 If Y/PY/NI to 2.1 or 2.2: Were there deviations from the intended intervention that arose because of the trial context? | N/PN | Y/PY | NA/NI | No deviations reported. |
| 2.4 If Y/PY/NI to 2.3: Were these deviations likely to have affected the outcome? | N/PN | Y/PY | NA/NI | NA |

| | | | | |
|---|---|---|---|---|
| 2.5 If Y/PY to 2.4: Were these deviations from intended intervention balanced between groups? | Y/PY | N/PN | NA/NI | NA |
| 2.6 Was an appropriate analysis used to estimate the effect of assignment to intervention? | Y/PY | N/PN | NI | ITT Analysis |
| 2.7 If N/PN/NI to 2.6: Was there potential for a substantial impact (on the result) of the failure to analyse participants in the group to which they were randomised? | N/PN | Y/PY | NA/NI | NA |
| Risk-of-bias judgment (low/high/some concerns) | Low risk of bias | | | For judgement we followed the suggested algorithm process:<br>Questions 2.1 to 2.5: 2.1: N/PN / 2.2: NI → 2.3: N/PN → Low risk<br>Questions 2.6 & 2.7: 2.6: Y/PY → Low risk<br>Part 1 & Part 2: Both low risk → Low risk |
| **Bias due to missing outcome data** | | | | |
| 3.1 Were data for this outcome available for all, or nearly all, participants randomised? | Y/PY | N/PN | NI | 90 % response rate |
| 3.2 If N/PN/NI to 3.1: Is there evidence that the result was not biased by missing outcome data? | Y/PY | N/PN | NA | NA |
| 3.3 If N/PN to 3.2: Could missingness in the outcome depend on its true value? | N/PN | Y/PY | NA/NI | NA |
| 3.4 If Y/PY/NI to 3.3: Is it likely that missingness in the outcome depended on its true value? | N/PN | Y/PY | NA/NI | NA |
| Risk-of-bias judgment (low/high/some concerns) | Low risk of bias | | | For judgement we followed the suggested algorithm process:<br>3.1: Y/PY → Low risk |
| **Bias in measurement of the outcome** | | | | |

| | | | | |
|---|---|---|---|---|
| 4.1 Was the method of measuring the outcome inappropriate? | N/PN | Y/PY | NI | See section "Outcome Measures" page 811 |
| 4.2 Could measurement or ascertainment of the outcome have differed between intervention groups? | N/PN | Y/PY | NI | Same measurements/ascertainments of the outcome |
| 4.3 If N/PN/NI to 4.1 and 4.2: Were outcome assessors aware of the intervention received by study participants? | N/PN | Y/PY | NI | Participant reported outcomes |
| 4.4 If Y/PY/NI to 4.3: Could assessment of the outcome have been influenced by knowledge of intervention received? | N/PN | Y/PY | NA/NI | Probably no as CG received alternative intervention. |
| 4.5 If Y/PY/NI to 4.4: Is it likely that assessment of the outcome was influenced by knowledge of intervention received? | N/PN | Y/PY | NA/NI | NA |
| Risk-of-bias judgment (low/high/some concerns) | Low risk of bias | | | For judgement we followed the suggested algorithm process:<br>4.1: N/PN → 4.2: N/PN → 4.3: Y/PY → 4.4: N/PN → Low risk |
| **Bias in selection of the reported result** | | | | |
| 5.1 Were the data that produced this result analysed in accordance with a prespecified analysis plan that was finalised before unblinded outcome data were available for analysis? | Y/PY | N/PN | NI | No study protocol found. |
| Is the numerical result being assessed likely to have been selected, on the basis of the results, from: | | | | |
| 5.2 ... multiple eligible outcome measurements (eg, scales, definitions, time points) within the outcome domain? | N/PN | Y/PY | NI | See 5.1 |
| 5.3 ... multiple eligible analyses of the data? | N/PN | Y/PY | NI | See 5.1 |
| Risk-of-bias judgment (low/high/some concerns) | Some concerns | | | For judgement we followed the suggested algorithm process: |

| | | |
|---|---|---|
| | | 5.2: NI / 5.3: NI → Some concerns |
| **Overall bias** | | |
| Risk-of-bias judgment (low/high/some concerns) | Some concerns | The study is judged to raise some concerns in at least one domain for this result, but not to be at high risk of bias for any domain. |

| **Zolnierczyk-Zreda (109)** | | | | |
|---|---|---|---|---|
| Bias domain and signalling question* | Response options | | | Comments |
| | Lower risk of bias | Higher risk of bias | Other | |
| **Bias arising from the randomisation process** | | | | |
| 1.1 Was the allocation sequence random? | Y/PY | N/PN | NI | "For randomization, sealed envelopes were prepared in advance, marked on the inside either '0' or '1'." |
| 1.2 Was the allocation sequence concealed until participants were enrolled and assigned to interventions? | Y/PY | N/PN | NI | "Randomization was performed prior to the start of the pre-test into experimental and control groups." |
| 1.3 Did baseline differences between intervention groups suggest a problem with the randomisation process? | N/PN | Y/PY | NI | Described on page 633, left column |
| Risk-of-bias judgment (low/high/some concerns) | High risk of bias | | | For judgement we followed the suggested algorithm process:<br>1.2: N/PN → High risk |
| **Bias due to deviations from intended interventions** | | | | |
| 2.1 Were participants aware of their assigned intervention during the trial? | N/PN | Y/PY | NI | Waitlist control group |

| | | | | |
|---|---|---|---|---|
| 2.2 Were carers and people delivering the interventions aware of participants' assigned intervention during the trial? | N/PN | Y/PY | NI | No information |
| 2.3 If Y/PY/NI to 2.1 or 2.2: Were there deviations from the intended intervention that arose because of the trial context? | N/PN | Y/PY | NA/NI | No information |
| 2.4 If Y/PY/NI to 2.3: Were these deviations likely to have affected the outcome? | N/PN | Y/PY | NA/NI | No information |
| 2.5 If Y/PY to 2.4: Were these deviations from intended intervention balanced between groups? | Y/PY | N/PN | NA/NI | No information |
| 2.6 Was an appropriate analysis used to estimate the effect of assignment to intervention? | Y/PY | N/PN | NI | ITT Analysis (excluding six persons (8% drop-out rate) |
| 2.7 If N/PN/NI to 2.6: Was there potential for a substantial impact (on the result) of the failure to analyse participants in the group to which they were randomised? | N/PN | Y/PY | NA/NI | NA |
| Risk-of-bias judgment (low/high/some concerns) | Some concerns | | | For judgement we followed the suggested algorithm process:<br>Questions 2.1 to 2.5:2.1: Y/PY / 2.2: NI → 2.3: NI → Some concerns<br>Questions 2.6 & 2.7: 2.6: Y/PY → low risk<br>Part 1 & Part 2: Some concerns in Part 2 → Some concerns |
| **Bias due to missing outcome data** | | | | |
| 3.1 Were data for this outcome available for all, or nearly all, participants randomised? | Y/PY | N/PN | NI | "Overall dropout from the study was relatively low (8%) and did not differ between the intervention and control groups." |
| 3.2 If N/PN/NI to 3.1: Is there evidence that the result was not biased by missing outcome data? | Y/PY | N/PN | NA | NA |

| | | | | |
|---|---|---|---|---|
| 3.3 If N/PN to 3.2: Could missingness in the outcome depend on its true value? | N/PN | Y/PY | NA/NI | NA |
| 3.4 If Y/PY/NI to 3.3: Is it likely that missingness in the outcome depended on its true value? | N/PN | Y/PY | NA/NI | NA |
| Risk-of-bias judgment (low/high/some concerns) | Low risk of bias | | | For judgement we followed the suggested algorithm process:<br>3.1: Y/PY → Low risk |
| **Bias in measurement of the outcome** | | | | |
| 4.1 Was the method of measuring the outcome inappropriate? | N/PN | Y/PY | NI | No clear information |
| 4.2 Could measurement or ascertainment of the outcome have differed between intervention groups? | N/PN | Y/PY | NI | Same measurements/ascertainments of the outcome |
| 4.3 If N/PN/NI to 4.1 and 4.2: Were outcome assessors aware of the intervention received by study participants? | N/PN | Y/PY | NI | Participant reported outcome |
| 4.4 If Y/PY/NI to 4.3: Could assessment of the outcome have been influenced by knowledge of intervention received? | N/PN | Y/PY | NA/NI | Assessment of the outcome could have been influenced. |
| 4.5 If Y/PY/NI to 4.4: Is it likely that assessment of the outcome was influenced by knowledge of intervention received? | N/PN | Y/PY | NA/NI | Knowledge of intervention status was unlikely to influence outcome assessment. |
| Risk-of-bias judgment (low/high/some concerns) | Some concerns | | | For judgement we followed the suggested algorithm process:<br>4.1: NI → 4.2: N/PN → 4.3: Y/PY → 4.4: Y/PY → 4.5: N/PN → Some concerns |
| **Bias in selection of the reported result** | | | | |
| 5.1 Were the data that produced this result analysed in accordance with a prespecified analysis plan that | Y/PY | N/PN | NI | No study protocol found. |

| | | | | |
|---|---|---|---|---|
| was finalised before unblinded outcome data were available for analysis? | | | | |
| Is the numerical result being assessed likely to have been selected, on the basis of the results, from: | | | | |
| 5.2 ... multiple eligible outcome measurements (eg, scales, definitions, time points) within the outcome domain? | N/PN | Y/PY | NI | See 5.1 |
| 5.3 ... multiple eligible analyses of the data? | N/PN | Y/PY | NI | See 5.1 |
| Risk-of-bias judgment (low/high/some concerns) | Some concerns | | | For judgement we followed the suggested algorithm process:<br>5.2: NI / 5.3: NI → Some concerns |
| **Overall bias** | | | | |
| Risk-of-bias judgment (low/high/some concerns) | High risk of bias | | | The study is judged to be at high risk of bias in at least one domain for this result. |

## 6.2 Controlled before–after studies

| **Allen (30)** | | | | |
|---|---|---|---|---|
| Bias domain and signalling question* | Response options | | | Comments |
| | Lower risk of bias | Higher risk of bias | Other | |
| **Risk of bias due to confounding** | | | | |
| 1.1 Is there potential for confounding of the effect of intervention in this study? | N/PN | Y/PY | / | |
| If N/PN to 1.1: the study can be considered to be at low risk of bias due to confounding and no further signalling questions need be considered | | | | |
| If Y/PY to 1.1: determine whether there is a need to assess time-varying confounding: | | | | |

| | | | | |
|---|---|---|---|---|
| 1.2. Was the analysis based on splitting participants’ follow up time according to intervention received? | N/PN | Y/PY | NA/NI | |
| If N/PN, answer questions relating to baseline confounding (1.4 to 1.6) | | | | |
| If Y/PY, go to question 1.3. | | | | |
| 1.3. Were intervention discontinuations or switches likely to be related to factors that are prognostic for the outcome? | N/PN | Y/PY | NA/NI | NA |
| If N/PN, answer questions relating to baseline confounding (1.4 to 1.6) | | | | |
| If Y/PY, answer questions relating to both baseline and time-varying confounding (1.7 and 1.8) | | | | |
| *Questions relating to baseline confounding only* | | | | |
| 1.4. Did the authors use an appropriate analysis method that controlled for all the important confounding domains? | Y/PY | N/PN | NA/NI | No information |
| 1.5. If Y/PY to 1.4: Were confounding domains that were controlled for measured validly and reliably by the variables available in this study? | Y/PY | N/PN | NA/NI | NA |
| 1.6. Did the authors control for any post-intervention variables that could have been affected by the intervention? | N/PN | Y/PY | NA/NI | “The Tukey B procedure was used for post hoc comparisons among appropriate pairs of means.” |
| *Questions relating to baseline and time-varying confounding* | | | | |
| 1.7. Did the authors use an appropriate analysis method that controlled for all the important confounding domains and for time-varying confounding? | Y/PY | N/PN | NA/NI | NA |

| | | | | |
|---|---|---|---|---|
| 1.8. If Y/PY to 1.7: Were confounding domains that were controlled for measured validly and reliably by the variables available in this study? | Y/PY | N/PN | NA/NI | NA |
| Risk-of-bias judgment (low/moderate/serious/critical/NI) | Serious risk of bias | | | |
| **Risk of bias in selection of participants into the study** | | | | |
| 2.1. Was selection of participants into the study (or into the analysis) based on participant characteristics observed after the start of intervention? | N/PN | Y/PY | NI | No clear information |
| If N/PN to 2.1: go to 2.4 | | | | |
| 2.2. If Y/PY to 2.1: Were the post-intervention variables that influenced selection likely to be associated with intervention? | N/PN | Y/PY | NA/NI | NA |
| 2.3 If Y/PY to 2.2: Were the post-intervention variables that influenced selection likely to be influenced by the outcome or a cause of the outcome? | N/PN | Y/PY | NA/NI | NA |
| 2.4. Do start of follow-up and start of intervention coincide for most participants? | Y/PY | N/PN | NI | No clear information. |
| 2.5. If Y/PY to 2.2 and 2.3, or N/PN to 2.4: Were adjustment techniques used that are likely to correct for the presence of selection biases? | Y/PY | N/PN | NA/NI | NA |
| Risk-of-bias judgment (low/moderate/serious/critical/NI) | Serious risk of bias | | | |
| **Risk of bias in classification of interventions** | | | | |
| 3.1 Were intervention groups clearly defined? | Y/PY | N/PN | NI | |
| 3.2 Was the information used to define intervention groups recorded at the start of the intervention? | Y/PY | N/PN | NI | |
| 3.3 Could classification of intervention status have been affected by knowledge of the outcome or risk of the outcome? | N/PN | Y/PY | NI | |
| Risk-of-bias judgment (low/moderate/serious/critical/NI) | Serious risk of bias | | | |
| **Risk of bias due to deviations from intended interventions** | | | | |

| | | | | |
|---|---|---|---|---|
| *If your aim for this study is to assess the effect of assignment to intervention, answer questions 4.1 and 4.2* | | | | |
| 4.1. Were there deviations from the intended intervention beyond what would be expected in usual practice? | N/PN | Y/PY | NI | Waitlist control group |
| 4.2. If Y/PY to 4.1: Were these deviations from intended intervention unbalanced between groups and likely to have affected the outcome? | N/PN | Y/PY | NA/NI | NA |
| *If your aim for this study is to assess the effect of starting and adhering to intervention, answer questions 4.3 to 4.6* | | | | NA |
| 4.3. Were important co-interventions balanced across intervention groups? | Y/PY | N/PN | NI | NA |
| 4.4. Was the intervention implemented successfully for most participants? | Y/PY | N/PN | NI | NA |
| 4.5. Did study participants adhere to the assigned intervention regimen? | Y/PY | N/PN | NI | NA |
| 4.6. If N/PN to 4.3, 4.4 or 4.5: Was an appropriate analysis used to estimate the effect of starting and adhering to the intervention? | Y/PY | N/PN | NA/NI | NA |
| Risk-of-bias judgment (low/moderate/serious/critical/NI) | Low risk of bias | | | |
| **Risk of bias due to missing data** | | | | |
| 5.1 Were outcome data available for all, or nearly all, participants? | Y/PY | N/PN | NI | No clear information. |
| 5.2 Were participants excluded due to missing data on intervention status? | N/PN | Y/PY | NI | No information |
| 5.3 Were participants excluded due to missing data on other variables needed for the analysis? | N/PN | Y/PY | NI | No information |
| 5.4 If PN/N to 5.1, or Y/PY to 5.2 or 5.3: Are the proportion of participants and reasons for missing data similar across interventions? | Y/PY | N/PN | NA/NI | NA |
| 5.5 If PN/N to 5.1, or Y/PY to 5.2 or 5.3: Is there evidence that results were robust to the presence of missing data? | Y/PY | N/PN | NA/NI | NA |

| | | | | |
|---|---|---|---|---|
| Risk-of-bias judgment (low/moderate/serious/critical/NI) | No information | | | |
| **Risk of bias in measurement of outcomes** | | | | |
| 6.1 Could the outcome measure have been influenced by knowledge of the intervention received? | N/PN | Y/PY | NI | |
| 6.2 Were outcome assessors aware of the intervention received by study participants? | N/PN | Y/PY | NI | Participant reported outcomes |
| 6.3 Were the methods of outcome assessment comparable across intervention groups? | Y/PY | N/PN | NI | |
| 6.4 Were any systematic errors in measurement of the outcome related to intervention received? | N/PN | Y/PY | NI | |
| Risk-of-bias judgment (low/moderate/serious/critical/NI) | Moderate risk of bias | | | |
| **Risk of bias in selection of the reported result** | | | | |
| Is the reported effect estimate likely to be selected, on the basis of the results, from... | | | | |
| 7.1. ... multiple outcome measurements within the outcome domain? | N/PN | Y/PY | NI | |
| 7.2 ... multiple analyses of the intervention-outcome relationship? | N/PN | Y/PY | NI | |
| 7.3 ... different subgroups? | N/PN | Y/PY | NI | Small sample size |
| Risk-of-bias judgment (low/moderate/serious/critical/NI) | Moderate risk of bias | | | |
| **Overall bias** | | | | |
| Risk-of-bias judgment (low/moderate/serious/critical/NI) | Serious risk of bias | | | The study is judged to be at serious risk of bias in at least one domain, but not at critical risk of bias in any domain. |

| **Cedstrand (39)** | | | | |
|---|---|---|---|---|
| Bias domain and signalling question* | Response options | | | Comments |
| | Lower risk of bias | Higher risk of bias | Other | |
| **Risk of bias due to confounding** | | | | |
| 1.1 Is there potential for confounding of the effect of intervention in this study? | N/PN | Y/PY | / | |
| If N/PN to 1.1: the study can be considered to be at low risk of bias due to confounding and no further signalling questions need be considered | | | | |
| If Y/PY to 1.1: determine whether there is a need to assess time-varying confounding: | | | | |
| 1.2. Was the analysis based on splitting participants' follow up time according to intervention received? | N/PN | Y/PY | NA/NI | |
| If N/PN, answer questions relating to baseline confounding (1.4 to 1.6) | | | | |
| If Y/PY, go to question 1.3. | | | | |
| 1.3. Were intervention discontinuations or switches likely to be related to factors that are prognostic for the outcome? | N/PN | Y/PY | NA/NI | NA |
| If N/PN, answer questions relating to baseline confounding (1.4 to 1.6) | | | | |
| If Y/PY, answer questions relating to both baseline and time-varying confounding (1.7 and 1.8) | | | | |
| *Questions relating to baseline confounding only* | | | | |
| 1.4. Did the authors use an appropriate analysis method that controlled for all the important confounding domains? | Y/PY | N/PN | NA/NI | p. 05, left column, up top. |

| | | | | |
|---|---|---|---|---|
| 1.5. If Y/PY to 1.4: Were confounding domains that were controlled for measured validly and reliably by the variables available in this study? | Y/PY | N/PN | NA/NI | No clear information. |
| 1.6. Did the authors control for any post-intervention variables that could have been affected by the intervention? | N/PN | Y/PY | NA/NI | No clear information. |
| *Questions relating to baseline and time-varying confounding* | | | | |
| 1.7. Did the authors use an appropriate analysis method that controlled for all the important confounding domains and for time-varying confounding? | Y/PY | N/PN | NA/NI | |
| 1.8. If Y/PY to 1.7: Were confounding domains that were controlled for measured validly and reliably by the variables available in this study? | Y/PY | N/PN | NA/NI | NA |
| Risk-of-bias judgment (low/moderate/serious/critical/NI) | Serious risk of bias | | | |
| **Risk of bias in selection of participants into the study** | | | | |
| 2.1. Was selection of participants into the study (or into the analysis) based on participant characteristics observed after the start of intervention? | N/PN | Y/PY | NI | “Two regions within a large Swedish construction company were recruited. Two large construction companies in Sweden were contacted, and after a few meetings, one of them agreed to |

| | | | | |
|---|---|---|---|---|
| | | | | participate in the study." |
| If N/PN to 2.1: go to 2.4 | | | | |
| 2.2. If Y/PY to 2.1: Were the post-intervention variables that influenced selection likely to be associated with intervention? | N/PN | Y/PY | NA/NI | |
| 2.3 If Y/PY to 2.2: Were the post-intervention variables that influenced selection likely to be influenced by the outcome or a cause of the outcome? | N/PN | Y/PY | NA/NI | |
| 2.4. Do start of follow-up and start of intervention coincide for most participants? | Y/PY | N/PN | NI | |
| 2.5. If Y/PY to 2.2 and 2.3, or N/PN to 2.4: Were adjustment techniques used that are likely to correct for the presence of selection biases? | Y/PY | N/PN | NA/NI | "Marginal means models adjusting for missing data patterns were applied to estimate potential differences in outcomes between groups over time." |
| Risk-of-bias judgment (low/moderate/serious/critical/NI) | Moderate risk of bias | | | |
| **Risk of bias in classification of interventions** | | | | |
| 3.1 Were intervention groups clearly defined? | Y/PY | N/PN | NI | |
| 3.2 Was the information used to define intervention groups recorded at the start of the intervention? | Y/PY | N/PN | NI | |

| | | | | |
|---|---|---|---|---|
| 3.3 Could classification of intervention status have been affected by knowledge of the outcome or risk of the outcome? | N/PN | Y/PY | NI | |
| Risk-of-bias judgment (low/moderate/serious/critical/NI) | Low risk of bias | | | |
| **Risk of bias due to deviations from intended interventions** | | | | |
| *If your aim for this study is to assess the effect of assignment to intervention, answer questions 4.1 and 4.2* | | | | |
| 4.1. Were there deviations from the intended intervention beyond what would be expected in usual practice? | N/PN | Y/PY | NI | Passive control group |
| 4.2. If Y/PY to 4.1: Were these deviations from intended intervention unbalanced between groups and likely to have affected the outcome? | N/PN | Y/PY | NA/NI | NA |
| *If your aim for this study is to assess the effect of starting and adhering to intervention, answer questions 4.3 to 4.6* | | | | NA |
| 4.3. Were important co-interventions balanced across intervention groups? | Y/PY | N/PN | NI | NA |
| 4.4. Was the intervention implemented successfully for most participants? | Y/PY | N/PN | NI | NA |
| 4.5. Did study participants adhere to the assigned intervention regimen? | Y/PY | N/PN | NI | NA |
| 4.6. If N/PN to 4.3, 4.4 or 4.5: Was an appropriate analysis used to estimate the effect of starting and adhering to the intervention? | Y/PY | N/PN | NA/NI | NA |
| Risk-of-bias judgment (low/moderate/serious/critical/NI) | Low risk of bias | | | |
| **Risk of bias due to missing data** | | | | |
| 5.1 Were outcome data available for all, or nearly all, participants? | Y/PY | N/PN | NI | Low response rate. |
| 5.2 Were participants excluded due to missing data on intervention status? | N/PN | Y/PY | NI | No information on reasons |

| | | | | |
|---|---|---|---|---|
| | | | | for low response rate. |
| 5.3 Were participants excluded due to missing data on other variables needed for the analysis? | N/PN | Y/PY | NI | See discussion. |
| 5.4 If PN/N to 5.1, or Y/PY to 5.2 or 5.3: Are the proportion of participants and reasons for missing data similar across interventions? | Y/PY | N/PN | NA/NI | "The response rates were higher in the intervention group, generating 101 complete cases vs. 41 in the control group." |
| 5.5 If PN/N to 5.1, or Y/PY to 5.2 or 5.3: Is there evidence that results were robust to the presence of missing data? | Y/PY | N/PN | NA/NI | See section "Statistical analysis" |
| Risk-of-bias judgment (low/moderate/serious/critical/NI) | Serious risk of bias | | | |
| **Risk of bias in measurement of outcomes** | | | | |
| 6.1 Could the outcome measure have been influenced by knowledge of the intervention received? | N/PN | Y/PY | NI | |
| 6.2 Were outcome assessors aware of the intervention received by study participants? | N/PN | Y/PY | NI | Participant reported outcome |
| 6.3 Were the methods of outcome assessment comparable across intervention groups? | Y/PY | N/PN | NI | |
| 6.4 Were any systematic errors in measurement of the outcome related to intervention received? | N/PN | Y/PY | NI | |

| | | | | |
|---|---|---|---|---|
| Risk-of-bias judgment (low/moderate/serious/critical/NI) | Moderate risk of bias | | | |
| **Risk of bias in selection of the reported result** | | | | |
| Is the reported effect estimate likely to be selected, on the basis of the results, from... | | | | |
| 7.1. ... multiple outcome measurements within the outcome domain? | N/PN | Y/PY | NI | Table 3 |
| 7.2 ... multiple analyses of the intervention-outcome relationship? | N/PN | Y/PY | NI | |
| 7.3 ... different subgroups? | N/PN | Y/PY | NI | |
| Risk-of-bias judgment (low/moderate/serious/critical/NI) | Low risk of bias | | | |
| **Overall bias** | | | | |
| Risk-of-bias judgment (low/moderate/serious/critical/NI) | Serious risk of bias | | | The study is judged to be at serious risk of bias in at least one domain, but not at critical risk of bias in any domain. |

| **Deval (42)** | | | | |
|---|---|---|---|---|
| Bias domain and signalling question* | Response options | | | Comments |
| | Lower risk of bias | Higher risk of bias | Other | |
| **Risk of bias due to confounding** | | | | |
| 1.1 Is there potential for confounding of the effect of intervention in this study? | N/PN | Y/PY | / | |

| | | | | |
|---|---|---|---|---|
| If N/PN to 1.1: the study can be considered to be at low risk of bias due to confounding and no further signalling questions need be considered | | | | |
| If Y/PY to 1.1: determine whether there is a need to assess time-varying confounding: | | | | |
| 1.2. Was the analysis based on splitting participants' follow up time according to intervention received? | N/PN | Y/PY | NA/NI | |
| If N/PN, answer questions relating to baseline confounding (1.4 to 1.6) | | | | |
| If Y/PY, go to question 1.3. | | | | |
| 1.3. Were intervention discontinuations or switches likely to be related to factors that are prognostic for the outcome? | N/PN | Y/PY | NA/NI | NA |
| If N/PN, answer questions relating to baseline confounding (1.4 to 1.6) | | | | |
| If Y/PY, answer questions relating to both baseline and time-varying confounding (1.7 and 1.8) | | | | |
| *Questions relating to baseline confounding only* | | | | |
| 1.4. Did the authors use an appropriate analysis method that controlled for all the important confounding domains? | Y/PY | N/PN | NA/NI | |
| 1.5. If Y/PY to 1.4: Were confounding domains that were controlled for measured validly and reliably by the variables available in this study? | Y/PY | N/PN | NA/NI | NA |
| 1.6. Did the authors control for any post-intervention variables that could have been affected by the intervention? | N/PN | Y/PY | NA/NI | NI |
| *Questions relating to baseline and time-varying confounding* | | | | |
| 1.7. Did the authors use an appropriate analysis method that controlled for all the important confounding domains and for time-varying confounding? | Y/PY | N/PN | NA/NI | NA |
| 1.8. If Y/PY to 1.7: Were confounding domains that were controlled for measured validly and reliably by the variables available in this study? | Y/PY | N/PN | NA/NI | NA |
| Risk-of-bias judgment (low/moderate/serious/critical/NI) | Serious risk of bias | | | |

| **Risk of bias in selection of participants into the study** | | | | |
|---|---|---|---|---|
| 2.1. Was selection of participants into the study (or into the analysis) based on participant characteristics observed after the start of intervention? | N/PN | Y/PY | NI | "During the first session, participants were asked to find a ''buddy'' (i.e. someone working in the same company, in a similar position and close to them in terms of personal characteristics such as age and gender). One hundred and twenty ''buddies'' constituted the control group." |
| If N/PN to 2.1: go to 2.4 | | | | |
| 2.2. If Y/PY to 2.1: Were the post-intervention variables that influenced selection likely to be associated with intervention? | N/PN | Y/PY | NA/NI | |
| 2.3 If Y/PY to 2.2: Were the post-intervention variables that influenced selection likely to be influenced by the outcome or a cause of the outcome? | N/PN | Y/PY | NA/NI | NA |
| 2.4. Do start of follow-up and start of intervention coincide for most participants? | Y/PY | N/PN | NI | See section "participants" |

| | | | | |
|---|---|---|---|---|
| 2.5. If Y/PY to 2.2 and 2.3, or N/PN to 2.4: Were adjustment techniques used that are likely to correct for the presence of selection biases? | Y/PY | N/PN | NA/NI | No clear information, but probably no. |
| Risk-of-bias judgment (low/moderate/serious/critical/NI) | Serious risk of bias | | | |
| **Risk of bias in classification of interventions** | | | | |
| 3.1 Were intervention groups clearly defined? | Y/PY | N/PN | NI | See section "ACT Intervention" |
| 3.2 Was the information used to define intervention groups recorded at the start of the intervention? | Y/PY | N/PN | NI | |
| 3.3 Could classification of intervention status have been affected by knowledge of the outcome or risk of the outcome? | N/PN | Y/PY | NI | |
| Risk-of-bias judgment (low/moderate/serious/critical/NI) | Low risk of bias | | | |
| **Risk of bias due to deviations from intended interventions** | | | | |
| *If your aim for this study is to assess the effect of assignment to intervention, answer questions 4.1 and 4.2* | | | | |
| 4.1. Were there deviations from the intended intervention beyond what would be expected in usual practice? | N/PN | Y/PY | NI | Passive control group |
| 4.2. If Y/PY to 4.1: Were these deviations from intended intervention unbalanced between groups and likely to have affected the outcome? | N/PN | Y/PY | NA/NI | NA |
| *If your aim for this study is to assess the effect of starting and adhering to intervention, answer questions 4.3 to 4.6* | | | | NA |
| 4.3. Were important co-interventions balanced across intervention groups? | Y/PY | N/PN | NI | NA |
| 4.4. Was the intervention implemented successfully for most participants? | Y/PY | N/PN | NI | NA |
| 4.5. Did study participants adhere to the assigned intervention regimen? | Y/PY | N/PN | NI | NA |

| | | | | |
|---|---|---|---|---|
| 4.6. If N/PN to 4.3, 4.4 or 4.5: Was an appropriate analysis used to estimate the effect of starting and adhering to the intervention? | Y/PY | N/PN | NA/NI | NA |
| Risk-of-bias judgment (low/moderate/serious/critical/NI) | Low risk of bias | | | |
| **Risk of bias due to missing data** | | | | |
| 5.1 Were outcome data available for all, or nearly all, participants? | Y/PY | N/PN | NI | "Of the initial 120 participants in the experimental and control group, 53 experimental group participants (44.17%) and 27 control group participants (22.50%) completed the two evaluations." |
| 5.2 Were participants excluded due to missing data on intervention status? | N/PN | Y/PY | NI | No information |
| 5.3 Were participants excluded due to missing data on other variables needed for the analysis? | N/PN | Y/PY | NI | No information |
| 5.4 If PN/N to 5.1, or Y/PY to 5.2 or 5.3: Are the proportion of participants and reasons for missing data similar across interventions? | Y/PY | N/PN | NA/NI | |
| 5.5 If PN/N to 5.1, or Y/PY to 5.2 or 5.3: Is there evidence that results were robust to the presence of missing data? | Y/PY | N/PN | NA/NI | |

| | | | | |
|---|---|---|---|---|
| Risk-of-bias judgment (low/moderate/serious/critical/NI) | Critical risk of bias | | | |
| **Risk of bias in measurement of outcomes** | | | | |
| 6.1 Could the outcome measure have been influenced by knowledge of the intervention received? | N/PN | Y/PY | NI | |
| 6.2 Were outcome assessors aware of the intervention received by study participants? | N/PN | Y/PY | NI | Participant reported outcomes |
| 6.3 Were the methods of outcome assessment comparable across intervention groups? | Y/PY | N/PN | NI | |
| 6.4 Were any systematic errors in measurement of the outcome related to intervention received? | N/PN | Y/PY | NI | |
| Risk-of-bias judgment (low/moderate/serious/critical/NI) | Moderate risk of bias | | | |
| **Risk of bias in selection of the reported result** | | | | |
| Is the reported effect estimate likely to be selected, on the basis of the results, from... | | | | |
| 7.1. ... multiple outcome measurements within the outcome domain? | N/PN | Y/PY | NI | Table 1 |
| 7.2 ... multiple analyses of the intervention-outcome relationship? | N/PN | Y/PY | NI | |
| 7.3 ... different subgroups? | N/PN | Y/PY | NI | |
| Risk-of-bias judgment (low/moderate/serious/critical/NI) | Low risk of bias | | | |
| **Overall bias** | | | | |
| Risk-of-bias judgment (low/moderate/serious/critical/NI) | Critical risk of bias | | | The study is judged to be at critical risk of bias in at least one domain. |

| |
|---|
| **Lange (57)** |

| Bias domain and signalling question* | Response options | | | Comments |
|---|---|---|---|---|
| | Lower risk of bias | Higher risk of bias | Other | |
| **Risk of bias due to confounding** | | | | |
| 1.1 Is there potential for confounding of the effect of intervention in this study? | N/PN | Y/PY | / | |
| If N/PN to 1.1: the study can be considered to be at low risk of bias due to confounding and no further signalling questions need be considered | | | | |
| If Y/PY to 1.1: determine whether there is a need to assess time-varying confounding: | | | | |
| 1.2. Was the analysis based on splitting participants' follow up time according to intervention received? | N/PN | Y/PY | NA/NI | |
| If N/PN, answer questions relating to baseline confounding (1.4 to 1.6) | | | | |
| If Y/PY, go to question 1.3. | | | | |
| 1.3. Were intervention discontinuations or switches likely to be related to factors that are prognostic for the outcome? | N/PN | Y/PY | NA/NI | NA |
| If N/PN, answer questions relating to baseline confounding (1.4 to 1.6) | | | | |
| If Y/PY, answer questions relating to both baseline and time-varying confounding (1.7 and 1.8) | | | | |
| *Questions relating to baseline confounding only* | | | | |
| 1.4. Did the authors use an appropriate analysis method that controlled for all the important confounding domains? | Y/PY | N/PN | NA/NI | See section "14 Analysis" |
| 1.5. If Y/PY to 1.4: Were confounding domains that were controlled for measured validly and reliably by the variables available in this study? | Y/PY | N/PN | NA/NI | NI |
| 1.6. Did the authors control for any post-intervention variables that could have been affected by the intervention? | N/PN | Y/PY | NA/NI | |

| | | | | |
|---|---|---|---|---|
| *Questions relating to baseline and time-varying confounding* | | | | |
| 1.7. Did the authors use an appropriate analysis method that controlled for all the important confounding domains and for time-varying confounding? | Y/PY | N/PN | NA/NI | NA |
| 1.8. If Y/PY to 1.7: Were confounding domains that were controlled for measured validly and reliably by the variables available in this study? | Y/PY | N/PN | NA/NI | NA |
| Risk-of-bias judgment (low/moderate/serious/critical/NI) | Moderate risk of bias | | | |
| **Risk of bias in selection of participants into the study** | | | | |
| 2.1. Was selection of participants into the study (or into the analysis) based on participant characteristics observed after the start of intervention? | N/PN | Y/PY | NI | |
| If N/PN to 2.1: go to 2.4 | | | | |
| 2.2. If Y/PY to 2.1: Were the post-intervention variables that influenced selection likely to be associated with intervention? | N/PN | Y/PY | NA/NI | NA |
| 2.3 If Y/PY to 2.2: Were the post-intervention variables that influenced selection likely to be influenced by the outcome or a cause of the outcome? | N/PN | Y/PY | NA/NI | NA |
| 2.4. Do start of follow-up and start of intervention coincide for most participants? | Y/PY | N/PN | NI | |
| 2.5. If Y/PY to 2.2 and 2.3, or N/PN to 2.4: Were adjustment techniques used that are likely to correct for the presence of selection biases? | Y/PY | N/PN | NA/NI | NA |
| Risk-of-bias judgment (low/moderate/serious/critical/NI) | Low risk of bias | | | |
| **Risk of bias in classification of interventions** | | | | |
| 3.1 Were intervention groups clearly defined? | Y/PY | N/PN | NI | |
| 3.2 Was the information used to define intervention groups recorded at the start of the intervention? | Y/PY | N/PN | NI | |
| 3.3 Could classification of intervention status have been affected by knowledge of the outcome or risk of the outcome? | N/PN | Y/PY | NI | |
| Risk-of-bias judgment (low/moderate/serious/critical/NI) | Low risk of bias | | | |

| | | | | |
|---|---|---|---|---|
| **Risk of bias due to deviations from intended interventions** | | | | |
| *If your aim for this study is to assess the effect of assignment to intervention, answer questions 4.1 and 4.2* | | | | |
| 4.1. Were there deviations from the intended intervention beyond what would be expected in usual practice? | N/PN | Y/PY | NI | |
| 4.2. If Y/PY to 4.1: Were these deviations from intended intervention unbalanced between groups and likely to have affected the outcome? | N/PN | Y/PY | NA/NI | |
| *If your aim for this study is to assess the effect of starting and adhering to intervention, answer questions 4.3 to 4.6* | | | | NA |
| 4.3. Were important co-interventions balanced across intervention groups? | Y/PY | N/PN | NI | NA |
| 4.4. Was the intervention implemented successfully for most participants? | Y/PY | N/PN | NI | NA |
| 4.5. Did study participants adhere to the assigned intervention regimen? | Y/PY | N/PN | NI | NA |
| 4.6. If N/PN to 4.3, 4.4 or 4.5: Was an appropriate analysis used to estimate the effect of starting and adhering to the intervention? | Y/PY | N/PN | NA/NI | NA |
| Risk-of-bias judgment (low/moderate/serious/critical/NI) | Low risk of bias | | | |
| **Risk of bias due to missing data** | | | | |
| 5.1 Were outcome data available for all, or nearly all, participants? | Y/PY | N/PN | NI | See Table 1 and section “12 Sample” |
| 5.2 Were participants excluded due to missing data on intervention status? | N/PN | Y/PY | NI | Clear intervention status |
| 5.3 Were participants excluded due to missing data on other variables needed for the analysis? | N/PN | Y/PY | NI | |
| 5.4 If PN/N to 5.1, or Y/PY to 5.2 or 5.3: Are the proportion of participants and reasons for missing data similar across interventions? | Y/PY | N/PN | NA/NI | NA |

| | | | | |
|---|---|---|---|---|
| 5.5 If PN/N to 5.1, or Y/PY to 5.2 or 5.3: Is there evidence that results were robust to the presence of missing data? | Y/PY | N/PN | NA/NI | NA |
| Risk-of-bias judgment (low/moderate/serious/critical/NI) | Low risk of bias | | | |
| **Risk of bias in measurement of outcomes** | | | | |
| 6.1 Could the outcome measure have been influenced by knowledge of the intervention received? | N/PN | Y/PY | NI | |
| 6.2 Were outcome assessors aware of the intervention received by study participants? | N/PN | Y/PY | NI | Participant reported outcomes |
| 6.3 Were the methods of outcome assessment comparable across intervention groups? | Y/PY | N/PN | NI | |
| 6.4 Were any systematic errors in measurement of the outcome related to intervention received? | N/PN | Y/PY | NI | |
| Risk-of-bias judgment (low/moderate/serious/critical/NI) | Moderate risk of bias | | | |
| **Risk of bias in selection of the reported result** | | | | |
| Is the reported effect estimate likely to be selected, on the basis of the results, from... | | | | |
| 7.1. ... multiple outcome measurements within the outcome domain? | N/PN | Y/PY | NI | Table 1 |
| 7.2 ... multiple analyses of the intervention-outcome relationship? | N/PN | Y/PY | NI | |
| 7.3 ... different subgroups? | N/PN | Y/PY | NI | Small sample size |
| Risk-of-bias judgment (low/moderate/serious/critical/NI) | Low risk of bias | | | |
| **Overall bias** | | | | |
| Risk-of-bias judgment (low/moderate/serious/critical/NI) | | | | |

**Ni (80)**

| Bias domain and signalling question* | Response options | | | Comments |
|---|---|---|---|---|
| | Lower risk of bias | Higher risk of bias | Other | |
| **Risk of bias due to confounding** | | | | |
| 1.1 Is there potential for confounding of the effect of intervention in this study? | N/PN | Y/PY | / | |
| If N/PN to 1.1: the study can be considered to be at low risk of bias due to confounding and no further signalling questions need be considered | | | | |
| If Y/PY to 1.1: determine whether there is a need to assess time-varying confounding: | | | | |
| 1.2. Was the analysis based on splitting participants' follow up time according to intervention received? | N/PN | Y/PY | NA/NI | |
| If N/PN, answer questions relating to baseline confounding (1.4 to 1.6) | | | | |
| If Y/PY, go to question 1.3. | | | | |
| 1.3. Were intervention discontinuations or switches likely to be related to factors that are prognostic for the outcome? | N/PN | Y/PY | NA/NI | NA |
| If N/PN, answer questions relating to baseline confounding (1.4 to 1.6) | | | | |
| If Y/PY, answer questions relating to both baseline and time-varying confounding (1.7 and 1.8) | | | | |
| *Questions relating to baseline confounding only* | | | | |
| 1.4. Did the authors use an appropriate analysis method that controlled for all the important confounding domains? | Y/PY | N/PN | NA/NI | See p. 12/13, section Hypothesis testing. |
| 1.5. If Y/PY to 1.4: Were confounding domains that were controlled for measured validly and reliably by the variables available in this study? | Y/PY | N/PN | NA/NI | NI |

| | | | | |
|---|---|---|---|---|
| 1.6. Did the authors control for any post-intervention variables that could have been affected by the intervention? | N/PN | Y/PY | NA/NI | NI |
| *Questions relating to baseline and time-varying confounding* | | | | |
| 1.7. Did the authors use an appropriate analysis method that controlled for all the important confounding domains and for time-varying confounding? | Y/PY | N/PN | NA/NI | NA |
| 1.8. If Y/PY to 1.7: Were confounding domains that were controlled for measured validly and reliably by the variables available in this study? | Y/PY | N/PN | NA/NI | NA |
| Risk-of-bias judgment (low/moderate/serious/critical/NI) | Serious risk of bias | | | |
| **Risk of bias in selection of participants into the study** | | | | |
| 2.1. Was selection of participants into the study (or into the analysis) based on participant characteristics observed after the start of intervention? | N/PN | Y/PY | NI | |
| If N/PN to 2.1: go to 2.4 | | | | |
| 2.2. If Y/PY to 2.1: Were the post-intervention variables that influenced selection likely to be associated with intervention? | N/PN | Y/PY | NA/NI | NA |
| 2.3 If Y/PY to 2.2: Were the post-intervention variables that influenced selection likely to be influenced by the outcome or a cause of the outcome? | N/PN | Y/PY | NA/NI | NA |
| 2.4. Do start of follow-up and start of intervention coincide for most participants? | Y/PY | N/PN | NI | |
| 2.5. If Y/PY to 2.2 and 2.3, or N/PN to 2.4: Were adjustment techniques used that are likely to correct for the presence of selection biases? | Y/PY | N/PN | NA/NI | NA |
| Risk-of-bias judgment (low/moderate/serious/critical/NI) | Low risk of bias | | | |
| **Risk of bias in classification of interventions** | | | | |
| 3.1 Were intervention groups clearly defined? | Y/PY | N/PN | NI | p. 9/10, section “sample and procedure” |

| | | | | |
|---|---|---|---|---|
| 3.2 Was the information used to define intervention groups recorded at the start of the intervention? | Y/PY | N/PN | NI | |
| 3.3 Could classification of intervention status have been affected by knowledge of the outcome or risk of the outcome? | N/PN | Y/PY | NI | |
| Risk-of-bias judgment (low/moderate/serious/critical/NI) | Low risk of bias | | | |
| **Risk of bias due to deviations from intended interventions** | | | | |
| *If your aim for this study is to assess the effect of assignment to intervention, answer questions 4.1 and 4.2* | | | | |
| 4.1. Were there deviations from the intended intervention beyond what would be expected in usual practice? | N/PN | Y/PY | NI | Waitlist control group |
| 4.2. If Y/PY to 4.1: Were these deviations from intended intervention unbalanced between groups and likely to have affected the outcome? | N/PN | Y/PY | NA/NI | NA |
| *If your aim for this study is to assess the effect of starting and adhering to intervention, answer questions 4.3 to 4.6* | | | | NA |
| 4.3. Were important co-interventions balanced across intervention groups? | Y/PY | N/PN | NI | NA |
| 4.4. Was the intervention implemented successfully for most participants? | Y/PY | N/PN | NI | NA |
| 4.5. Did study participants adhere to the assigned intervention regimen? | Y/PY | N/PN | NI | NA |
| 4.6. If N/PN to 4.3, 4.4 or 4.5: Was an appropriate analysis used to estimate the effect of starting and adhering to the intervention? | Y/PY | N/PN | NA/NI | NA |
| Risk-of-bias judgment (low/moderate/serious/critical/NI) | Low risk of bias | | | |
| **Risk of bias due to missing data** | | | | |
| 5.1 Were outcome data available for all, or nearly all, participants? | Y/PY | N/PN | NI | Only two participants were excluded due to |

| | | | | |
|---|---|---|---|---|
| | | | | missing data. |
| 5.2 Were participants excluded due to missing data on intervention status? | N/PN | Y/PY | NI | |
| 5.3 Were participants excluded due to missing data on other variables needed for the analysis? | N/PN | Y/PY | NI | |
| 5.4 If PN/N to 5.1, or Y/PY to 5.2 or 5.3: Are the proportion of participants and reasons for missing data similar across interventions? | Y/PY | N/PN | NA/NI | NA |
| 5.5 If PN/N to 5.1, or Y/PY to 5.2 or 5.3: Is there evidence that results were robust to the presence of missing data? | Y/PY | N/PN | NA/NI | NA |
| Risk-of-bias judgment (low/moderate/serious/critical/NI) | Low risk of bias | | | |
| **Risk of bias in measurement of outcomes** | | | | |
| 6.1 Could the outcome measure have been influenced by knowledge of the intervention received? | N/PN | Y/PY | NI | |
| 6.2 Were outcome assessors aware of the intervention received by study participants? | N/PN | Y/PY | NI | Participant reported outcomes |
| 6.3 Were the methods of outcome assessment comparable across intervention groups? | Y/PY | N/PN | NI | |
| 6.4 Were any systematic errors in measurement of the outcome related to intervention received? | N/PN | Y/PY | NI | |
| Risk-of-bias judgment (low/moderate/serious/critical/NI) | Moderate risk of bias | | | |
| **Risk of bias in selection of the reported result** | | | | |
| Is the reported effect estimate likely to be selected, on the basis of the results, from... | | | | |
| 7.1. ... multiple outcome measurements within the outcome domain? | N/PN | Y/PY | NI | |
| 7.2 ... multiple analyses of the intervention-outcome relationship? | N/PN | Y/PY | NI | |
| 7.3 ... different subgroups? | N/PN | Y/PY | NI | Small sample size |
| Risk-of-bias judgment (low/moderate/serious/critical/NI) | Low risk of bias | | | |

| **Overall bias** | | |
|---|---|---|
| Risk-of-bias judgment (low/moderate/serious/critical/NI) | | |

| **Reitz (84)** | | | | |
|---|---|---|---|---|
| Bias domain and signalling question* | Response options | | | Comments |
| | Lower risk of bias | Higher risk of bias | Other | |
| **Risk of bias due to confounding** | | | | |
| 1.1 Is there potential for confounding of the effect of intervention in this study? | N/PN | Y/PY | / | |
| If N/PN to 1.1: the study can be considered to be at low risk of bias due to confounding and no further signalling questions need be considered | | | | |
| If Y/PY to 1.1: determine whether there is a need to assess time-varying confounding: | | | | |
| 1.2. Was the analysis based on splitting participants' follow up time according to intervention received? | N/PN | Y/PY | NA/NI | |
| If N/PN, answer questions relating to baseline confounding (1.4 to 1.6) | | | | |
| If Y/PY, go to question 1.3. | | | | |
| 1.3. Were intervention discontinuations or switches likely to be related to factors that are prognostic for the outcome? | N/PN | Y/PY | NA/NI | NA |
| If N/PN, answer questions relating to baseline confounding (1.4 to 1.6) | | | | |
| If Y/PY, answer questions relating to both baseline and time-varying confounding (1.7 and 1.8) | | | | |
| *Questions relating to baseline confounding only* | | | | |

| | | | | |
|---|---|---|---|---|
| 1.4. Did the authors use an appropriate analysis method that controlled for all the important confounding domains? | Y/PY | N/PN | NA/NI | No information |
| 1.5. If Y/PY to 1.4: Were confounding domains that were controlled for measured validly and reliably by the variables available in this study? | Y/PY | N/PN | NA/NI | NA |
| 1.6. Did the authors control for any post-intervention variables that could have been affected by the intervention? | N/PN | Y/PY | NA/NI | No information. |
| *Questions relating to baseline and time-varying confounding* | | | | |
| 1.7. Did the authors use an appropriate analysis method that controlled for all the important confounding domains and for time-varying confounding? | Y/PY | N/PN | NA/NI | NA |
| 1.8. If Y/PY to 1.7: Were confounding domains that were controlled for measured validly and reliably by the variables available in this study? | Y/PY | N/PN | NA/NI | NA |
| Risk-of-bias judgment (low/moderate/serious/critical/NI) | Serious risk of bias | | | |
| **Risk of bias in selection of participants into the study** | | | | |
| 2.1. Was selection of participants into the study (or into the analysis) based on participant characteristics observed after the start of intervention? | N/PN | Y/PY | NI | |
| If N/PN to 2.1: go to 2.4 | | | | |
| 2.2. If Y/PY to 2.1: Were the post-intervention variables that influenced selection likely to be associated with intervention? | N/PN | Y/PY | NA/NI | NA |
| 2.3 If Y/PY to 2.2: Were the post-intervention variables that influenced selection likely to be influenced by the outcome or a cause of the outcome? | N/PN | Y/PY | NA/NI | NA |
| 2.4. Do start of follow-up and start of intervention coincide for most participants? | Y/PY | N/PN | NI | |
| 2.5. If Y/PY to 2.2 and 2.3, or N/PN to 2.4: Were adjustment techniques used that are likely to correct for the presence of selection biases? | Y/PY | N/PN | NA/NI | NA |
| Risk-of-bias judgment (low/moderate/serious/critical/NI) | Low risk of bias | | | |
| **Risk of bias in classification of interventions** | | | | |

| | | | | |
|---|---|---|---|---|
| 3.1 Were intervention groups clearly defined? | Y/PY | N/PN | NI | p. 227 |
| 3.2 Was the information used to define intervention groups recorded at the start of the intervention? | Y/PY | N/PN | NI | |
| 3.3 Could classification of intervention status have been affected by knowledge of the outcome or risk of the outcome? | N/PN | Y/PY | NI | |
| Risk-of-bias judgment (low/moderate/serious/critical/NI) | Low risk of bias | | | |
| **Risk of bias due to deviations from intended interventions** | | | | |
| *If your aim for this study is to assess the effect of assignment to intervention, answer questions 4.1 and 4.2* | | | | |
| 4.1. Were there deviations from the intended intervention beyond what would be expected in usual practice? | N/PN | Y/PY | NI | Waitlist control group |
| 4.2. If Y/PY to 4.1: Were these deviations from intended intervention unbalanced between groups and likely to have affected the outcome? | N/PN | Y/PY | NA/NI | NA |
| *If your aim for this study is to assess the effect of starting and adhering to intervention, answer questions 4.3 to 4.6* | | | | NA |
| 4.3. Were important co-interventions balanced across intervention groups? | Y/PY | N/PN | NI | NA |
| 4.4. Was the intervention implemented successfully for most participants? | Y/PY | N/PN | NI | NA |
| 4.5. Did study participants adhere to the assigned intervention regimen? | Y/PY | N/PN | NI | NA |
| 4.6. If N/PN to 4.3, 4.4 or 4.5: Was an appropriate analysis used to estimate the effect of starting and adhering to the intervention? | Y/PY | N/PN | NA/NI | NA |
| Risk-of-bias judgment (low/moderate/serious/critical/NI) | Low risk of bias | | | |
| **Risk of bias due to missing data** | | | | |
| 5.1 Were outcome data available for all, or nearly all, participants? | Y/PY | N/PN | NI | |
| 5.2 Were participants excluded due to missing data on intervention status? | N/PN | Y/PY | NI | |
| 5.3 Were participants excluded due to missing data on other variables needed for the analysis? | N/PN | Y/PY | NI | |

| | | | | |
|---|---|---|---|---|
| 5.4 If PN/N to 5.1, or Y/PY to 5.2 or 5.3: Are the proportion of participants and reasons for missing data similar across interventions? | Y/PY | N/PN | NA/NI | NA |
| 5.5 If PN/N to 5.1, or Y/PY to 5.2 or 5.3: Is there evidence that results were robust to the presence of missing data? | Y/PY | N/PN | NA/NI | NA |
| Risk-of-bias judgment (low/moderate/serious/critical/NI) | Low risk of bias | | | |
| **Risk of bias in measurement of outcomes** | | | | |
| 6.1 Could the outcome measure have been influenced by knowledge of the intervention received? | N/PN | Y/PY | NI | |
| 6.2 Were outcome assessors aware of the intervention received by study participants? | N/PN | Y/PY | NI | Participant reported outcomes |
| 6.3 Were the methods of outcome assessment comparable across intervention groups? | Y/PY | N/PN | NI | |
| 6.4 Were any systematic errors in measurement of the outcome related to intervention received? | N/PN | Y/PY | NI | |
| Risk-of-bias judgment (low/moderate/serious/critical/NI) | Moderate risk of bias | | | |
| **Risk of bias in selection of the reported result** | | | | |
| Is the reported effect estimate likely to be selected, on the basis of the results, from... | | | | |
| 7.1. ... multiple outcome measurements within the outcome domain? | N/PN | Y/PY | NI | Table III & IV |
| 7.2 ... multiple analyses of the intervention-outcome relationship? | N/PN | Y/PY | NI | |
| 7.3 ... different subgroups? | N/PN | Y/PY | NI | |
| Risk-of-bias judgment (low/moderate/serious/critical/NI) | Low risk of bias | | | |
| **Overall bias** | | | | |
| Risk-of-bias judgment (low/moderate/serious/critical/NI) | | | | |

## Vonderlin (94)

| Bias domain and signalling question* | Response options | | | Comments |
|---|---|---|---|---|
| | Lower risk of bias | Higher risk of bias | Other | |
| **Risk of bias due to confounding** | | | | |
| 1.1 Is there potential for confounding of the effect of intervention in this study? | N/PN | Y/PY | / | |
| If N/PN to 1.1: the study can be considered to be at low risk of bias due to confounding and no further signalling questions need be considered | | | | |
| If Y/PY to 1.1: determine whether there is a need to assess time-varying confounding: | | | | |
| 1.2. Was the analysis based on splitting participants' follow up time according to intervention received? | N/PN | Y/PY | NA/NI | |
| If N/PN, answer questions relating to baseline confounding (1.4 to 1.6) | | | | |
| If Y/PY, go to question 1.3. | | | | |
| 1.3. Were intervention discontinuations or switches likely to be related to factors that are prognostic for the outcome? | N/PN | Y/PY | NA/NI | NA |
| If N/PN, answer questions relating to baseline confounding (1.4 to 1.6) | | | | |
| If Y/PY, answer questions relating to both baseline and time-varying confounding (1.7 and 1.8) | | | | |
| *Questions relating to baseline confounding only* | | | | |
| 1.4. Did the authors use an appropriate analysis method that controlled for all the important confounding domains? | Y/PY | N/PN | NA/NI | See section "Statistical Analyses" |
| 1.5. If Y/PY to 1.4: Were confounding domains that were controlled for measured validly and reliably by the variables available in this study? | Y/PY | N/PN | NA/NI | See section "Statistical Analyses" |

| | | | | |
|---|---|---|---|---|
| 1.6. Did the authors control for any post-intervention variables that could have been affected by the intervention? | N/PN | Y/PY | NA/NI | No information |
| *Questions relating to baseline and time-varying confounding* | | | | |
| 1.7. Did the authors use an appropriate analysis method that controlled for all the important confounding domains and for time-varying confounding? | Y/PY | N/PN | NA/NI | NA |
| 1.8. If Y/PY to 1.7: Were confounding domains that were controlled for measured validly and reliably by the variables available in this study? | Y/PY | N/PN | NA/NI | NA |
| Risk-of-bias judgment (low/moderate/serious/critical/NI) | Moderate risk of bias | | | |
| **Risk of bias in selection of participants into the study** | | | | |
| 2.1. Was selection of participants into the study (or into the analysis) based on participant characteristics observed after the start of intervention? | N/PN | Y/PY | NI | Passive control group, health insurance data, Figure 2 |
| If N/PN to 2.1: go to 2.4 | | | | |
| 2.2. If Y/PY to 2.1: Were the post-intervention variables that influenced selection likely to be associated with intervention? | N/PN | Y/PY | NA/NI | See section “Control Group” |
| 2.3 If Y/PY to 2.2: Were the post-intervention variables that influenced selection likely to be influenced by the outcome or a cause of the outcome? | N/PN | Y/PY | NA/NI | NA |
| 2.4. Do start of follow-up and start of intervention coincide for most participants? | Y/PY | N/PN | NI | |
| 2.5. If Y/PY to 2.2 and 2.3, or N/PN to 2.4: Were adjustment techniques used that are likely to correct for the presence of selection biases? | Y/PY | N/PN | NA/NI | See section “Control group” |
| Risk-of-bias judgment (low/moderate/serious/critical/NI) | Moderate risk of bias | | | |

| | | | | |
|---|---|---|---|---|
| **Risk of bias in classification of interventions** | | | | |
| 3.1 Were intervention groups clearly defined? | Y/PY | N/PN | NI | p. 4 |
| 3.2 Was the information used to define intervention groups recorded at the start of the intervention? | Y/PY | N/PN | NI | |
| 3.3 Could classification of intervention status have been affected by knowledge of the outcome or risk of the outcome? | N/PN | Y/PY | NI | |
| Risk-of-bias judgment (low/moderate/serious/critical/NI) | Low risk of bias | | | |
| **Risk of bias due to deviations from intended interventions** | | | | |
| *If your aim for this study is to assess the effect of assignment to intervention, answer questions 4.1 and 4.2* | | | | |
| 4.1. Were there deviations from the intended intervention beyond what would be expected in usual practice? | N/PN | Y/PY | NI | Passive control group |
| 4.2. If Y/PY to 4.1: Were these deviations from intended intervention unbalanced between groups and likely to have affected the outcome? | N/PN | Y/PY | NA/NI | NA |
| *If your aim for this study is to assess the effect of starting and adhering to intervention, answer questions 4.3 to 4.6* | | | | NA |
| 4.3. Were important co-interventions balanced across intervention groups? | Y/PY | N/PN | NI | NA |
| 4.4. Was the intervention implemented successfully for most participants? | Y/PY | N/PN | NI | NA |
| 4.5. Did study participants adhere to the assigned intervention regimen? | Y/PY | N/PN | NI | NA |
| 4.6. If N/PN to 4.3, 4.4 or 4.5: Was an appropriate analysis used to estimate the effect of starting and adhering to the intervention? | Y/PY | N/PN | NA/NI | NA |
| Risk-of-bias judgment (low/moderate/serious/critical/NI) | Low risk of bias | | | |
| **Risk of bias due to missing data** | | | | |
| 5.1 Were outcome data available for all, or nearly all, participants? | Y/PY | N/PN | NI | |
| 5.2 Were participants excluded due to missing data on intervention status? | N/PN | Y/PY | NI | |

| | | | | |
|---|---|---|---|---|
| 5.3 Were participants excluded due to missing data on other variables needed for the analysis? | N/PN | Y/PY | NI | |
| 5.4 If PN/N to 5.1, or Y/PY to 5.2 or 5.3: Are the proportion of participants and reasons for missing data similar across interventions? | Y/PY | N/PN | NA/NI | |
| 5.5 If PN/N to 5.1, or Y/PY to 5.2 or 5.3: Is there evidence that results were robust to the presence of missing data? | Y/PY | N/PN | NA/NI | See section “Statistical Analyses” |
| Risk-of-bias judgment (low/moderate/serious/critical/NI) | Low risk of bias | | | |
| **Risk of bias in measurement of outcomes** | | | | |
| 6.1 Could the outcome measure have been influenced by knowledge of the intervention received? | N/PN | Y/PY | NI | |
| 6.2 Were outcome assessors aware of the intervention received by study participants? | N/PN | Y/PY | NI | Participant reported outcomes |
| 6.3 Were the methods of outcome assessment comparable across intervention groups? | Y/PY | N/PN | NI | |
| 6.4 Were any systematic errors in measurement of the outcome related to intervention received? | N/PN | Y/PY | NI | |
| Risk-of-bias judgment (low/moderate/serious/critical/NI) | Moderate risk of bias | | | |
| **Risk of bias in selection of the reported result** | | | | |
| Is the reported effect estimate likely to be selected, on the basis of the results, from... | | | | |
| 7.1. ... multiple outcome measurements within the outcome domain? | N/PN | Y/PY | NI | Table 2, 3 & 4 |
| 7.2 ... multiple analyses of the intervention-outcome relationship? | N/PN | Y/PY | NI | Table 2, 3 & 4 |
| 7.3 ... different subgroups? | N/PN | Y/PY | NI | “To achieve the highest possible level of comparability, |

| | | | | |
|---|---|---|---|---|
| | | | | the study participants in the CG were recruited in two steps using propensity score matching (PSM)—a statistical method to build comparable CGs in observational studies (Austin, 2011)." |
| Risk-of-bias judgment (low/moderate/serious/critical/NI) | Moderate risk of bias | | | |
| **Overall bias** | | | | |
| Risk-of-bias judgment (low/moderate/serious/critical/NI) | | | | |

| **Vonderlin (98)** | | | | |
|---|---|---|---|---|
| Bias domain and signalling question* | Response options | | | Comments |
| | Lower risk of bias | Higher risk of bias | Other | |
| **Risk of bias due to confounding** | | | | |
| 1.1 Is there potential for confounding of the effect of intervention in this study? | N/PN | Y/PY | / | |

| | | | | |
|---|---|---|---|---|
| If N/PN to 1.1: the study can be considered to be at low risk of bias due to confounding and no further signalling questions need be considered | | | | |
| If Y/PY to 1.1: determine whether there is a need to assess time-varying confounding: | | | | |
| 1.2. Was the analysis based on splitting participants' follow up time according to intervention received? | N/PN | Y/PY | NA/NI | |
| If N/PN, answer questions relating to baseline confounding (1.4 to 1.6) | | | | |
| If Y/PY, go to question 1.3. | | | | |
| 1.3. Were intervention discontinuations or switches likely to be related to factors that are prognostic for the outcome? | N/PN | Y/PY | NA/NI | NA |
| If N/PN, answer questions relating to baseline confounding (1.4 to 1.6) | | | | |
| If Y/PY, answer questions relating to both baseline and time-varying confounding (1.7 and 1.8) | | | | |
| *Questions relating to baseline confounding only* | | | | |
| 1.4. Did the authors use an appropriate analysis method that controlled for all the important confounding domains? | Y/PY | N/PN | NA/NI | "To address this limitation, we used propensity score matching to control for key confounding variables (e.g., age,gender)." |
| 1.5. If Y/PY to 1.4: Were confounding domains that were controlled for measured validly and reliably by the variables available in this study? | Y/PY | N/PN | NA/NI | No clear information |

| | | | | |
|---|---|---|---|---|
| 1.6. Did the authors control for any post-intervention variables that could have been affected by the intervention? | N/PN | Y/PY | NA/NI | No information |
| *Questions relating to baseline and time-varying confounding* | | | | |
| 1.7. Did the authors use an appropriate analysis method that controlled for all the important confounding domains and for time-varying confounding? | Y/PY | N/PN | NA/NI | NA |
| 1.8. If Y/PY to 1.7: Were confounding domains that were controlled for measured validly and reliably by the variables available in this study? | Y/PY | N/PN | NA/NI | NA |
| Risk-of-bias judgment (low/moderate/serious/critical/NI) | Moderate risk of bias | | | |
| **Risk of bias in selection of participants into the study** | | | | |
| 2.1. Was selection of participants into the study (or into the analysis) based on participant characteristics observed after the start of intervention? | N/PN | Y/PY | NI | Passive control group, health insurance data, Figure 1 |
| If N/PN to 2.1: go to 2.4 | | | | |
| 2.2. If Y/PY to 2.1: Were the post-intervention variables that influenced selection likely to be associated with intervention? | N/PN | Y/PY | NA/NI | |
| 2.3 If Y/PY to 2.2: Were the post-intervention variables that influenced selection likely to be influenced by the outcome or a cause of the outcome? | N/PN | Y/PY | NA/NI | NA |
| 2.4. Do start of follow-up and start of intervention coincide for most participants? | Y/PY | N/PN | NI | |
| 2.5. If Y/PY to 2.2 and 2.3, or N/PN to 2.4: Were adjustment techniques used that are likely to correct for the presence of selection biases? | Y/PY | N/PN | NA/NI | See section “Participants” |
| Risk-of-bias judgment (low/moderate/serious/critical/NI) | Moderate risk of bias | | | |
| **Risk of bias in classification of interventions** | | | | |

| | | | | |
|---|---|---|---|---|
| 3.1 Were intervention groups clearly defined? | Y/PY | N/PN | NI | Detailed description on recruitment process (p. 1754) and intervention. |
| 3.2 Was the information used to define intervention groups recorded at the start of the intervention? | Y/PY | N/PN | NI | |
| 3.3 Could classification of intervention status have been affected by knowledge of the outcome or risk of the outcome? | N/PN | Y/PY | NI | |
| Risk-of-bias judgment (low/moderate/serious/critical/NI) | Low risk of bias | | | |
| **Risk of bias due to deviations from intended interventions** | | | | |
| *If your aim for this study is to assess the effect of assignment to intervention, answer questions 4.1 and 4.2* | | | | |
| 4.1. Were there deviations from the intended intervention beyond what would be expected in usual practice? | N/PN | Y/PY | NI | Passive control group |
| 4.2. If Y/PY to 4.1: Were these deviations from intended intervention unbalanced between groups and likely to have affected the outcome? | N/PN | Y/PY | NA/NI | NA |
| *If your aim for this study is to assess the effect of starting and adhering to intervention, answer questions 4.3 to 4.6* | | | | NA |
| 4.3. Were important co-interventions balanced across intervention groups? | Y/PY | N/PN | NI | NA |
| 4.4. Was the intervention implemented successfully for most participants? | Y/PY | N/PN | NI | NA |
| 4.5. Did study participants adhere to the assigned intervention regimen? | Y/PY | N/PN | NI | NA |
| 4.6. If N/PN to 4.3, 4.4 or 4.5: Was an appropriate analysis used to estimate the effect of starting and adhering to the intervention? | Y/PY | N/PN | NA/NI | NA |
| Risk-of-bias judgment (low/moderate/serious/critical/NI) | Low risk of bias | | | |

| | | | | |
|---|---|---|---|---|
| **Risk of bias due to missing data** | | | | |
| 5.1 Were outcome data available for all, or nearly all, participants? | Y/PY | N/PN | NI | Figure 1 |
| 5.2 Were participants excluded due to missing data on intervention status? | N/PN | Y/PY | NI | |
| 5.3 Were participants excluded due to missing data on other variables needed for the analysis? | N/PN | Y/PY | NI | |
| 5.4 If PN/N to 5.1, or Y/PY to 5.2 or 5.3: Are the proportion of participants and reasons for missing data similar across interventions? | Y/PY | N/PN | NA/NI | Figure 1 |
| 5.5 If PN/N to 5.1, or Y/PY to 5.2 or 5.3: Is there evidence that results were robust to the presence of missing data? | Y/PY | N/PN | NA/NI | Section “Data Analyses” |
| Risk-of-bias judgment (low/moderate/serious/critical/NI) | Moderate risk of bias | | | |
| **Risk of bias in measurement of outcomes** | | | | |
| 6.1 Could the outcome measure have been influenced by knowledge of the intervention received? | N/PN | Y/PY | NI | Insurance data and 2-year follow-up |
| 6.2 Were outcome assessors aware of the intervention received by study participants? | N/PN | Y/PY | NI | Objective outcome |
| 6.3 Were the methods of outcome assessment comparable across intervention groups? | Y/PY | N/PN | NI | Insurance data |
| 6.4 Were any systematic errors in measurement of the outcome related to intervention received? | N/PN | Y/PY | NI | |
| Risk-of-bias judgment (low/moderate/serious/critical/NI) | Low risk of bias | | | |
| **Risk of bias in selection of the reported result** | | | | |
| Is the reported effect estimate likely to be selected, on the basis of the results, from... | | | | |
| 7.1. ... multiple outcome measurements within the outcome domain? | N/PN | Y/PY | NI | |

| | | | | |
|---|---|---|---|---|
| 7.2 ... multiple analyses of the intervention-outcome relationship? | N/PN | Y/PY | NI | Fig. 2 & Fig. 3 |
| 7.3 ... different subgroups? | N/PN | Y/PY | NI | "To achieve the highest possible level of comparability, we recruited study participants in the CG in two steps using propensity score matching (PSM)—a statistical method to build comparable CGs in observational studies (Austin, 2011)." |
| Risk-of-bias judgment (low/moderate/serious/critical/NI) | Moderate risk of bias | | | |
| **Overall bias** | | | | |
| Risk-of-bias judgment (low/moderate/serious/critical/NI) | Moderate risk of bias | | | The study is judged to be at low or moderate risk of bias for all domains |

| **Wasylkiw (99)** | | | | |
|---|---|---|---|---|
| Bias domain and signalling question* | Response options | | | Comments |
| | Lower risk of bias | Higher risk of bias | Other | |
| **Risk of bias due to confounding** | | | | |
| 1.1 Is there potential for confounding of the effect of intervention in this study? | N/PN | Y/PY | / | |
| If N/PN to 1.1: the study can be considered to be at low risk of bias due to confounding and no further signalling questions need be considered | | | | |
| If Y/PY to 1.1: determine whether there is a need to assess time-varying confounding: | | | | |
| 1.2. Was the analysis based on splitting participants' follow up time according to intervention received? | N/PN | Y/PY | NA/NI | |
| If N/PN, answer questions relating to baseline confounding (1.4 to 1.6) | | | | |
| If Y/PY, go to question 1.3. | | | | |
| 1.3. Were intervention discontinuations or switches likely to be related to factors that are prognostic for the outcome? | N/PN | Y/PY | NA/NI | NA |
| If N/PN, answer questions relating to baseline confounding (1.4 to 1.6) | | | | |
| If Y/PY, answer questions relating to both baseline and time-varying confounding (1.7 and 1.8) | | | | |
| *Questions relating to baseline confounding only* | | | | |
| 1.4. Did the authors use an appropriate analysis method that controlled for all the important confounding domains? | Y/PY | N/PN | NA/NI | No information on confounding. |

| | | | | |
|---|---|---|---|---|
| 1.5. If Y/PY to 1.4: Were confounding domains that were controlled for measured validly and reliably by the variables available in this study? | Y/PY | N/PN | NA/NI | NA |
| 1.6. Did the authors control for any post-intervention variables that could have been affected by the intervention? | N/PN | Y/PY | NA/NI | “Post hoc analysis with Wilcoxon signed-rank tests showed that scores on mindfulness were lower pre-intervention (M¼3.50, SE¼0.29) than those at four weeks postintervention (M¼4.35, SE¼0.16; Z¼−2.366, p¼0.018) and at eight weeks post-intervention (M¼4.30, SE¼0.28; Z¼−2.371, p¼0.018) but no differences between four and eight weeks post-intervention (Z¼−0.405, p¼0.069).” |

| | | | | |
|---|---|---|---|---|
| *Questions relating to baseline and time-varying confounding* | | | | |
| 1.7. Did the authors use an appropriate analysis method that controlled for all the important confounding domains and for time-varying confounding? | Y/PY | N/PN | NA/NI | NA |
| 1.8. If Y/PY to 1.7: Were confounding domains that were controlled for measured validly and reliably by the variables available in this study? | Y/PY | N/PN | NA/NI | NA |
| Risk-of-bias judgment (low/moderate/serious/critical/NI) | Serious risk of bias | | | |
| **Risk of bias in selection of participants into the study** | | | | |
| 2.1. Was selection of participants into the study (or into the analysis) based on participant characteristics observed after the start of intervention? | N/PN | Y/PY | NI | |
| If N/PN to 2.1: go to 2.4 | | | | |
| 2.2. If Y/PY to 2.1: Were the post-intervention variables that influenced selection likely to be associated with intervention? | N/PN | Y/PY | NA/NI | NA |
| 2.3 If Y/PY to 2.2: Were the post-intervention variables that influenced selection likely to be influenced by the outcome or a cause of the outcome? | N/PN | Y/PY | NA/NI | NA |
| 2.4. Do start of follow-up and start of intervention coincide for most participants? | Y/PY | N/PN | NI | |
| 2.5. If Y/PY to 2.2 and 2.3, or N/PN to 2.4: Were adjustment techniques used that are likely to correct for the presence of selection biases? | Y/PY | N/PN | NA/NI | NA |
| Risk-of-bias judgment (low/moderate/serious/critical/NI) | Low risk of bias | | | |
| **Risk of bias in classification of interventions** | | | | |
| 3.1 Were intervention groups clearly defined? | Y/PY | N/PN | NI | Information on recruitment process (p.898) as well as training program is presented (p.899). Due to |

| | | | | |
|---|---|---|---|---|
| | | | | program type (weekend program) participants had to take part here. |
| 3.2 Was the information used to define intervention groups recorded at the start of the intervention? | Y/PY | N/PN | NI | |
| 3.3 Could classification of intervention status have been affected by knowledge of the outcome or risk of the outcome? | N/PN | Y/PY | NI | |
| Risk-of-bias judgment (low/moderate/serious/critical/NI) | Low risk of bias | | | |
| **Risk of bias due to deviations from intended interventions** | | | | |
| *If your aim for this study is to assess the effect of assignment to intervention, answer questions 4.1 and 4.2* | | | | |
| 4.1. Were there deviations from the intended intervention beyond what would be expected in usual practice? | N/PN | Y/PY | NI | Passive control group |
| 4.2. If Y/PY to 4.1: Were these deviations from intended intervention unbalanced between groups and likely to have affected the outcome? | N/PN | Y/PY | NA/NI | NA |
| *If your aim for this study is to assess the effect of starting and adhering to intervention, answer questions 4.3 to 4.6* | | | | NA |
| 4.3. Were important co-interventions balanced across intervention groups? | Y/PY | N/PN | NI | NA |
| 4.4. Was the intervention implemented successfully for most participants? | Y/PY | N/PN | NI | NA |
| 4.5. Did study participants adhere to the assigned intervention regimen? | Y/PY | N/PN | NI | NA |
| 4.6. If N/PN to 4.3, 4.4 or 4.5: Was an appropriate analysis used to estimate the effect of starting and adhering to the intervention? | Y/PY | N/PN | NA/NI | NA |
| Risk-of-bias judgment (low/moderate/serious/critical/NI) | Low risk of bias | | | |
| **Risk of bias due to missing data** | | | | |

| | | | | |
|---|---|---|---|---|
| 5.1 Were outcome data available for all, or nearly all, participants? | Y/PY | N/PN | NI | |
| 5.2 Were participants excluded due to missing data on intervention status? | N/PN | Y/PY | NI | |
| 5.3 Were participants excluded due to missing data on other variables needed for the analysis? | N/PN | Y/PY | NI | |
| 5.4 If PN/N to 5.1, or Y/PY to 5.2 or 5.3: Are the proportion of participants and reasons for missing data similar across interventions? | Y/PY | N/PN | NA/NI | NA |
| 5.5 If PN/N to 5.1, or Y/PY to 5.2 or 5.3: Is there evidence that results were robust to the presence of missing data? | Y/PY | N/PN | NA/NI | NA |
| Risk-of-bias judgment (low/moderate/serious/critical/NI) | Low risk of bias | | | |
| **Risk of bias in measurement of outcomes** | | | | |
| 6.1 Could the outcome measure have been influenced by knowledge of the intervention received? | N/PN | Y/PY | NI | |
| 6.2 Were outcome assessors aware of the intervention received by study participants? | N/PN | Y/PY | NI | Participant reported outcomes |
| 6.3 Were the methods of outcome assessment comparable across intervention groups? | Y/PY | N/PN | NI | |
| 6.4 Were any systematic errors in measurement of the outcome related to intervention received? | N/PN | Y/PY | NI | |
| Risk-of-bias judgment (low/moderate/serious/critical/NI) | Moderate risk of bias | | | |
| **Risk of bias in selection of the reported result** | | | | |
| Is the reported effect estimate likely to be selected, on the basis of the results, from... | | | | |
| 7.1. ... multiple outcome measurements within the outcome domain? | N/PN | Y/PY | NI | Table II & III |
| 7.2 ... multiple analyses of the intervention-outcome relationship? | N/PN | Y/PY | NI | |
| 7.3 ... different subgroups? | N/PN | Y/PY | NI | Small sample size |
| Risk-of-bias judgment (low/moderate/serious/critical/NI) | Moderate risk of bias | | | |

| **Overall bias** | | |
|---|---|---|
| Risk-of-bias judgment (low/moderate/serious/critical/NI) | Serious risk of bias | The study is judged to be at serious risk of bias in at least one domain, but not at critical risk of bias in any domain. |

| **Yong (103)** | | | | |
|---|---|---|---|---|
| Bias domain and signalling question* | Response options | | | Comments |
| | Lower risk of bias | Higher risk of bias | Other | |
| **Risk of bias due to confounding** | | | | |
| 1.1 Is there potential for confounding of the effect of intervention in this study? | N/PN | Y/PY | / | |
| If N/PN to 1.1: the study can be considered to be at low risk of bias due to confounding and no further signalling questions need be considered | | | | |
| If Y/PY to 1.1: determine whether there is a need to assess time-varying confounding: | | | | |
| 1.2. Was the analysis based on splitting participants' follow up time according to intervention received? | N/PN | Y/PY | NA/NI | |
| If N/PN, answer questions relating to baseline confounding (1.4 to 1.6) | | | | |
| If Y/PY, go to question 1.3. | | | | |
| 1.3. Were intervention discontinuations or switches likely to be related to factors that are prognostic for the outcome? | N/PN | Y/PY | NA/NI | NA |

| | | | | |
|---|---|---|---|---|
| If N/PN, answer questions relating to baseline confounding (1.4 to 1.6) | | | | |
| If Y/PY, answer questions relating to both baseline and time-varying confounding (1.7 and 1.8) | | | | |
| *Questions relating to baseline confounding only* | | | | |
| 1.4. Did the authors use an appropriate analysis method that controlled for all the important confounding domains? | Y/PY | N/PN | NA/NI | See section “Data Analysis” |
| 1.5. If Y/PY to 1.4: Were confounding domains that were controlled for measured validly and reliably by the variables available in this study? | Y/PY | N/PN | NA/NI | No clear information |
| 1.6. Did the authors control for any post-intervention variables that could have been affected by the intervention? | N/PN | Y/PY | NA/NI | |
| *Questions relating to baseline and time-varying confounding* | | | | |
| 1.7. Did the authors use an appropriate analysis method that controlled for all the important confounding domains and for time-varying confounding? | Y/PY | N/PN | NA/NI | NA |
| 1.8. If Y/PY to 1.7: Were confounding domains that were controlled for measured validly and reliably by the variables available in this study? | Y/PY | N/PN | NA/NI | NA |
| Risk-of-bias judgment (low/moderate/serious/critical/NI) | Moderate risk of bias | | | |
| **Risk of bias in selection of participants into the study** | | | | |
| 2.1. Was selection of participants into the study (or into the analysis) based on participant characteristics observed after the start of intervention? | N/PN | Y/PY | NI | |
| If N/PN to 2.1: go to 2.4 | | | | |
| 2.2. If Y/PY to 2.1: Were the post-intervention variables that influenced selection likely to be associated with intervention? | N/PN | Y/PY | NA/NI | NA |
| 2.3 If Y/PY to 2.2: Were the post-intervention variables that influenced selection likely to be influenced by the outcome or a cause of the outcome? | N/PN | Y/PY | NA/NI | NA |

| | | | | |
|---|---|---|---|---|
| 2.4. Do start of follow-up and start of intervention coincide for most participants? | Y/PY | N/PN | NI | See section "Recruitment and Data Collection" |
| 2.5. If Y/PY to 2.2 and 2.3, or N/PN to 2.4: Were adjustment techniques used that are likely to correct for the presence of selection biases? | Y/PY | N/PN | NA/NI | No information |
| Risk-of-bias judgment (low/moderate/serious/critical/NI) | Low risk of bias | | | |
| **Risk of bias in classification of interventions** | | | | |
| 3.1 Were intervention groups clearly defined? | Y/PY | N/PN | NI | No clear information |
| 3.2 Was the information used to define intervention groups recorded at the start of the intervention? | Y/PY | N/PN | NI | No clear information |
| 3.3 Could classification of intervention status have been affected by knowledge of the outcome or risk of the outcome? | N/PN | Y/PY | NI | |
| Risk-of-bias judgment (low/moderate/serious/critical/NI) | Serious risk of bias | | | |
| **Risk of bias due to deviations from intended interventions** | | | | |
| *If your aim for this study is to assess the effect of assignment to intervention, answer questions 4.1 and 4.2* | | | | |
| 4.1. Were there deviations from the intended intervention beyond what would be expected in usual practice? | N/PN | Y/PY | NI | |
| 4.2. If Y/PY to 4.1: Were these deviations from intended intervention unbalanced between groups and likely to have affected the outcome? | N/PN | Y/PY | NA/NI | NA |
| *If your aim for this study is to assess the effect of starting and adhering to intervention, answer questions 4.3 to 4.6* | | | | NA |
| 4.3. Were important co-interventions balanced across intervention groups? | Y/PY | N/PN | NI | NA |
| 4.4. Was the intervention implemented successfully for most participants? | Y/PY | N/PN | NI | NA |

| | | | | |
|---|---|---|---|---|
| 4.5. Did study participants adhere to the assigned intervention regimen? | Y/PY | N/PN | NI | NA |
| 4.6. If N/PN to 4.3, 4.4 or 4.5: Was an appropriate analysis used to estimate the effect of starting and adhering to the intervention? | Y/PY | N/PN | NA/NI | NA |
| Risk-of-bias judgment (low/moderate/serious/critical/NI) | Low risk of bias | | | |
| **Risk of bias due to missing data** | | | | |
| 5.1 Were outcome data available for all, or nearly all, participants? | Y/PY | N/PN | NI | See section "Study Participants" |
| 5.2 Were participants excluded due to missing data on intervention status? | N/PN | Y/PY | NI | See section "Recruitment and Data Collection" |
| 5.3 Were participants excluded due to missing data on other variables needed for the analysis? | N/PN | Y/PY | NI | No clear information. |
| 5.4 If PN/N to 5.1, or Y/PY to 5.2 or 5.3: Are the proportion of participants and reasons for missing data similar across interventions? | Y/PY | N/PN | NA/NI | Table 2 |
| 5.5 If PN/N to 5.1, or Y/PY to 5.2 or 5.3: Is there evidence that results were robust to the presence of missing data? | Y/PY | N/PN | NA/NI | NI |
| Risk-of-bias judgment (low/moderate/serious/critical/NI) | Serious risk of bias | | | |
| **Risk of bias in measurement of outcomes** | | | | |
| 6.1 Could the outcome measure have been influenced by knowledge of the intervention received? | N/PN | Y/PY | NI | |
| 6.2 Were outcome assessors aware of the intervention received by study participants? | N/PN | Y/PY | NI | |
| 6.3 Were the methods of outcome assessment comparable across intervention groups? | Y/PY | N/PN | NI | |
| 6.4 Were any systematic errors in measurement of the outcome related to intervention received? | N/PN | Y/PY | NI | |
| Risk-of-bias judgment (low/moderate/serious/critical/NI) | Serious risk of bias | | | |

| | | | | |
|---|---|---|---|---|
| **Risk of bias in selection of the reported result** | | | | |
| Is the reported effect estimate likely to be selected, on the basis of the results, from... | | | | |
| 7.1. ... multiple outcome measurements within the outcome domain? | N/PN | Y/PY | NI | |
| 7.2 ... multiple analyses of the intervention-outcome relationship? | N/PN | Y/PY | NI | |
| 7.3 ... different subgroups? | N/PN | Y/PY | NI | |
| Risk-of-bias judgment (low/moderate/serious/critical/NI) | Low risk of bias | | | |
| **Overall bias** | | | | |
| Risk-of-bias judgment (low/moderate/serious/critical/NI) | Serious risk of bias | | | The study is judged to be at serious risk of bias in at least one domain, but not at critical risk of bias in any domain. |

# S7. Narrative study results

Four studies (30,48,56,103) could not be included in the meta-analysis due to a lack of available or insufficient data (e.g. no SD or pre or post data). Therefore, we will present the results of these studies in the following.

| **Allen (1980)** | | | |
|---|---|---|---|
| **Table II: Summary of Self-Reported Variables That Showed Significant Effects** | | | |
| **Condition** | **Pretraining** | **Posttraining** | **Follow-Up** |
| *Outcome domain: Psychological stress* | | | |
| Self-rating of stress experienced on Job (higher score is less stress) | | | |
| Stress management | 9.9 | 9.6 | 10.1 |
| Group discussion | 10.3 | 10.2 | 9.7 |
| Wait list | 10.8 | 10.5 | 11.4 |
| *Outcome domain: mental health* | | | |
| State anxiety (higher score is more anxious | | | |
| Stress management | 37. | 34.1 | 32.4 |
| Group discussion | 36.7 | 33.9 | 32.9 |
| Wait list | 33.5 | 31.3 | 30.7 |
| Trait anxiety (higher score is more anxious | | | |
| Stress management | 42.8 | 35.9 | 33.9 |
| Group discussion | 36.5 | 36.6 | 34.3 |
| Wait list | 35.6 | 35.7 | 33.6 |
| **Description in text, Allen (1980), p.433:**<br>“There was a main effect of time of assessment for state anxiety, F(2,54) = 3.86, p< .05, and trait anxiety, F(2,54) = 3.86, p< .10. For state anxiety there was a significant decrease, for all three groups combined, from pretest to posttest; the value for follow-up was also significantly lower than the pretest value but did not differ from the posttest score. For trait anxiety the posttest value did not differ significantly from the pretest value. However, the value for the follow-up assessment, for all three groups combined, was significantly (p< .05) lower than the pretest value.”<br>“For self-rating of the subjective experience of stress, at follow-up the groups differed significantly, F(2,27) = 2.55, p< .10. The waiting list group was significantly (p< .10) lower in report of experienced stress (higher numerical value) than the discussion group, with the stress management group falling between the two.” | | | |
| | | | |
| **Table III: Summary of Rating of Job Performance** | | | |
| **Condition** | **Pretraining** | **Posttraining** | **Follow-Up** |
| *Outcome domain: Work-related outcomes* | | | |
| Overall efficiency (higher score means more efficient) | | | |
| Stress management | 3.5 | 3.7 | 3.6 |
| Group discussion | 3.8 | 4.2 | 4.5 |
| Wait list | 4.1 | 4.2 | 4.4 |
| Handling of problems (higher score means better performance) | | | |
| Stress management | 3.8 | 3.8 | 3.9 |
| Group discussion | 4.1 | 4.4 | 4.4 |
| Wait list | 4.2 | 4.5 | 4.3 |
| **Description in text, Allen (1980), p.434:**<br>“For rated overall efficiency, there was a main effect for experimental conditions, F(2,54) = 2.67, p< .10. The participants in the wait list condition rated themselves as significantly more efficient, across assessment times, than the participants in the stress management condition.”<br>“Rated handling of problems also showed a main effect of experimental conditions at the posttest in the covariance analysis, F(2,27) = 2.66, p< .10. The wait list condition was again superior to the stress management condition at the. 10 level.” | | | |

| **Gast (2022)** | | | | | | |
|---|---|---|---|---|---|---|
| **Table 2: Medians, interquartile range, Mann–Whitney–U test statistics, and p-values for all variables collected** | | | | | | |
| | **Median T1 (Interquartile range)** | **Median T1 (Interquartile range)** | **Median T2 (Interquartile range)** | **Median T2 (Interquartile range)** | **Mann–Whitney –U test statistics** | **p** |
| | Intervention Group | Waiting group | Intervention Group | Waiting group | | |
| *Outcome: Psychological stress* | | | | | | |
| Perceived Stress Scale | 3.00 (2.00;5.00) | 4.00 (3.00;6.00) | 4.00 (2.00;6.00) | 4.00 (3.00;6.00) | 439.00 | 0.051 |
| Effort–Reward Effort Subscale | 15.00 (14.00;18.00) | 16.00 (14.25;19.00) | 17.00 (14.00;20.00) | 16.00 (14.00;19.75) | 459.00 | 0.183 |
| Effort–Reward Reword Subscale | 48.00 (39.00;43.00) | 50.00 (39.50;54.00) | 45.00 (33.75;53.00) | 51.00 (40.00;53.00) | 478.50 | 0.389 |
| Irritation Scale | 7.50 (14.00;24.00) | 22.00 (16.50;29.50) | 20.00 (16.00;28.50) | 21.00 (17.25;30.00) | 371.50 | 0.011 * |
| *Outcome: Mental health* | | | | | | |
| Patient Health Questionnaire -4 | 1.00 (0.00;2.00) | 1.50 (1.00;3.00) | 1.00 (0.00;3.00) | 1.50 (0.25;3.00) | 545.00 | 0.382 |
| * Significant at the 0.05 level | | | | | | |

**Description in text, Gast (2022), p.7.:**
"The Irritation Scale showed lower irritation in the IG at baseline, while values did not differ between IG and WG at follow-up. […] There were no significant changes in the Effort–Reward Imbalance (ERI), the Perceived Stress (PSS), […], nor regarding depression and anxiety symptoms (PHQ-4)."

**Janka (2017)**

*Results on outcome domain psychological stress.*
**Description in the results section from Janka (2017), p. 121f.:**
Figure 2 depicts the changes in the PSS score across the observation interval in both study groups. In the biofeedback group, the post-test score was markedly decreased compared to the pre-test score, and the score slightly increased at follow-up. In the control group, a small increase in the score was observed across the observation interval.

The ANOVA revealed an interaction between group and point of measurement (F[2, 68]=3.32, p=.042, partial eta squared=0.089). Post-hoc t tests comparing the pre-test value with those obtained at the post-test and follow-up revealed changes in the biofeedback group (pretest vs. post-test: t[17]=3.10, p=.007; pre-test vs. follow-up: t[17]=2.27, p=.036); the difference between the post-test and follow-up was not significant t[17]=−1.41, p=.18.

None of the post-hoc comparisons revealed significant changes across time in the control group (pre-test vs. post-test: t[17]=−0.27, p=.79; pre-test vs. follow-up: t[17]=−0.94, p=.36; pre-test vs. follow-up: t[17]=−0.69, p=.51). Cohen´s d computed in the biofeedback group was 0.86 for the change between the pre-test and post-test and 0.63 for the change between the pre-test and follow-up.

Fig. 2 Changes in PSS scores across the observation period in both study groups (*bars* represent standard errors of the mean)

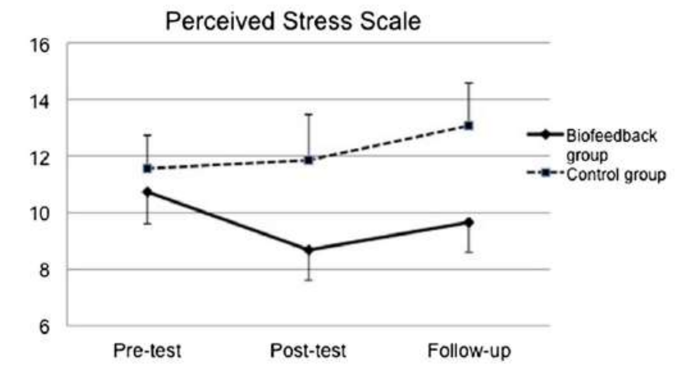


| **Yong (2020)** | | | | | | | |
|---|---|---|---|---|---|---|---|
| **Table 3: Pretest Score and Intervention Effects at Posttest 1,2 and 3 (N=45)[a]** | | | | | | | |
| | | **Intervention Effects Exp Change – Control Change** | | | | | |
| **Variable** | **Pretest Mean (SD)** | **Posttest** | **Mean** | **95% CI** | **P** | **Exp Change Mean (SE)** | **Control Change Mean (SE)** |
| *Outcome domain: Mental health* | | | | | | | |
| Burnout | 65.91 (13.72) | 1 | 6.97 | (-10.66, 24.60) | < .001[b] | -2.54 (9.41) | 18.62 (18.62) |
| | | 2 | 6.84 | (-8.18, 21.86) | < .001[b] | - 2.42 (8.95) | 30.67 (15.80) |
| | | 3 | 16.65 | (-4.09, 37.39) | .113 | -0.63 (13.88) | 21.05 (15.16) |
| | | 1-3 | 10.15 | (-4.75, 25.06) | .177 | -1.86 (9.46) | 23.44 (10.83) |
| Depression & anxiety | 42.24 (13.69) | 1 | -3.90 | (-10.03, 2.23) | .207 | -3.38 (12.56) | 0.52 (6.44 |
| | | 2 | -8.80 | (-16.83, -0.76) | .034[b] | -5.08 (13.22) | 3.71 (13.47) |
| | | 3 | -7.55 | (-15.42, 0.33) | .074[b] | -3.83 (12.72) | 3.71 (13.47) |
| | | 1-3 | -6.75 | (-13.12, -0.38) | .053[b] | -4.10 (11.44) | 2.65 (9.48) |
| *Outcome domain: Work-related outcomes* | | | | | | | |
| Job satisfaction | 28.27 (4.33) | 1 | 0.18 | (-1.70, 2.06) | .849 | -0.25 (3.04) | -0.43 (3.20) |
| | | 2 | 5.00 | (2.35, 7.66) | .001 | 0.29 (3.13) | -4.71 (5.52) |
| | | 3 | 3.00 | (0.01, 5.99) | .049 | 1.33 (3.66) | -1.67 (6.13 |
| | | 1-3 | 2.73 | (0.87, 4.49) | .005 | 0.46 (2.41) | -2.27 (3.72) |
| *Outcome domain: Leadership-related outcomes* | | | | | | | |
| Leadership practice | 202.78 (39.14) | 1 | 6.97 | (-10.66, 24.60) | .082[b] | 14.88 (27.67) | 7.90 (30.97) |
| | | 2 | 6.84 | (-8.18, 21.86) | .345[b] | 18.13 (20.45) | 11.29 (29.25) |
| | | 3 | 16.65 | (-4.09, 37.39) | .113 | 22.13 (25.02) | 5.48 (42.73) |
| | | 1-3 | 10.15 | (-4.75, 25.06) | .057[b] | 18.38 (20.88) | 8.22 (28.53) |
| *a* Pretest is baseline, posttest 1 is 5-week postintervention, posttest 2 is 12-week follow-up, and posttest 3 is 24-week follow-up. | | | | | | | |

| |
|---|
| *b* Wilcoxon rank sum tests of change scores for variables that were not normally distributed (p ≤ .10) on Shapiro-Wilk tests of pretest distributions. |
| **Description in text, Yong (2020), p.220f.:**<br>"*Job satisfaction* showed a significant increase in the experimental group compared with the control group in posttests 2 (d = 0.04, p = .001) and 3 (d = 1.15, p = .049). The mean time constant intervention effect of posttests 1-3 also showed a significant increase in the experimental group (2.73, d = 0.69, p = .005)."<br>"*Leadership* showed an increasing tendency in the experimental group during posttest 1 (d = 0.18, p = .082) but did not show a significant difference in posttests 2 and 3. The mean time constant intervention effect of posttests 1-3 showed increasing tendency in the experimental group (10.15, d = 0.26, p = .057)."<br>"*Burnout* in the experimental group was significantly reduced compared with the control group during posttest 1 (d = -1.54, p < .001) and posttest 2 (d = -2.41, p < .001). However, no significant difference was found in posttest 3; the mean time constant intervention effect of posttests 1-3 also was not significant."<br>"No significant differences were found in the two groups' *anxiety and depression* during posttest 1; however, a significant reduction in the experimental group was identified during posttest 2 (d = -0.64, p = .034). Posttest 3 showed a decreasing tendency in the experimental group (d = -0.55, p = .074), and the mean time constant intervention effect of posttests 1-3 showed a decreasing tendency in the experimental group (-6.75, d = -0.49, p = .053)." |

# S8. Meta-analysis results and sensitivity analyses of all analyzed interventions

## 8.1 Summary – All analyzed interventions

```
> summary(inter.total)
                              SMD             95%-CI %W(random)
Bennett (2011)             0,0847 [-0,2410;  0,4104]        5,6
Blank (2018)               0,1113 [-0,5090;  0,7316]        4,0
Cedestrand (2022)          0,4120 [-0,1056;  0,9295]        4,6
Deval (2017)              -0,0979 [-0,5615;  0,3658]        4,9
Igu (2023)                -1,4497 [-1,9544; -0,9450]        4,7
Lange (2019)              -0,3895 [-1,0409;  0,2619]        3,9
Li (2017)                 -0,1238 [-0,4100;  0,1623]        5,8
Limm (2011)               -0,0500 [-0,3661;  0,2660]        5,7
Ly (2014)                 -0,2751 [-0,7363;  0,1860]        4,9
Martin (2020)             -0,2082 [-0,5026;  0,0862]        5,8
Mellner (2022)             0,1307 [-0,4898;  0,7513]        4,0
Munafo (2016)             -0,0910 [-0,7959;  0,6138]        3,6
Ni (2022)                 -0,4025 [-0,8694;  0,0644]        4,9
Nübold (2020)              0,0421 [-0,2568;  0,3410]        5,8
Reitz (2020)              -0,2162 [-0,7377;  0,3054]        4,6
Sawyer (2023)             -0,1686 [-1,0711;  0,7340]        2,8
Shonin (2014)             -0,5778 [-0,9025; -0,2531]        5,6
Vonderlin (2023)          -0,1631 [-0,7199;  0,3936]        4,4
Wasylkiw (2015)           -0,1566 [-1,0146;  0,7013]        3,0
Zolnierczyk-Zreda (2016)   0,7254 [ 0,3878;  1,0629]        5,6
Vonderlin (2021)          -0,8507 [-1,1185; -0,5829]        5,9

Number of studies: k = 21
Number of observations: o = 2275 (o.e = 1029, o.c = 1246)

                         SMD            95%-CI     t p-value
Random effects model -0,1789 [-0,3837; 0,0260] -1,82  0,0835
Prediction interval          [-1,0327; 0,6749]

Quantifying heterogeneity:
 tau^2 = 0,1561 [0,0649; 0,3487]; tau = 0,3950 [0,2547; 0,5905]
 I^2 = 79,1% [68,7%; 86,1%]; H = 2,19 [1,79; 2,68]

Test of heterogeneity:
     Q d.f.  p-value
 95,75   20 < 0,0001

Details on meta-analytical method:
- Inverse variance method
- Restricted maximum-likelihood estimator for tau^2
- Q-Profile method for confidence interval of tau^2 and tau
- Hartung-Knapp adjustment for random effects model (df = 20)
- Prediction interval based on t-distribution (df = 19)
- Hedges' g (bias corrected standardised mean difference; using exact formulae)
```

## 8.2 Forest plot – All analyzed interventions

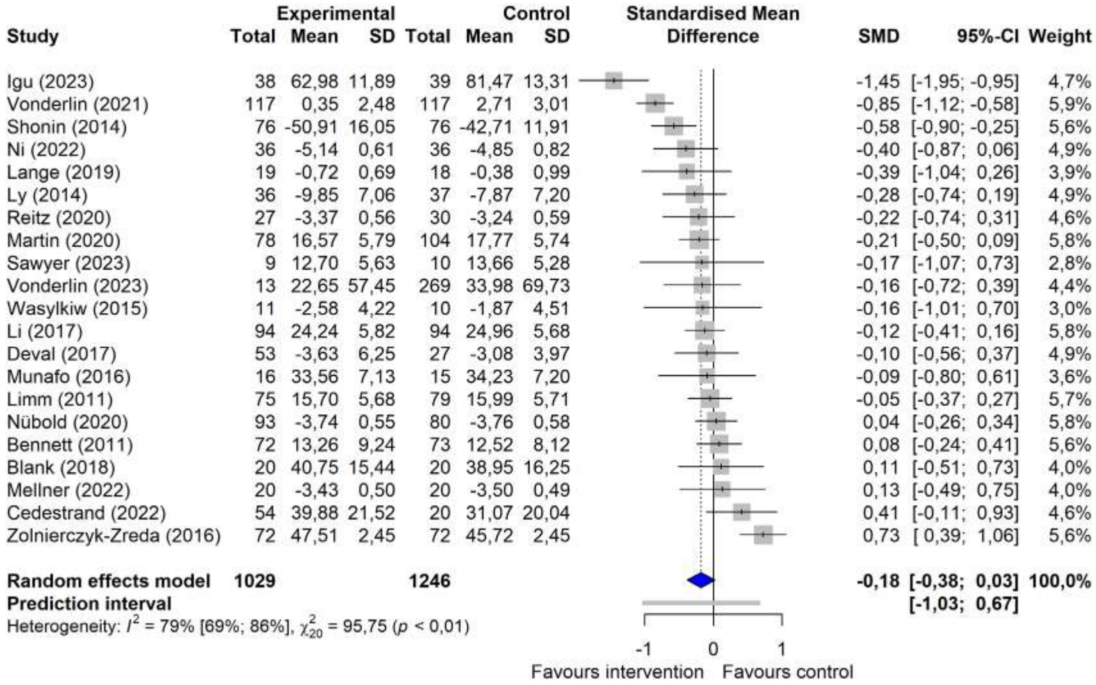


## 8.3 Sensitivity analysis – All analyzed interventions

```
> find.outliers(inter.total)
Identified outliers (random-effects model)
------------------------------------------
"Igu (2023)", "Zolnierczyk-Zreda (2016)", "Vonderlin (2021)"

Results with outliers removed
-----------------------------
Number of studies: k = 18
Number of observations: o = 1820 (o.e = 802, o.c = 1018)

                         SMD              95%-CI      t p-value
Random effects model -0,1268 [-0,2440; -0,0095] -2,12  0,0341
Prediction interval          [-0,4007;  0,1472]

Quantifying heterogeneity:
 tau^2 = 0,0131 [0,0000; 0,0616]; tau = 0,1146 [0,0000; 0,2481]
 I^2 = 9,1% [0,0%; 45,0%]; H = 1,05 [1,00; 1,35]

Test of heterogeneity:
     Q d.f. p-value
 18,70   17  0,3461

Details on meta-analytical method:
- Inverse variance method
- Restricted maximum-likelihood estimator for tau^2
- Q-Profile method for confidence interval of tau^2 and tau
- Hartung-Knapp adjustment for random effects model (df = )
- Prediction interval based on t-distribution (df = 16)
- Hedges' g (bias corrected standardised mean difference; using exact formulae)
```

# S9. Meta-analysis results and sensitivity analyses of main level outcome categories

## 9.1 Psychological stress

### 9.1.1 Summary – Psychological stress

```
> summary(stress.total)
                               SMD             95%-CI %W(random)
Blank (2018)                0,1113 [-0,5090;  0,7316]        8,3
Cedstrand (2022)            0,4120 [-0,1056;  0,9295]        8,9
Deval (2017)               -0,1374 [-0,6014;  0,3265]        9,3
Lange (2019)               -0,4694 [-1,1239;  0,1851]        8,1
Li (2017)                  -0,2403 [-0,5273;  0,0466]       10,2
Limm (2011)                -0,0841 [-0,4002;  0,2321]       10,0
Ly (2014)                  -0,2614 [-0,7223;  0,1995]        9,3
Sawyer (2023)              -0,0883 [-0,5353;  0,3587]        9,4
Shonin (2014)              -0,8468 [-1,1791; -0,5146]       10,0
Wasylkiw (2015)            -0,1127 [-0,9699;  0,7445]        6,9
Zolnierczyk-Zreda (2016)    1,4826 [ 1,1127;  1,8525]        9,8

Number of studies: k = 11
Number of observations: o = 1040 (o.e = 549, o.c = 491)

                           SMD            95%-CI     t p-value
Random effects model -0,0172 [-0,4257; 0,3913] -0,09  0,9271
Prediction interval          [-1,3868; 1,3524]

Quantifying heterogeneity:
 tau^2 = 0,3308 [0,1307; 1,0324]; tau = 0,5751 [0,3616; 1,0161]
 I^2 = 89,5% [83,3%; 93,4%]; H = 3,09 [2,45; 3,91]

Test of heterogeneity:
     Q d.f.  p-value
 95,64   10 < 0,0001

Details on meta-analytical method:
- Inverse variance method
- Restricted maximum-likelihood estimator for tau^2
- Q-Profile method for confidence interval of tau^2 and tau
- Hartung-Knapp adjustment for random effects model (df = 10)
- Prediction interval based on t-distribution (df = 9)
- Hedges' g (bias corrected standardised mean difference; using exact formulae)
```

## 9.1.2 Forest plot – Psychological stress

| Study | Experimental Total | Experimental Mean | Experimental SD | Control Total | Control Mean | Control SD | SMD | 95%-CI | Weight |
|---|---|---|---|---|---|---|---|---|---|
| Shonin (2014) | 76 | -111,72 | 27,15 | 76 | -91,47 | 19,89 | -0,85 | [-1,18; -0,51] | 10,0% |
| Lange (2019) | 19 | 2,65 | 0,89 | 18 | 3,23 | 1,50 | -0,47 | [-1,12; 0,19] | 8,1% |
| Ly (2014) | 36 | 21,92 | 7,82 | 37 | 23,89 | 7,05 | -0,26 | [-0,72; 0,20] | 9,3% |
| Li (2017) | 94 | 0,68 | 0,24 | 94 | 0,76 | 0,44 | -0,24 | [-0,53; 0,05] | 10,2% |
| Deval (2017) | 53 | 25,29 | 7,28 | 27 | 26,20 | 4,94 | -0,14 | [-0,60; 0,33] | 9,3% |
| Wasylkiw (2015) | 11 | 14,93 | 4,74 | 10 | 15,58 | 6,30 | -0,11 | [-0,97; 0,74] | 6,9% |
| Sawyer (2023) | 39 | 20,06 | 7,35 | 38 | 20,69 | 6,74 | -0,09 | [-0,54; 0,36] | 9,4% |
| Limm (2011) | 75 | 26,54 | 7,35 | 79 | 27,16 | 7,45 | -0,08 | [-0,40; 0,23] | 10,0% |
| Blank (2018) | 20 | 40,75 | 15,44 | 20 | 38,95 | 16,25 | 0,11 | [-0,51; 0,73] | 8,3% |
| Cedstrand (2022) | 54 | 39,88 | 21,52 | 20 | 31,07 | 20,04 | 0,41 | [-0,11; 0,93] | 8,9% |
| Zolnierczyk-Zreda (2016) | 72 | 132,50 | 4,23 | 72 | 126,20 | 4,23 | 1,48 | [ 1,11; 1,85] | 9,8% |
| **Random effects model** | **549** | | | **491** | | | **-0,02** | **[-0,43; 0,39]** | **100,0%** |
| **Prediction interval** | | | | | | | | **[-1,39; 1,35]** | |

Heterogeneity: $I^2$ = 90% [83%; 93%], $\chi^2_{10}$ = 95,64 ($p$ < 0,01)

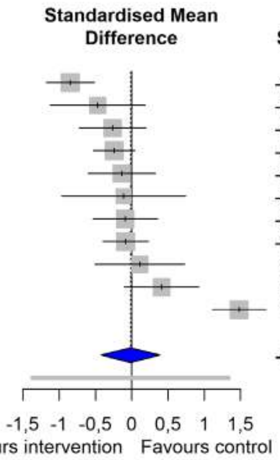


## 9.1.3 Sensitivity analysis – Psychological stress

```
> find.outliers(stress.total)
Identified outliers (random-effects model)
------------------------------------------
"Shonin (2014)", "Zolnierczyk-Zreda (2016)"

Results with outliers removed
-----------------------------
Number of studies: k = 9
Number of observations: o = 744 (o.e = 401, o.c = 343)

                          SMD             95%-CI     t p-value
Random effects model -0,1180 [-0,2770; 0,0410] -1,71  0,1253
Prediction interval          [-0,2949; 0,0588]

Quantifying heterogeneity:
 tau^2 = 0 [0,0000; 0,1463]; tau = 0 [0,0000; 0,3825]
 I^2 = 0,0% [0,0%; 64,8%]; H = 1,00 [1,00; 1,69]

Test of heterogeneity:
    Q d.f. p-value
 6,80    8  0,5585

Details on meta-analytical method:
- Inverse variance method
- Restricted maximum-likelihood estimator for tau^2
- Q-Profile method for confidence interval of tau^2 and tau
- Hartung-Knapp adjustment for random effects model (df = 8)
- Prediction interval based on t-distribution (df = 7)
- Hedges' g (bias corrected standardised mean difference; using exact formulae)
```

## 9.2 Mindfulness

### 9.2.1 Summary – Mindfulness

```
> summary(mindful.total)
                     SMD             95%-CI %W(random)
Lange (2019)    -0,1121 [-0,7574; 0,5331]       10,6
Deval (2017)     0,3616 [-0,1053; 0,8286]       16,3
Ni (2022)       -0,4025 [-0,8694; 0,0644]       16,3
Wasylkiw (2015) -0,5194 [-1,3931; 0,3543]        6,5
Mellner (2022)   0,1307 [-0,4898; 0,7513]       11,2
Nübold (2020)    0,1857 [-0,1138; 0,4853]       25,0
Reitz (2020)    -0,2488 [-0,7709; 0,2733]       14,2

Number of studies: k = 7
Number of observations: o = 480 (o.e = 259, o.c = 221)

                          SMD             95%-CI     t p-value
Random effects model -0,0266 [-0,3182; 0,2650] -0,22  0,8310
Prediction interval          [-0,6245; 0,5713]

Quantifying heterogeneity:
 tau^2 = 0,0386 [0,0000; 0,4221]; tau = 0,1964 [0,0000; 0,6497]
 I^2 = 34,3% [0,0%; 72,2%]; H = 1,23 [1,00; 1,90]

Test of heterogeneity:
    Q d.f. p-value
 9,14    6  0,1660

Details on meta-analytical method:
- Inverse variance method
- Restricted maximum-likelihood estimator for tau^2
- Q-Profile method for confidence interval of tau^2 and tau
- Hartung-Knapp adjustment for random effects model (df = 6)
- Prediction interval based on t-distribution (df = 5)
- Hedges' g (bias corrected standardised mean difference; using exact formulae)
```

### 9.2.2 Forest plot – Mindfulness

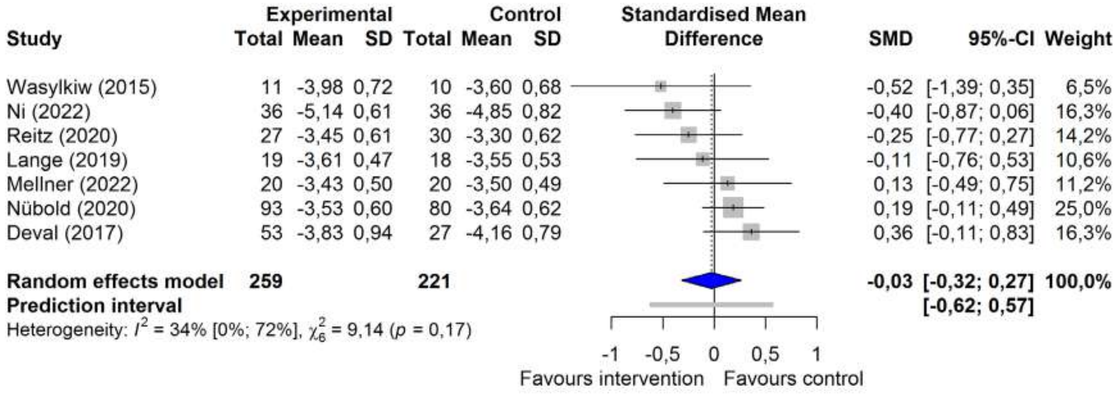


### 9.2.3 Sensitivity analysis – Mindfulness

```
> find.outliers(mindful.total)
No outliers detected (random-effects model).
```

# 9.3 Mental health

## 9.3.1 Summary – Mental health

```
> summary(mental.total)
                              SMD             95%-CI %W(random)
Limm (2011)                0,0165 [-0,2995;  0,3325]        8,9
Vonderlin (2021)          -0,2118 [-0,4688;  0,0452]        9,3
Igu (2023)                -1,4497 [-1,9544; -0,9450]        7,5
Sawyer (2023)             -0,2577 [-0,7064;  0,1910]        7,9
Li (2017)                 -0,1649 [-0,4512;  0,1215]        9,1
Deval (2017)              -0,2269 [-0,6917;  0,2379]        7,8
Ly (2014)                 -0,1445 [-0,6039;  0,3150]        7,9
Martin (2020)             -0,2082 [-0,5026;  0,0862]        9,1
Munafo (2016)             -0,0910 [-0,7959;  0,6138]        6,0
Bennett (2011)             0,0847 [-0,2410;  0,4104]        8,9
Shonin (2014)             -1,0856 [-1,4267; -0,7445]        8,8
Zolnierczyk-Zreda (2016) -0,9091 [-1,2526; -0,5655]        8,7

Number of studies: k = 12
Number of observations: o = 1537 (o.e = 766, o.c = 771)

                          SMD             95%-CI     t p-value
Random effects model -0,3831 [-0,6866; -0,0797] -2,78  0,0179
Prediction interval           [-1,3838;  0,6175]

Quantifying heterogeneity:
 tau^2 = 0,1830 [0,0720; 0,6264]; tau = 0,4278 [0,2683; 0,7914]
 I^2 = 82,7% [71,1%; 89,6%]; H = 2,40 [1,86; 3,11]

Test of heterogeneity:
     Q d.f.  p-value
 63,58   11 < 0,0001

Details on meta-analytical method:
- Inverse variance method
- Restricted maximum-likelihood estimator for tau^2
- Q-Profile method for confidence interval of tau^2 and tau
- Hartung-Knapp adjustment for random effects model (df = 11)
- Prediction interval based on t-distribution (df = 10)
- Hedges' g (bias corrected standardised mean difference; using exact formulae)
```

## 9.3.2 Forest plot – Mental health

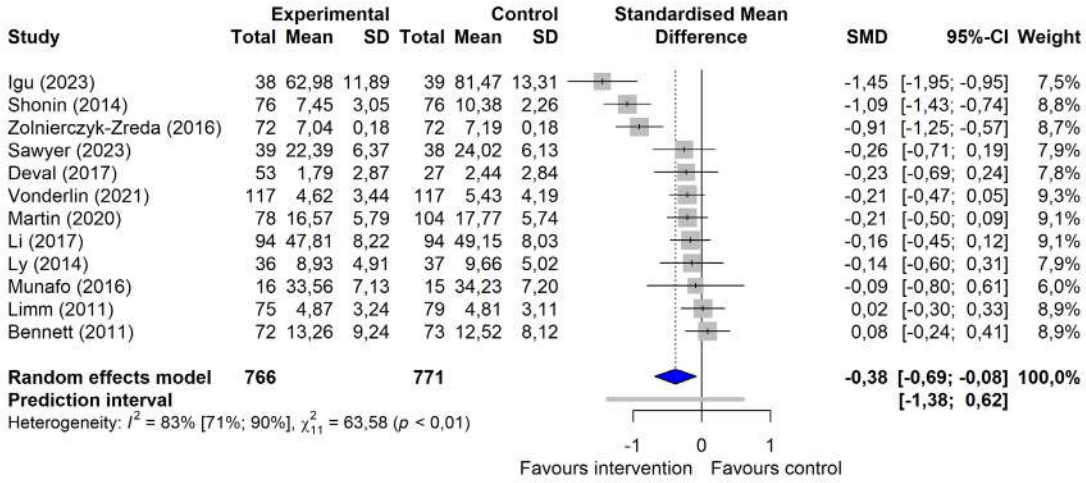

### 9.3.3 Sensitivity analysis – Mental health

```
> find.outliers(mental.total)
Identified outliers (random-effects model)
------------------------------------------
"Igu (2023)", "Shonin (2014)"

Results with outliers removed
-----------------------------
Number of studies: k = 10
Number of observations: o = 1308 (o.e = 652, o.c = 656)

                          SMD             95%-CI     t p-value
Random effects model -0,2151 [-0,4139; -0,0163] -2,45  0,0369
Prediction interval          [-0,7597;  0,3295]

Quantifying heterogeneity:
 tau^2 = 0,0474 [0,0037; 0,2090]; tau = 0,2177 [0,0605; 0,4571]
 I^2 = 57,9% [15,0%; 79,1%]; H = 1,54 [1,08; 2,19]

Test of heterogeneity:
     Q d.f. p-value
 21,36    9  0,0112

Details on meta-analytical method:
- Inverse variance method
- Restricted maximum-likelihood estimator for tau^2
- Q-Profile method for confidence interval of tau^2 and tau
- Hartung-Knapp adjustment for random effects model (df = 9)
- Prediction interval based on t-distribution (df = 8)
- Hedges' g (bias corrected standardised mean difference; using exact formulae)
```

## 9.4 Work-related outcomes

### 9.4.1 Summary – Work-related outcomes

```
> summary(work.total)
                              SMD             95%-CI %W(random)
Deval (2017)              -0,1121 [-0,5759;  0,3516]       19,9
Sawyer (2023)             -0,2638 [-0,7126;  0,1850]       20,0
Shonin (2014)             -0,5163 [-0,8396; -0,1930]       20,6
Vonderlin (2023)          -0,1631 [-0,7199;  0,3936]       19,4
Zolnierczyk-Zreda (2016) -2,3158 [-2,7402; -1,8913]       20,1

Number of studies: k = 5
Number of observations: o = 735 (o.e = 253, o.c = 482)

                          SMD             95%-CI     t p-value
Random effects model -0,6791 [-1,8361; 0,4779] -1,63  0,1785
Prediction interval          [-3,8490; 2,4909]

Quantifying heterogeneity:
 tau^2 = 0,8181 [0,2607; 7,1030]; tau = 0,9045 [0,5106; 2,6652]
 I^2 = 94,3% [89,5%; 96,9%]; H = 4,19 [3,09; 5,68]

Test of heterogeneity:
     Q d.f.  p-value
 70,24    4 < 0,0001

Details on meta-analytical method:
- Inverse variance method
- Restricted maximum-likelihood estimator for tau^2
- Q-Profile method for confidence interval of tau^2 and tau
- Hartung-Knapp adjustment for random effects model (df = 4)
- Prediction interval based on t-distribution (df = 3)
- Hedges' g (bias corrected standardised mean difference; using exact formulae)
```

### 9.4.2 Forest plot – Work-related outcomes

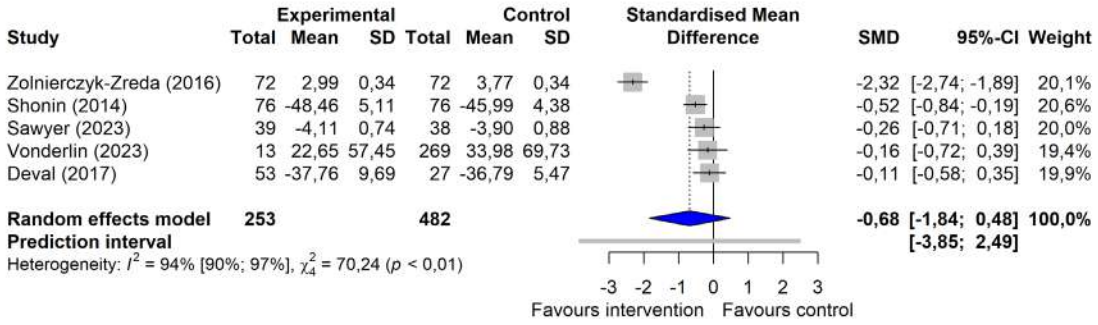


### 9.4.3 Sensitivity analysis – Work-related outcomes

```
> find.outliers(work.total)
Identified outliers (random-effects model)
------------------------------------------
"Zolnierczyk-Zreda (2016)"

Results with outliers removed
-----------------------------
Number of studies: k = 4
Number of observations: o = 591 (o.e = 181, o.c = 410)

                         SMD             95%-CI     t p-value
Random effects model -0,3195 [-0,6345; -0,0044] -3,23  0,0483
Prediction interval          [-0,8803;  0,2414]

Quantifying heterogeneity:
 tau^2 = 0,0042 [0,0000; 0,4096]; tau = 0,0645 [0,0000; 0,6400]
 I^2 = 0,0% [0,0%; 84,7%]; H = 1,00 [1,00; 2,56]

Test of heterogeneity:
    Q d.f. p-value
 2,55    3  0,4663

Details on meta-analytical method:
- Inverse variance method
- Restricted maximum-likelihood estimator for tau^2
- Q-Profile method for confidence interval of tau^2 and tau
- Hartung-Knapp adjustment for random effects model (df = 3)
- Prediction interval based on t-distribution (df = 2)
- Hedges' g (bias corrected standardised mean difference; using exact formulae)
```

# 9.5 Leadership-related outcomes

## 9.5.1 Summary – Leadership-related outcomes

```
> summary(leader.total)
                     SMD             95%-CI %W(random)
Ly (2014)        -0,3784 [-0,8416;  0,0847]       16,8
Lange (2019)     -0,5352 [-1,1926;  0,1222]       16,6
Wasylkiw (2015)  -0,2084 [-1,0676;  0,6508]       16,3
Nübold (2020)    -0,1290 [-0,4282;  0,1702]       16,9
Reitz (2020)     -0,1792 [-0,7003;  0,3418]       16,7
Vonderlin (2021) -5,5455 [-6,1131; -4,9778]       16,7

Number of studies: k = 6
Number of observations: o = 595 (o.e = 303, o.c = 292)

                         SMD            95%-CI     t p-value
Random effects model -1,1633 [-3,4233; 1,0966] -1,32  0,2430
Prediction interval          [-7,5716; 5,2449]

Quantifying heterogeneity:
 tau^2 = 4,5534 [1,7209; 27,7833]; tau = 2,1339 [1,3118; 5,2710]
 I^2 = 98,3% [97,6%; 98,8%]; H = 7,73 [6,41; 9,32]

Test of heterogeneity:
      Q d.f.  p-value
 298,54    5 < 0,0001

Details on meta-analytical method:
- Inverse variance method
- Restricted maximum-likelihood estimator for tau^2
- Q-Profile method for confidence interval of tau^2 and tau
- Hartung-Knapp adjustment for random effects model (df = 5)
- Prediction interval based on t-distribution (df = 4)
- Hedges' g (bias corrected standardised mean difference; using exact formulae)
```

## 9.5.2 Forest plot – Leadership-related outcomes

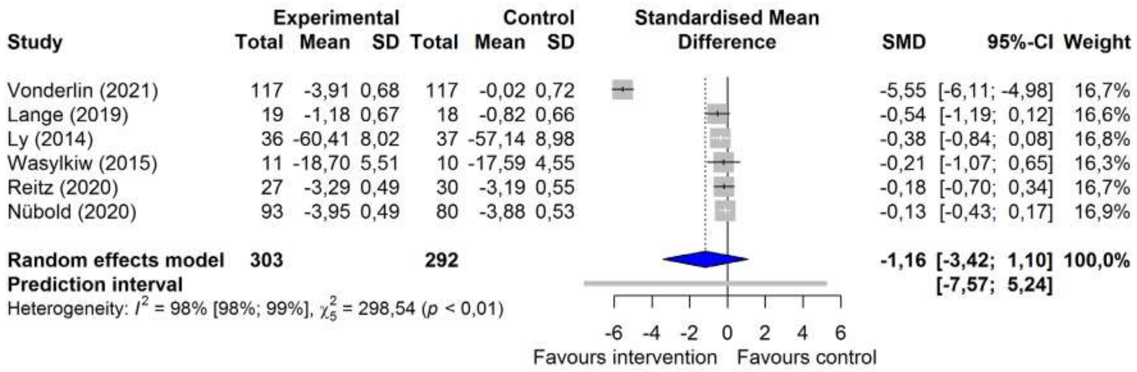

### 9.5.3 Sensitivity analysis – Leadership-related outcomes

```
> find.outliers(leader.total)
Identified outliers (random-effects model)
------------------------------------------
"Vonderlin (2021)"

Results with outliers removed
-----------------------------
Number of studies: k = 5
Number of observations: o = 361 (o.e = 186, o.c = 175)

                         SMD             95%-CI     t p-value
Random effects model -0,2323 [-0,4400; -0,0246] -2,19  0,0283
Prediction interval          [-0,5695;  0,1049]

Quantifying heterogeneity:
 tau^2 = 0 [0,0000; 0,1514]; tau = 0 [0,0000; 0,3891]
 I^2 = 0,0% [0,0%; 79,2%]; H = 1,00 [1,00; 2,19]

Test of heterogeneity:
    Q d.f. p-value
 1,70    4  0,7909

Details on meta-analytical method:
- Inverse variance method
- Restricted maximum-likelihood estimator for tau^2
- Q-Profile method for confidence interval of tau^2 and tau
- Hartung-Knapp adjustment for random effects model (df = )
- Prediction interval based on t-distribution (df = 3)
- Hedges' g (bias corrected standardised mean difference; using exact formulae)
```

# S10. Meta-analysis results and sensitivity analyses of identified subcategories

## 10.1 Perceived stress

### 10.1.1 Summary – Perceived stress

```
> summary(per.stress.total)
                      SMD            95%-CI %W(random)
Blank (2018)      0,1113 [-0,5090; 0,7316]       14,2
Deval (2017)     -0,1374 [-0,6014; 0,3265]       25,4
Ly (2014)        -0,2614 [-0,7223; 0,1995]       25,7
Sawyer (2023)    -0,2015 [-0,6495; 0,2464]       27,2
Wasylkiw (2015) -0,1127 [-0,9699; 0,7445]        7,4

Number of studies: k = 5
Number of observations: o = 291 (o.e = 159, o.c = 132)

                          SMD            95%-CI     t p-value
Random effects model -0,1496 [-0,3834; 0,0841] -1,25  0,2097
Prediction interval          [-0,5292; 0,2300]

Quantifying heterogeneity:
 tau^2 = 0 [0,0000; 0,0820]; tau = 0 [0,0000; 0,2863]
 I^2 = 0,0% [0,0%; 79,2%]; H = 1,00 [1,00; 2,19]

Test of heterogeneity:
    Q d.f. p-value
 0,97    4  0,9147

Details on meta-analytical method:
- Inverse variance method
- Restricted maximum-likelihood estimator for tau^2
- Q-Profile method for confidence interval of tau^2 and tau
- Hartung-Knapp adjustment for random effects model (df = )
- Prediction interval based on t-distribution (df = 3)
- Hedges' g (bias corrected standardised mean difference; using exact formulae)
```

### 10.1.2 Forest plot – Perceived stress

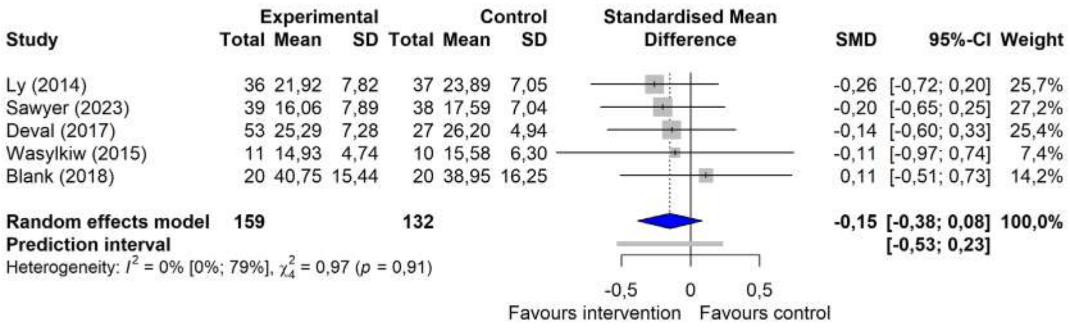


### 10.1.3 Sensitivity analysis – Perceived stress

```
> find.outliers(per.stress.total)
No outliers detected (random-effects model).
```

# 10.2 Job stress

## 10.2.1 Summary – Job stress

```
> summary(job.stress.total)
                              SMD              95%-CI %W(random)
Lange (2019)              -0,4694 [-1,1239;  0,1851]       18,5
Li (2017)                 -0,2403 [-0,5273;  0,0466]       20,6
Limm (2011)                0,1637 [-0,1528;  0,4803]       20,4
Shonin (2014)             -0,8468 [-1,1791; -0,5146]       20,4
Zolnierczyk-Zreda (2016)   1,4826 [ 1,1127;  1,8525]       20,2

Number of studies: k = 5
Number of observations: o = 675 (o.e = 336, o.c = 339)

                         SMD            95%-CI    t p-value
Random effects model 0,0243 [-1,0926; 1,1412] 0,06  0,9547
Prediction interval         [-3,0512; 3,0998]

Quantifying heterogeneity:
 tau^2 = 0,7710 [0,2507; 6,6048]; tau = 0,8781 [0,5007; 2,5700]
 I^2 = 95,7% [92,4%; 97,5%]; H = 4,80 [3,63; 6,36]

Test of heterogeneity:
     Q d.f.  p-value
 92,32    4 < 0,0001

Details on meta-analytical method:
- Inverse variance method
- Restricted maximum-likelihood estimator for tau^2
- Q-Profile method for confidence interval of tau^2 and tau
- Hartung-Knapp adjustment for random effects model (df = 4)
- Prediction interval based on t-distribution (df = 3)
- Hedges' g (bias corrected standardised mean difference; using exact formulae)
```

## 10.2.2 Forest plot – Job Stress

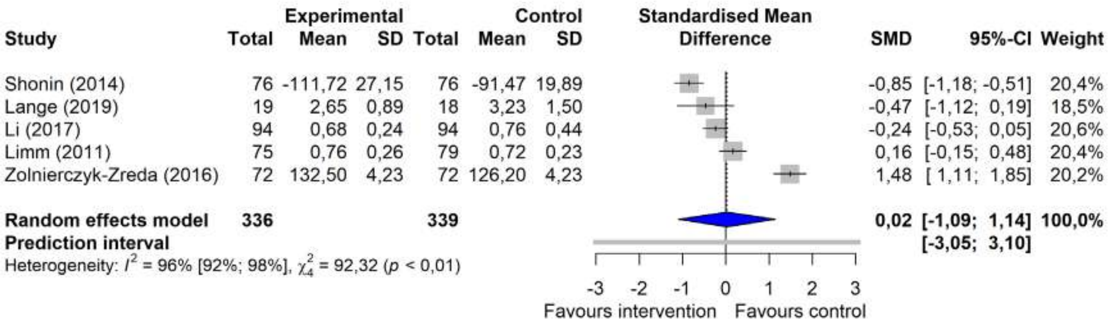


## 10.2.3 Sensitivity analysis – Job stress

```
> find.outliers(job.stress.total)
No outliers detected (random-effects model).
```

## 10.3 Subsyndromal symptoms

### 10.3.1 Summary – Subsyndromal symptoms

```
> summary(sub.mental.total)
                              SMD              95%-CI %W(random)
Limm (2011)                0,0165 [-0,2995;  0,3325]       13,3
Vonderlin (2021)          -0,2118 [-0,4688;  0,0452]       13,8
Igu (2023)                -1,4497 [-1,9544; -0,9450]       11,5
Sawyer (2023)             -0,2577 [-0,7064;  0,1910]       12,0
Li (2017)                 -0,1649 [-0,4512;  0,1215]       13,6
Munafo (2016)             -0,0910 [-0,7959;  0,6138]        9,4
Bennett (2011)             0,0847 [-0,2410;  0,4104]       13,3
Zolnierczyk-Zreda (2016) -0,9091 [-1,2526; -0,5655]       13,1

Number of studies: k = 8
Number of observations: o = 1050 (o.e = 523, o.c = 527)

                          SMD            95%-CI     t p-value
Random effects model -0,3630 [-0,7980; 0,0720] -1,97  0,0891
Prediction interval          [-1,5937; 0,8677]

Quantifying heterogeneity:
 tau^2 = 0,2201 [0,0722; 1,1115]; tau = 0,4692 [0,2687; 1,0543]
 I^2 = 83,7% [69,4%; 91,3%]; H = 2,47 [1,81; 3,39]

Test of heterogeneity:
     Q d.f.  p-value
 42,87    7 < 0,0001

Details on meta-analytical method:
- Inverse variance method
- Restricted maximum-likelihood estimator for tau^2
- Q-Profile method for confidence interval of tau^2 and tau
- Hartung-Knapp adjustment for random effects model (df = 7)
- Prediction interval based on t-distribution (df = 6)
- Hedges' g (bias corrected standardised mean difference; using exact formulae)
```

### 10.3.2 Forest plot – Subsyndromal symptoms

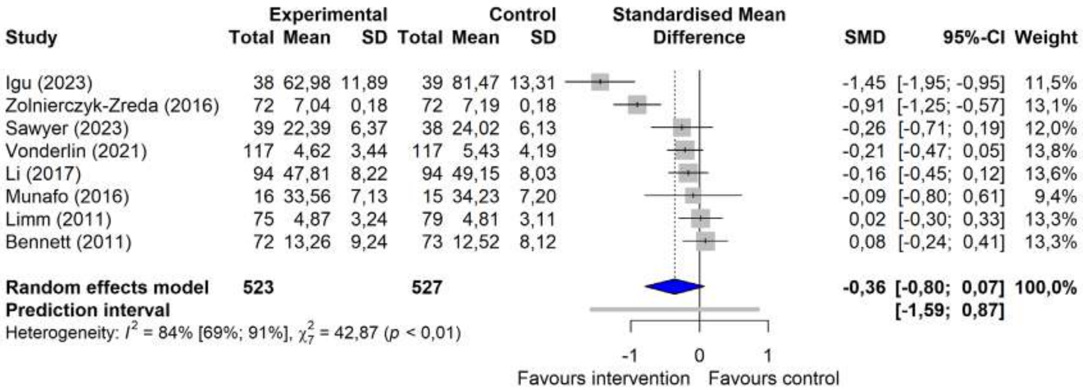

### 10.3.3 Sensitivity analysis – Subsyndromal symptoms

```
> find.outliers(sub.mental.total)
Identified outliers (random-effects model)
------------------------------------------
"Igu (2023)"

Results with outliers removed
----------------------------
Number of studies: k = 7
Number of observations: o = 973 (o.e = 485, o.c = 488)

                         SMD            95%-CI     t p-value
Random effects model -0,2233 [-0,5332; 0,0865] -1,76  0,1283
Prediction interval          [-1,0342; 0,5875]

Quantifying heterogeneity:
 tau^2 = 0,0825 [0,0141; 0,4891]; tau = 0,2873 [0,1187; 0,6993]
 I^2 = 71,8% [38,8%; 87,0%]; H = 1,88 [1,28; 2,77]

Test of heterogeneity:
     Q d.f. p-value
 21,27    6  0,0016

Details on meta-analytical method:
- Inverse variance method
- Restricted maximum-likelihood estimator for tau^2
- Q-Profile method for confidence interval of tau^2 and tau
- Hartung-Knapp adjustment for random effects model (df = 6)
- Prediction interval based on t-distribution (df = 5)
- Hedges' g (bias corrected standardised mean difference; using exact formulae)
```

## 10.4 General mental health

### 10.4.1 Summary – General mental health

```
> summary(gen.mental.total)
                    SMD              95%-CI %W(random)
Deval (2017)   -0,2269 [-0,6917;  0,2379]       18,4
Ly (2014)      -0,1445 [-0,6039;  0,3150]       18,5
Martin (2020)  -0,2082 [-0,5026;  0,0862]       21,5
Bennett (2011)  0,0847 [-0,2410;  0,4104]       21,0
Shonin (2014)  -1,0856 [-1,4267; -0,7445]       20,7

Number of studies: k = 5
Number of observations: o = 632 (o.e = 315, o.c = 317)

                         SMD            95%-CI     t p-value
Random effects model -0,3200 [-0,8848; 0,2449] -1,57  0,1909
Prediction interval          [-1,8106; 1,1706]

Quantifying heterogeneity:
 tau^2 = 0,1766 [0,0415; 1,6244]; tau = 0,4202 [0,2036; 1,2745]
 I^2 = 84,9% [66,5%; 93,2%]; H = 2,58 [1,73; 3,84]

Test of heterogeneity:
     Q d.f.  p-value
 26,55    4 < 0,0001

Details on meta-analytical method:
- Inverse variance method
- Restricted maximum-likelihood estimator for tau^2
- Q-Profile method for confidence interval of tau^2 and tau
- Hartung-Knapp adjustment for random effects model (df = 4)
- Prediction interval based on t-distribution (df = 3)
- Hedges' g (bias corrected standardised mean difference; using exact formulae)
```

## 10.4.2 Forest plot – General mental health

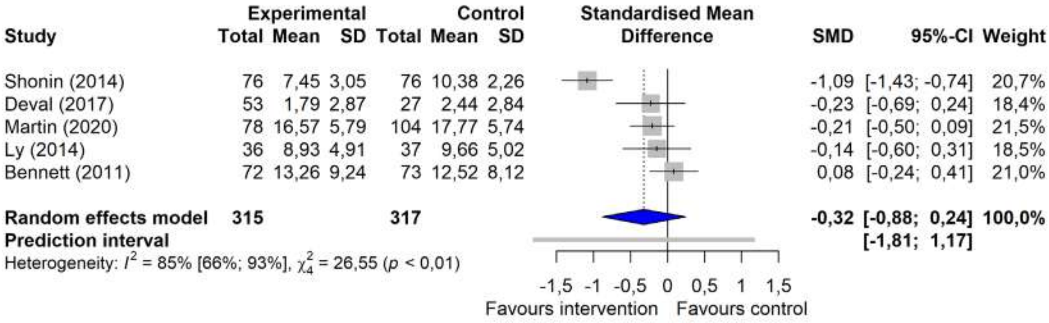


## 10.4.3 Sensitivity analysis – General mental health

```
> find.outliers(gen.mental.total)
No outliers detected (random-effects model).
```

# S11. Results of subgroup analyses

Subgroup analyses were performed, when outcomes pooled more than 10 studies.

## 11.1 Subgroup analyses – All analyzed interventions

| | k | g | 95% CI | p | $I^2$ | Q | df | $p_{subgroup}$ |
|---|---|---|---|---|---|---|---|---|
| **Risk of bias** | | | | | | 0.70 | 1 | 0.4 |
| Some concerns | 9 | -0.27 | -0.54 to -0.01 | 0.04 | 72.2% | | | |
| High | 12 | -0.12 | -0.45 to 0.22 | 0.46 | 80.9% | | | |
| **Study design** | | | | | | 0.45 | 1 | 0.5 |
| RCT | 13 | -0.14 | -0.43 to 0.16 | 0.34 | 81.0% | | | |
| CBA | 8 | -0.26 | -0.59 to 0.05 | 0.09 | 70.2% | | | |
| **Control group** | | | | | | 0.12 | 2 | 0.94 |
| Passive | 8 | -0.18 | -0.50 to 0.14 | 0.24 | 77.5% | | | |
| Waitlist | 10 | -0.17 | -0.57 to 0.22 | 0.34 | 83.6% | | | |
| Alternative | 3 | -0.26 | -1.19 to 0.67 | 0.35 | 55.4% | | | |
| **Follow-up** | | | | | | 1.93 | 3 | 0.59 |
| Short-term | 8 | -0.11 | -0.28 to 0.05 | 0.18 | 0.0% | | | |
| Medium-term | 6 | -0.13 | -0.70 to 0.45 | 0.59 | 90.9% | | | |
| Long-term | 4 | -0.03 | -0.36 to 0.29 | 0.76 | 13.0% | | | |
| Mixed | 3 | -0.64 | -2.58 to 1.29 | 0.29 | 87.4% | | | |
| **Management level** | | | | | | 1.24 | 3 | 0.74 |
| Lower | 3 | -0.04 | -1.15 to 1.06 | 0.88 | 62.4% | | | |
| Middle | 5 | -0.03 | -0.68 to 0.62 | 0.92 | 87.3% | | | |
| Diverse | 8 | -0.20 | -0.48 to 0.82 | 0.14 | 74.6% | | | |
| NR | 5 | -0.37 | -1.10 to 0.37 | 0.24 | 84.5% | | | |
| **Higher proportion (>50%) of female participants** | | | | | | 5.77 | 2 | 0.06 |
| Yes | 10 | -0.27 | -0.50 to -0.03 | 0.03 | 66.6% | | | |
| No | 10 | -0.17 | -0.56 to 0.22 | 0.34 | 83.5% | | | |
| NR | 1 | 0.41 | -0.10 to 0.93 | 0.12 | / | | | |
| **Intervention type** | | | | | | 6.12 | 5 | 0.29 |
| Multimodal | 9 | -0.13 | -0.50 to 0.22 | 0.41 | 85.8% | | | |
| Alternative to the others | 1 | 0.11 | -0.51 to 0.73 | 0.72 | / | | | |
| Organization-focused | 1 | 0.41 | -0.10 to 0.93 | 0.12 | / | | | |
| Cognitive-Behavioral | 6 | -0.34 | -0.89 to 0.20 | 0.16 | 79.6% | | | |
| Biofeedback | 1 | -0.09 | -0.79 to 0.61 | 0.80 | / | | | |
| Relaxation | 3 | -0.24 | -1.11 to 0.62 | 0.35 | 73.7% | | | |
| **No. of intervention type components** | | | | | | 4.74 | 2 | 0.09 |
| 1 | 12 | -0.21 | -0.50 to 0.07 | 0.13 | 71.9% | | | |
| 2 | 6 | 0.06 | -0.38 to 0.50 | 0.73 | 73.8% | | | |
| 3 | 3 | -0.53 | -1.45 to 0.39 | 0.13 | 64.5% | | | |
| **Intervention length** | | | | | | 2.20 | 5 | 0.82 |
| <1 week | 1 | 0.11 | -0.51 to 0.73 | 0.72 | / | | | |
| 1-4 weeks | 2 | -0.14 | -2.93 to 2.64 | 0.63 | 59.5% | | | |
| 5-8 weeks | 7 | -0.25 | -0.48 to -0.03 | 0.03 | 0.0% | | | |
| 9-12 weeks | 4 | -0.31 | -1.78 to 1.15 | 0.54 | 94.1% | | | |
| >12 weeks | 5 | -0.23 | -0.70 to 0.24 | 0.24 | 84.4% | | | |
| NR | 2 | 0.63 | -3.85 to 3.97 | 0.87 | 76.0% | | | |
| **Delivery Mode** | | | | | | 2.62 | 3 | 0.45 |
| Self-practice | 4 | -0.07 | -0.33 to 0.20 | 0.49 | 0.4% | | | |
| Individual sessions | 1 | -0.09 | -0.79 to 0.61 | 0.80 | / | | | |
| Group sessions | 5 | -0.01 | -0.25 to 0.23 | 0.90 | 0.0% | | | |
| Mixed | 11 | -0.32 | -0.70 to 0.06 | 0.09 | 87.0% | | | |
| **Training setting** | | | | | | | | |
| Face-to-face | 12 | -0.23 | -0.58 to 0.12 | 0.17 | 86.7% | | | |
| Virtual | 5 | -0.07 | -0.27 to 0.12 | 0.39 | 0.0% | | | |

| | | | | | | | | |
|---|---|---|---|---|---|---|---|---|
| Blended | 4 | -0.12 | -0.77 to 0.52 | 0.59 | 51.1% | | | |

*k* number of studies, *g* standardized mean difference scores, 95% CI (Hedges' g), *CI* confidence interval, *Q* Cochran's Q heterogeneity statistic to test moderator effects, *df* degrees of freedom of Cochran's Q

## 11.2 Subgroup analyses – Psychological stress

| | **k** | **g** | **95% CI** | **p** | **$I^2$** | **Q** | **df** | **$p_{subgroup}$** |
|---|---|---|---|---|---|---|---|---|
| **Risk of bias** | | | | | | 3.06 | 1 | 0.08 |
| Some concerns | 5 | -0.33 | -0.80 to 0.14 | 0.12 | 70.7% | | | |
| High | 6 | 0.23 | -0.47 to 0.94 | 0.43 | 91.6% | | | |
| **Study design** | | | | | | 0.03 | 1 | 0.87 |
| RCT | 7 | 0.01 | -0.66 to 0.67 | 0.98 | 93.4% | | | |
| CBA | 4 | -0.05 | -0.64 to 0.550 | 0.82 | 37.2% | | | |
| **Control group** | | | | | | 1.22 | 2 | 0.54 |
| Passive | 5 | -0.11 | -0.49 to 0.27 | 0.46 | | | | |
| Waitlist | 4 | 0.27 | -1.04 to 1.57 | 0.56 | | | | |
| Alternative | 2 | -0.40 | -6.47 to 5.66 | 0.55 | | | | |
| **Follow-up** | | | | | | 1.92 | 2 | 0.38 |
| Short-term | 5 | -0.31 | -0.79 to 0.17 | 0.15 | 65.3% | | | |
| Medium-term | 3 | 0.33 | -2.25 to 2.91 | 0.64 | 95.1% | | | |
| Long-term | 3 | -0.03 | 0.80 to 0.74 | 0.88 | 57.2% | | | |
| **Management level** | | | | | | 2.08 | 3 | 0.56 |
| Lower | 2 | 0.14 | -3.03 to 3.31 | 0.67 | 51.4% | | | |
| Middle | 5 | 0.08 | -1.02 to 1.18 | 0.85 | 95.4% | | | |
| Diverse | 3 | -0.20 | -0.55 to 0.15 | 0.13 | 0.0% | | | |
| NR | 1 | -0.14 | -0.60 to 0.33 | 0.56 | / | | | |
| **Higher proportion (>50%) of female participants** | | | | | | 4.91 | 2 | 0.09 |
| Yes | 3 | -0.40 | -1.53 to 0.72 | 0.26 | 75.5% | | | |
| No | 7 | 0.07 | -0.54 to 0.68 | 0.79 | 91.0% | | | |
| NR | 1 | 0.41 | -0.10 to 0.93 | 0.12 | / | | | |
| **Intervention type** | | | | | | 7.38 | 4 | 0.12 |
| Alternative to the others | 1 | 0.11 | -0.51 to 0.73 | 0.72 | / | | | |
| Organization-focused | 1 | 0.41 | -0.10 to 0.93 | 0.12 | / | | | |
| Multimodal | 3 | 0.33 | -2.25 to 2.91 | 0.64 | 95.1% | | | |
| Cognitive-Behavioral | 4 | -0.17 | -0.36 to -0.00 | 0.05 | 0.0% | | | |
| Relaxation | 2 | -0.59 | -5.04 to 3.86 | 0.34 | 59.2% | | | |
| Biofeedback | / | / | / | | / | | | |
| **No. of intervention type components** | | | | | | 1.99 | 2 | 0.37 |
| 1 | 8 | -0.18 | -0.49 to 0.19 | 0.21 | 67.2% | | | |
| 2 | 2 | 0.70 | -9.28 to 10.68 | 0.53 | 96.4% | | | |
| 3 | 1 | -0.47 | -1.12 to 0.18 | 0.16 | / | | | |
| **Intervention length** | | | | | | 7.75 | 4 | 0.10 |
| <1 week | 1 | 0.11 | -0.51 to 0.73 | 0.72 | / | | | |
| 1-4 weeks | / | / | / | | / | | | |
| 5-8 weeks | 4 | -0.40 | -0.98 to 0.18 | 0.12 | 64.0% | | | |
| 9-12 weeks | 3 | 0.33 | -2.25 to 2.91 | 0.64 | 95.1% | | | |
| >12 weeks | 2 | -0.17 | -1.16 to 0.82 | 0.27 | 0.0% | | | |
| NR | 1 | 0.41 | -0.10 to 0.93 | 0.12 | / | | | |
| **Delivery Mode** | | | | | | 0.66 | 2 | 0.72 |
| Self-practice | 1 | -0.26 | -0.72 to 0.20 | 0.27 | / | | | |
| Group sessions | 4 | -0.07 | -0.45 to 0.32 | 0.62 | 35.7% | | | |
| Mixed | 6 | 0.01 | -0.85 to 0.88 | 0.97 | 94.4% | | | |
| **Training setting** | | | | | | 0.67 | 2 | 0.72 |
| Face-to-face | 6 | 0.04 | -0.77 to 0.86 | 0.89 | 94.4% | | | |
| Virtual | 2 | -0.17 | -1.27 to 0.93 | 0.30 | 0.0% | | | |

| | | | | | | | | |
|---|---|---|---|---|---|---|---|---|
| Blended | 3 | -0.02 | -1.18 to 1.14 | 0.95 | 54.9% | | | |

*k* number of studies, *g* standardized mean difference scores, 95% CI (Hedges' g), *CI* confidence interval, *Q* Cochran's Q heterogeneity statistic to test moderator effects, *df* degrees of freedom of Cochran's Q

## 11.3 Subgroup analyses – Mental health

| | k | g | 95% CI | p | $I^2$ | Q | df | $p_{subgroup}$ |
|---|---|---|---|---|---|---|---|---|
| **Risk of bias** | | | | | | 0.98 | 1 | 0.32 |
| Some concerns | 6 | -0.25 | -0.71 to0.21 | 0.23 | 83.5% | | | |
| High | 6 | -0.52 | -1.06 to 0.02 | 0.06 | 83.3% | | | |
| **Study design** | | | | | | 1.01 | 1 | 0.3 |
| RCT | 10 | -0.42 | -0.79 to -0.04 | 0.03 | 85.5% | | | |
| CBA | 2 | -0.21 | -0.44 to 0.01 | 0.06 | 0.0% | | | |
| **Control group** | | | | | | 3.04 | 2 | 0.22 |
| Passive | 4 | -0.13 | -0.35 to 0.09 | 0.15 | 0.0% | | | |
| Waitlist | 6 | -0.48 | -1.06 to 0.11 | 0.09 | 85.7% | | | |
| Alternative | 2 | -0.64 | -6.92 to 5.65 | 0.42 | 83.9% | | | |
| **Follow-up** | | | | | | 3.78 | 2 | 0.15 |
| Short-term | 4 | -0.49 | -1.52 to 0.55 | 0.23 | 83.3% | | | |
| Medium-term | 6 | -0.43 | -0.91 to 0.05 | 0.07 | 86.3% | | | |
| Long-term | 2 | -0.08 | -1.23 to 1.06 | 0.53 | 0.0% | | | |
| **Management level** | | | | | | 6.26 | 3 | 0.10 |
| Lower | 1 | -0.26 | -0.71 to 0.19 | 0.26 | / | | | |
| Middle | 3 | -0.73 | -1.95 to 0.49 | 0.12 | 81.6% | | | |
| Diverse | 6 | -0.10 | -0.24 to 0.03 | 0.11 | 0.0% | | | |
| NR | 2 | -0.81 | -8.69 to 7.07 | 0.41 | 94.2% | | | |
| **Higher proportion (>50%) of female participants** | | | | | | 0.32 | 2 | 0.57 |
| Yes | 6 | -0.31 | -0.74 to 0.13 | 0.13 | 81.4% | | | |
| No | 6 | -0.47 | -1.06 to 0.13 | 0.10 | 86.1% | | | |
| **Intervention types** | | | | | | 13.04 | 3 | 0.00 |
| Alternative to the others | / | / | / | / | / | | | |
| Organization-focused | / | / | / | / | / | | | |
| Multimodal | 4 | -0.32 | -0.99 to 0.35 | 2.24 | 83.8% | | | |
| Cognitive-Behavioral | 6 | -0.34 | -0.88 to 0.20 | 1.69 | 80.1% | | | |
| Relaxation | 1 | -1.08 | -1.43 to -0.74 | 4.44 | / | | | |
| Biofeedback | 1 | -0.09 | -0.79 to 0.61 | 8.00 | / | | | |
| **No. of intervention type components** | | | | | | 0.87 | 2 | 0.65 |
| 1 | 8 | -0.42 | -0.86 to 0.03 | 0.06 | 84.5% | | | |
| 2 | 3 | -0.36 | -1.63 to 0.91 | 0.34 | 88.4% | | | |
| 3 | 1 | -0.21 | -0.47 to 0.04 | 0.11 | / | | | |
| **Intervention length** | | | | | | 6.56 | 3 | 0.09 |
| <1 week | / | / | / | / | / | | | |
| 1-4 weeks | / | / | / | / | / | | | |
| 5-8 weeks | 4 | -0.42 | -1.20 to 0.35 | 0.18 | 81.2% | | | |
| 9-12 weeks | 3 | -0.86 | -2.33 to 0.60 | 0.12 | 83.6% | | | |
| >12 weeks | 4 | -0.09 | -0.31 to 0.13 | 0.29 | 0.0% | | | |
| NR | 1 | -0.21 | -0.50 to 0.09 | 0.16 | / | | | |
| **Delivery Mode** | | | | | | 7.15 | 3 | 0.07 |
| Self-practice | 3 | -0.09 | -0.49 to 0.32 | 0.45 | 0.0% | | | |
| Individual sessions | 1 | -0.09 | -0.79 to 0.61 | 0.80 | / | | | |
| Group sessions | 3 | -0.11 | -0.44 to 0.21 | 0.26 | 0.0% | | | |
| Mixed | 5 | -0.76 | -1.43 to -0.09 | 0.03 | 87.7% | | | |
| **Training setting** | | | | | | 3.67 | 1 | 0.05 |
| Face-to-face | 8 | -0.51 | -0.97 to -0.06 | 0.03 | 86.7% | | | |
| Virtual | 4 | -0.12 | -0.37 to 0.14 | 0.24 | 0.0% | | | |

| | | | | | | | | |
|---|---|---|---|---|---|---|---|---|
| Blended | / | / | / | / | / | | | |

*k* number of studies, *g* standardized mean difference scores, 95% CI (Hedges' g), *CI* confidence interval, *Q* Cochran's Q heterogeneity statistic to test moderator effects, *df* degrees of freedom of Cochran's Q

# S12. Results of meta-regression

Meta-regression was performed, when more than 10 studies were pooled per analyzed variable.

## 12.1 Meta-regression – All analyzed outcomes

| | k | Estimate (se) | $p_{moderator}$ | $I^2$ | $p_{residual\ heterogeneity}$ |
|---|---|---|---|---|---|
| **Age** | 18 | -0.02 (0.03) | 0.57 | 74.92% | < 0.0001 |
| **%female** | 20 | 0.00 (0.00) | 0.52 | 78.12% | < 0.0001 |
| **Sample size** | 21 | -0.00 (0.00) | 0.77 | 78.87% | < 0.0001 |
| **Publication year** | 21 | -0.03 (0.02) | 0.24 | 77.02% | < 0.0001 |

*k* number of studies *se* standard error $I^2$ heterogeneity statistics

## 12.2 Meta-regression – Psychological Stress

| | k | Estimate (se) | $p_{moderator}$ | $I^2$ | $p_{residual\ heterogeneity}$ |
|---|---|---|---|---|---|
| **Age** | 10 | -0.02 (0.06) | 0.72 | 89.81% | < 0.0001 |
| **%female** | 10 | 0.00 (0.00) | 0.90 | 89.11% | < 0.0001 |
| **Sample size** | 11 | 0.00 (0.00) | 0.85 | 88.46% | < 0.0001 |
| **Publication year** | 11 | 0.03 (0.05) | 0.64 | 88.21% | < 0.0001 |

*k* number of studies *se* standard error $I^2$ heterogeneity statistics

## 12.3 Meta-regression – Mental health

| | k | Estimate (se) | $p_{moderator}$ | $I^2$ | $p_{residual\ heterogeneity}$ |
|---|---|---|---|---|---|
| **Age** | 10 | 0.02 (0.07) | 0.81 | 87.49% | < 0.0001 |
| **%female** | 12 | 0.00 (0.00) | 0.73 | 85.74% | < 0.0001 |
| **Sample size** | 12 | 0.00 (0.00) | 0.68 | 85.53% | < 0.0001 |
| **Publication year** | 12 | -0.04 (0.03) | 0.26 | 84.10% | < 0.0001 |

*k* number of studies *se* standard error $I^2$ heterogeneity statistics

# S13. Funnel plots for detection of publication bias

## 13.1 All analyzed interventions

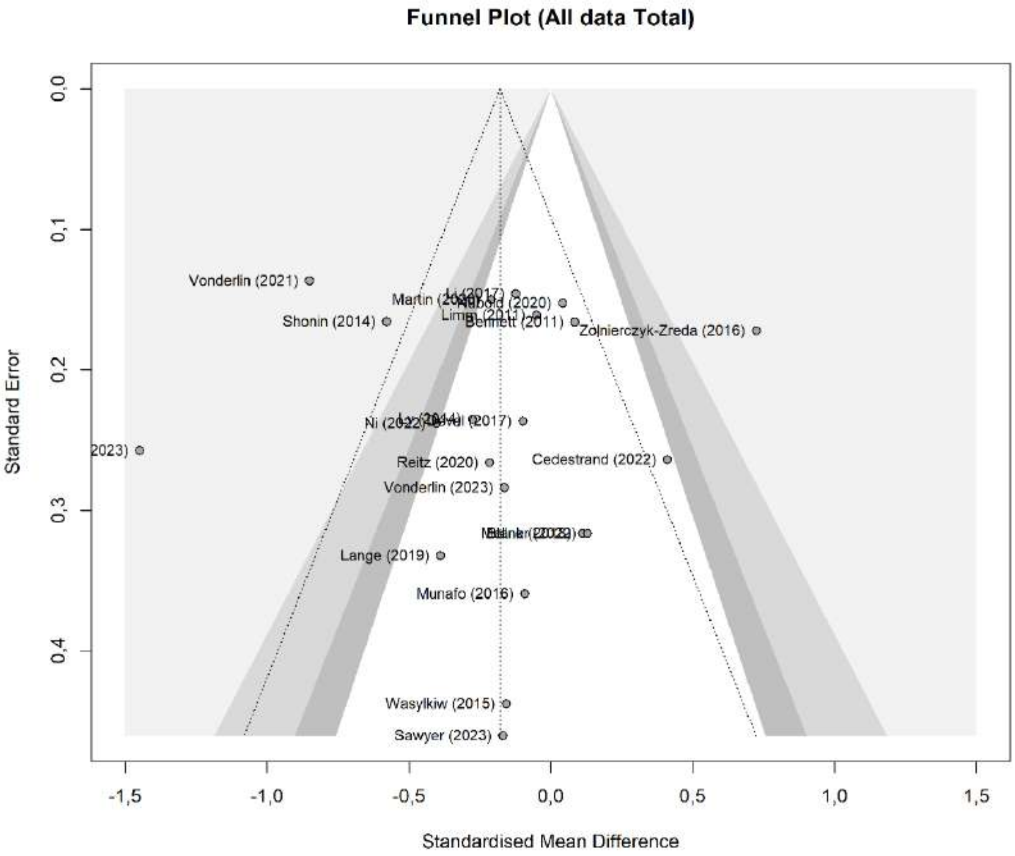

## 13.2 Psychological stress

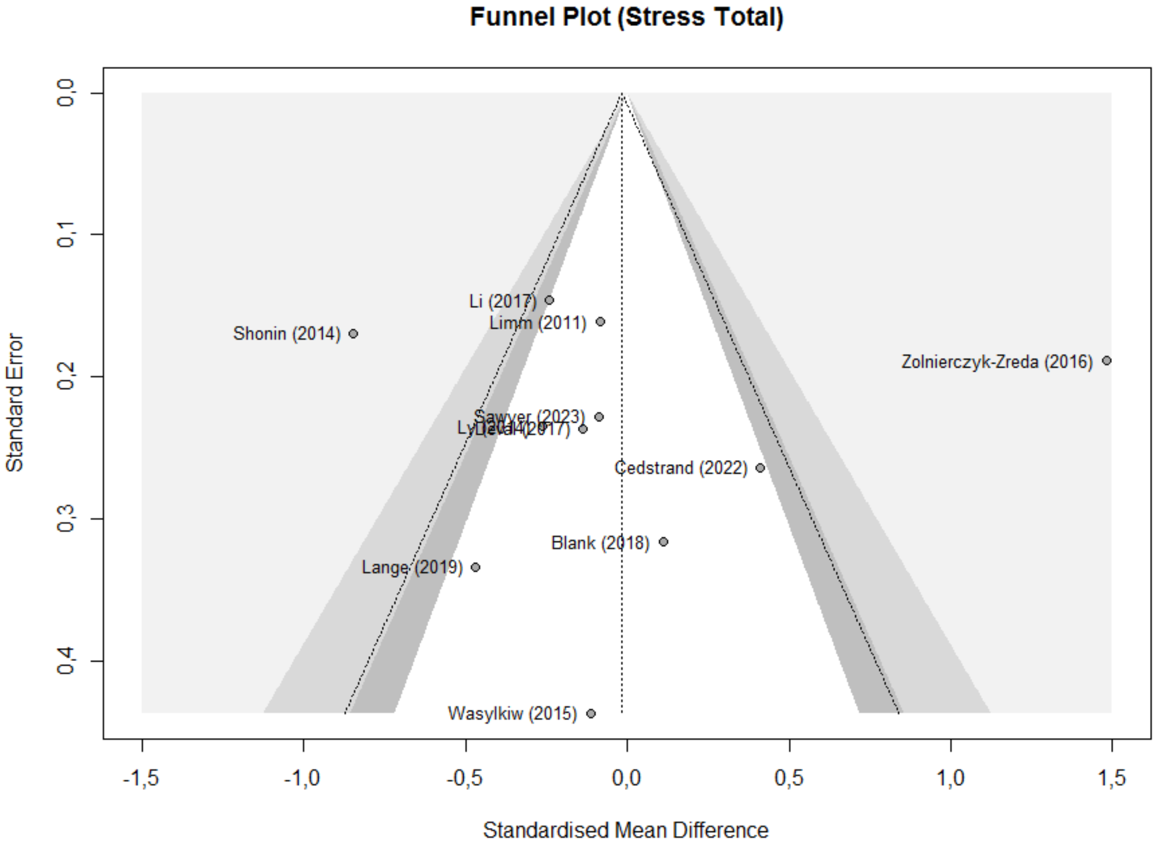

## 13.3 Perceived Stress

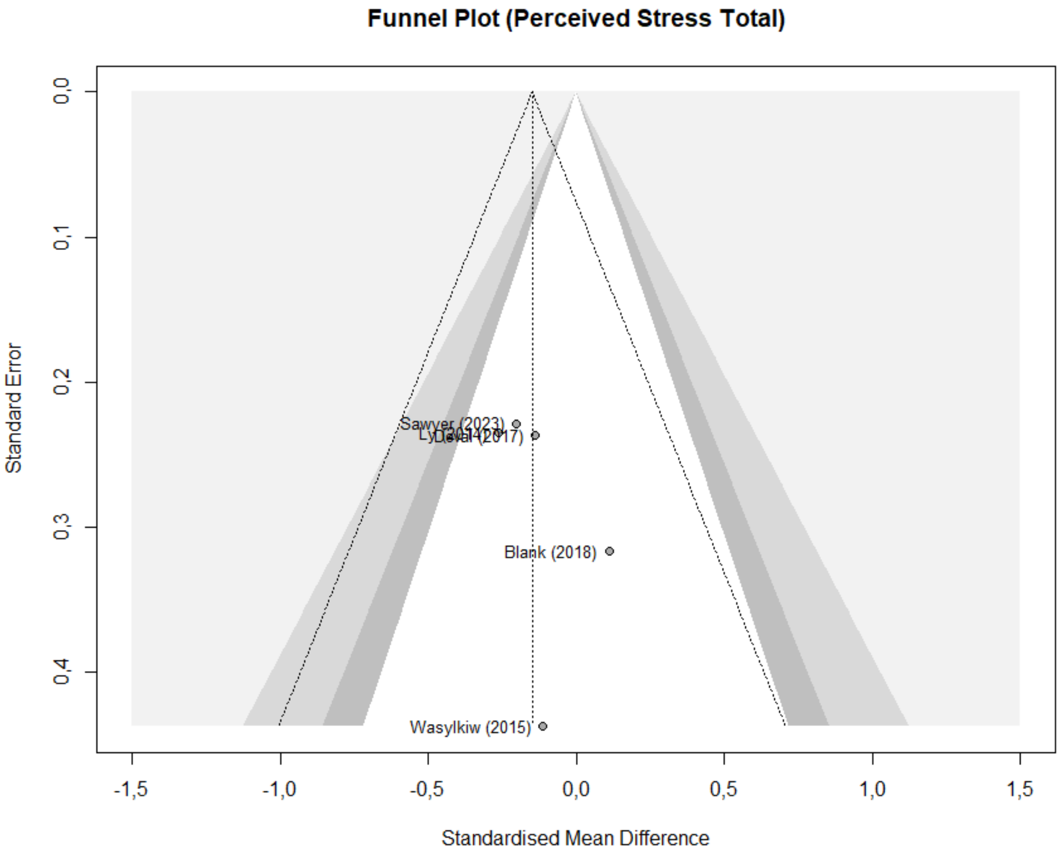

## 13.4 Job stress

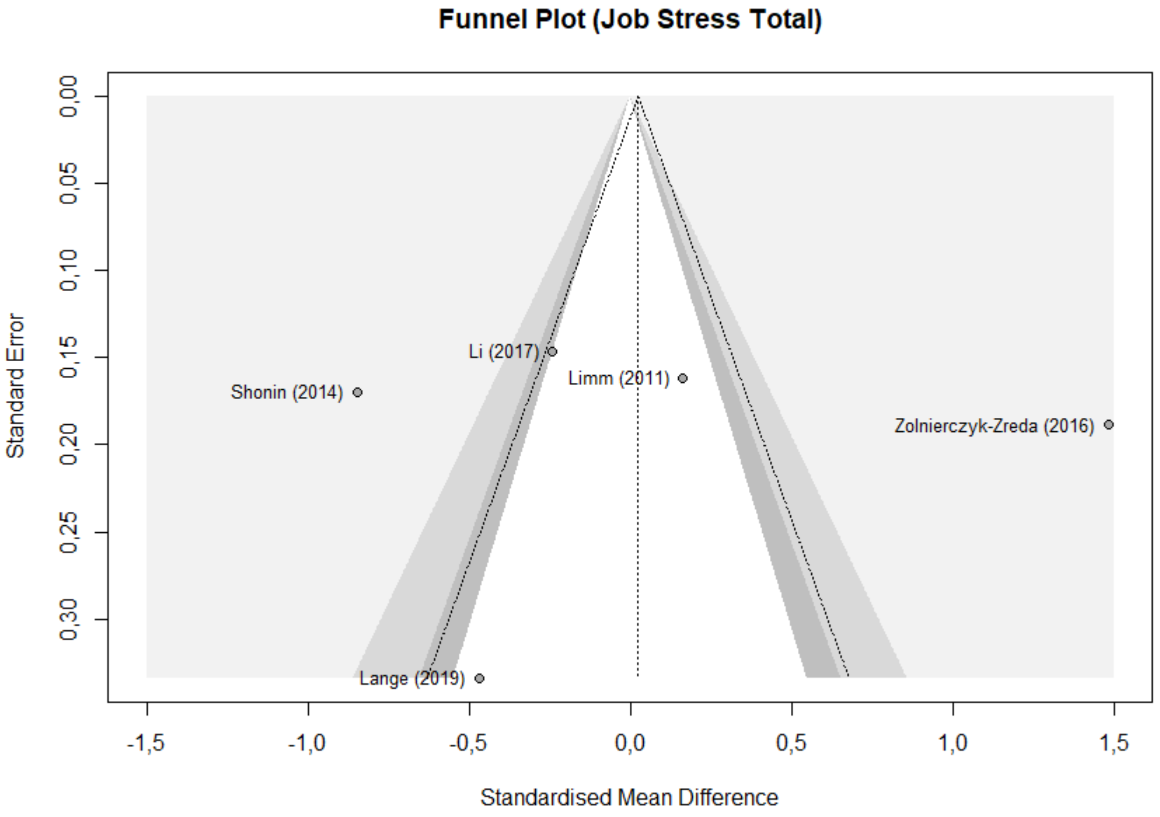

## 13.5 Mindfulness

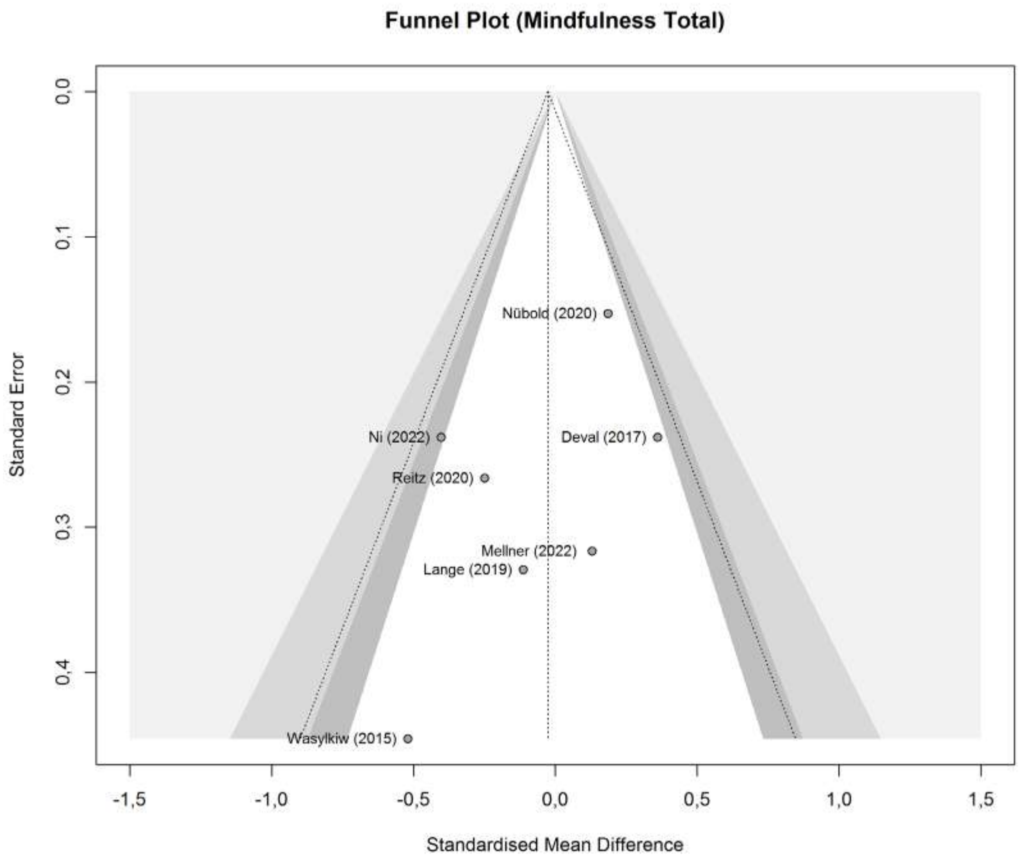

## 13.6 Mental health

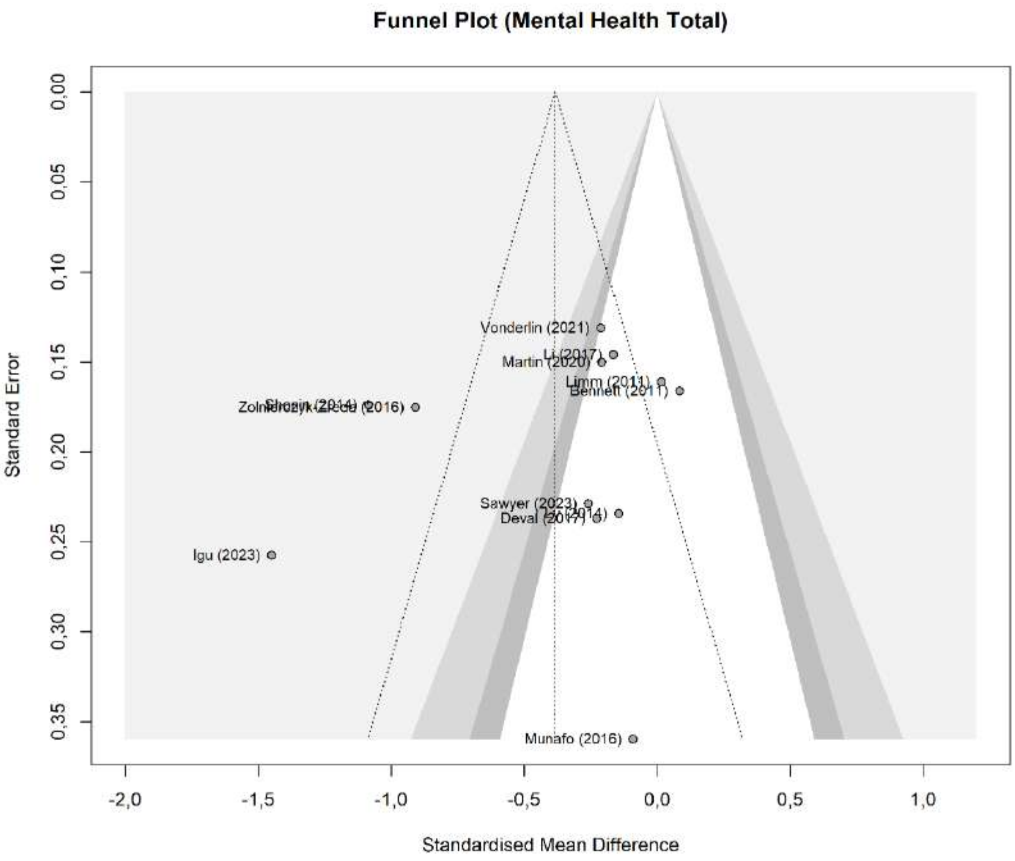

## 13.7 Subsyndromal symptoms

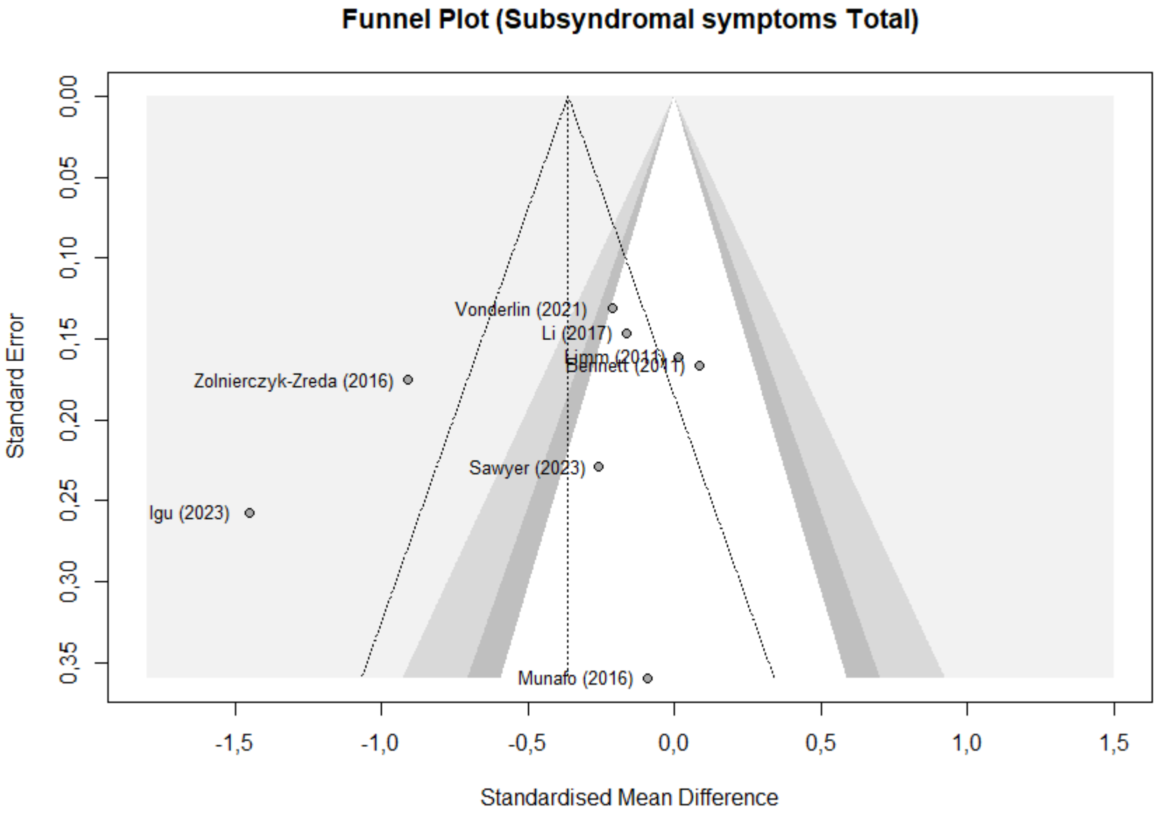

## 13.8 General mental health

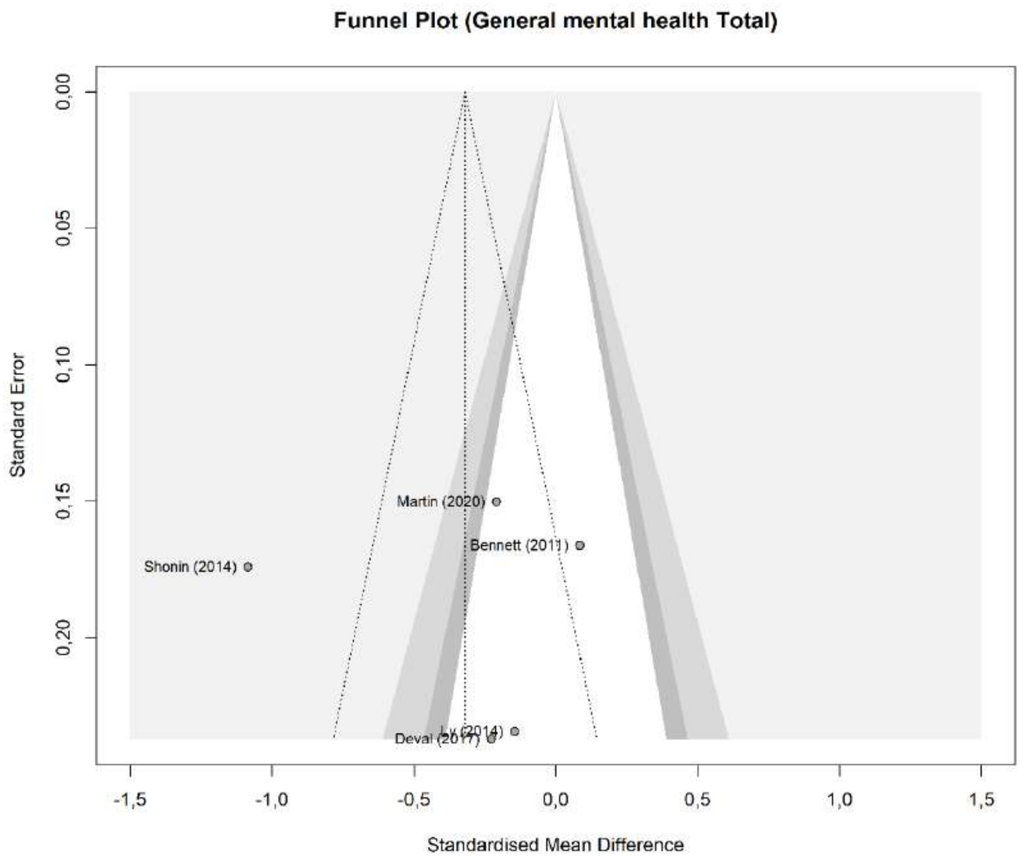

## 13.9 Work-related outcomes

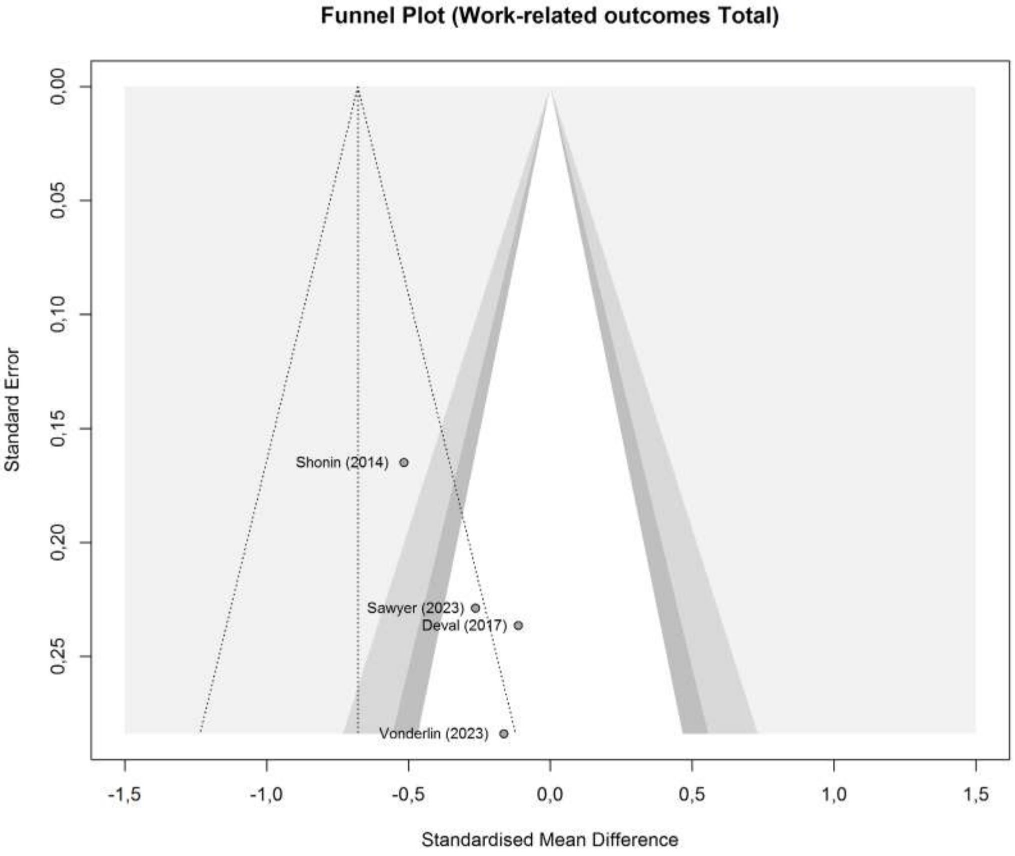

## 13.10 Leadership-related outcomes

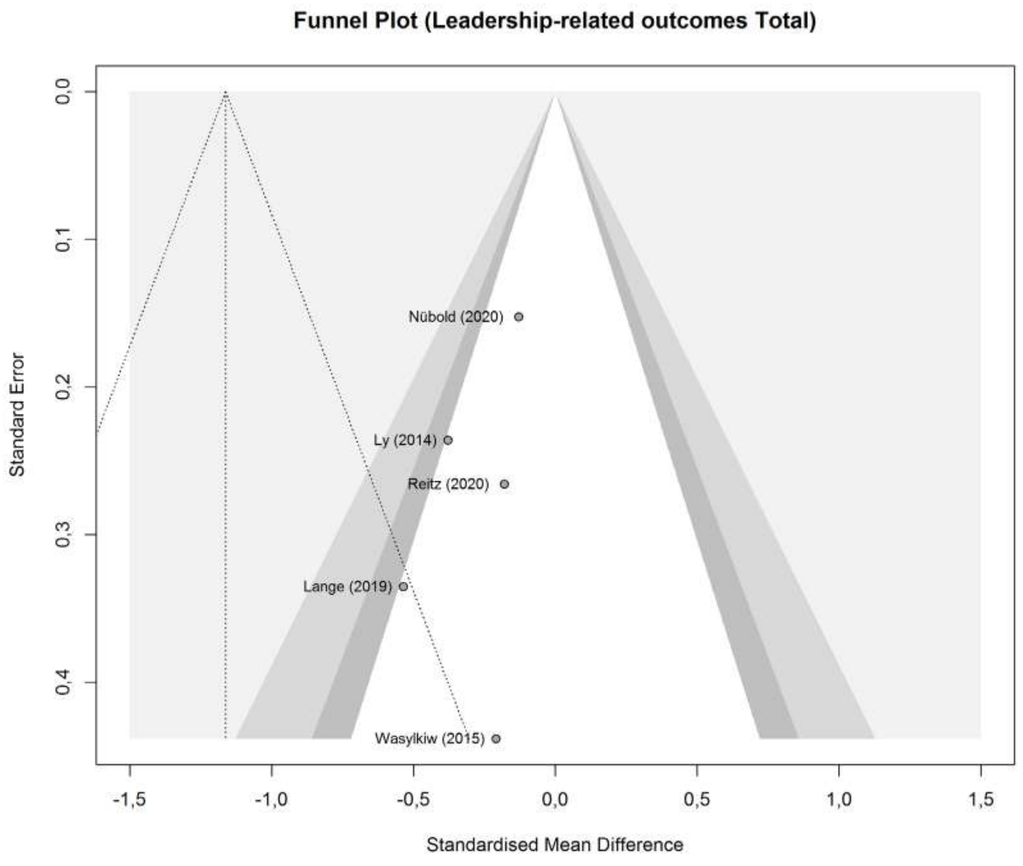

# S14. Eggers´ test

When a meta-analysis included ≥10 studies, we applied the Egger´s test (114).

| Outcome domain | intercept | 95% CI | t | p | Decision |
|---|---|---|---|---|---|
| All analyzed outcomes (k=21) | 0.32 | -2.59 to 3.22 | 0.21 | 0.83 | Eggers' test does not indicate the presence of funnel plot asymmetry. |
| Psychological stress (k=11) | 0.74 | -5.85 to 7.33 | 0.22 | 0.83 | Eggers' test does not indicate the presence of funnel plot asymmetry. |
| Mental health (k=12) | -2.18 | -7.75 to -3.9 | -77 | 0.46 | Eggers' test does not indicate the presence of funnel plot asymmetry. |

# S15. GRADE evidence profile

| | | *Reasons for considering lowering certainty* | | | | | | Reasons for lowering certainty | | | |
|---|---|---|---|---|---|---|---|---|---|---|---|
| Outcome | Number of studies | Risk of bias | Inconsistency | Indirectness | Imprecision | Publication bias | **AFTER DOWNGRADE** Certainty of evidence | Large effect | Dose response | All plausible confounding and bias | **AFTER UPGRADE** Certainty of evidence |
| *Main level outcome categories* | | | | | | | | | | | |
| Psychological stress<br><br>All studies: -0.02 (95% CI, -0.43 to 0.39)<br>Without outliers (k=9): -0.12 (95% CI, -0.28 to 0.04) | 11 studies (36,39,42,57,63,68,69,85,89,99,109) | We downgrade by -0.5 as sensitivity analyses reveal a small contradictory effect size between studies with some concerns RoB and those with high RoB. The proportion of high RoB studies (55%) and | We downgrade by -1 because of considerable heterogeneity indicated by I^2=89% and the 95% PI= -1.39 to 1.35 | - | We downgrade by -0.5 because of a moderate 95% confidence interval (-0.63 to -0.07). When outliners are excluded 95% confidence interval narrows (-0.28 to 0.04). Broad | - | **LOW** | - | - | - | **LOW** |

| | | | | | | | | | | | |
|---|---|---|---|---|---|---|---|---|---|---|---|
| | | studies with some concerns RoB (45%) was nearly equal. | | | sample size (IG: n=549; CG: n=491) | | | | | | |
| Mindfulness<br><br>All studies: -0.03 (95% CI, -0.32 to 0.27)<br>No outliers | 7 studies (42,57,76,80,82,84,99) | We downgrade by -1 because five studies (71%) had a high RoB, and there were contradictory effect sizes between studies with high RoB and those with some concerns RoB. | I²=34%; PI= -0.62 to 0.57 | - | We downgrade by -1 because of a moderate confidence interval (-0.80 to 0.07) and a low number of participants (IG: n= 259; CG: n= 221). | - | **LOW** | - | - | - | **LOW** |
| Mental health<br><br>All studies: -0.38 (95% CI, -0.69 to -0.08)*<br>Without outliers (k=10): -0.21 (95% CI, -0.41 to -0.02)* | 12 studies (34,42,54,63,68,69,72,78,85,89, 98,109) | We downgrade by -0.5 due to an equal proportion of high RoB and some concerns RoB studies, the absence of contradicto | We downgrade by -1 because of considerable heterogeneity indicated by I²=83% and the 95% PI= -1.38 to 0.62 | - | We downgrade by -0.5 because of a moderate confidence interval (-0.69 to -0.08). When outliers are excluded, | - | **LOW** | - | - | - | **LOW** |

| | | | | | | | | | | | |
|---|---|---|---|---|---|---|---|---|---|---|---|
| | | ry effect sizes between high RoB studies and those with some concerns RoB as well as a broad population sample. | | | confidence interval narrows and gets significant (-0.41 to -0.02). | | | | | | |
| Work-related outcomes<br><br>All studies: -0.68 (95% CI, -1.84 to 0.48)<br>Without outliers (k=4): -0.32 (95% CI, -0.63 to -0.00)* | 5 studies (42,85,89,98,109) | We downgrade by -1 because 4 studies (66%) had a high RoB, and there were broad 95% confidence intervals in high RoB studies (-3.96 to 2.16) and studies with some concerns RoB (-2.44 to 1.61). | We downgrade by -1 because of considerable heterogeneity indicated by I²=94% and the 95% PI= -3.85 to 2.49 | - | We downgrade by -1 because of a broad confidence interval (-1.84 to 0.48). Confidence interval narrows and gets significant when outliers are excluded (-0.63 to -0.00). Furthermore, there is a low number of particpants (IG: n=253; CG: n=482). | We downgrade by -1 because of funnel plot asymmetry. | **VERY LOW** | - | - | - | **VERY LOW** |

| | | | | | | | | | | | |
|---|---|---|---|---|---|---|---|---|---|---|---|
| Leadership-related outcomes<br><br>All studies: -1.16 (95% CI, -3.42 to 1.10)<br>Without outliers (k=5): -0.23 (95% CI, -0.44 to -0.02)* | 6 studies (57,69,82,84,94,99) | We downgrade by -1 because of 3 studies (50%) had a high RoB and broad 95% confidence intervals by some concerns RoB studies (-9.45 to 5.15). | We downgrade by -1 because of considerable heterogeneity indicated by I²= 98% and the 95% PI= -7.57 to 5.24 | - | We downgrade by -1 because of a broad confidence interval (-3.42 to 1.10). Confidence interval narrows when outliers are excluded (-0.44 to ,0.02). Furthermore, there is a low number of participants (IG: n=303; CG:n=292 ). | We downgrade by -1 because of funnel plot asymmetry. | **VERY LOW** | - | - | - | **VERY LOW** |
| *Subcategories (emerged from main outcome categories)* | | | | | | | | | | | |
| Psychological stress: Perceived stress<br><br>All studies: -0.15 (95% CI, -0.38 to 0.08)<br>No outliers | 5 studies (36,42,69,85,99) | We downgrade by -1 because three studies (60%) had a high RoB and broad 95% confidence interval in some concerns | I²=0%; PI= -0.53 to 0.23 | - | We downgrade by -0.5 because of a low number of participants (IG: n= 159; CG: n=132) | We downgrade by -0.5 because visual inspection of the funnel plot suggests the possibility of a | **LOW** | - | - | - | **LOW** |

| | | | | | | | | | | | | |
|---|---|---|---|---|---|---|---|---|---|---|---|---|
| | | RoB (-2.40 to 2.14) | | | | missing study with a high standard error and a negative effect. | | | | | | |
| Psychological stress: Job stress<br><br>All studies: 0.02 (95% CI, -1.09 to 1.14)<br>No outliers | 5 studies (57,63,68,89,109) | We downgrade by -1.5 due to two studies (40%) indicating a high risk of bias, and sensitivity analyses revealed a contradictory effect size between studies with some concerns RoB versus those with high RoB. Additionally, the 95% confidence interval was notably wide in high RoB studies (- | We downgrade by -1 because of considerable heterogeneity indicated by $I^2$=96% and the 95% PI= -3,05 to 3,10 | - | We downgrade by -0,5 because of a moderate 95% confidence interval (-1,09 to 1,14). | - | **VERY LOW** | - | - | - | | **VERY LOW** |

| | | | | | | | | | | | |
|---|---|---|---|---|---|---|---|---|---|---|---|
| | | 10,33 to 11,5). | | | | | | | | | |
| Mental health: Subsyndromal symptoms<br><br>All studies: -0,36 (95% CI, -0,80 to 0,07)<br>Without outliers (k=7): -0,22 (-0,53 to 0,09) | 8 studies (34,54,63,68,78,85,94,109) | We downgrade by -0.5 due to an equal proportion of high RoB studies and studies with some concerns RoB, as well as the absence of contradictory effect sizes between high RoB studies and those with some concerns RoB. | We downgrade by -1 because of considerable heterogeneity indicated by I²=84% and the 95% PI= -1.59 to 0.87 | - | We downgrade by -0.5 because of a moderate confidence interval (-0.80 to 0.07). Confidence interval narrows when outliers are excluded (-0.53 to 0.09). | - | **LOW** | - | - | - | **LOW** |
| Mental health: General mental health<br><br>All studies: -0.32 (95% CI, -0.88 to 0.24)<br>No outliers | 5 studies (34,42,69,72,89) | We downgraded by -0.5 due to an almost equal proportion of high RoB studies and studies with some concerns RoB, as | We downgrade by -1 because of considerable heterogeneity indicated by I²=85% and the 95% PI= -1.81 to 1.17 | - | We downgrade by -0.5 because of a moderate confidence interval (-0.88 to 0.24). | - | **LOW** | - | - | - | **LOW** |

| | | | | | | | | | | | |
|---|---|---|---|---|---|---|---|---|---|---|---|
| | | well as the absence of contradictory effect sizes between high RoB studies and those with some concerns RoB. | | | | | | | | | |

# S16. Traffic-light plots of the domain-level judgements for each individual result

## 16.1 Randomized controlled trials

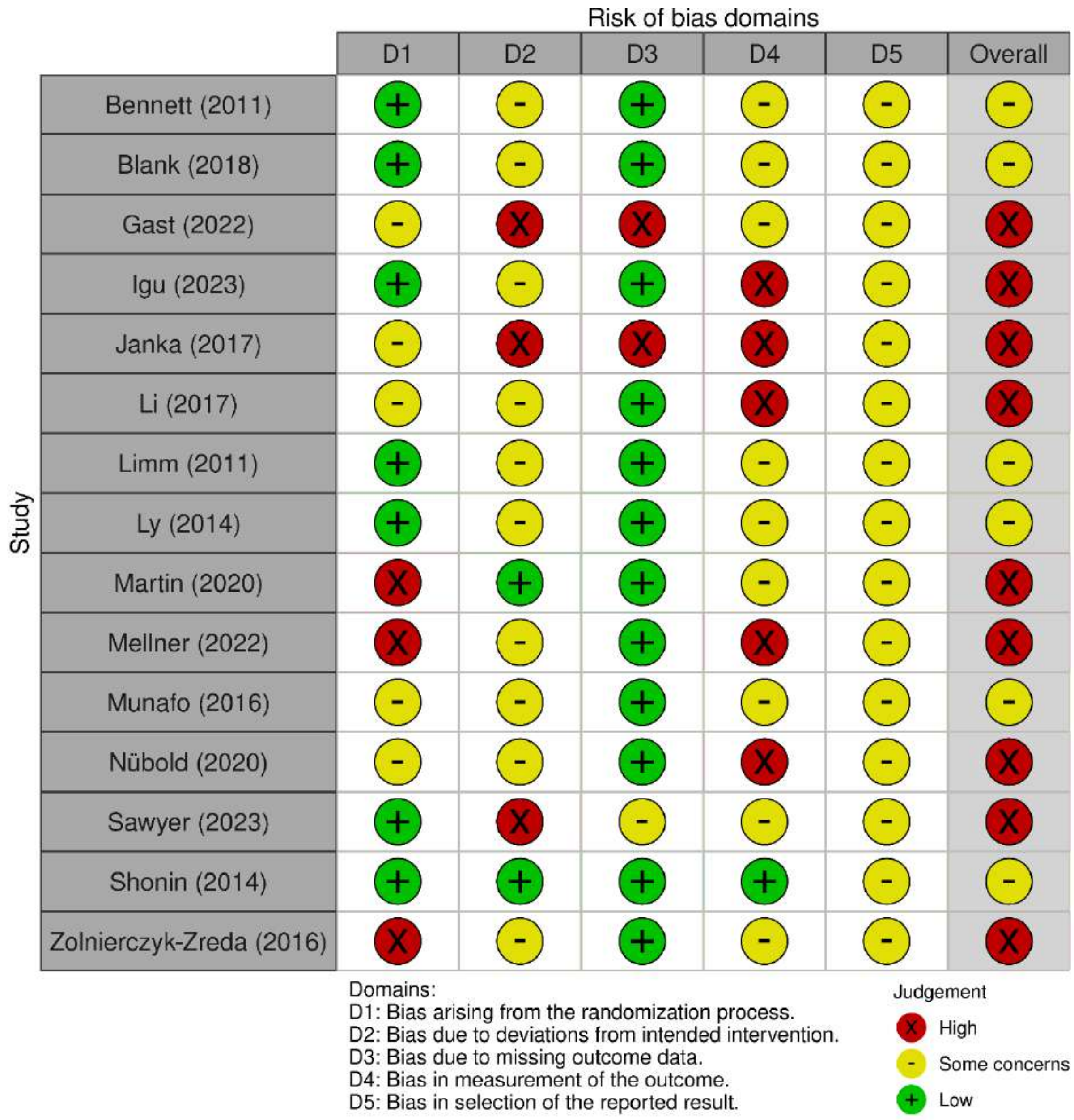


Figure was created using the tool robvis (115).

## 16.2 Controlled before–after studies

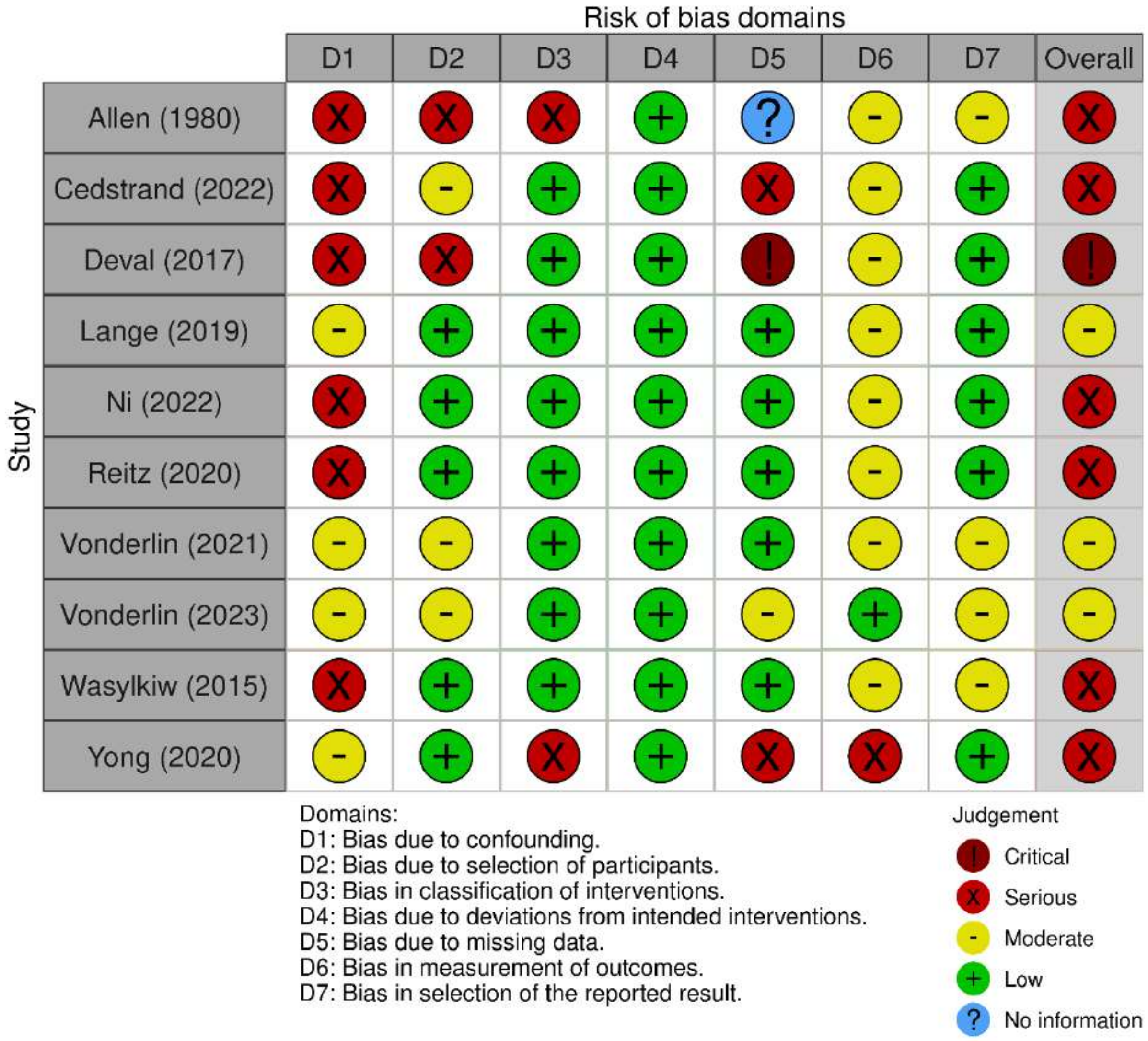


Figure was created using the tool robvis (115).

# S17. Weighted-bar plots of risk of bias judgements within each bias domain

## 17.1 Randomized controlled trials

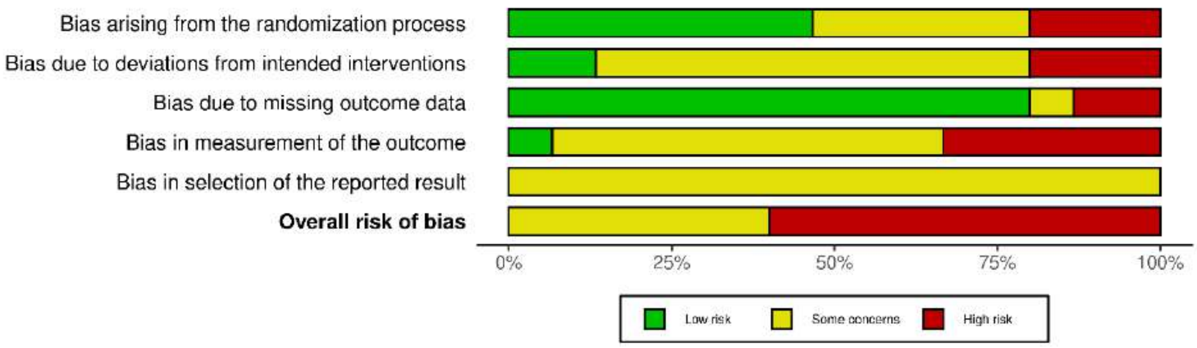


Figure was created using the tool robvis (115).

## 17.2 Controlled before–after studies

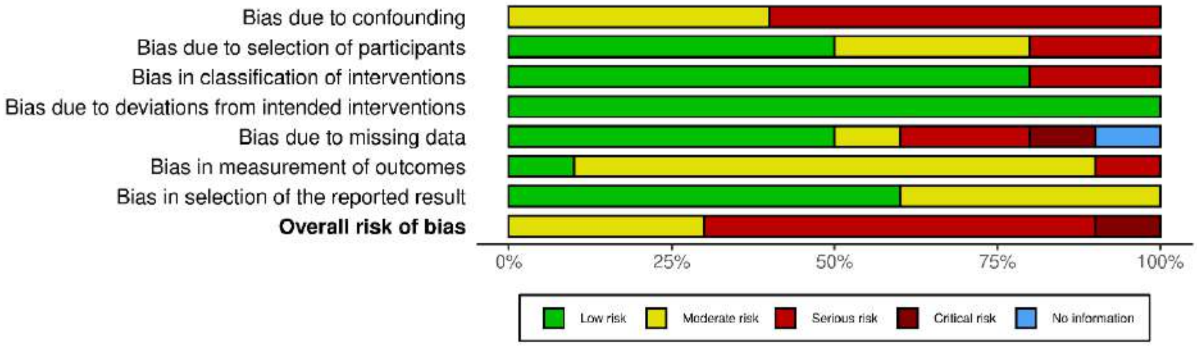


Figure was created using the tool robvis (115).

# S18. References

1. Balint EM, Angerer P, Guendel H, Marten-Mittag B, Jarczok MN. Stress Management Intervention for Leaders Increases Nighttime SDANN: results from a Randomized Controlled Trial. IJERPH 2022;19(7).
2. Bernardez B, Panach JI, Parejo JA, Duran A, Juristo N, Ruiz-Cortes A. An empirical study to evaluate the impact of mindfulness on helpdesk employees. SCIENCE OF COMPUTER PROGRAMMING 2023;230.
3. Biggs Amanda, Brough Paula, Barbour Jennifer P. Enhancing work-related attitudes and work engagement: A quasi-experimental study of the impact of an organizational intervention. Theory and Methods to Prevent and Manage Occupational Stress: Innovations from Around the Globe 2014;21(1):43–68.
4. Born M, Küllenberg J, Drews A, Bossmann U, Zwack J, Gündel H, et al. The effects of a dilemma management training program on mental health: a prospective study with mid-level executives in hospitals. Leadership in health services (Bradford, England) 2022;ahead-of-print(ahead-of-print).
5. Carlisle Thomas William. Effects of the transcendental meditation program on Psychological, health, social, and behavioral indicators of stress reduction and Human Resource development in the indian workplace: Dissertation Abstracts International Section A: Humanities and Social Sciences; 2005.
6. Coetzer Michiel Frederick. The impact of a servant leadership intervention on work engagement and burnout: Dissertation Abstracts International Section A: Humanities and Social Sciences; 2021.
7. Dalgaard VL, Gayed A, Hansen AKL, Grytnes R, Nielsen K, Kirkegaard T, et al. A study protocol outlining the development and evaluation of a training program for frontline managers on leading well-being and the psychosocial work environment in Danish hospital settings - a cluster randomized waitlist controlled trial. BMC Public Health 2023;23(1):848. PubMed PMID: 37165331; PubMed Central PMCID: PMC10170839.
8. DRKS00023457. Evaluation of a stress management intervention for leaders in small to medium sized companies. https://trialsearch.who.int/Trial2.aspx?TrialID=DRKS00023457 [Internet] 2020. Available from: https://www.cochranelibrary.com/central/doi/10.1002/central/CN-02239658/full.
9. Lehmann JAM, Schwarz E, Rahmani Azad Z, Gritzka S, Seifried-Dübon T, Diebig M, et al. Effectiveness and cost effectiveness of a stress management training for leaders of small and medium sized enterprises - study protocol for a randomized controlled-trial. BMC Public Health 2021;21(1):468. PubMed PMID: 33685418; PubMed Central PMCID: PMC7938293.
10. Grant Anthony M, Curtayne Linley, Burton Geraldine. Executive coaching enhances goal attainment, resilience and workplace well-being: A randomised controlled study. The Journal of Positive Psychology 2009;4(5):396–407.
11. Gray NS, Davies H, Brad R, Snowden RJ. Reducing sickness absence and stigma due to mental health difficulties: a randomised control treatment trial (RCT) of a low intensity psychological intervention and stigma reduction programme for common mental disorder (Prevail). BMC Public Health 2023;23(1):1324.
12. Harenstam A, Pousette A, Berntson E. Improving organizational and working conditions for managers in the Swedish public sector: A conceptual model and evaluation of interventions. ECONOMIC AND INDUSTRIAL DEMOCRACY 2022;43(1):72–97.

13. Iodice P, Cannito L, Chaigneau A, Palumbo R. Learned self-regulation in top-level managers through neurobiofeedback training improves decision making under stress. Scientific reports 2022;12(1):6127.
14. Arensman E, Leduc M, O'Brien C, Corcoran P, Griffin E, Leduc C, et al. Implementation and evaluation of a multi-level mental health promotion intervention for the workplace (MENTUPP): study protocol for a cluster randomised controlled trial. Trials 2023;24(1):621.
15. Jeon YH, Simpson JM, Li Z, Cunich MM, Thomas TH, Chenoweth L, et al. Cluster Randomized Controlled Trial of An Aged Care Specific Leadership and Management Program to Improve Work Environment, Staff Turnover, and Care Quality. Journal of the American Medical Directors Association 2015;16(7):629.e19-28.
16. Kawakami N, Takao S, Kobayashi Y, Tsutsumi A. Effects of web-based supervisor training on job stressors and psychological distress among workers: a workplace-based randomized controlled trial. J Occup Health 2006;48(1):28–34.
17. Kersemaekers WM, Vreeling K, Verweij H, van der Drift M, Cillessen L, van Dierendonck D, et al. Effectiveness and feasibility of a mindful leadership course for medical specialists: a pilot study. BMC Med Educ 2020;20(1):34.
18. MacPhee M, Dahinten VS, Hejazi S, Laschinger H, Kazanjian A, McCutcheon A, et al. Testing the effects of an empowerment-based leadership development programme: part 1-leader outcomes. J Nurs Manag 2014;22(1):4–15.
19. Maddi Salvatore R, Kahn Stephen, Maddi Karen L. The effectiveness of hardiness training. Consulting Psychology Journal: Practice and Research 1998;50(2):78–86.
20. NCT04727255. Engaged and Resilient - a Preventive Intervention to Promote Psychological Well-being and Mental Health for Leaders. https://clinicaltrials.gov/show/NCT04727255 [Internet] 2021. Available from: https://www.cochranelibrary.com/central/doi/10.1002/central/CN-02234485/full.
21. NCT05154019. Managing Minds at Work: a Feasibility Pilot Trial. https://clinicaltrials.gov/show/NCT05154019 [Internet] 2021. Available from: https://www.cochranelibrary.com/central/doi/10.1002/central/CN-02353720/full.
22. Nielsen K, Daniels K. Enhancing team leaders' well-being states and challenge experiences during organizational change: A randomized, controlled study. Human Relations 2012;65(9):1207–31.
23. Nishiuchi K, Tsutsumi A, Takao S, Mineyama S, Kawakami N. Effects of an education program for stress reduction on supervisor knowledge, attitudes, and behavior in the workplace: a randomized controlled trial. J Occup Health 2007;49(3):190–8.
24. Pipe TB, Bortz JJ, Dueck A, Pendergast D, Buchda V, Summers J. Nurse leader mindfulness meditation program for stress management: a randomized controlled trial. Journal of nursing administration 2009;39(3):130–7.
25. Sreekumar TS, Nagendra HR, Ilavarasu JV. Effect of yoga intervention on mindfulness, perceived stress, emotion regulation and affect: a study on senior managers in an Indian multinational corporate. International Journal of Indian Culture and Business Management 2021;22(1):37–52.
26. Tan NA, Peters EK, Reb J. Effects of a Mindfulness-Based Leadership Training on Leadership Behaviors and Effectiveness. Mindfulness 2023.
27. Zheng Yuan, Gu Xiaodan, Jiang Mingyan, Zeng Xianglong. How might mindfulness-based interventions reduce job burnout? Testing a potential self-regulation model with a randomized controlled trial. Mindfulness 2022;13(8):1907–22.
28. Zimber A, Rudolf A, Teufel S. A training program to reduce distress among geriatric caregivers. Z Gerontol Geriatr 2001;34(5):401–7.

29. De Armond David Lee. Effects of the Transcendental Meditation(rtm) program on psychological, physiological, behavioral and organizational consequences of stress in managers and executives: Dissertation Abstracts International: Section B: The Sciences and Engineering; 1996.

30. Allen JK, Blanchard EB. Biofeedback-based stress management training with a population of business managers. Biofeedback and Self-Regulation 1980;5(4):427–38. PubMed PMID: 7213824.

31. Schar M, Reeder LG, Dirken JM. Stress and cardiovascular health: an international cooperative study. II. The male population of a factory at Zurich. Soc Sci Med (1967) 1973;7(8):585–603. PubMed PMID: 4731469.

32. Spielberger CD, Gorusch R, Lushene R. Manual for the state-trait anxiety inventory. Palo Alto: Consulting Psychologists Press; 1970.

33. Peters RK, Benson H, Peters JM. Daily relaxation response breaks in a working population: II. Effects on blood pressure. Am J Public Health 1977;67(10):954–9. PubMed PMID: 333958; PubMed Central PMCID: PMC1653726.

34. Bennett JB, Broome KM, Schwab-Pilley A, Gilmore P. A web-based approach to address cardiovascular risks in managers: results of a randomized trial. Journal of Occupational and Environmental Medicine 2011;53(8):911–8. PubMed PMID: 21785368; PubMed Central PMCID: PMC3160446.

35. Cook RF, Billings DW, Hersch RK, Back AS, Hendrickson A. A field test of a web-based workplace health promotion program to improve dietary practices, reduce stress, and increase physical activity: randomized controlled trial. J Med Internet Res 2007;9(2):e17. PubMed PMID: 17581811; PubMed Central PMCID: PMC1913939.

36. Blank C, Gatterer K, Leichtfried V, Pollhammer D, Mair-Raggautz M, Duschek S, et al. Short Vacation Improves Stress-Level and Well-Being in German-Speaking Middle-Managers-A Randomized Controlled Trial. Int J Environ Res Public Health 2018;15(1). PubMed PMID: 29342844; PubMed Central PMCID: PMC5800229.

37. Fliege H, Rose M, Arck P, Walter OB, Kocalevent R-D, Weber C, et al. The Perceived Stress Questionnaire (PSQ) reconsidered: validation and reference values from different clinical and healthy adult samples. Psychosom Med 2005;67(1):78–88. PubMed PMID: 15673628.

38. Levenstein S, Prantera C, Varvo V, Scribano ML, Berto E, Luzi C, et al. Development of the Perceived Stress Questionnaire: a new tool for psychosomatic research. J Psychosom Res 1993;37(1):19–32. PubMed PMID: 8421257.

39. Cedstrand E, Augustsson H, Alderling M, Sánchez Martinez N, Bodin T, Nyberg A, et al. Effects of a co-created occupational health intervention on stress and psychosocial working conditions within the construction industry: A controlled trial. Front Public Health 2022;10:973890. PubMed PMID: 36211695; PubMed Central PMCID: PMC9542354.

40. Burr H, Berthelsen H, Moncada S, Nübling M, Dupret E, Demiral Y, et al. The Third Version of the Copenhagen Psychosocial Questionnaire. Saf Health Work 2019;10(4):482–503. PubMed PMID: 31890332; PubMed Central PMCID: PMC6933167.

41. Berthelsen H, Westerlund H, Bergström G, Burr H. Validation of the Copenhagen Psychosocial Questionnaire Version III and Establishment of Benchmarks for Psychosocial Risk Management in Sweden. Int J Environ Res Public Health 2020;17(9). PubMed PMID: 32370228; PubMed Central PMCID: PMC7246423.

42. Deval C, Bernard-Curie S, Monestès J-L. Effects of an acceptance and commitment therapy intervention on leaders' and managers' psychological flexibility. Journal de Thérapie Comportementale et Cognitive 2017;27(1):34–42.

43. Bellinghausen L, Collange J, Botella M, Emery J-L, Albert É. Validation factorielle de l'échelle française de stress perçu en milieu professionnel. Santé Publique 2009;Vol. 21(4):365–73.

44. Jermann F, Billieux J, Larøi F, d'Argembeau A, Bondolfi G, Zermatten A, et al. Mindful Attention Awareness Scale (MAAS): Psychometric properties of the French translation and exploration of its relations with emotion regulation strategies. Psychol Assess 2009;21(4):506–14. PubMed PMID: 19947785.

45. Salama-Younes M, Montazeri A, Ismaïl A, Roncin C. Factor structure and internal consistency of the 12-item General Health Questionnaire (GHQ-12) and the Subjective Vitality Scale (VS), and the relationship between them: a study from France. Health Qual Life Outcomes 2009;7:22. PubMed PMID: 19265516; PubMed Central PMCID: PMC2653033.

46. Warr P, Cook J, Wall T. Scales for the measurement of some work attitudes and aspects of psychological well-being. Journal of Occupational Psychology 1979;52(2):129–48.

47. Diener E, Emmons RA, Larsen RJ, Griffin S. The Satisfaction With Life Scale. J Pers Assess 1985;49(1):71–5. PubMed PMID: 16367493.

48. Gast M, Lehmann J, Schwarz E, Hirning C, Hoelzer M, Guendel H, et al. A Single-Day Training for Managers Reduces Cognitive Stigma Regarding Mental Health Problems: A Randomized Trial. Int J Environ Res Public Health 2022;19(7). PubMed PMID: 35409821; PubMed Central PMCID: PMC8998400.

49. Warttig SL, Forshaw MJ, South J, White AK. New, normative, English-sample data for the Short Form Perceived Stress Scale (PSS-4). J Health Psychol 2013;18(12):1617–28. PubMed PMID: 24155195.

50. Cohen S, Kamarck T, Mermelstein R. A Global Measure of Perceived Stress. Journal of Health and Social Behavior 1983;24(4):385.

51. Rödel A, Siegrist J, Hessel A, Brähler E. Fragebogen zur Messung beruflicher Gratifikationskrisen. Zeitschrift für Differentielle und Diagnostische Psychologie 2004;25(4):227–38.

52. Mohr G, Rigotti T, Müller A. Irritation - ein Instrument zur Erfassung psychischer Beanspruchung im Arbeitskontext. Skalen- und Itemparameter aus 15 Studien. Zeitschrift für Arbeits- und Organisationspsychologie A&O 2005;49(1):44–8.

53. Löwe B, Wahl I, Rose M, Spitzer C, Glaesmer H, Wingenfeld K, et al. A 4-item measure of depression and anxiety: validation and standardization of the Patient Health Questionnaire-4 (PHQ-4) in the general population. J Affect Disord 2010;122(1-2):86–95. PubMed PMID: 19616305.

54. Igu NCN, Onyishi CN, Amujiri BA, Binuomote MO, Modebelu MN, Okafor IP, et al. Raising Leadership Self-Efficacy and Minimizing Organizational Burnout Among School Administrators in a GROW Model of Cognitive Behavioral Coaching. Journal of Leadership & Organizational Studies 2023;30(4):464–82.

55. Maslach C, Jackson SE. The measurement of experienced burnout. J Organ Behavior 1986;2:99–113.

56. Janka A, Adler C, Brunner B, Oppenrieder S, Duschek S. Biofeedback Training in Crisis Managers: A Randomized Controlled Trial. Appl Psychophysiol Biofeedback 2017;42(2):117–25. PubMed PMID: 28349228.

57. Lange S, Rowold J. Mindful leadership: Evaluation of a mindfulness-based leader intervention. Gr Interakt Org 2019;50(3):319–35.

58. Mohr G, Müller A, Rigotti T, Aycan Z, Tschan F. The Assessment of Psychological Strain in Work Contexts. European Journal of Psychological Assessment 2006;22(3):198–206.

59. Bohlmeijer E, Prenger R, Taal E, Cuijpers P. The effects of mindfulness-based stress reduction therapy on mental health of adults with a chronic medical disease: a meta-analysis. J Psychosom Res 2010;68(6):539–44. PubMed PMID: 20488270.

60. Michalak J, Zarbock G, Drews M, Otto D, Mertens D, Ströhle G, et al. Erfassung von Achtsamkeit mit der deutschen Version des Five Facet Mindfulness Questionnaires (FFMQ-D). Zeitschrift für Gesundheitspsychologie 2016;24(1):1–12.

61. Baer RA, Smith GT, Hopkins J, Krietemeyer J, Toney L. Using self-report assessment methods to explore facets of mindfulness. Assessment 2006;13(1):27–45. PubMed PMID: 16443717.

62. Rowold J, Poethke U. Fragebogen zur Integrativen Führung. Göttingen: Hogrefe; 2017.

63. Li J, Riedel N, Barrech A, Herr RM, Aust B, Mörtl K, et al. Long-Term Effectiveness of a Stress Management Intervention at Work: A 9-Year Follow-Up Study Based on a Randomized Wait-List Controlled Trial in Male Managers. Biomed Res Int 2017;2017:2853813. PubMed PMID: 29181392; PubMed Central PMCID: PMC5664277.

64. Siegrist J. Adverse health effects of high-effort/low-reward conditions. J Occup Health Psychol 1996;1(1):27–41. PubMed PMID: 9547031.

65. Siegrist J, Starke D, Chandola T, Godin I, Marmot M, Niedhammer I, et al. The measurement of effort-reward imbalance at work: European comparisons. Soc Sci Med 2004;58(8):1483–99. PubMed PMID: 14759692.

66. Herrmann C. International experiences with the Hospital Anxiety and Depression Scale—a review of validation data and clinical results. J Psychosom Res 1997;42(1):17–41. PubMed PMID: 9055211.

67. Vilagut G, Forero CG, Pinto-Meza A, Haro JM, Graaf R de, Bruffaerts R, et al. The mental component of the short-form 12 health survey (SF-12) as a measure of depressive disorders in the general population: results with three alternative scoring methods. Value in health: the journal of the International Society for Pharmacoeconomics and Outcomes Research 2013;16(4):564–73. PubMed PMID: 23796290.

68. Limm H, Gündel H, Heinmüller M, Marten-Mittag B, Nater UM, Siegrist J, et al. Stress management interventions in the workplace improve stress reactivity: a randomised controlled trial. Occup Environ Med 2011;68(2):126–33. PubMed PMID: 20833759.

69. Ly KH, Asplund K, Andersson G. Stress management for middle managers via an acceptance and commitment-based smartphone application: A randomized controlled trial. Internet Interventions 2014;1(3):95–101.

70. Goldberg D, Williams P. General Health Questionnaire (GHQ). Swindon, Wiltshire, UK: nferNelson; 2000.

71. Bass BM, Avolio BJ, Jung DI, Berson Y. Predicting unit performance by assessing transformational and transactional leadership. J Appl Psychol 2003;88(2):207–18. PubMed PMID: 12731705.

72. Martin A, Kilpatrick M, Scott J, Cocker F, Dawkins S, Brough P, et al. Protecting the Mental Health of Small-to-Medium Enterprise Owners: A Randomized Control Trial Evaluating a Self-Administered Versus Telephone Supported Intervention. Journal of Occupational and Environmental Medicine 2020;62(7):503–10. PubMed PMID: 32730026; PubMed Central PMCID: PMC7337118.

73. Andrews G, Slade T. Interpreting scores on the Kessler Psychological Distress Scale (K10). Aust N Z J Public Health 2001;25(6):494–7. PubMed PMID: 11824981.

74. Ronald C. Kessler, Catherine Barber, Arne Beck, Patricia Berglund, Paul D. Cleary, David McKenas, et al. The World Health Organization Health and Work Performance Questionnaire (HPQ). Journal of Occupational and Environmental Medicine [Internet] 2003 [cited 2023 Dec 3];45(2):156–74. Available from: http://www.jstor.org/stable/44997208.

75. Sanderson K, Tilse E, Nicholson J, Oldenburg B, Graves N. Which presenteeism measures are more sensitive to depression and anxiety? J Affect Disord 2007;101(1-3):65–74. PubMed PMID: 17156851.

76. Mellner C, Osika W, Niemi M. Mindfulness practice improves managers' job demands-resources, psychological detachment, work-nonwork boundary control, and work-life balance – a randomized controlled trial. IJWHM 2022;15(4):493–514.

77. Lilja JL, Frodi-Lundgren A, Hanse JJ, Josefsson T, Lundh L-G, Sköld C, et al. Five Facets Mindfulness Questionnaire—reliability and factor structure: a Swedish version. Cogn Behav Ther 2011;40(4):291–303. PubMed PMID: 21770845.

78. Munafò M, Patron E, Palomba D. Improving Managers' Psychophysical Well-Being: Effectiveness of Respiratory Sinus Arrhythmia Biofeedback. Appl Psychophysiol Biofeedback 2016;41(2):129–39. PubMed PMID: 26446978.

79. Spielberger CD, Pedrabissi L, Santinello M. STAI, state-trait anxiety inventory, Forma Y: Manuale. Firenze: Organizzazioni Speciali; 1996.

80. Ni D, Zheng X, Liang LH. How and when leader mindfulness influences team member interpersonal behavior: Evidence from a quasi-field experiment and a field survey. Human Relations 2023;76(12):1940–65.

81. Brown KW, Ryan RM. The benefits of being present: mindfulness and its role in psychological well-being. J Pers Soc Psychol 2003;84(4):822–48. PubMed PMID: 12703651.

82. Nübold A, van Quaquebeke N, Hülsheger UR. Be(com)ing Real: a Multi-source and an Intervention Study on Mindfulness and Authentic Leadership. J Bus Psychol 2020;35(4):469–88.

83. Neider LL, Schriesheim CA. The Authentic Leadership Inventory (ALI): Development and empirical tests. The Leadership Quarterly 2011;22(6):1146–64.

84. Reitz M, Waller L, Chaskalson M, Olivier S, Rupprecht S. Developing leaders through mindfulness practice. JMD 2020;39(2):223–39.

85. Sawyer AT, Tao H, Bailey AK. The Impact of a Psychoeducational Group Program on the Mental Well-Being of Unit-Based Nurse Leaders: A Randomized Controlled Trial. Int J Environ Res Public Health 2023;20(11). PubMed PMID: 37297639; PubMed Central PMCID: PMC10252280.

86. Stamm BH. Professional Quality of Life: Compassion Satisfaction and Fatigue Version 5 (ProQOL) [Internet]; 2009 [cited 2023 Dec 3]. Available from: https://proqol.org/.

87. Center for Victims of Torture (CVT). ProQOL: Professional Quality of Life [Internet] [cited 2023 Dec 3]. Available from: https://proqol.org/.

88. Thompson ER, Phua FTT. A Brief Index of Affective Job Satisfaction. Group & Organization Management 2012;37(3):275–307.

89. Shonin E, van Gordon W, Dunn TJ, Singh NN, Griffiths MD. Meditation Awareness Training (MAT) for Work-related Wellbeing and Job Performance: A Randomised Controlled Trial. Int J Ment Health Addiction 2014;12(6):806–23.

90. Health and Safety Executive. HSE Management Standards Work-Related Stress Indicator Tool. London; n.d.

91. Lovibond PF, Lovibond SH. PsycTESTS Dataset; 1995.

92. Russell SS, Spitzmüller C, Lin LF, Stanton JM, Smith PC, Ironson GH. Shorter can Also be Better: The Abridged Job in General Scale. Educational and Psychological Measurement 2004;64(5):878–93.

93. Welbourne TM, Johnson DE, Erez A. The Role-Based Performance Scale: Validity Analysis of a Theory-Based Measure. Academy of Management Journal 1998;41(5):540–55.

94. Vonderlin R, Müller G, Schmidt B, Biermann M, Kleindienst N, Bohus M, et al. Effectiveness of a mindfulness- and skill-based health-promoting leadership intervention on supervisor and employee levels: A quasi-experimental multisite field study. J Occup Health Psychol 2021;26(6):613–28. PubMed PMID: 34591521.

95. Herrmann-Lingen C, Buss U, Snaith P. Hospital anxiety and depression scale-German version (HADS-D): Huber; 2011.

96. Zigmond AS, Snaith RP. The hospital anxiety and depression scale. Acta Psychiatr Scand 1983;67(6):361–70. PubMed PMID: 6880820.

97. Franke F, Felfe J, Pundt A. The Impact of Health-Oriented Leadership on Follower Health: Development and Test of a New Instrument Measuring Health-Promoting Leadership. German Journal of Human Resource Management: Zeitschrift für Personalforschung 2014;28(1-2):139–61.

98. Vonderlin R, Schmidt B, Biermann M, Lyssenko L, Heinzel-Gutenbrunner M, Kleindienst N, et al. Improving Health and Reducing Absence Days at Work: Effects of a Mindfulness- and Skill-Based Leadership Intervention on Supervisor and Employee Sick Days. Mindfulness 2023;14(7):1751–66.

99. Wasylkiw L, Holton J, Azar R, Cook W. The impact of mindfulness on leadership effectiveness in a health care setting: a pilot study. J Health Organ Manag 2015;29(7):893–911. PubMed PMID: 26556157.

100. Anderson RJ. The leadership assessment circle profile. Industrial and Commercial Training 2006;38(4):175–84.

101. Anderson RJ. Agreement in self-other ratings of leader effectiveness: the role of demographics and personality. The Leadership Circle [Internet] 2012. Available from: www.theleadershipcircle.com/wp-content/uploads/2011/05/15_Agreement_In_Self.pdf.

102. Walumbwa FO, Avolio BJ, Gardner WL, Wernsing TS, Peterson SJ. Authentic Leadership: Development and Validation of a Theory-Based Measure†. Journal of Management 2008;34(1):89–126.

103. Yong JSJ, Park JFJ, Park Y, Lee H, Lee G, Rim S. Effects of Holy Name Meditation on the Quality of Life of Hospital Middle Manager Nurses in Korea: a 6-Month Follow-Up. Journal of continuing education in nursing 2020;51(5):215–24.

104. Maslach C, Jackson SE. The measurement of experienced burnout. J Organ Behavior 1981;2(2):99–113.

105. Kim KI, Kim JH, Won HT. Korean manual of symptom checklist-90-revision. Seoul, Korea: Chungang Aptitude; 1984.

106. Derogatis LR, Lazarus L. SCL-90—R, Brief Symptom Inventory, and matching clinical rating scales. In: Maruish ME, editor. The use of psychological testing for treatment planning and outcome assessment. Hillsdale: Lawrence Erlbaum; 1994. p. 217–18.

107. Chamiec-Case RR. Developing a tool to measure social workers’ perceptions regarding the extent to which they integrate their spirituality in the workplace [Unpublished doctoral dissertation]. New York: Fordham University; 2006.

108. Kouzes JM, Posner BZ. The leadership challenge: How to make extraordinary things happen in organizations. J-B Leadership Challenge. Hoboken, New Jersey: The Leadership Challenge a Wiley Brand; 2017. 378 p.

109. Żołnierczyk-Zreda D, Sanderson M, Bedyńska S. Mindfulness-based stress reduction for managers: a randomized controlled study. Occup Med (Lond) 2016;66(8):630–5. PubMed PMID: 27440398.

110. Williams S, Cooper CL. Measuring occupational stress: development of the pressure management indicator. J Occup Health Psychol 1998;3(4):306–21. PubMed PMID: 9805279.

111. Bradburn NM. The Structure of Psychological Well-Being. Chicago: Aldine; 1969.

112. Sterne JAC, Savović J, Page MJ, Elbers RG, Blencowe NS, Boutron I, et al. RoB 2: a revised tool for assessing risk of bias in randomised trials. BMJ 2019;2:l4898.

113. Sterne JA, Hernán MA, Reeves BC, Savović J, Berkman ND, Viswanathan M, et al. ROBINS-I: a tool for assessing risk of bias in non-randomised studies of interventions. BMJ 2016;355:i4919. PubMed PMID: 27733354; PubMed Central PMCID: PMC5062054.

114. Egger M, Davey Smith G, Schneider M, Minder C. Bias in meta-analysis detected by a simple, graphical test. BMJ 1997;315(7109):629–34. PubMed PMID: 9310563; PubMed Central PMCID: PMC2127453.

115. McGuinness LA, Higgins JPT. Risk-of-bias VISualization (robvis): An R package and Shiny web app for visualizing risk-of-bias assessments. Res Synth Methods 2021;12(1):55–61. PubMed PMID: 32336025.
